# Supplementary material for: Development of support material for health professionals who are implementing Shared Decision-making in breast cancer screening: validation using the Delphi technique
Source: BMJ Open. 2022 Feb 1;12(2):e052566. doi: 10.1136/bmjopen-2021-052566 (PMC8808455; doi:10.1136/bmjopen-2021-052566)
Supplement: Supplementary data [file bmjopen-2021-052566supp002.pdf]

Supplemental material
BMJ Publishing Group Limited (BMJ) disclaims all liability and resp
placed on this supplemental material which has been supplied by
BMJ Open

La participación de los profesionales de la salud en la Toma de Decisiones Compartida en el cribado de cáncer de mama

Manual de apoyo para implementación de la Toma de Decisiones Compartida

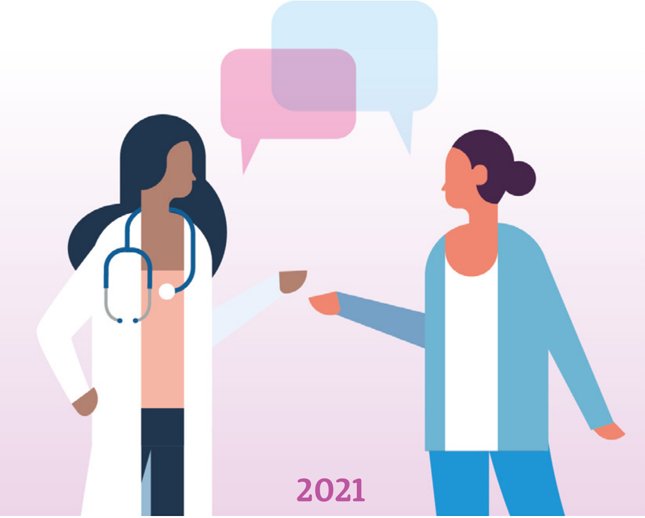

BMJ Publishing Group Limited (BMJ) disclaims all liability and resp
placed on this supplemental material which has been supplied by

Supplemental material
BMJ Publishing Group Limited (BMJ) disclaims all liability and resp
placed on this supplemental material which has been supplied by
BMJ Open

La participación de los profesionales de la salud en la Toma de Decisiones Compartida en el cribado de cáncer de mama

Manual de apoyo para implementación de la Toma de Decisiones Compartida

2021

AUTORES

María José Hernández-Leal[1,a,b,c], María José Pérez-Lacasta[2,a,c], Misericòrdia Carles-Lavila[3,a,b,c] on behalf of the ProShare Group.

1. PhD student, Msc and Bsc Nursing. Mail: mariajose.hernandez@urv.cat
2. PhD, Mail: mariajose.perez@urv.cat
3. PhD. Mail: misericordia.carles@urv.cat

a. Department of Economics, Universitat Rovira i Virgili.
b. Centre de Recerca en Economia i Sostenibilitat (ECO-SOS).
c. Grup de Recerca en Anàlisi Estadístic i Econòmic en Salut (GRAEES).

Equipo de investigación ProShare:

Misericòrdia Carles Lavila
Núria Codern-Bové
María José Hernández Leal
María José Pérez Lacasta
Roger Pla
Vanesa Ramos García
Carmen Vidal

Cómo citar este documento:

Hernández-Leal MJ, Carles-Lavila M, Pérez-Lacasta M. La participación de los profesionales de la salud en la Toma de Decisiones Compartida en el cribado de cáncer de mama: Manual de apoyo para implementación de la TDC. España: María José Hernández editor; 2021.

Supplemental material BMJ Publishing Group Limited (BMJ) disclaims all liability and resp placed on this supplemental material which has been supplied by BMJ Open

EVALUADORES EXTERNOS

Dra. Lilisbeth Perestelo-Pérez
Servicio de Evaluación y Planificación.
Dirección del Servicio Canario de la Salud, España.
Red de Investigación en Servicios de Salud en Enfermedades Crónicas (REDISSEC).

Dr. Victor Montori
Knowledge and Evaluation Research (KER) Unit.
Mayo Clinic - Minnesota, USA.

Dra. Montserrat Rué
Institut de Recerca Biomèdica de Lleida (IRBLLEIDA).
Departament de Ciències Mèdiques Bàsiques.
Universitat de Lleida, España.

DISEÑO

Miquel A. Fernández
www.mafsdisseny.com

FINANCIACIÓN

- The European Regional Development Fund (ERDF). European Union's Horizon 2020 research and innovation programme under the Marie Skłodowska-Curie grant agreement No. 713679 from the Universitat Rovira i Virgili (URV).
- Proyecto PI18/00773 "La colaboración de los profesionales sanitarios para incluir la toma de decisiones compartida en el programa de cribado de cáncer de mama" financiado por el Instituto de Salud Carlos III y cofinanciado por la Unión Europea (FEDER) "Una manera de hacer Europa".

BMJ Publishing Group Limited (BMJ) disclaims all liability and resp
placed on this supplemental material which has been supplied by

BMJ Publishing Group Limited (BMJ) disclaims all liability and resp[illegible] placed on this supplemental material which has been supplied by

ÍNDICE

OBJETIVO DEL MANUAL 6

¿A QUIÉN VA DIRIGIDO? 6

Introducción 7

Toma de decisiones compartida 11

1. ¿Qué es? 12
2. ¿Por qué es importante? 15
3. Las limitaciones del modelo 17
4. ¿Qué habilidades o competencias necesitan los profesionales sanitarios? 18
5. ¿Qué opinan los pacientes? 22

La toma de decisiones compartida en el cribado de cáncer de mama 25

1. El Programa de cribado 26
2. Implementación de la TDC en cribado de cáncer de mama 30
3. Autoevaluación del proceso de TDC 39

Referencias bibliográficas 43

OBJETIVO DEL MANUAL

Este manual pretende ser un documento de referencia que sirva de guía para el desarrollo e implementación de la **Toma de Decisiones Compartida (TDC)** en el cribado del cáncer de mama.

¿A QUIÉN VA DIRIGIDO?

A profesionales sanitarios de la comunidad autónoma de Catalunya relacionados con el cribado de cáncer de mama y que tengan contacto directo con las mujeres que deben participar en el programa.

Introducción

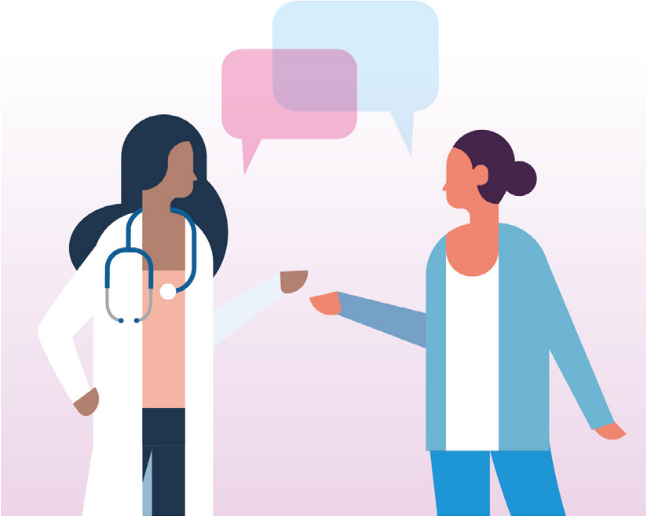

BMJ Publishing Group Limited (BMJ) disclaims all liability and resp
placed on this supplemental material which has been supplied by

El objetivo del **cribado de cáncer de mama** es detectar tempranamente un tumor, en una fase preclínica. Esto permite mejorar las opciones de tratamiento y disminuir la mortalidad[1,2]. A pesar de estos beneficios, el cribado puede producir también efectos adversos: falsos negativos, falsos positivos, sobrediagnóstico y sobretratamiento[2,3,4]. Ante el desconocimiento del grado en que afectarán los efectos positivos y negativos a cada mujer en la decisión del cribado, el modelo de **Toma de Decisiones Compartida** (TDC) permite que pacientes y profesionales de la salud disminuyan la incertidumbre de la decisión[5,6].

La TDC ha sido utilizada principalmente en los países occidentales para mejorar las decisiones en salud de acuerdo a las preferencias de los pacientes y la evidencia científica[7]. Así, en 2012 The European Patients' Forum inició la campaña *"nothing about me, without me"* (nada sobre mí, sin mí)[8] para involucrar a las personas en las decisiones sobre su salud[9].

En este contexto, algunas investigaciones han explorado cómo se desarrolla la TDC, así, por ejemplo, en España solo el 24% de los pacientes afirma haber tomado la decisión conjunta con su profe-

BMJ Publishing Group Limited (BMJ) disclaims all liability and resp
placed on this supplemental material which has been supplied by

sional sanitario considerando sus características y preferencias personales o sociales[10]. Esto evidencia la falta de estrategias para involucrar a las personas en su salud, debido a posibles deficiencias en las habilidades comunicativas y la inexistencia de vías de diálogo productivas entre los distintos actores del encuentro clínico[11].

Para mejorar las estrategias, la **Agencia de Calidad y Evaluación Sanitarias de Cataluña** (AQuAs) describe a las Herramienta de Ayuda a la Toma de Decisiones (HATD) como un elemento central para la TDC, aunque actualmente no dispone una de ellas para el cribado de cáncer de mama[12]. Sin embargo, un estudio reciente desarrolló una HATD[13].

A pesar de que en la actualidad ha habido un incremento de materiales destinados a las pacientes[14], son escasos aquellos que ayudan a los profesionales para incorporar las preferencias y los valores de las personas en la toma de decisiones en salud. En este sentido, este documento ofrece a los profesionales sanitarios evidencias científicas sobre la TDC para que sean aplicables al proceso de cribado de cáncer de mama.

BMJ Publishing Group Limited (BMJ) disclaims all liability and resp placed on this supplemental material which has been supplied by

Toma de decisiones compartida

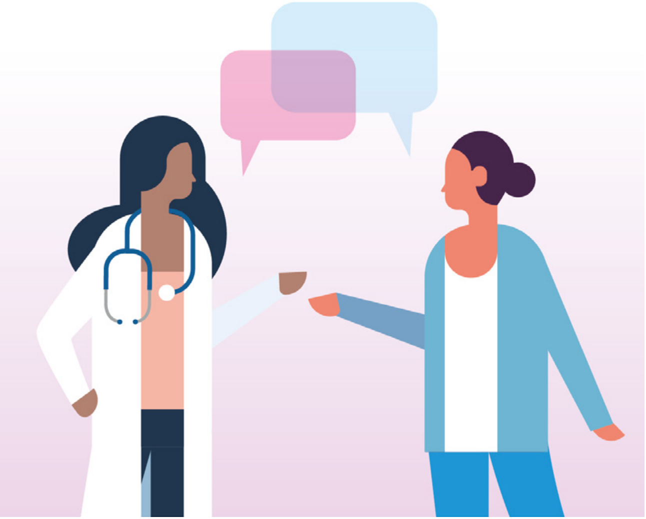

BMJ Publishing Group Limited (BMJ) disclaims all liability and resp[illegible] placed on this supplemental material which has been supplied by

1. ¿Qué es?

La Toma de Decisiones Compartida (TDC) surge en la década de los 60-70 y corresponde a un modelo de atención participativo ubicado entre el estilo de atención paternalista y el informativo[15,16].

Figura 1: Modelos de atención en salud

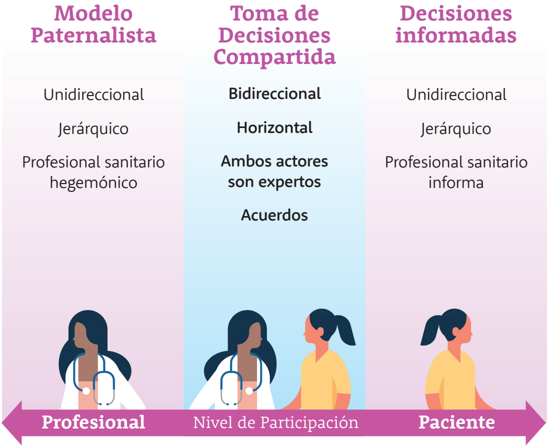

Fuente: Elaboración propia del grupo ProShare

La TDC fomenta la participación del paciente[17] para tomar una decisión conjunta con el profesional sanitario en relación con cambios en el estilo de vida, pruebas diagnósticas, tratamientos y acciones terapéuticas donde pueda existir algún grado de incertidumbre[18,19]. La TDC se desarrolla durante el encuentro clínico y ambos actores son considerados expertos: el paciente en su situación de salud, valores, creencias y preferencias; y el profesional sanitario en la evidencia científica e información de las opciones terapéuticas disponibles[20].

Figura 2: Rol de los participantes en el encuentro clínico

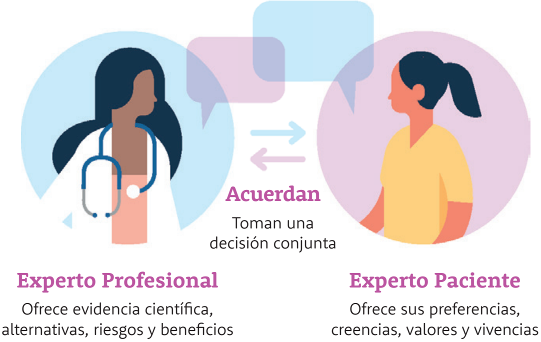

Fuente: Elaboración propia del grupo ProShare

BMJ Publishing Group Limited (BMJ) disclaims all liability and resp[illegible]
placed on this supplemental material which has been supplied by [illegible]

Por tanto, el objetivo de la TDC se centra en garantizar que las personas tomen decisiones sobre su salud cuando están suficientemente informadas[21]. Para lograrlo se requiere de una negociación continua entre ambos expertos centrando el diálogo en los valores, preferencias, circunstancias del paciente, así cómo en los beneficios, daños, riesgos y opciones terapéuticas ofrecidas por el profesional sanitario. Como resultado final de esta discusión se consigue personas más autónomas, un mayor nivel de compromiso y responsabilidad en su salud[20,22–24].

Figura 3: Elementos de la Toma de Decisiones Compartida

A) Intercambio de información entre el paciente y el profesional sanitario	B) Deliberación sobre las distintas opciones	**C) Tomar una decisión consensuada**

Fuente: Adaptación de Elwyn et al. 2012[20]

BMJ Publishing Group Limited (BMJ) disclaims all liability and resp
Supplemental material placed on this supplemental material which has been supplied by BMJ Open

2. ¿Por qué es importante?

La TDC se sustenta en el principio de autonomía de los pacientes. La Ley 21/2000 Derechos de Información relativos a la Salud, Autonomía del Paciente y Documentación Clínica protege su derecho a decidir libremente después de recibir la información adecuada entre las opciones clínicas disponibles[25]. Por tanto, los profesionales están legalmente sujetos al cumplimiento de este principio, y no puede limitarse a la voluntad del profesional.

Junto con lo anterior, la implementación de la TDC ha evidenciado una serie de beneficios en los pacientes, en los profesionales y también en los sistemas sanitarios[10, 24, 26, 27]:

- ✔ Aumenta la participación de los pacientes.
- ✔ Mejora la comunicación paciente-profesional sanitario.
- ✔ Mayor adherencia a tratamientos.
- ✔ Mejora los resultados biométricos en salud.
- ✔ Aumenta la satisfacción de los pacientes en la atención de salud.

BMJ Publishing Group Limited (BMJ) disclaims all liability and resp[illegible] placed on this supplemental material which has been supplied by

- Disminuye la preocupación y la ansiedad en los pacientes.
- Reduce el conflicto decisional de los pacientes cuando se enfrentan a tomar una decisión diagnóstica y/o terapéutica.
- Mejora el conocimiento de la enfermedad, las opciones diagnósticas y terapéuticas en los pacientes.
- Aumenta la precisión en la percepción del riesgo.
- Aumenta la elección de opciones más beneficiosas.
- Reduce el uso de tratamientos muy invasivos y costosos.
- Reduce la variabilidad injustificada en la práctica asistencial.
- Contribuye a la racionalización del uso de recursos del sistema sanitario.

BMJ Publishing Group Limited (BMJ) disclaims all liability and resp[illegible] placed on this supplemental material which has been supplied by

3. Las limitaciones del modelo

Aun existen pocos estudios que realicen un seguimiento prolongado a los pacientes para determinar con certeza cuál es el impacto de su aplicación a largo plazo. Por otro lado, existe la creencia entre los profesionales sanitarios que ellos ya aplican la SDM[28]. Sin embargo, algunos estudios han demostrado que esta presunción no se refleja en la práctica[29,30]. Finalmente, aunque los pacientes tienden a elegir las mismas opciones que si no se hubiese aplicado una SDM, se ha demostrado que, en el caso del cribado para el cáncer de mama, las mujeres valoran casi 5 veces más la reducción de la mortalidad que el riesgo de un sobrediagnóstico[31] y esta diferencia radica en un mayor conocimiento, en la adherencia al acuerdo, y la disminución del conflicto decisional.

4. ¿Qué habilidades o competencias necesitan los profesionales sanitarios?

Comunicar de forma equilibrada los riesgos y beneficios de cualquier opción terapéutica no es tarea fácil[26], para conseguirlo se han identificado dos tipos de competencias que deben desarrollar los profesionales sanitarios para aplicarlas en la TDC[32]:

4.1 Competencias relacionales

Son las habilidades que proporcionan un ambiente cómodo para que el paciente comparta sus preocupaciones. Para lograrlo el profesional debe tener un interés genuino en querer involucrarse, comprender el punto de vista del paciente y utilizar un lenguaje sencillo.

Entre las competencias del profesional destacan:

- Realizar una escucha activa.
- Respetar las decisiones tomadas por el paciente.
- Realizar preguntas abiertas.
- Generar en todo momento contacto visual.
- Dejar que los tiempos sean pautados por el paciente.
- Reconocer sus señales emocionales o verbales.
- Usar habilidades comunicacionales: el resumen, la clarificación, el reflejo, la empatía, entre otras[33].

4.2 Competencias de comunicación de riesgo

Son las habilidades que sirven para discutir con el paciente la incertidumbres y comunicar de forma efectiva los riesgos y beneficios de las diferentes opciones. Se debe evaluar la evidencia en relación a cada contexto particular, es decir, considerar los antecedentes personales: historia familiar, historia clínica y factores de riesgo o protectores que podrían aumentar o disminuir los beneficios/daños de las opciones[32].

Para esto se recomienda evitar el lenguaje técnico, adaptar la cantidad de información a las necesidades actuales del paciente, utilizar diagramas, comprobar la comprensión de la información ofrecida, incorporar valores del paciente a la evidencia, transmitir información objetiva, facilitar la participación y evaluar la información que ya dispone el paciente[34].

En resumen, para poseer una óptima relación con el paciente se resumen en las siguientes habilidades[33] (Figura 4).

Figura 4: Habilidades comunicativas

Escucha	Lenguaje	No verbal	Cultural	Actitudinal
Escucha general y activa	**Verbal:** tono apropiado y adaptado al nivel educativo<br><br>**Escrita:** comunicación clara y uso de material educativo	**Expresivo:** lenguaje corporal y contacto visual<br><br>**Receptivo:** responde a lenguaje corporal y emociones	Adaptar comunicación a la cultura, edad y enfermedad	Respetar las opiniones y derecho de decidir del paciente

Fuente: Adaptación de Laughlin T, Wetmore S, Allen T, Brailovsky C, Crichton T, Bethune C, Donoff M, Lawrence K. 2012[34].

BMJ Publishing Group Limited (BMJ) disclaims all liability and resp[illegible] placed on this supplemental material which has been supplied by [illegible]

Si quieres **profundizar en habilidades comunicacionales** revisa los siguientes enlaces:

- Shared decision making and the concept of equipoise: the competences of involving patients in healthcare choices.

  https://bjgp.org/content/bjgp/50/460/892.full.pdf

- The role of physician–patient communication in promoting patient–participatory decision making.

  https://www.ncbi.nlm.nih.gov/pmc/articles/PMC5060521/

- La comunicación médico-paciente: ¿Cuáles son las habilidades efectivas?

  https://scielo.conicyt.cl/pdf/rmc/v138n8/art16.pdf

- Video Decisions Compartides, Generalitat de Catalunya.

  http://decisionscompartides.gencat.cat/ca/inici

5. ¿Qué opinan los pacientes?

Un estudio desarrollado en España del año 2012 identificó que al 60% de los pacientes les hubiese gustado que el profesional sanitario les pidiese su opinión, aunque no se les animó a hacerlo. Además, la mayoría hubieran deseado recibir más información de la que se les entregó[35]. Otros estudios en el cribado de cáncer de mama han demostrado que sólo entre un 8% a 10% de las mujeres recibieron información respecto al sobrediagnóstico[36].

Los pacientes consideran que el profesional sanitario debe involucrarse en las decisiones de sus pacientes, es decir, no abandonarlos en el proceso de decisión[37]. En 2013, otro estudio determinó cuáles son los elementos que más valoran los pacientes en la TDC, siendo los más significativos el rol comunicativo del profesional sanitario, la percepción de una escucha comprensiva, la sensación de una preocupación real por su salud y por sus necesidades, una conversación acorde al contexto y la constatación de un dominio de la información[38].

La TDC fortalece la relación profesional sanitario-paciente y la alianza terapéutica porque el hecho de participar activamente en las decisiones de su salud, disminuye la incertidumbre, aumenta el conocimiento y la posibiidad de manejar mejor su enfermedad[37]. En definitiva, los pacientes sienten mayor tranquilidad.

BMJ Publishing Group Limited (BMJ) disclaims all liability and resp
placed on this supplemental material which has been supplied by

 BMJ Publishing Group Limited (BMJ) disclaims all liability and resp... placed on this supplemental material which has been supplied by

La toma de decisiones compartida en el cribado de cáncer de mama

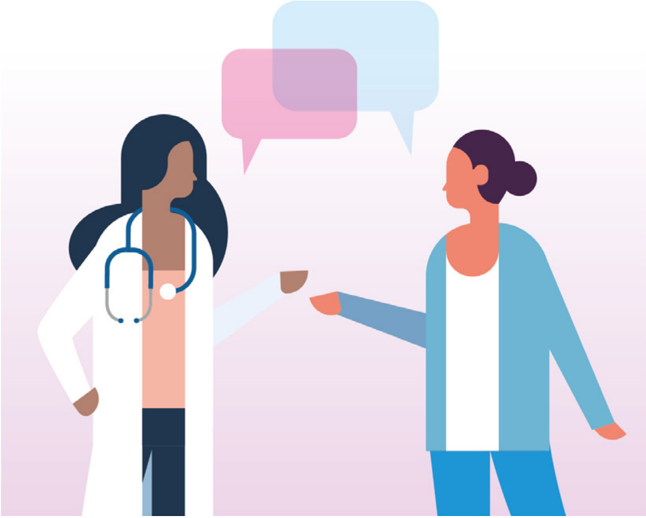

BMJ Publishing Group Limited (BMJ) disclaims all liability and resp[illegible] placed on this supplemental material which has been supplied by

1. El Programa de cribado

1.1 Cribado poblacional en Cataluña

El Plan Estratégico del *Pla Director d'Oncologia* de la Generalitat de Catalunya menciona como objetivo disminuir el impacto del cáncer de mama en la población mediante el cribado[39]. Sin embargo, no hace referencia a la forma de incorporar a la mujer en las decisiones de su salud, a pesar de que su participación es uno de los pilares del Plan de Salud de Cataluña 2016-2020[40].

Actualmente, cada Programa dependiente del *Pla Director d'Oncologia*, se realiza cada dos años y en base al Registro Central de Asegurados (RCA), la captación de las mujeres entre los 50 y los 69 años mediante una carta dirigida a su domicilio particular. En ella, se les invita a realizar, de forma gratuita, una mamografía en un centro de salud previamente asignado[41]. Este mecanismo no incorpora un espacio de contacto entre el profesional sanitario y la mujer, donde ella pueda resolver sus dudas o inquietudes, ni permite ofrecerle información su-

BMJ Publishing Group Limited (BMJ) disclaims all liability and resp
placed on this supplemental material which has been supplied by

ficiente para hacerla partícipe en la decisión sobre su participación -o no- en el programa de cribado.

Para subsanar esta falta de contacto se requiere un cambio en la organización y en los medios de información a las mujeres[42]. Así, el uso de HATD ha demostrado ser un apoyo para el profesional sanitario y para las mujeres en el momento de tomar una decisión conjunta respecto al cribado.

Si quieres **profundizar en HATD** revisa los siguientes enlaces:

- Documento *Desarrollo de Herramientas de Ayuda para la Toma de Decisiones Compartida derivadas de las recomendaciones de las Guías de Práctica Clínica*.
- Sitios web PyDeSalud: *https://pydesalud.com/toma-de-decisiones-compartidas/*
- Sitio web de The Ottawa Hospital: *https://decisionaid.ohri.ca/AZsearch.php?criteria=screening*
- Video demostrativo *Una Demostración - Toma de decisiones compartidas - Mayo Clinic: https://www.youtube.com/watch?v=qwyx7yAP5zA&t=4s*

 BMJ Publishing Group Limited (BMJ) disclaims all liability and resp placed on this supplemental material which has been supplied by

1.2 ¿Por qué aplicar la TDC al cribado de cáncer de mama?

Las pruebas diagnósticas para la detección precoz del cáncer de mama han tomado fuerza como estándar de salud pública al reconocer la reducción de la mortalidad, así de cada 200 mujeres que se realizan mamografías de cribado cada dos años, entre los 50 y los 69 años, 1 mujer se salva gracias a la detección precoz del tumor y 40 necesitarán pruebas adicionales para descartar el cáncer[13]. Sin embargo, estudios recientes demuestran que existe una escasa o nula percepción de sus daños o efectos adversos.

Los principales riesgos atribuidos al cribado de cáncer de mama son los falsos positivos, falsos negativos y el sobrediagnóstico[19]. Este último concepto se define como aquellos tumores que crecen tan lentamente que nunca llegarían a ser un problema de salud e incluso desaparecerían de forma espontánea, sin necesidad de tratamiento. Actualmente, se desconoce qué tipo de lesiones progresarán y cuáles no, por tanto, se ofrece tratamiento

BMJ Publishing Group Limited (BMJ) disclaims all liability and resp[illegible] placed on this supplemental material which has been supplied by

a todas las mujeres diagnosticadas de cáncer de mama (lo que se conoce como sobretratamiento)[13] que provoca una sensación de fragilidad y vulnerabilidad en la mujer, intolerancia a la incertidumbre, vinculación a procedimientos de mayor riesgo, como son las biopsias[43] y, finalmente, se traduce en un aumento del gasto sanitario[8].

Ante la incertidumbre entre los beneficios y efectos adversos, se recomienda el uso de TDC con el fin de definir en base a la evidencia científica actual y los valores de las mujeres la decisión de participar o no en el cribado.

Para mayor información sobre las definiciones, incidencia, estadísticas de riesgo, beneficios y efectos adversos del cribado de cáncer de mamas se pueden encontrar en la HATD desarrollada el año 2016[13].

BMJ Publishing Group Limited (BMJ) disclaims all liability and resp placed on this supplemental material which has been supplied by

2. Implementación de la TDC en cribado de cáncer de mama

Antes de iniciar el proceso de TDC se debe establecer una relación de confianza basada en la empatía, facilitar una comunicación fluida y de calidad[23,44]. El proceso debe ser deliberativo[21], es decir, las mujeres toman conciencia que tomarán una decisión y que puede requerir más de un encuentro clínico[21]. Además, este debe ser dinámico ya que las fases deben adaptarse a las necesidades, inquietudes y prioridades de cada mujer[45].

Una vez proporcionada la información, se debe explorar explícitamente si la mujer desea desempeñar un rol activo o pasivo en la decisión[23,46] de lo contrario puede inducir a un rol pasivo en las mujeres[17,23,46]. Sin embargo, no es impedimento para corroborar durante todo el encuentro clínico el rol que desean desempeñar, ya que éste puede cambiar de uno activo a uno pasivo o viceversa en el transcurso de la conversación.

BMJ Publishing Group Limited (BMJ) disclaims all liability and resp[illegible] placed on this supplemental material which has been supplied by

Existen algunos factores que promueven la participación, entre el profesional y la mujer, la motivación de los profesionales de la salud para involucrar a la persona en sus decisiones, la percepción de que la TDC producirá un impacto positivo en el proceso clínico, la alta alfabetización de la mujer o el propio deseo de ella de ser parte activa en las decisiones que afectan a su salud[26,47]. Sin embargo, la falta del tiempo en el encuentro clínico, la edad avanzada de las mujeres, personas con dificultad de comunicarse en el idioma del profesional, el bajo nivel socioeconómico de las mujeres, su baja alfabetización[23,26,46,47] y la presencia de patologías de salud mental[23,43] limitan la TDC.

BMJ Publishing Group Limited (BMJ) disclaims all liability and resp placed on this supplemental material which has been supplied by

2.1 Fases y modelo *"Three-talk"*

Las tres fases que componen este modelo son[48]:

FASE 1
Crear equipo

El principal objetivo de esta fase es comunicar la necesidad de tomar una decisión en equipo, cuyos integrantes son el profesional sanitario y la mujer. Aquí se comunican los objetivos de la decisión, por qué se debe tomar (presencia de factores de riesgo personal) y las alternativas disponibles basadas en la evidencia. El profesional debe enfatizar en que (a) la mujer pude decidir no tomar una decisión en ese momento y solicitar el apoyo de otros actores como familiares u otros especialistas y (b) debe ser receptivo a las reacciones que puede generar en la mujer el enfrentarse a esta decisión. Por tanto, debe recalcar que la acompañará en el proceso hasta que se sienta segura para llevar a cabo la decisión.

Supplemental material placed on this supplemental material which has been supplied by the author(s) BMJ Publishing Group Limited (BMJ) disclaims all liability and resp

Figura 5: Crear equipo

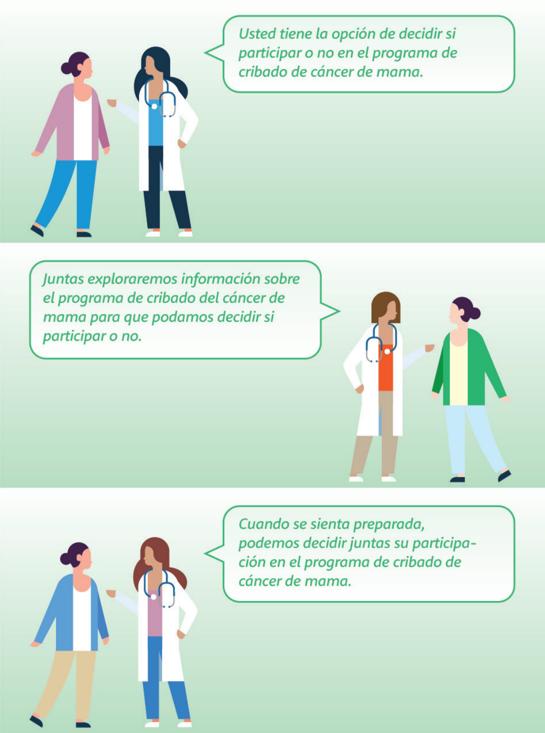

Fuente: Elaboración propia del grupo ProShare.

FASE 2

Plantear las opciones y explorar preferencias

El principal objetivo de esta fase es informar claramente, según las características de cada mujer, los efectos adversos y beneficios del cribado. Para esto debe explorar sus valores, sus preocupaciones, expectativas y preferencias iniciales (prioridades basadas en los conocimientos preexistentes o ideas preconcebidas respecto al cribado[21]). Además ampliar en detalle las opciones, considerando riesgos y beneficios. De esta forma, las preferencias iniciales pasarán a ser preferencias informadas (preferencias personales basadas en los valores una vez que se ha asegurado la comprensión de los riesgos y beneficios más relevantes del cribado[21]).

Para explicar los riesgos específicos se recomienda utilizar alguna HATD, ya que mejorará la comprensión de la información incluso en mujeres con baja alfabetización[13].

Supplemental material BMJ Publishing Group Limited (BMJ) disclaims all liability and resp[illegible] placed on this supplemental material which has been supplied by [illegible] *BMJ Open*

Figura 6: Plantear las opciones y explorar preferencias

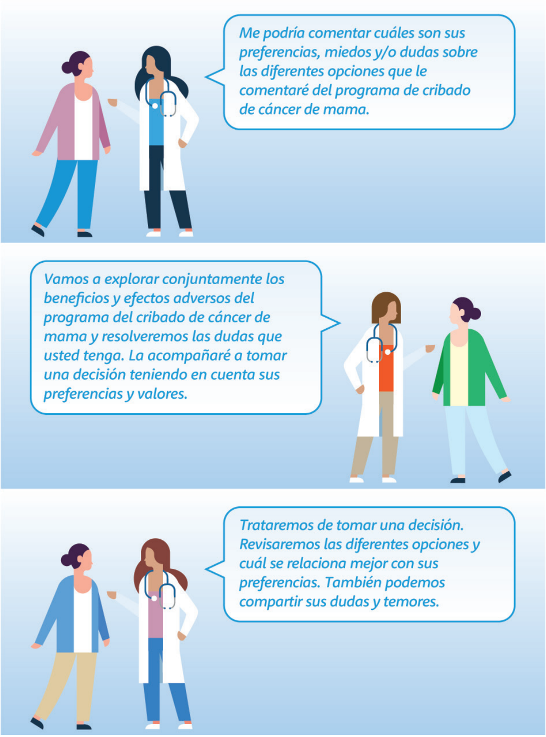

Fuente: Elaboración propia del grupo ProShare.

FASE 3
Tomar una decisión compartida

En esta fase se argumentan las alternativas y se toma una decisión respecto a la participación en el cribado de cáncer de mama[48]. El profesional debe reforzar la idea de acompañamiento en la decisión. Además, debe dar el tiempo suficiente que permita a la mujer reflexionar en torno a sus prioridades, incluso incluyendo la idea de diferirla para otro momento o delegarla en el profesional; en este último caso se recomienda identificar los elementos que le impiden hacerlo por ella misma. Finalmente, debe confirmar la decisión y acordar un plan de acción[23,46] y seguimiento que permita un circuito de retroalimentación entre el profesional y la mujer[45,46].

BMJ Publishing Group Limited (BMJ) disclaims all liability and resp[illegible] placed on this supplemental material which has been supplied by [illegible]

Figura 7: Tomar una decisión compartida

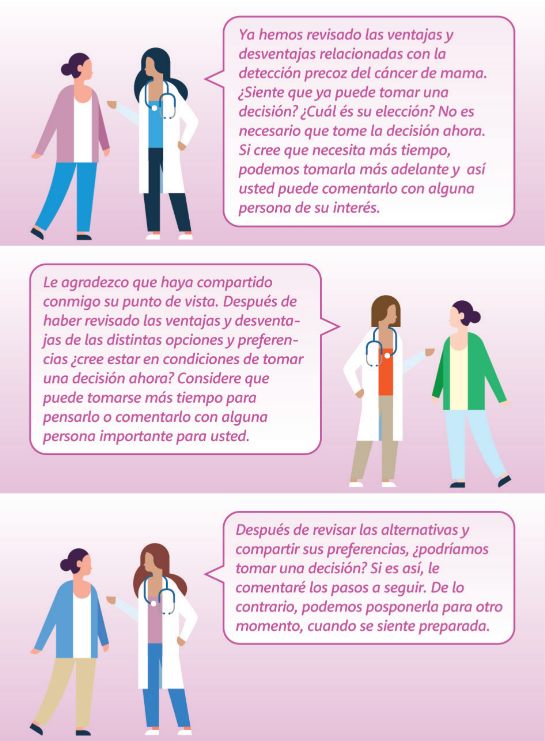

Fuente: Elaboración propia del grupo ProShare.

Supplemental material BMJ Publishing Group Limited (BMJ) disclaims all liability and resp[illegible] placed on this supplemental material which has been supplied by [illegible] BMJ Open

Figura 8: Fases de la Toma de Decisiones Compartida

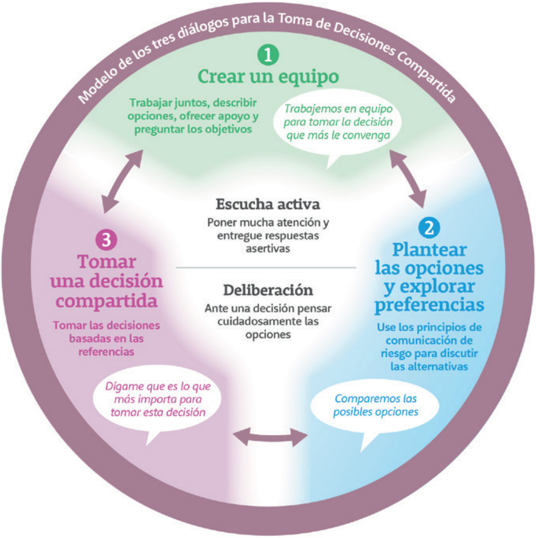

Fuente: Adaptación de Three-talk model of shared decision making. Elwyn G, et al 2017. Uso autorizado por el autor[48].

3. Autoevaluación del proceso de TDC

El instrumento SDM-Q-doc[49] es una encuesta de autoevaluación para profesionales sanitarios que permite medir el nivel de participación que se le ha ofrecido a la mujer para tomar decisiones. Este instrumento está compuesto por nueve ítems que deben ser valorados por el profesional dentro de seis alternativas; desde totalmente en desacuerdo (valor 1) a totalmente de acuerdo (valor 6).

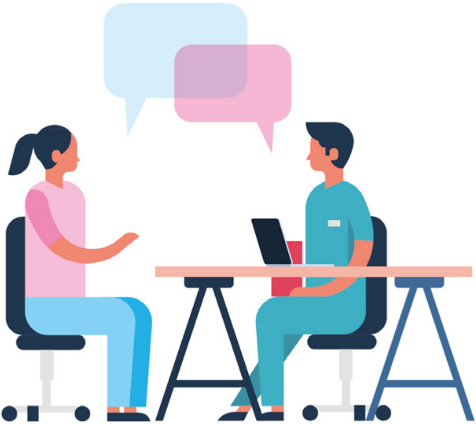

Criterio	Puntos*					
	1	2	3	4	5	6
Informé claramente a la mujer de la necesidad de tomar una decisión sobre su participación en el cribado de cáncer de mama	☐	☐	☐	☐	☐	☐
Pregunté a la mujer de forma precisa cómo le gustaría participar en la toma de decisiones	☐	☐	☐	☐	☐	☐
Informé a la mujer que existe la opción de participar o no en el cribado	☐	☐	☐	☐	☐	☐
Expliqué claramente a la mujer las ventajas y desventajas de cada opción	☐	☐	☐	☐	☐	☐
Ayudé a la mujer a entender toda la información sobre beneficios y efectos adversos	☐	☐	☐	☐	☐	☐
Pregunté a la mujer qué opción prefería	☐	☐	☐	☐	☐	☐
La mujer y yo hemos valorado ampliamente todas las opciones	☐	☐	☐	☐	☐	☐
La mujer y yo hemos escogido conjuntamente una opción	☐	☐	☐	☐	☐	☐
La mujer y yo nos hemos puesto de acuerdo sobre el seguimiento de su atención sanitaria posterior	☐	☐	☐	☐	☐	☐

* La puntuación va de: "Totalmente en desacuerdo" (1) a "Totalmente de acuerdo" (6)

Fuente: SDM-Q-doc adaptado al cribado de cáncer de mama.

Procedimiento y Resultados de la autoevaluación

Para conocer el resultado se deben sumar los puntos obtenidos en cada sección, identificando así las fases del modelo "*Three-talk*".

Fase de la TDC	Puntos	Interpretación
Fase 1 **"Crear equipo"**		3 a 12 puntos: sin adherencia a la TDC<br>13 a 18 puntos: adherente a la TDC
Fase 2 **"Plantear opciones y explorar preferencias"**		3 a 12 puntos: sin adherencia a la TDC<br>13 a 18 puntos: adherente a la TDC
Fase 3 **"Tomar una decisión"**		3 a 12 puntos: sin adherencia a la TDC<br>13 a 18 puntos: adherente a la TDC
**Puntuación total:**		**9 a 27:** Falta de adherencia a la TDC<br>**28 a 36:** Indiferencia a la TDC<br>**37 a 45:** Leve adherencia a la TDC<br>**46 a 54:** Fuerte adherencia a favor de la TDC

Supplemental material BMJ Publishing Group Limited (BMJ) disclaims all liability and resp[illegible] placed on this supplemental material which has been supplied by [illegible] BMJ Open

La interpretación y puntos de corte se basaron en la evidencia de Pollard, Bansback y Bryan (2015)[5]. Se dividieron los puntos totales (54 puntos) según los porcentajes de corte descritos; >80% "Fuerte adherencia del profesional a favor de la TDC"; 60-80% "Leve adherencia del profesional a la TDC"; 40-60% "Indiferencia del profesional a la TDC" y <40% "Falta de adherencia del profesional a la TDC". Por otro lado, los puntos de corte por cada fase definen como "adherente" aquellos superiores al 60% y "sin adherencia" a la TDC el resto, de un total de 15 puntos (3 al 18). Finalmente, la división por fases corresponde a una adaptación de la escala SDM-Q-doc contrastándola con las características del modelo "*Three-talk*"[48].

Se recomienda realizar esta autoevaluación con periodicidad ya que permite a los profesionales sanitarios identificar los puntos fuertes y débiles en cuanto a la forma en que incorporan la participación en salud de las mujeres. De este modo, se facilita focalizar los esfuerzos en mejorar los aspectos más débiles con formación y entrenamiento, y finalmente hacer un seguimiento de los avances conseguidos.

BMJ Publishing Group Limited (BMJ) disclaims all liability and resp
Supplemental material placed on this supplemental material which has been supplied by
BMJ Open

Referencias bibliográficas

1. Organización Mundial de la Salud. (WHO) Control del cáncer de mama; 2020 [citado 30 de junio de 2020]. Available from: https://www.who.int/topics/cancer/breastcancer/es/index3.html
2. Febles G. Revisión de los beneficios del screening del cáncer de mama y análisis de las controversias. *Rev. Imagenol.* [Internet] 2015;19 (1)9-18. Available from: https://www.webcir.org/revistavirtual/articulos/2016/4_noviembre/ur/febles_esp.pdf
3. Day N. Overdiagnosis and breast cancer screening. *Breast Cancer Res* [Internet]. 2005;7(5):228–9. Available from: http://www.ncbi.nlm.nih.gov/pubmed/16168144
4. Nehmat H. Overdiagnosis of breast cancer in population screening: does it make breast screening worthless? *Cancer Biol Med* [Internet]. 2017;14(1):1–8. Available from: http://www.cancerbiomed.org/index.php/cocr/article/view/1003
5. Pollard S, Bansback N, Bryan S. Physician attitudes toward shared decision making: A systematic review. *Patient Educ Couns* [Internet]. 2015; 98(9):1046-57. Available from: https://www.sciencedirect.com/science/article/abs/pii/S0738399115002256?via%3Dihub
6. Munthe C, Sandman L, Cutas D. Person Centred Care and Shared Decision Making: Implications for Ethics, Public Health and Research. *Health Care Anal* [Internet]. 2012;20(3):231–49. Available from: http://link.springer.com/10.1007/s10728-011-0183-y
7. Barry M, Edgman-Levitan S. Shared Decision Making — The Pinnacle of Patient-Centered Care. *N Engl J Med* [Internet]. 2012;366(9):780–1. Available from: http://www.nejm.org/doi/10.1056/NEJMp1109283
8. Coulter A, Collins A. Making shared decision-making a reality. No decision about me, without me [Internet]. The King's Fund. Edwina Rowling, editor. London; 2011. Available from: www.kingsfund.org.uk
9. European patients' forum. 150 Million reasons to act; *EPF's Patients' Manifesto for the European Parliament and Commission*. [Internet]. 2009. Available from: https://www.eu-patient.eu/globalassets/events/old/manifesto/epf_manifesto.pdf

Supplemental material placed on this supplemental material which has been supplied by BMJ Publishing Group Limited (BMJ) disclaims all liability and resp BMJ Open

10. Confederación Crohn y Colitis Ulcerosa. Se incumple el modelo de decisiones compartidas con los pacientes [Internet]. Available from: https://www.accuesp.com/se-incumple-el-modelo-de-decisiones-compartidas-con-los-pacientes

11. Ministerio de Sanidad, Consumo y Bienestar Social de España. Marco Estratégico para la Atención Primaria y Comunitaria [Internet]. 2019. Available from: https://www.mscbs.gob.es/profesionales/proyectosActividades/docs/Marco_Estrategico_APS_25Abril_2019.pdf

12. Agencia de Calidad y Evaluación Sanitarias de Cataluña (AQuAs). Estado de situación de las herramientas de ayuda a la toma de decisiones clínicas en Cataluña. [Internet]. 2020 [citado 30 de junio de 2020]. Available from: http://aquas.gencat.cat/ca/ambits/avaluacio-tecnologies-qualitat/guies/eines_ajut_presa_decisions_catalunya/

13. Toledo-Chavarri A, Rué M, Codern-Bove N, Carles-Lavila M, Perestelo-Perez L, Perez-Lacasta MJ, and Feijoo-Cid M. A Qualitative Study on a Decision Aid for Breast Cancer Screening: Views from Women and Health Professionals. *Eur J Cancer Care (Engl)* [Internet]. 2017. 26(3). Available from: https://onlinelibrary.wiley.com/doi/abs/10.1111/ecc.12660

14. Grupo Español de Investigación en Cáncer de Mama. Guía GEICAM de Práctica Clínica: Para el Diagnóstico y Tratamiento del Cáncer de Mama Metastásico. Edición: Comuniland S.L. Madrid; 2015. Available from: https://portal.guiasalud.es/wp-content/uploads/2019/06/gpc_538_geicam_cancer_mama_resumida.pdf

15. Charles C, Gafni A, Whelan T. Self-reported use of shared decision-making among breast cancer specialists and perceived barriers and facilitators to implementing this approach. *Health Expect* [Internet]. 2004 Dec;7(4):338–48. Available from: http://www.ncbi.nlm.nih.gov/pubmed/15544686

16. Barr P, Scholl I, Bravo P, Faber M, Elwyn G, McAllister M. Assessment of Patient Empowerment - A Systematic Review of Measures. *PLoS One* [Internet]. 2015;10(5). Available from: http://journals.plos.org/plosone/article/file?id=10.1371/journal.pone.0126553&type=printable

Supplemental material placed on this supplemental material which has been supplied by BMJ Publishing Group Limited (BMJ) disclaims all liability and resp... BMJ Open

17. Elwyn G, Edwards A, Kinnersley P, Grol R. Shared decision making and the concept of equipoise: the competences of involving patients in healthcare choice. *Br J Gen Pract* [Internet]. 2000;50:892–7. Available from: https://bjgp.org/content/bjgp/50/460/892.full.pdf

18. Participa y decide sobre tu salud. ¿Qué es la toma de decisiones compartida? [Internet]. 2020 [citado 30 de junio de 2020]. Available from: https://www.pydesalud.com/toma-de-decisiones-compartidas/

19. Marmot M, Altman D, Cameron D, Dewar J, Thompson S, Wilcox M. The benefits and harms of breast cancer screening: An independent review. *Br J Cancer* [Internet]. 2013;108(11): 2205–2240. Available from: https://www.ncbi.nlm.nih.gov/pmc/articles/PMC3693450/

20. Reyes A. Toma de decisiones compartidas: por una medicina cuidadosa y amable [Internet]. Parte 1. *Por una Medicina Interna de Alto Valor*. 2018. Available from: https://medicinainternaaltovalor.fesemi.org/el-universo-del-paciente/toma-de-decisiones-compartidas-por-una-medicina-cuidadosa-y-amable-parte-1/

21. Elwyn G, Frosch D, Thomson R, Joseph-Williams N, Lloyd A, Kinnersley P, et al. Shared decision making: a model for clinical practice. *J Gen Intern Med* [Internet]. 2012;27(10):1361–7. Available from: http://www.ncbi.nlm.nih.gov/pubmed/22618581

22. Peralta L, Ruiz J, Castillo R. La participación del paciente en la toma de decisiones en las consultas de atención primaria. *Universidad de Cantabria*. 2010; Available from: http://www.tesisenred.net/bitstream/handle/10803/10705/TesisLPM.pdf?sequence=1

23. Información farmacoterapéutica de la Comarca. Participación del paciente en la toma de decisiones. *INFAC* [Internet]. 2014;22(3):12–7. Available from: https://www.euskadi.eus/contenidos/informacion/cevime_infac_2014a/es_def/adjuntos/INFAC_Vol_22_n_3_Decisiones_compartidas.pdf

24. Bravo P, Contreras A. Perestelo-Pérez L, Pérez-Ramos J, Málaga G. En busca de una salud más participativa: Compartiendo decisiones de salud. *Rev Peru Med Exp Salud Publica*. 2013;30(4):691-7.

25. Boletín Oficial del Estado. Ley 14/1986 de 2 de febrero, General de Sanidad. BOE de 21/2000. [Internet]. Available from: https://www.boe.es/boe/dias/2001/02/02/pdfs/A04121-04125.pdf

Supplemental material BMJ Publishing Group Limited (BMJ) disclaims all liability and resp[illegible] placed on this supplemental material which has been supplied by [illegible] BMJ Open

26. Gravel K, Légaré F, Graham I. Barriers and facilitators to implementing shared decision-making in clinical practice: a systematic review of health professionals' perceptions. *Implementation Sci*. 2006;1(16). Available from: http://www.implementationscience.com/content/1/1/16

27. Légaré F, Ratté S, Stacey D, Kryworuchko J, Gravel K, Graham ID, et al. Interventions for improving the adoption of shared decision making by healthcare professionals. *Cochrane Database of Systematic Reviews* [Internet]. Chichester, UK: John Wiley & Sons, Ltd; 2010. p. CD006732. Available from: http://www.ncbi.nlm.nih.gov/pubmed/20464744

28. Stiggelbout AM, Pieterse AH, De Haes JC. Shared decision making: Concepts, evidence, and practice. *Patient Educ Couns*. 2015;(10):1172-9. doi: 10.1016/j.pec.2015.06.022. Epub 2015 Jul 15. PMID: 26215573.

29. Hernández-Leal M, Perestelo-Pérez L, Bravo P. Percepción de los profesionales de salud para la Toma de Decisiones Compartida en Atención Primaria. *Revista chilena de medicina familiar*. forthcoming.

30. Couët N, Desroches S, Robitaille H, Vaillancourt H, Leblanc A, Turcotte S, Elwyn G, Légaré F. Assessments of the extent to which health-care providers involve patients in decision making: a systematic review of studies using the OPTION instrument. *Health Expect*. 2015;18(4):542-61. doi: 10.1111/hex.12054.

31. Pérez-Lacastra, Carles-Lavila M, Codern-Bové N, Cardona-Cardona A, Rué-Monné M. Eliciting women's preferences for the design of a breast cancer screening program. *Patient Educ Couns*. forthcoming.

32. Légaré F, Moumjid-Ferdjaoui N, Drolet R, Stacey D, Härter M, Bastian H, et al. Core competencies for shared decision making training programs: insights from an international, interdisciplinary working group. *J Contin Educ Health Prof* [Internet]. 2013;33(4):267–73. Available from: http://www.ncbi.nlm.nih.gov/pubmed/24347105

33. Ford S, Schofield T, Hope T. What are the ingredients for a successful evidence-based patient choice consultation? A qualitative study. *Soc Sci Med* [Internet]. 2003;56:589–602. Available from: https://pubmed.ncbi.nlm.nih.gov/12570976/

34. Laughlin T, Wetmore S, Allen T, et al. Defining competency-based evaluation objectives in family medicine: communication skills. *Can Fam Physician* [Internet]. 2012;58(4):e217–e224. Available from: https://www.ncbi.nlm.nih.gov/pmc/articles/PMC3325474/.

35. Ruiz R, Peralta L, Pérula L, Olloqui Mundet J, Carrión T, Sobrino A, et al. Opiniones y percepciones de los pacientes sobre su participación en la toma de decisiones en las consultas de medicina de familia. *Aten Primaria* [Internet]. 2012;44(1):5–10. Available from: https://www.sciencedirect.com/science/article/pii/S0212656711001545?via%3Dihub

36. Hersch, J. Jansen & K. McCaffery (2018) Decision-making about mammographic screening: pursuing informed choice, Climacteric, 21:3, 209-213, DOI: 10.1080/13697137.2017.1406912

37. Moreau A, Carol L, Dedianne M, Dupraz C, Perdrix C, Lainé X, et al. What perceptions do patients have of decision making (DM)? Toward an integrative patient-centered care model. A qualitative study using focus-group interviews. *Patient Educ Couns* [Internet]. 2012:1;87(2):206–11. Available from: https://www.sciencedirect.com/science/article/abs/pii/S0738399111004599

38. Thorne S, Oliffe JL, Stajduhar KI. Communicating shared decision-making: Cancer patient perspectives. Patient Educ Couns [Internet]. 2013;90(3):291–6. Available from: https://www.sciencedirect.com/science/article/abs/pii/S0738399112000912

39. Generalitat de Catalunya. Departament de Salut. Pla director 2017. [Internet]. Available from: http://salutweb.gencat.cat/ca/ambits_actuacio/linies_dactuacio/estrategies_salut/cancer/pla_director/

40. Generalitat de Catalunya. Departament de Salut. Pla director D'Oncologia 2016-2020: Un sistema centrat en la persona: públic, universal i just. 2016. [Internet]. Available from: http://salutweb.gencat.cat/web/.content/_departament/pla-de-salut/Pla-de-salut-2016-2020/documents/Pla_salut_Catalunya_2016_2020.pdf

41. Institut Català d'Oncologia. Detecció Precoç de Càncer de Mama. [Internet]. 2020 [citado 30 de junio de 2020]. Available from: http://ico.gencat.cat/ca/el-cancer/programes_de_deteccio_precoc/programa_de_deteccio_precoc_de_cancer_de_mama/index.html

Supplemental material placed on this supplemental material which has been supplied by BMJ Publishing Group Limited (BMJ) disclaims all liability and resp BMJ Open

42. Servicio de epidemiología y Prevención del cáncer (SEPC). Esquema funcional del Programa de Detección Precoz del Cáncer de Mama. [Internet]. 2020 [citado 30 de junio de 2020]. Available from: https://epicancer.cat/prevencion_cancer_mama.

43. Coll-Benejam T, Bravo-Toledo R , Marcos-Calvo M, Astier-Peña M. Impacto del sobrediagnóstico y sobretratamiento en el paciente, el sistema sanitario y la Sociedad. *Aten Primaria.* [Internet]. 2018;50(S2):86-95. Available from: https://www.elsevier.es/es-revista-atencion-primaria-27-articulo-impacto-del-sobrediagnostico-sobretratamiento-el-S0212656718305158

44. Rowland K, Politi M. Shared Decision-Making and the Patient-Provider Relationship. In: Handbook of Health Decision Science [Internet]. New York, NY: Springer New York; 2016. p. 181–92. Available from: http://link.springer.com/10.1007/978-1-4939-3486-7_14

45. Elwyn G, Petronella Catharina Anna Vermunt N. Goal-Based Shared Decision-Making: Developing an Integrated Model. *Journal of Patient Experience*. 2019;1-9. Available from: https://journals.sagepub.com/doi/full/10.1177/2374373519878604

46. Barani M, Kopitowski K. Toma de decisiones compartidas: centrando los cuidados médicos realmente en nuestros pacientes. *Rev Hosp Ital BAires* [Internet]. 2013;33(2):60–4. Available from: https://www.hospitalitaliano.org.ar/multimedia/archivos/noticias_attachs/47/documentos/14745_60-64-HI-33-2-2BARANI_A.pdf

47. Hernández-Leal, MJ, Pérez-Lacasta MJ, Feijoo-Cid M, Ramos-García V, Carles-Lavila M. Healthcare professionals' behaviour regarding the implementation of shared decision-making in screening programmes: A systematic review. Patient Educ Couns [Internet]. 2021. https://doi.org/10.1016/j.pec.2021.01.032

48. Elwyn G, Duran M, Song J, Aarts J, J Barr P, Berger Z, etc al. A three-talk model for shared decision making: multistage consultation process. *BMJ*. 2017;359. [Internet]. Available from: https://www.bmj.com/content/359/bmj.j4891.long

49. De las Cuevas C, Perestelo-Perez L, Rivero-Santana A, Cebolla-Martí A, Scholl I, Härter M. Validation of the Spanish version of the 9-item Shared Decision-Making Questionnaire. *Health Expect* [Internet]. 2015;18(6):2143–53. Available from: https://pubmed.ncbi.nlm.nih.gov/24593044/

BMJ Publishing Group Limited (BMJ) disclaims all liability and resp
placed on this supplemental material which has been supplied by

BMJ Publishing Group Limited (BMJ) disclaims all liability and resp

La participación de los profesionales de la salud en la Toma de Decisiones Compartida en el cribado de cáncer de mama

Manual de apoyo para implementación de la Toma de Decisiones Compartida

2021
