# Supplementary material for: Development of support material for health professionals who are implementing Shared Decision-making in breast cancer screening: validation using the Delphi technique
Source: BMJ Open. 2022 Feb 1;12(2):e052566. doi: 10.1136/bmjopen-2021-052566 (PMC8808455; doi:10.1136/bmjopen-2021-052566)
Supplement: Supplementary data [file bmjopen-2021-052566supp005.pdf]

# *Guía práctica de implementación de la TDC para profesionales sanitarios*

ProShare
SHARED DECISION MAKING IN HEALTH

*“Ahora sabemos que usted puede decidir qué hacer en relación al cribado, vamos a hablar cuáles son las característi-cas del cribado para que usted pueda conocer sus opciones”*

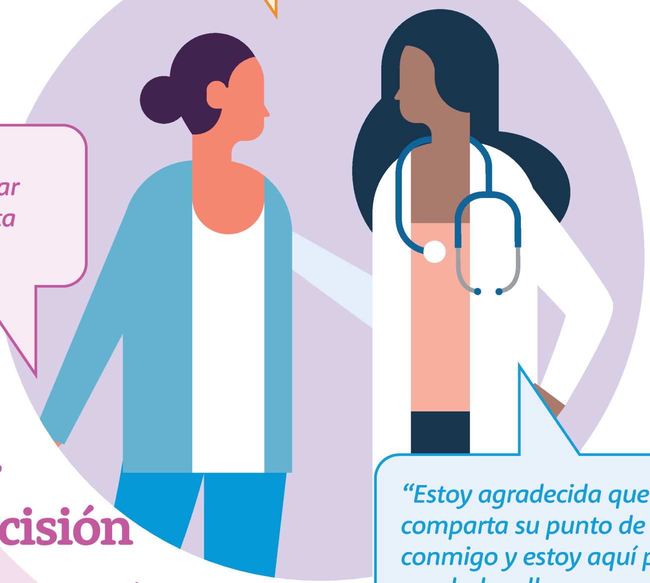

## Comunicar la necesidad de tomar una decisión

## 1 Crear equipo

- Introduzca la posibilidad de tomar decisiones acerca de su salud
- Comente los factores de riesgo y los que le afectan en particular
- Resalte que la acompañará en todo momento y puede contar con el apoyo de familiares u otros profesionales

**Factores de Riesgo** Se estima que 1 de cada 8 mujeres en España padecerán cáncer de mamá a lo largo de su vida. Los factores de riesgo son: edad, antecedentes familiares y personales de cáncer de mama, alteraciones de la mama, hormonal, radioterapia y estilo de vida.

Fuente: AECC: https://www.aecc.es/es/ todo-sobre-cancer/tipos-cancer/cancer-mama/mas-informacion/evolucion-cancer-mama

## 2 Plantear las opciones

### Informar de la opción de acudir o no a la mamografía

- Explore los conocimientos de la mujer sobre la mamografía
- Introduzca efectos adversos y beneficios de la mamografía a través de una Herramienta de Ayuda a la Toma de Decisiones (HATD)
- Considere las preferencias, creencias, valores y miedos de la mujer sobre la mamografía
- Resuma las opciones y compruebe si la mujer ha comprendido la nueva información

*“Estoy agradecida que comparta su punto de vista conmigo y estoy aquí para ayudarla a llegar a una buena decisión. Vamos a resumir sus preferencias y comprobar si aún tiene alguna duda”*

**La mamografía** es utilizada como *gold stándar* para el cribado a nivel internacional; ha demostrado disminuir del 20 al 30% la mortalidad, pero también genera falsos negativos, falsos positivos y sobrediagnóstico; su función es adelantar el diagnóstico sin esperar que aparezcan síntomas de la enfermedad. La mamografía es una radiografía de dos proyecciones (cráneo-caudal y látero-oblicua) en cada pecho que posteriormente se analiza con una doble lectura (anexo 1: HATD).

*"¿Cree estar en condiciones de tomar la decisión o necesita más tiempo?”*

## 3 Tomar una decisión

### Tomar una decisión compartida respecto a la mamografía

- Dé el tiempo necesario para permitir la reflexión
- Aclare las dudas y valore las preferencias
- Diseñe un plan de seguimiento de la decisión

**La Toma de Decisiones Comparti-das (TDC)** es un modelo de atención participativo ubicado entre un estilo de atención paternalista e informa-tivo que fomenta la participación de las mujeres para tomar una decisión con el profesional sanitario cuando existe algún grado de incertidum-bre. La TDC se desarrolla durante el encuentro clínico, ambos actores se consideran como expertos: la mujer en su situación de salud, valores, creencias y preferencias. El profesio-nal en la evidencia científica y en cómo dar información de las opcio-nes terapéuticas disponibles.

BMJ Publishing Group Limited (BMJ) disclaims all liability and responsibility arising from any reliance placed on this supplemental material which has been supplied by the author(s)

# *Guía práctica de implementación de la TDC para profesionales sanitarios*

ProShare
SHARED DECISION MAKING IN HEALTH

## Resultados

| Fase de la TDC | Puntos | Interpretación |
|---|---|---|
| Fase 1 **"Crear equipo"** | | 13 a 18 puntos: adherente a la TDC<br>3 a 12 puntos: sin adherencia a la TDC |
| Fase 2 **"Plantear opciones"** | | 13 a 18 puntos: adherente a la TDC<br>3 a 12 puntos: sin adherencia a la TDC |
| Fase 3 **"Tomar una decisión"** | | 13 a 18 puntos: adherente a la TDC<br>3 a 12 puntos: sin adherencia a la TDC |
| **Puntuación total:** | | **46 a 54:** Fuerte adherencia a favor de la TDC<br>**37 a 45:** Leve adherencia a la TDC<br>**28 a 36:** Indiferencia a la TDC<br>**9 a 27:** Falta de adherencia a la TDC |

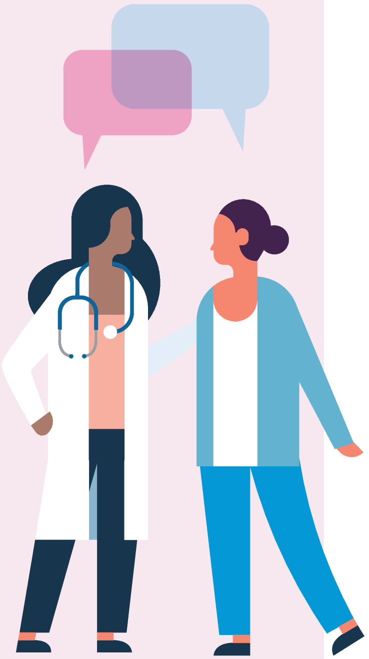

| Criterio | Puntos* | | | | | |
|---|---|---|---|---|---|---|
| | 1 | 2 | 3 | 4 | 5 | 6 |
| Informé claramente a la mujer de la necesidad de tomar una decisión sobre su participación en el cribado de cáncer de mama | ☐ | ☐ | ☐ | ☐ | ☐ | ☐ |
| Pregunté a la mujer de forma precisa cómo le gustaría participar en la toma de decisiones | ☐ | ☐ | ☐ | ☐ | ☐ | ☐ |
| Informé a la mujer que existe la opción de participar o no en el cribado | ☐ | ☐ | ☐ | ☐ | ☐ | ☐ |
| Expliqué claramente a la mujer las ventajas y desventajas de cada opción | ☐ | ☐ | ☐ | ☐ | ☐ | ☐ |
| Ayudé a la mujer a entender toda la información sobre beneficios y efectos adversos | ☐ | ☐ | ☐ | ☐ | ☐ | ☐ |
| Pregunté a la mujer qué opción prefería | ☐ | ☐ | ☐ | ☐ | ☐ | ☐ |
| La mujer y yo hemos valorado ampliamente todas las opciones | ☐ | ☐ | ☐ | ☐ | ☐ | ☐ |
| La mujer y yo hemos escogido conjuntamente una opción | ☐ | ☐ | ☐ | ☐ | ☐ | ☐ |
| La mujer y yo nos hemos puesto de acuerdo sobre el seguimiento de su atención sanitaria posterior | ☐ | ☐ | ☐ | ☐ | ☐ | ☐ |

* La puntuación va de: "Totalmente en desacuerdo" (1) a "Totalmente de acuerdo" (6)

*De las Cuevas C, Perestelo-Perez L, Rivero-Santana A, Cebolla-Martí A, Scholl I, Härter M. Validation of the Spanish version of the 9-item Shared Decision-Making Questionnaire. Health Expect [Internet]. 2015;18(6):2143–53. Available from: http://www.ncbi.nlm.nih.gov/pubmed/24593044*
