# Supplementary material for: Development of support material for health professionals who are implementing Shared Decision-making in breast cancer screening: validation using the Delphi technique
Source: BMJ Open. 2022 Feb 1;12(2):e052566. doi: 10.1136/bmjopen-2021-052566 (PMC8808455; doi:10.1136/bmjopen-2021-052566)
Supplement: Author's manuscript [file bmjopen-2021-052566.draft_revisions.pdf]

BMJ Open is committed to open peer review. As part of this commitment we make the peer review history of every article we publish publicly available.

When an article is published we post the peer reviewers' comments and the authors' responses online. We also post the versions of the paper that were used during peer review. These are the versions that the peer review comments apply to.

The versions of the paper that follow are the versions that were submitted during the peer review process. They are not the versions of record or the final published versions. They should not be cited or distributed as the published version of this manuscript.

BMJ Open is an open access journal and the full, final, typeset and author-corrected version of record of the manuscript is available on our site with no access controls, subscription charges or pay-per-view fees (http://bmjopen.bmj.com).

If you have any questions on BMJ Open's open peer review process please email info.bmjopen@bmj.com

BMJ Open

Development of support material for health professionals who are implementing Shared Decision-Making in breast cancer screening: Validation using Delphi methodology

Journal:	*BMJ Open*
Manuscript ID	bmjopen-2021-052566
Article Type:	Original research
Date Submitted by the Author:	20-Apr-2021
Complete List of Authors:	Hernández Leal, María José; Universitat Rovira i Virgili, Economic<br>Codern-Bové, Núria; Universitat Autònoma de Barcelona<br>Pérez-Lacasta, María José; University Rovira i Virgili, Economica<br>Cardona, Angels<br>Vidal, Carmen; Catalan Institute of Oncology, Cancer Prevention and Control Programme<br>Carles-Lavila, Misericòrdia; Universitat Rovira i Virgili, Department of Economics
Keywords:	MEDICAL EDUCATION & TRAINING, Breast tumours < ONCOLOGY, PREVENTIVE MEDICINE, PRIMARY CARE, PUBLIC HEALTH, QUALITATIVE RESEARCH

SCHOLARONE™
Manuscripts

I, the Submitting Author has the right to grant and does grant on behalf of all authors of the Work (as defined in the below author licence), an exclusive licence and/or a non-exclusive licence for contributions from authors who are: i) UK Crown employees; ii) where BMJ has agreed a CC-BY licence shall apply, and/or iii) in accordance with the terms applicable for US Federal Government officers or employees acting as part of their official duties; on a worldwide, perpetual, irrevocable, royalty-free basis to BMJ Publishing Group Ltd ("BMJ") its licensees and where the relevant Journal is co-owned by BMJ to the co-owners of the Journal, to publish the Work in this journal and any other BMJ products and to exploit all rights, as set out in our licence.

The Submitting Author accepts and understands that any supply made under these terms is made by BMJ to the Submitting Author unless you are acting as an employee on behalf of your employer or a postgraduate student of an affiliated institution which is paying any applicable article publishing charge ("APC") for Open Access articles. Where the Submitting Author wishes to make the Work available on an Open Access basis (and intends to pay the relevant APC), the terms of reuse of such Open Access shall be governed by a Creative Commons licence – details of these licences and which Creative Commons licence will apply to this Work are set out in our licence referred to above.

Other than as permitted in any relevant BMJ Author's Self Archiving Policies, I confirm this Work has not been accepted for publication elsewhere, is not being considered for publication elsewhere and does not duplicate material already published. I confirm all authors consent to publication of this Work and authorise the granting of this licence.

For peer review only

Original Research Article

Title: Development of support material for health professionals who are implementing Shared Decision-Making in breast cancer screening: Validation using Delphi methodology

María José Hernández-Leal[1,a,b,c], Núria Codern-Bové[2,d], María José Pérez-Lacasta[3,a,c], Angels Cardona[4,e], Carmen Vidal-Lancis[5,f], Misericòrdia Carles-Lavila[6,a,b,c] on behalf of the ProShare Group

1. PhD student, MSc and BSc Nursing. Email: mariajose.hernandez@urv.cat
2. PhD. Email: nuriacodern@euit.fdsll.cat
3. PhD. Email: mariajose.perez@urv.cat
4. MSc. Email: angels.cardona@areaq.net
5. MPH. Email: cvidal@iconcologia.net
6. PhD. Email: misericordia.carles@urv.cat

a. Department of Economics, Universitat Rovira i Virgili.
b. Research Centre on Economics and Sustainability (ECO-SOS).
c. Research Group on Statistics, Economic Evaluation and Health (GRAEES).
d. Terrassa University School of Nursing and Occupational Therapy (EUIT), Universitat Autònoma de Barcelona, Terrassa, Spain.
e. Area Q: Evaluation and Research in the Field of Social Sciences and Health.
f. Cancer Prevention and Control Program, Catalan Institute of Oncology-IDIBELL, L'Hospitalet de Llobregat, Spain,

ProShare group: Misericòrdia Carles-Lavila, Núria Codern-Bové, María José Hernández-Leal, María José Pérez, Roger Pla, Vanesa Ramos, Carmen Vidal.

Corresponding author:
Misericòrdia Carles-Lavila, Department of Economics, Universitat Rovira i Virgili, Avenida Universitat 1, 43204, Reus, Spain. Email: misericordia.carles@urv.cat

Word count: 4000

For peer review only

ABSTRACT

Background: No documentation currently exists that provides health professionals with training in Shared Decision-Making (SDM) for breast cancer screening. We have therefore developed a handbook and clinical practice guide to this topic, based on the Three-talk model.

Objective: To evaluate a handbook and clinical practice guide aimed at healthcare professionals, focused on the application of Shared Decision-Making to breast cancer screening.

Design: A study using Delphi methodology in three rounds of questions. The 20 participants were SDM experts and health professionals who provide care for women in the field of breast cancer prevention in Spain. The criterion established for consensus was a coefficient of concordance (Cc) above 75, for questions using a Likert scale of 1 to 6 with a cut-off point equal to or greater than 4.

Results: Participants considered the Three-talk model suitable for the screening context. The handbook's sections and level of detail were considered satisfactory (Cc=90). The summary provided by the clinical practice guide was considered necessary (Cc=75), as was the self-assessment tool for professionals (Cc=85). Content was added: addressing the limitations of the SDM model; extending the number of example dialogues for health professionals to three; providing supplementary resources on using Patients Decisions Aids (PtDAs) and adding references on communication skills.

Conclusions and aplications: The first handbook and clinical practice guide providing unique SDM support material for health professionals have been developed. Both aim to familiarise professionals with the SDM model, thereby fostering women's participation in the decision on whether to be screened for breast cancer. The evaluation concluded that the handbook is useful and innovative but, to facilitate implementation, practical strategies and a plan for piloting it are needed.

Keywords: Shared Decision-Making; breast cancer screening; health professionals.

STRENGTHS AND LIMITATIONS OF THIS STUDY

- Development of a handbook and a clinical practice guide to Shared Decision-Making for breast cancer screening.
- Adaptation of the Three-talk model to breast cancer screening.
- Participation of professionals in validating the design of the support materials.
- Facilitating the application of a person-centred model to the screening context.

For peer review only

BACKGROUND

Breast cancer (BC) screening programmes have undergone extensive development in the European Union over the past few decades (1). The aim of these programmes is early detection of malignant tumours in women (2). Screening is the most effective type of examination identified to date. Its main benefit is reducing mortality (3) and improving survival of women aged over 50 years (4). Of every 200 women who receive screening mammograms every two years, aged between 50 and 69 years, one woman is saved thanks to early detection of the tumour (5). Nevertheless, like any clinical tool, it has adverse effects: false positives, false negatives and overdiagnosis that, in term, is associated with overtreatment (5-9). It is still not possible to determine which women will experience the positive and negative effects of screening, and to what extent. The Shared Decision-Making model therefore enables health professionals and women to deliberate over the best choice on the basis of scientific evidence, and women's preferences and values, in order to come to a shared decision in contexts of uncertainty (10-11).

In Spain, the public BC screening programme has been active since the 1990s (12). The programme currently falls under the Oncology Master Plan (*Plan Director de Oncología*) in Catalonia (13). However, while there are strategies for incorporating women's values and preferences into the decision on whether or not to have the examination, there is no associated framework on how to put them into practice (14). For BC screening, the Breast Cancer Detection Programme (*Programa de Detección del Cáncer de Mama*) sends women between 50 and 69 years a letter informing them of the time and date at which they should attend their local centre to have a mammogram (15). The programme achieves a high level of coverage, but it fails to incorporate an opportunity for the woman and the professional to exchange information and enter into dialogue on the decision.

In order to promote the women's participation, several research teams have developed projects that involve women in making the decision on screening. In 2017, Toledo-Chavari and colleagues created a Patient decision aids (PtDAs) (16) (Annexes 1), consisting of a trifold leaflet that provides balanced information on the benefits and adverse effects, for the professionals and women to use during the clinical consultation. However, on the basis of the barriers and enabling factors cited in the literature (17-19), the researchers decided that it was not enough to use the PtDAs alone, concluding that SDM training material aimed at health professionals was also needed.

Working within the framework of the ProShare Study, our research team has therefore developed a handbook (Annexes 2) and clinical practice guide (20) (Annexes 3) aimed at health professionals who have direct relationships with women. These documents provide guidance on performing SDM in BC screening and on how to progress towards professional practice that promotes the active participation of women.

According to the literature, SDM in BC screening requires at least three elements: providing the patient with information and education, interpersonal communication between doctor and patient, and a decision (21). Given its inclusion of these three elements, it was decided to use the Three-talk model, adapting its three steps to BC screening: 1) Team talk; 2) Option talk and exploring preferences; 3) Decision talk (22).

For peer review only

METHODS

Delphi Methodology

A Delphi study was conducted. Delphi methodology enables experts to reach agreements on specific topics through intuitive thinking, in a virtual, anonymous and confidential space. Experts meet under these conditions when it is difficult to meet in person due to economic, geographic or time-related constraints (23-24). According to the literature, experts can be grouped into two broad categories: *Subjects (Su)* – people who would use the instrument in their profession; and *Specialists (Sp)* – people who have knowledge about the subject due to their academic and/or professional experience (23-24).

Participants

The handbook and clinical practice guide, entitled 'The participation of health professionals in Shared Decision-Making in breast cancer screening' (*La participación de los profesionales de la salud en la Toma de Decisiones Compartida en el cribado de cáncer de mama*) (Appendix 2) (20), were developed by the ProShare research team. The first version was produced with the participation of three researchers with experience in Shared Decision-Making and BC screening, who acted as external reviewers, and two health professionals, who produced the plan for piloting the questionnaire online (using a Google form).

The inclusion criteria for participants were as follows:

- *Subjects*: a) health professionals, preferably from primary care services, who provide direct care to women through breast cancer prevention activities, and b) who have at least five years' experience (25) in the Spanish health system.
- *Specialists*: a) international-level researchers whose research career has focused on the Shared Decision-Making model, and b) who are proficient in Spanish (given that the handbook has been produced in this language). Preference was given to individuals who had developed educational support material for professionals.

Sampling

Two sampling strategies were used to recruit participants: convenience sampling for the health professionals and snowball sampling for the specialists.

Recruitment

Invitations were sent by email to 43 potential participants, 30 of whom accepted.

Data collection

Three rounds of questionnaires were administered between July and October 2020. The aim of the questionnaires was to evaluate the usefulness of the topics, relevance of the content and document design. In the first and second rounds, open questions were given, using a Likert scale of 1 to 6, in which 1 was 'completely disagree' and 6 was 'completely agree'. In the last round, responses were dichotomous. The second and third rounds (R2 and R3, respectively) were developed on the basis of the elements about which no agreement had been reached in the previous round.

Data analysis

The researchers (MJH-MC-MJP-NC-AC) analysed participants' responses at the end of each round, considering responses whose score on the Likert scale was 4 or above to be positive. Agreement was determined to be reached when the coefficient of concordance (Cc) was greater

For peer review only

than 75 (26). For R3, the criteria established by Martínez (2003) were considered in order to bring the Delphi process to a close (27).

This research was approved by the Medicinal Product Research Ethics Committee (CEIm) of the Institut d'Investigació Sanitària Pere Virgili (Pere Virgili Health Research Institute). Informed consent was secured, which stated that participants accepted the conditions of participation upon agreeing to respond to the questionnaire. These conditions specified that responses were confidential and would only be used for the purposes of this research.

RESULTS

Of the 30 professionals who initially agreed to participate, 20 (66.6%) went on to respond in the first round (R1), 16 (53.3%) in the second (R2) and 17 (56.6%) in the third (R3) (Figure 1). In R1, the mean age of the experts was 46.6 years (SD 10.25), 75% were female, 65% were doctors, 70% worked in the public sector and they had an average of 19 years' (SD 9.69) experience (Table 1).

Figure 1: Flow diagram of participation in each round
Table 1: Characteristics of the participants

Round 1

R1 was designed to achieve two objectives: evaluating the content and the design of the support material. For this purpose, participants were asked 33 Likert-scale questions, 1 multiple-choice question and 6 open questions on the handbook and were given 2 Likert-scale questions and 4 open questions on the clinical practice guide (Table 2).

Table 2: R1 responses

A Cc greater than 75 was recorded for 32 of the Likert-scale questions and the minimum Cc was not reached for only 3 of them. These questions concluded that Figure 5, entitled 'Flow diagram of the Early Detection of Breast Cancer programme', was clear (Cc=60) and useful (Cc=70). The same applied to the question that determined Figure 6 – Team talk (page 34) – to be clear (Cc=75). These questions were incorporated into R2.

In the multiple-choice question, participants were asked which section of the handbook should be edited: 10 responded 'none'; 5 chose the section entitled 'Which skills or competencies do health professionals need?'; 3 selected the 'Screening programme' section, and 2 chose the 'Introduction'.

In their open responses, most participants considered the initiative to be positive and thought that it would enable health professionals to access information on SDM through use of the Three-talk model in BC screening (Box 1). However, one of the participants suggested using the Agency for Healthcare Research and Quality model.

Box 1: Response to the question: Are the steps based on "Three-talk" suitable for the application of SDM in breast cancer screening? Please explain briefly

For peer review only

P3 (R1): *Yes, it shows how the health professional can implement SDM in a three-step process in a brief, practical and easy-to-read way. It describes the characteristics that differentiate each step, and specific examples of implementation in breast cancer screening.*

The participants also provided some suggestions to modify the handbook. The most frequently cited concerned the length of the handbook, recommending reducing the content (Box 2) and incorporating example dialogues, communication skills (Box 3) and instructions for using the PtDAs. The comments were incorporated into the questions in R2.

Box 2: Response to the question: How would you improve the elements selected in the previous question?

P7: *I think that the handbook is very long, which may reduce motivation to read it.*
P6: *Very long and it doesn't show how to use the tool.*

Box 3: Response to the question: What other content would you include in the clinical practice guide?

P3: *Provide more information or example dialogues on how to use communication skills. This last [point] if the health professionals don't have a grounding or training in active listening, motivational interviewing, empathy, reflection, etc.*
P10: *I'd go into greater depth on relationship-building skills and give a few links to where they can find exercises to train themselves [in this].*

Finally, in response to the question on whether the dialogues in each step represent their objective, most participants agreed ('Team talk' step, n=10; 'option talk' step, n=7; 'Decision talk' step, n=12) and made suggestions on the wording of the dialogues. Suggestions were also made to adapt the name of the original *the Three-talk* steps to one that was more representative of the screening context. All the suggestions were incorporated into R2 to be approved or rejected by the other participants.

Only one of the questions evaluating the clinical practice guide did not reach the minimum Cc established: 'Do you consider a guide that concisely summarises the SDM steps to be necessary?' (*¿Cree necesaria una guía que resuma de forma rápida las fases de la TDC?*) (Cc=75). This question was incorporated into R2. In the open questions, the participants suggested changing the wording of the step 1 dialogues (n=3) and incorporating a review of communicative skills (Box 4); the same applied to step 2, but participants added a comment about using relative risks instead of absolute ones (n=1) (Box 5).

Box 4: Response to the question: What elements would you change in step 1: 'Team talk'?

P3: *I'd include a few reviews, such as [on] active listening and deliberation. Perhaps using a phrase like 'Remember to pay close attention and give assertive responses (active listening), and to think the options through carefully for the decision (deliberation)'.*

For peer review only

Box 5: Response to the question: What elements would you change in step 2: 'Option talk?

P15: *Change relative risks to absolute risks.*

They also proposed: eliminating the definition of SDM for step 3 in the guide (n=4), incorporating a brief clarification noting that women may also consult other people for support with making the decision (n=3) and mentioning the possibility of reversing the decision (n=4) (Box 6). Between 6 and 8 people stated that they would not makes any change to steps 1, 2 or 3.

Box 6: Response to the question: What elements would you change in step 3: 'Decision talk'?

P11: *I'd add the possibility of reversing the decision; I'd take out the explanation about SDM.*

Finally, in the last question – 'What other content would you include in the clinical practice guide?' – the participants reiterated the need to include a review of communication skills (n=3) and one of them proposed changing the self-assessment to use either the ASQ3 or the CollaboRATE instrument.

Round 2

R2 was structured around the open-question responses and included the elements about which agreement had not been reached in the previous round. Thirteen Likert-scale questions, 5 multiple-choice question and 6 open questions were produced on the handbook. For the clinical practice guide, 2 Likert-scale questions and 5 open questions were included (Table 3).

Table 3: R2 responses

Of the 13 Likert-scale questions, only 3 reached a score of Cc>75. These underlined the need to: reduce the length of the handbook (Cc=81.3), create a clinical practice guide to accompany the handbook (Cc=81.3), and mention the possibility of reversing the decision in the follow-up plan (Cc=87.6).

The closed questions included the following – 'Which elements of the handbook would you shorten?' (*¿Qué elementos reducirían del Manual?*) – to which the two most significant answers were the 'Introduction' (50%) and 'None' (31.3%). Following the comments made in the previous round, alternative formulations of the example phrases for the dialogues in each *the Three-talk* steps were given, as well as a change of name for step 2 to 'Option talk and exploring preferences' (*Plantear opciones y explorar preferencias*), on which consensus was reached (81,3%).

In their responses to the open questions, those who considered the proposed dialogues not to be representative of the steps had the opportunity to suggest how they could be reworded. Finally, participants were able to include their final comments on the handbook and the clinical practice guide. Most had no further suggestions for either document, but some participants

For peer review only

included comments about shortening the handbook (Box 7) and including this material in clinical practice guides, in order to improve implementation (Box 8).

Box 7: Response to 'Provide your final comments on the handbook'

> P10: *None, the idea of including appendices on communication skills for the health professional, and on the screening tests for the women, seems like an excellent idea to me, to avoid making the handbook longer but offer additional tools for those health workers and women who would like more information.*

Box 8: Response to 'Provide your final comments on the guide'

> P10: *Clinical practice guidelines on the preventive approach to breast cancer that includes these points on shared decision-making would be very useful to support implementation. In any case, I don't think that it is a prerequisite to be able to produce the handbook that you are working on. This handbook could be incorporated into future Clinical Practice Guidelines (CPG).*

Round 3

R3 was structured around the 10 elements about which no agreement was reached in R2. Six questions with closed, dichotomous answers were posed in the section evaluating the handbook, and 1 in the section evaluating the clinical practice guide, in addition to an open question. Of these, only those proposing an improvement to the organisation of the clinical practice guide, a change of colours and a review of cross-cutting communication skills in SDM reached a Cc of over 75% (Table 4).

Table 4: R3 responses

Given that agreement was not reached on the Flow diagram for the Early Detection of Breast Cancer Programme, this figure was removed from the handbook, in light of the fact that it only applies to the region of Catalonia. The other elements about which no agreement was reached were: the need to incorporate more examples of professional dialogues (64.7%); incorporating information about joint responsibility for the decision (41.2%); adding information on the limitations of the SDM model (58.8%), as well as adding supplementary resources on using the DST (52.9%) and on communication skills and competencies (58.8%). The researchers believed that the additional content would not entail substantial changes to the handbook and would provide more information to professionals who are not familiar with the model, so all these elements were incorporated into the handbook.

The texts included were developed in line with the proposals submitted by the participants in previous rounds. For example, the following elements were highlighted in the professional dialogues: the possibility of reversing the decision, needing more time, and accessing support from a third person to make the decision (Figure 2).

Figure 2: Example of dialogues for the professionals to "Team talk" step

The Delphi process was brought to close in R3, taking into account the criteria cited by Martínez regarding the elements about which agreement was not reached (27): a) the limited number of items for which Cc>75 was not achieved (6 of the 61 Likert-scale and closed questions); b) limited resources and time; c) the possibility that participants would abandon the

For peer review only

study in a subsequent round, which would affect the external validity of the study. The latter two criteria applied in the context of the COVID-19 pandemic, given that half of the participants are health professionals who work in health centres.

DISCUSSION

The literature mentions certain barriers to applying SDM in BC screening, including limited time in clinical appointments and health professionals' lack of training in providing more participatory care (19). This was the motivation for producing the first handbook and clinical practice guide on this subject, aimed at supporting health professionals by providing them with the essential elements for implementing SDM with women in a BC screening context.

The most relevant results included validation of the support materials using Delphi methodology, taking into account the opinion of experts to reach agreements on editing the design and content, as well as their recommendation to incorporate these materials into the clinical practice guide. This validation included the possibility of adapting a generic model – *Three Talk* – to one designed specifically for the BC screening context.

Of the 43 participants who were invited to respond to the Delphi questionnaires, more than half expressed interest in the topic of the research and collaborating in it. However, only 20 of these went on to participate in the study. This may be related to the timetabling of the questionnaires, which coincided with the end of the first wave of the COVID-19 pandemic and the resurgence of cases at the beginning of the second wave. In spite of this, the professionals who decided to participate at the beginning of the process fulfilled their commitment, illustrated by the fact that participant numbers only decreased by three between rounds, these having been lost from the Subjects category (n=3).

Discussion between the participants

It was easy to reach an agreement on the main content elements in the first round. Regarding the structure and development of SDM using the *Three-talk* model (22), which was considered suitable for BC screening, one of the participants initially suggested using the model created by the Agency for Healthcare Research and Quality (28). However, this alternative model contains five steps and the model proposed by the authors, which involves fewer steps, met all the requirements of SDM. Regarding the set of nine figures in the handbook, only one was eliminated, and the wording of three was edited.

The participants also easily came to an agreement that the initial version of the handbook was very long, at 56 pages. The length was due to the fact that it will be published in a pocket edition, which is equivalent to 23 pages in a larger textbook edition. The researchers decided to maintain the smaller format because it is more transportable, although they cut down the content elements agreed by the participants.

For six of the items, it was not possible to reach an agreement. While agreement should ideally be reached for all items, when a new round will not provide any more information or is unlikely to achieve a better result, the rounds of questions may be brought to a close despite a small number of disagreements remaining (27). The change in the formulation of the responses between R2 (Likert scale) and R3 (dichotomous) meant that participants had to opt for one of the options rather than rating their level of agreement with the statements, which undoubtedly made it more difficult to arrive at an agreement.

For peer review only

Certain responses to the open questions were analysed in depth by the researchers. One of the participants in R1 suggested that the professional self-assessment method could be changed from SDM-Q-doc (29) to Ask 3Q (30) or CollaboRATE (31). However, Ask 3Q is a methodology for applying SDM, making it an equivalent to the Three-talk model. Given that the Three-talk model received a positive evaluation from the participants, the change was not made. The other tool, CollaboRATE, is designed for the patient to evaluate the professional, which was not the purpose of this questionnaire (31). Our objective was for the professional to be able to evaluate how he or she performs SDM, resulting in self-guided learning of this methodology. The researchers therefore kept the original version, SDM-Q-doc, and adapted it for screening.

The decision on the flow diagram was affected by whether participants came from the region of Catalonia (of those living in Catalonia, 5/6 wanted to keep it, albeit improving its resolution; in contrast, the specialists originating outside Spain (7/11) opted to remove it). Given that the objective of the handbook is to be used in other territories, the research group decided to eliminate the flow diagram.

The example dialogues suggesting how professionals should conduct SDM at each point in the process were widely accepted as a fundamental part of the handbook, although no consensus was reached on whether to include more example dialogues for each steps (Su=4/6; Sp=7/11). While Cc>75 was not reached, a larger proportion of both groups advocated providing more examples. This may be directly related to the fact that both groups believed that SDM training for health professionals is still incomplete. Some of these participants therefore called for the handbook to provide more support, giving professionals greater confidence in implementation through use of the aforementioned dialogues. The same conclusion can be made regarding the decision to include more bibliographic references on communication skills and relationship-building competencies (Su=3/6; Sp=7/11) and on including information about PtDas (Su=5/6; Sp=4/11). In the latter case, the results differed between the two groups: most of the Subject participants wanted to add information on these tools, perhaps highlighting their lack of knowledge about them or training in their use, while the Specialists did not consider their inclusion to be as relevant, due to their familiarity with the tools.

How to improve the application of SDM to screening

While 83% of the health professionals expressed a high level of interest in promoting Shared Decision-Making during the clinical encounter (32), they recognised their lack of training in the SDM model as one of the most significant barriers to its implementation in the screening context (19).

A review of the training that health professionals receive confirmed our belief that there is a lack of strategies able to familiarise health professionals with this model. In Spain, the topic has been introduced into medicine and health-related degree programmes (33-36). However, it is not framed precisely within a SDM model, but is closer to communication or clinical communication skills, which have been used interchangeably as equivalents to the model. The level of detail and the strategies used in this training are also unknown. Most training in SDM is acquired in postgraduate-level study aimed at doctors and nurses (37), whereas particular attention should also be paid to health workers in primary care (including support and technical staff, as well as clinicians), who provide person-centred healthcare in a holistic manner (38).

Experts in SDM have argued that it is necessary to prioritise adapting curricula to consolidate this training, by emphasising education in communication skills and the accreditation of these competencies (39), within the framework of a horizontal care model. Additionally, experts highlight the need to create partnerships between universities and interdisciplinary research groups to develop this material (39).

Experts also recommend training methodology based on practical activities such as role plays, as well as working in small teams of six people, training of over a day in length, and providing constructive feedback on students' capacity to express empathy, give assertive responses, engage in active listening, and other skills (40). This handbook and clinical practice guide therefore include dialogues and specific examples of how to apply them. This will serve as reference material supporting an initial grounding in SDM for professionals who have not received formal training in this subject, and as supplementary material for those who have, enabling them to apply the skills and competencies acquired in the specific context of BC screening.

The final structure of our document responds to the need described in the preceding paragraph and highlighted by the participants in the Delphi study.

Given the change of paradigm that SDM entails, all measures that help familiarise professionals with SDM are important. For example, adding a section into Clinical Practice Guidelines (CPG) on how to include the patient in decision-making, thereby coordinating evidence-based practice with SDM (41), may be useful. Patients may even participate to some degree in its development, as is current practice in such organisations as the National Institute for Health and Clinical Excellence (NICE) and the Scottish Intercollegiate Guidelines Network (42). In this sense, our proposed handbook and clinical practice guide, as well as the PtDas whose quality has already been evaluated and certified by international organisations such as The Ottawa Hospital (43), may be considered complementary materials.

LIMITATIONS

The main limitation of the study was participant recruitment, which is a typical constraint. It was a particular problem in this case, given that the empirical work coincided with the successive waves of the COVID-19 pandemic, hindering the active participation of some professionals who had initially agreed to participate in the study. Despite this, there were fewer withdrawals from Round 2 onwards than might have been expected in the circumstances.

The change in the formulation of the R2 (Likert scale) and R3 (dichotomous) responses may have made it more difficult to reach the established minimum Cc for agreement. Nevertheless, with reference to Martínez (2003) (27), the research team determined that one more round would not have provided any added value to the results, for the reasons described in the preceding sections. Nevertheless, the decision made regarding those elements about which no agreement had been reached did not significantly affect the participants' opinions regarding the basic concepts on which the initial questionnaire was based.

Finally, it should be noted that a systematic literature review (2018) showed that more research is still needed to determine the real impact that training interventions have on health professionals regarding SDM, given that the level of certainty of the studies was low or very low. In this research, professionals who had received standard training were compared with those who had been trained in SDM; from the 15 studies, it was concluded that the results for

For peer review only

patients' satisfaction, knowledge, decision-related conflict, regret, level of health and quality of life differed little or not at all (37). In spite of this, the demand for information and training expressed by this study's participants leads us to believe that this first handbook aimed at health professionals for implementation in a BC screening context will help bring clarity to the healthcare model centred on patients and their needs and preferences. However, we have also noted the need to expand training in SDM and develop empirical strategies to facilitate its implementation.

CONCLUSION

A horizontal relationship between patients and health professionals enables person-centred care to be delivered, in which that person is considered a protagonist in the decisions made about his or her health. This has been recognised by several governmental organisations and incorporated into discourse and strategies. However, the practical application of this model is an area in which progress has yet to be made. The handbook and clinical practice guide therefore aim to familiarise professionals with the model, helping them to engage women in the decision of whether have BC screening or not. The results obtained enable us to conclude that, in order to be applied as public policy, a pilot study with health professionals is needed, which should be supplemented by formal training in SDM.

ACKNOWLEDGEMENTS

We would like to express our gratitude to the three expert reviewers in Shared Decision-Making and breast cancer: Victor Montori, Lilisbeth Perestelo-Pérez and Montserrat Rué; as well as the external reviewers, Lluís Colomés Figuera and Josep Maria Sabaté. We would also like to thank the 20 participants in the study who put their time, effort and perseverance into answering all the rounds of questions that the research team posed them.

FUNDING

Financial support for this study was provided entirely by a grant from Instituto de Salud Carlos III through the project PI18/00773 (co-funded by the European Regional Development Fund), and by the European Union's Horizon 2020 research and innovation programme, under Marie Skłodowska-Curie grant agreement No 713679 from Universitat Rovira i Virgili (URV). The funding agreement ensured the authors' independence in designing the study, interpreting the data, and writing and publishing the report.

DECLARATION OF CONFLICTS OF INTEREST

The authors declare that they have no conflict of interest.

REFERENCES

1. Council of Europe, Committee of Ministers: 'On screening as a tool of preventive medicine', Recommendation No R (94) 11, Strasbourg, Council of Europe, 1994.
2. World Health Organization. Control del cáncer de mama; 2020 [cited 30 June 2020]. Available from: https://www.who.int/topics/cancer/breastcancer/es/index3.html

For peer review only

3. Pérez-Lacasta MJ, Martínez-Alonso M, Garcia M, Sala M, Perestelo-Pérez L, Vidal C, Corden-Bové N, Feijoo-Cid M, Toledo-Chávarri A, Cardona Á, Pons A, Carles-Lacastra M, Rué M. Effect of information about the benefits and harms of mammography on women's decision making: The InforMa randomised controlled trial. Plos One. [Internet]. 2019;14(3). Available from: https://journals.plos.org/plosone/article?id=10.1371/ journal.pone.0214057
4. Grupo Español de Investigación en Cáncer de Mama (GEICAM). Guía GEICAM de Práctica Clínica: Para el Diagnóstico y Tratamiento del Cáncer de Mama Metastásico [GEICAM Clinical Practice Guide: For the Diagnosis and Treatment of Metastatic Breast Cancer]. Edition: Comuniland S.L. Madrid; 2015. Available from: https://portal.guiasalud.es/wp-content/uploads/2019/06/gpc_538_geicam_cancer_mama_resumida.pdf
5. Nehmat H. Overdiagnosis of breast cancer in population screening: does it make breast screening worthless? Cancer Biol Med [Internet]. 2017;14(1):1–8. Available from: http://www.cancerbiomed.org/index. php/cocr/article/view/1003
6. Febles G. Revisión de los beneficios del screening del cáncer de mama y análisis de las controversias [Review of the benefits of breast cancer screening and analysis of the controversies]. Rev. Imagenol. [Internet] 2015;19 (1)9- 18. Available from: https://www.webcir.org/revistavirtual/articu-los/2016/4_noviembre/ur/febles_esp.pdf
7. Day N. Overdiagnosis and breast cancer screening. Breast Cancer Res [Internet]. 2005;7(5):228–9. Available from: http://www.ncbi.nlm.nih.gov/pubmed/1616814445
8. Carter S, Barratt A. What is overdiagnosis and why should we take it seriously in cancer screening? Public Heal Res Pract [Internet]. 2017;27(3). Available from: http://www.phrp.com.au/?p=36726
9. Marmot M, Altman D, Cameron D, Dewar J, Thompson S, Wilcox M. The benefits and harms of breast cancer screening: An independent review. Br J Cancer [Internet]. 2013;108(11): 2205–2240. Available from: https:// www.ncbi.nlm.nih.gov/pmc/articles/PMC3693450/
10. Munthe C, Sandman L, Cutas D. Person Centred Care and Shared Decision Making: Implications for Ethics, Public Health and Research. Health Care Anal [Internet]. 2012;20(3):231–49. Available from: http://link. springer.com/10.1007/s10728-011-0183-y
11. Charles C, Gafni A, Whelan T. Decision-making in the physician-patient encounter: revisiting the shared treatment decision-making model. Soc Sci Med [Internet]. 1999;49(5):651–61. Available from: http://www.ncbi.nlm.nih.gov/pubmed/10452420
12. Carmona-Torres JM, Cobo-Cuenca A, Martín-Espinoza N Piriz-Campos R, Laredo-Aguilera J, Rodríguez -Borrego M. Prevalencia en la realización de mamografías en España: análisis por comunidades 2006-2014 y factores que influyen [The frequency with which mammograms are performed in Spain: Analysis by autonomous community from 2006 to 2014, and influencing factors]. Aten Primaria. 2018;50(4):228-237. Available from: https://www.elsevier.es/es-revista-atencion-primaria-27-articulo-prevalencia-realizacion-mamografias-espana-analisis-S021265671630539X
13. Generalitat de Catalunya. Departament de Salut. Pla director 2017. [Master plan 2017]. Available from: http://salutweb.gencat.cat/ca/ambits_actua-cio/linies_dactuacio/estrategies_salut/cancer/pla_director/
14. Generalitat de Catalunya. Departament de Salut. Pla director D'Oncologia 2016-2020: Un sistema centrat en la persona: públic, universal i just [Master Oncology Plan 2016-2020: A person-centred system: Public, universal and fair]. 2016. [Internet]. Available

from: http://salutweb.gencat.cat/web/.con-tent/_departament/pla-de-salut/Pla-de-salut-2016-2020/documents/ Pla_salut_Catalunya_2016_2020.pdf
15. Institut Català d`Oncologia. Detecció Precoç de Càncer de Mama [Early detection of breast cancer]. [Internet]. 2020 [cited 30 June 2020]. Available from: http://ico. gencat.cat/ca/el-cancer/programes_de_deteccio_precoc/programa_de_deteccio_precoc_de_cancer_de_mama/index.html
16. Toledo-Chavarri A, Rué M, Codern-Bove N, Carles-Lavila M, Perestelo-Perez L, Perez-Lacasta MJ, and Feijoo-Cid M. A Qualitative Study on a Decision Aid for Breast Cancer Screening: Views from Women and Health Professionals. Eur J Cancer Care (Engl) [Internet]. 2017:26(3). Available from: https://onlinelibrary.wiley.com/doi/abs/10.1111/ecc.12660
17. Légaré F, Witteman H. Shared decision making: examining key elements and barriers to adoption into routine clinical practice. Health Aff (Mill- wood). [Internet]. 2013;32(2):276-84 Available from: https://www. healthaffairs.org/doi/pdf/10.1377/hlthaff.2012.1078
18. Charles C, Gafni A, Whelan T. Self-reported use of shared decision-making among breast cancer specialists and perceived barriers and facilitators to implementing this approach. Health Expect [Internet]. 2004;7(4):338–48. Available from: http://www.ncbi.nlm.nih.gov/pubmed/15544686
19. Hernández-Leal MJ, Pérez-Lacasta M, Feijoo-Cid M, Ramos-García V, Carles-Lavila M. Healthcare professionals behaviour to implement in shared decision making in screening programs: A systematic review. Patient Educ Couns. 2021. doi: https://doi.org/10.1016/j.pec.2021.01.032
20. Hernández-Leal MJ, Carles-Lavila M, Pérez-Lacasta M. La participación de los profesionales de la salud en la Toma de Decisiones Compartida en el cribado de cáncer de mama: Manual de apoyo para implementación de la TDC [The participation of health professionals in Shared Decision-Making in breast cancer screening: A handbook to support the implementation of SDM]. Spain: María José Hernández editor; 2020.
21. Croes KD, Jones NR, DuBenske LL, Schrager SB, Mahoney JE, Little TA, Burnside ES. Core Elements of Shared Decision-making for Women Considering Breast Cancer Screening: Results of a Modified Delphi Survey. J Gen Intern Med. 2020;35(6):1668-1677. doi: 10.1007/s11606-019-05298-8. Epub 2020 Mar 19. PMID: 32193817; PMCID: PMC7280383.
22. Elwyn G, Duran M, Song J, Aarts J, J Barr P, Berger Z, etc al. A three-talk model for shared decision making: multistage consultation process. BMJ. 2017;359. [Internet]. Available from: https://www.bmj.com/con-tent/359/bmj.j4891.long
23. Reguant-Álvarez M. y Torrado-Fonseca M. El método Delphi [The Delphi method]. Revista d'Innovació i Recerca en Educació. Universidad de Barcelona. Institut de Ciéncies de P´Educació. 2016:9(1). Available from: https://revistes.ub.edu/index.php/REIRE/article/view/reire2016.9.1916
24. López-Gómez, E. El método Delphi en la investigación actual en educación: una revisión teórica y metodológica [The Delphi method in current education research: A theoretical and methodological review]. Educación XX1. 2018;21(1):17-40
25. Benner P, Lee P, Stannard D. (1999). Clinical wisdom in critical care: a thinking- in-action approach. Saunders.
26. García-Ruiz M, Lena-Acebo F. Aplicación del metodo delphi en el diseño de una investigación cuantitativa sobre el fenómeno FABLAB [Application of the Delphi method to the design of quantitative research on the FABLAB

phenomenon]. Empiria. Revista de metodología de ciencias sociales, 2018:40;129-166. doi:https://doi.org/10.5944/empiria.40.2018.22014.
27. Martínez-Piñeiro M. La técnica Delphi como estrategia de consulta a los implicados en la evaluación de programas [The Delphi technique as a strategy for consulting participants in the evaluation of programmes]. RIE. 2003; 21(2): 449-463. Available from: https://revistas.um.es/rie/article/view/99311/94911
28. The SHARE Approach—Essential Steps of Shared Decision Making: Quick Reference Guide. 2014. [Internet]. Available from: https://www.ahrq.gov/health-literacy/curriculum-tools/shareddecisionmaking/tools/tool-1/index.html
29. De las Cuevas C, Perestelo-Perez L, Rivero-Santana A, Cebolla-Martí A, Scholl I, Härter M. Validation of the Spanish version of the 9-item Shared Decision-Making Questionnaire. Health Expect. 2015; 18(6):2143-53. doi: 10.1111/hex.12183. Epub 2014 Mar 5. PMID: 24593044; PMCID: PMC5810694.
30. Shepherd HL, Barratt A, Trevena LJ, McGeechan K, Carey K, Epstein RM, Butow PN, Del Mar CB, Entwistle V, Tattersall MH. Three questions that patients can ask to improve the quality of information physicians give about treatment options: a cross-over trial. Patient Educ Couns. 2011;84(3):379-85. doi: 10.1016/j.pec.2011.07.022. Epub 2011 Aug 9. PMID: 21831558.
31. Elwyn G, Barr PJ, Grande SW, Thompson R, Walsh T, Ozanne EM. Developing CollaboRATE: a fast and frugal patient-reported measure of shared decision making in clinical encounters. Patient Educ Couns. 2013;93(1):102-7. doi: 10.1016/j.pec.2013.05.009. Epub 2013 Jun 12. PMID: 23768763.
32. Martínez KA, Deshpande A, Ruff AL, Bolen SD, Teng K, Rothberg MB. Are Providers Prepared to Engage Younger Women in Shared Decision-Making for Mammography? J Womens Health (Larchmt). 2018;27(1):24-31. DOI: 10.1089/jwh.2016.6047. Epub 2017 Jun 28. PMID: 28657820.
33. Universidad Complutense de Madrid. Guía Docente 2015-2016 [Teaching guide 2015-2016]. Facultad de Medicina. Available from: https://medicina.ucm.es/data/cont/media/www/pag-17227/Gu%C3%ADa%20Medicina%202015-2016.pdf
34. Universitat Autónoma de Barcelona. Programa Bioética y Comunicación 2020/2021 [Bioethics and Communication Programme 2020/2021]. Available from: https://guies.uab.cat/guies_docents/public/portal/html/2020/assignatura/102950/es
35. Complejo Hospitalario Universitario de Badajoz (CHUB). Guía Docente Medicina Intensiva CHUB [CHUB Intensive Care Medicine Teaching Guide]. 2020. Available from: https://www.areasaludbadajoz.com/docencia_investigacion/comision_docencia/Guias_e_Itinerarios_Formativos/Guia_formativa_UCI.pdf
36. Ministerio de Ciencia e Innovación de España. Orden CIN/2134/2008, de 3 de julio, por la que establecen los requisitos para la verificación de los títulos universitarios oficiales que habilitan para el ejercicio de la profesión de Enfermero [Order CIN/2134/2008 of 3 July, establishing the requirements for verification of the official university certificates that act as qualifications for practising the profession of Nurse]. Agencia Estatal Boletín Oficial del Estado. 2008 (174). Available from: https://www.boe.es/eli/es/o/2008/07/03/cin2134
37. Légaré F, Adekpedjou R, Stacey D, Turcotte S, Kryworuchko J, Graham ID, et. al. Interventions for increasing the use of shared decision making by healthcare professionals. Cochrane Database Syst Rev. 2018;7(7):CD006732. DOI: 10.1002/14651858.CD006732.pub4. PMID: 30025154; PMCID: PMC6513543.

38. Dois A, Bravo P, Contreras A, Soto MG, Mora I. Formación y competencias para los equipos de atención primaria desde la mirada de expertos chilenos [Training and competencies for primary care teams from the perspective of Chilean experts; Treinamento e competências para equipes de atenção primária na perspectiva de especialistas chilenos]. Rev Panam Salud Publica. 2018 Sep 24;42:e147. Spanish. doi: 10.26633/RPSP.2018.147. PMID: 31093175; PMCID: PMC6386070.
39. García-Altés A, Peiró M, Artells JJ. Priorización de medidas para la consolidación de la toma de decisiones compartidas en las prestaciones del Sistema Nacional de Salud español [Prioritising measures for consolidating shared decision-making in the services rendered by the Spanish National Health System]. Gac Sanit. 2019;33(5):408-414. Spanish. doi: 10.1016/j.gaceta.2018.04.017. Epub 2018 Jul 20. PMID: 30033094.
40. Berkhof M, van Rijssen HJ, Schellart AJ, Anema JR, van der Beek AJ. Effective training strategies for teaching communication skills to physicians: an overview of systematic reviews. Patient Educ Couns. 2011;84(2):152-62. doi: 10.1016/j.pec.2010.06.010. Epub 2010 Jul 29. PMID: 20673620.
41. Perestelo-Perez L, Rivero-Santana A, Perez-Ramos J, Gonzalez-Lorenzo M, Roman JG, Serrano-Aguilar P. Shared decision making in Spain: current state and future perspectives. Z Evid Fortbild Qual Gesundhwes. 2011;105(4):289-95. doi: 10.1016/j.zefq.2011.04.013. Epub 2011 Apr 30. PMID: 21620323.
42. Grupo de trabajo de implicación de pacientes en el desarrollo de GPC. Implicación de Pacientes en el Desarrollo de Guías de Práctica Clínica: Manual Metodológico [Involvement of Patients in the Development of Clinical Practice Guides: Methodological Handbook]. Ministerio de Sanidad, Servicios Sociales e Igualdad. Instituto Aragonés de Ciencias de la Salud-IACS. Guías de Práctica Clínica en el SNS: IACS No 2010/01.
43. The Ottawa Hospital. Patient Decision Aids. Available from: https://decisionaid.ohri.ca/AZsearch.php?criteria=screening

TABLES

Table 1: Characteristics of the participants

Variable		Round 1		Round 2		Round 3	
		N	%	N	%	N	%
Sex	Female	15	75	12	75	13	76.47
	Male	5	25	4	25	4	23.52
	Total	20	100	16	100	17	100
Age range (years)	31-40	7	35	7	43.75	7	41.17
	41-50	6	30	4	25	5	29.41
	51-60	5	25	4	25	4	23.52
	61-70	2	10	1	6.25	1	5.88
	Total	20	100	16	100	17	100
Ownership of the affiliated institute, health centre or research site	Public sector	14	70	11	68.75	11	64.7
	Private sector	6	30	5	31.25	6	35.29
	Total	20	100	16	100	17	100
Profession	Nursing	4	20	2	12.5	3	17.64
	Medicine	13	65	11	68.75	11	64.7
	Psychology	1	5	1	6.25	1	5.88
	Other	2	10	2	12.5	2	11.76
	Total	20	100	16	100	17	100
Specialty	Family and community medicine or nursing	14	70	11	68.75	12	70.58
	Public health	1	5	1	6.25	1	5.88
	Gynaecology	1	5	1	6.25	1	5.88
	Endocrinology	1	5	1	6.25	1	5.88
	Research in health services	1	5	1	6.25	1	5.88
	Content development for Decision Support Systems for Healthcare	1	5	1	6.25	1	5.88
	None	1	5	0	0	0	0
	Total	20	100	16	100	17	100
Experience (years)	6-10	6	30	6	37.5	6	35.29
	11-20	6	30	5	31.25	6	35.29
	21-30	6	30	5	31.25	5	29.41
	31-40	2	10	0	0	0	0
	Total	20	100	16	100	17	100

For peer review only

Development of support material for health professionals who are implementing Shared Decision-Making in breast cancer screening: Validation using Delphi methodology

Support material for SDM in breast cancer screening

Table 2: R1 responses

Section	Questions using a Likert scale of 1 (Completely disagree) to 6 (Completely agree)	1	2	3	4	5	6	Cc*
Evaluation of the Handbook on Shared Decision-Making in Breast Cancer Screening	1. The sections of the handbook are effective for understanding the application of SDM to breast cancer screening	0	5	5	10	55	25	90
	4. The 'Contents' section is suitable for this handbook	0	5	5	5	35	50	90
	5. The 'Objective of the material' section is suitable for this handbook	0	5	0	5	30	60	95
	6. The 'Who is it aimed at?' section is suitable for this handbook	0	5	0	10	35	50	95
	7. The 'Introduction' section is suitable for this handbook	5	10	0	10	45	30	85
	8. The 'Shared Decision-Making: What is it?' section is suitable for this handbook	0	5	5	10	40	40	90
	9. The 'Shared Decision-Making: Why is it important?' section is suitable for this handbook	0	5	0	5	45	45	95
	10. The 'Shared Decision-Making: 'What skills or competencies do health professionals need?' section is suitable for this handbook	0	5	5	35	35	20	90
	11. The 'Shared Decision-Making: What do patients think?' section is suitable for this handbook	0	10	0	10	35	45	90
	12. The 'Shared Decision-Making in breast cancer screening: The screening programme' section is suitable for this handbook	0	10	10	5	30	45	80
	13. The 'Shared Decision-Making in breast cancer screening: Implementation of SDM in breast cancer screening' section is suitable for this handbook	0	5	0	5	45	45	95
	14. The 'Shared Decision-Making in breast cancer screening: Self-assessment of the SDM process' section is suitable for this handbook	0	10	10	10	35	35	80
	15. The handbook provides the minimum content on SDM in breast cancer screening that health professionals should be familiar with	0	5	0	20	50	25	95
	16. The content of the handbook is sufficiently detailed	0	5	5	5	35	50	90
	19.a. Figure 1: Models of healthcare (page 14) is useful	0	0	10	15	30	45	90
	19.b. Figure 1: Models of healthcare (page 14) is clear	0	0	15	10	20	55	85
	20.a. Figure 2: Role of the participants in the clinical encounter (page 15) is useful	0	5	5	10	35	45	90
	20.b. Figure 2: Role of the participants in the clinical encounter (page 15) is clear	0	5	5	15	30	45	90
	21.a. Figure 3: Elements of Shared Decision-Making (page 16) is useful	0	0	10	20	25	45	90
	21.b. Figure 3: Elements of Shared Decision-Making (page 16) is clear	0	0	5	20	25	50	95
	22.a. Figure 4: Communication skills (page 21) is useful	5	0	10	30	15	40	85
	22.b. Figure 4: Communication skills (page 21) is clear	0	0	10	20	25	45	90
	23.a. Figure 5: Flow diagram of the Early Detection of Breast Cancer programme (page 27) is useful	5	5	20	15	20	35	70
	23.b. Figure 5: Flow diagram of the Early Detection of Breast Cancer programme (page 27) is clear	10	10	20	15	15	30	60
	24.a. Figure 6: Team talk (page 34) is useful	10	5	0	30	25	30	85
	24.b. Figure 6: Team talk (page 34) is clear	10	0	15	20	25	30	75
	26.a. Figure 7: Option talk (page 36) is useful	5	5	0	30	40	20	90
	26.b. Figure 7: Option talk (page 36) is clear	5	0	10	45	15	25	85
	28.a. Figure 8: Decision talk (page 38) is useful	0	5	10	5	35	45	80

For peer review only

	28.b. Figure 8: Decision talk (page 38) is clear	0	0	5	15	30	50	95
	30.a. Figure 9: Shared Decision-Making steps (page 39) is useful	0	0	5	20	25	50	95
	30.b. Figure 9: Shared Decision-Making steps (page 39) is clear	0	0	10	35	10	45	90
	31. Does its design (colours, images) make the handbook easier to read for an SDM professional?	0	0	5	20	35	40	95

Closed questions	Options	Percentage (%)
2. Which section of the handbook do you think should be changed?	a) Front cover	0
	b) Objective of the material	0
	c) Who is it aimed at?	0
	d) Introduction	10
	e) Shared Decision-Making: What is it?	0
	f) Shared Decision-Making: Why is it important?	0
	g) Shared Decision-Making: What skills or competencies do health professionals need?	25
	h) Shared Decision-Making: What do patients think?	0
	i) Shared Decision-Making in breast cancer screening: The screening programme	15
	j) Shared Decision-Making in breast cancer screening: Implementation of SDM in breast cancer screening	0
	k) Shared Decision-Making in breast cancer screening: Self-assessment of the SDM process	0
	l) None	50
	Total	100

Evaluation of the Clinical Practice Guide: Implementation of SDM for Health Professionals	Questions using a Likert scale of 1 (Completely disagree) to 6 (Completely agree)	1	2	3	4	5	6	Cc*
	1. Do you think that a clinical practice guide concisely summarising the SDM steps is necessary?	0	15	10	5	5	65	75
	6. Is it useful to incorporate the Self-assessment section in the clinical practice guide?	0	5	10	25	25	35	85

$*Cc = \left(1 - \frac{Vn}{Vt}\right) x100$

For consensus, a Coefficient of Concordance (Cc) >75 was used. Vn = Number of negative votes (score of less than 4); Vt = Total number of votes (n=6)[26].

For peer review only

Table 3: R2 responses

Section	Questions using a Likert scale of 1 (Completely disagree) to 6 (Completely agree)	1	2	3	4	5	6	CC*
Evaluation of the Handbook on Shared Decision-Making in Breast Cancer Screening	1. Eliminate Figure 5: Flow diagram of the Early Detection of Breast Cancer Programme (page 27)	6.3	18.8	18.8	6.3	43.8	6.3	56.4
	2. Shorten content: the handbook format is very long	0	12.5	6.3	18.8	25	37.5	81.3
	4. Incorporate more examples of dialogues between the professional and the woman into each phase	18.8	6.3	6.3	31.3	31.3	6.3	68.9
	5. Add information on communication skills and competencies resources	0	12.5	25	12.5	43.8	6.3	62.6
	6. Add information on joint responsibility for the shared decision-making agreement	6.3	31.3	12.5	0	37.5	12.5	50
	7. Add information about resources on using the Patients Decision Aids (PtDAs). Note that this tool is intended to be used with the women	0	18.8	12.5	18.8	18.8	31.3	68.9
	8. Add information on the limitations of the SDM model	6.3	18.8	25	12.5	25	12.5	50
	9. Provide example dialogues on exploring the women's values, beliefs and preferences	0	18.8	12.5	18.8	31.3	18.8	68.9

Evaluation of the Handbook on Shared Decision-Making in Breast Cancer Screening (continued):

Closed questions	Options	Percentage (%)
3. Which element of the handbook would you shorten?	a) Objective of the material	0
	b) Who is it aimed at?	0
	c) Introduction	50
	d) Shared Decision-Making: What is it?	0
	e) Shared Decision-Making: Why is it important?	0
	f) Shared Decision-Making: What skills or competencies do health professionals need?	0
	g) Shared Decision-Making: What do patients think?	6.3
	h) Shared Decision-Making in breast cancer screening: The screening programme	6.3
	i) Shared Decision-Making in breast cancer screening: Implementation of SDM in breast cancer screening	6.3
	j) Shared Decision-Making in breast cancer screening: Self-assessment of the SDM process	0
	k) None	31.3
	Total	100
10. Change the name of phase 2	a) Option talk (current name)	18.8
	b) Option talk and exploring preferences (proposal)	81.3
	c) Other	0
	Total	100
12. Phase 1 dialogue: Team Talk (page 34):	a) Now that we know that you can decide what to do about screening, we're going to talk about the characteristics of screening, so that you know what your options are (current dialogue).	12.5
	a) You have the option of deciding whether or not to have early-detection tests for breast cancer. If you're happy to, we can explore together what risks and benefits the test involves for you (proposal).	81.3
	c) Other	6.2
	Total	100

	14. Phase 2 dialogue: Option Talk (page 36)	a) I appreciate you sharing your views with me and I'm here to help you come to a good decision. Let's do a recap of your preferences and check whether you have any more questions (current dialogue).	18.8
		B) I'm here to help you make a decision. Let's look at what your preferences are and the various options available, and we'll check whether you have any questions about them (proposal).	75
		c) Other	6.2
		Total	100
	16. Phase 3 dialogue: Decision Talk (page 38):	a) Do you think that you're ready to make the decision or do you need more time? (current dialogue).	12.5
		b) Now that we've gone over the advantages and disadvantages of early detection, do you think that you can make the decision? Bear in mind that this can be delayed if you need more time or to talk about it with someone of your choice (proposal).	81.3
		c) Other	6.2
		Total	100

	Questions using a Likert scale of 1 (Completely disagree) to 6 (Completely agree)	1	2	3	4	5	6	Cc*
Evaluation of the Clinical Practice Guide: Implementation of SDM for Health Professionals	1. A clinical practice guide is necessary for this handbook	6.3	0	12.5	25	31.3	25	81.3
	2. Improve the design of the clinical practice guide to improve understanding (colour, structure, etc.)	6.3	0	18.8	37.5	25	12.5	75
	3. Eliminate additional information (definitions of Risk factors, Mammography, Shared Decision-Making)	6.3	18.8	18.8	6.3	18.8	31.3	56.4
	4. Mention the possibility of reversing the decision in the follow-up plan	6.3	0	6.3	18.8	25	43.8	87.6
	5. Mention relationship-building competencies: active listening, showing empathy, clarification, etc.	12.5	6.3	12.5	6.3	43.8	18.8	68.9

$*Cc = \left(1 - \frac{Vn}{Vt}\right)x100$

For consensus, a Coefficient of Concordance (Cc) >75 was used. Vn = Number of negative votes (score of less than 4); Vt = Total number of votes (n=6)[26].

For peer review only

Table 4: R3 responses

Section	Closed questions	Options	Percentage (%)
Evaluation of the Handbook on Shared Decision-Making in Breast Cancer Screening	1. Given that no consensus has been reached (56.4 %) on whether or not to eliminate Figure 5: Flow diagram of the Early Detection of Breast Cancer programme (page 27), please select one of the following options:	a) Eliminate. It does not add relevant information to this handbook	47.1
		b) Keep. Translate to Spanish and improve the image resolution	52.9
		Total	
	2. Given that there is no consensus (68.9 %) about whether to add more examples of dialogues between the professional and the women for each phase, please select one of the following options:	a) One example per phase (current format)	35.3
		b) Three examples per phase (proposed new format) The image will be adapted to a more readable size for the handbook	64.7
		Total	100
	3. Given that there is no consensus (62.6 %) about whether to add information on communication skills and competencies resources to the handbook, please select one of the following options:	a) Yes, it is necessary to incorporate bibliographic references into the handbook for those who would like to find out more about this topic.	58.8
		b) No, the handbook is already too long to add more information.	64.7
		Total	100
	4. Given that there is no consensus (50 %) about whether to include information on joint responsibility for the SDM agreement, please select one of the following options:	a) Yes, it should be included because the information is not clear	41.2
		b) It is not necessary, it is already clear that the responsibility is shared	58.8
		Total	100
	5. Given that there is no consensus (68.9 %) about whether bibliographic references should be added on the Decision Support Tool (DST) – note that the DST is an appendix to the handbook, to be used by the woman and health professional – please select one of the following options:	a) Yes, they should be added	52.9
		b) No, this is not necessary	47.1
		Total	100
	6. Given that there is no consensus (50 %) about whether to add information on the limitations of the model, please select one of the following options:	a) Yes, this is necessary because not doing so would mean producing one-sided material	58.8
		b) No, it is not necessary because the objective of the handbook is to show the advantages of implementing it	41.2
		Total	100
Evaluation of the Clinical Practice Guide: Implementation of SDM for Health Professionals	1. Given that there is no consensus about the design and content of the guide, please select one of the following options. The infographic will be adapted to a more readable size for the guide.	a) Current format	23.5
		b) Proposed new format	76.5
		Total	100

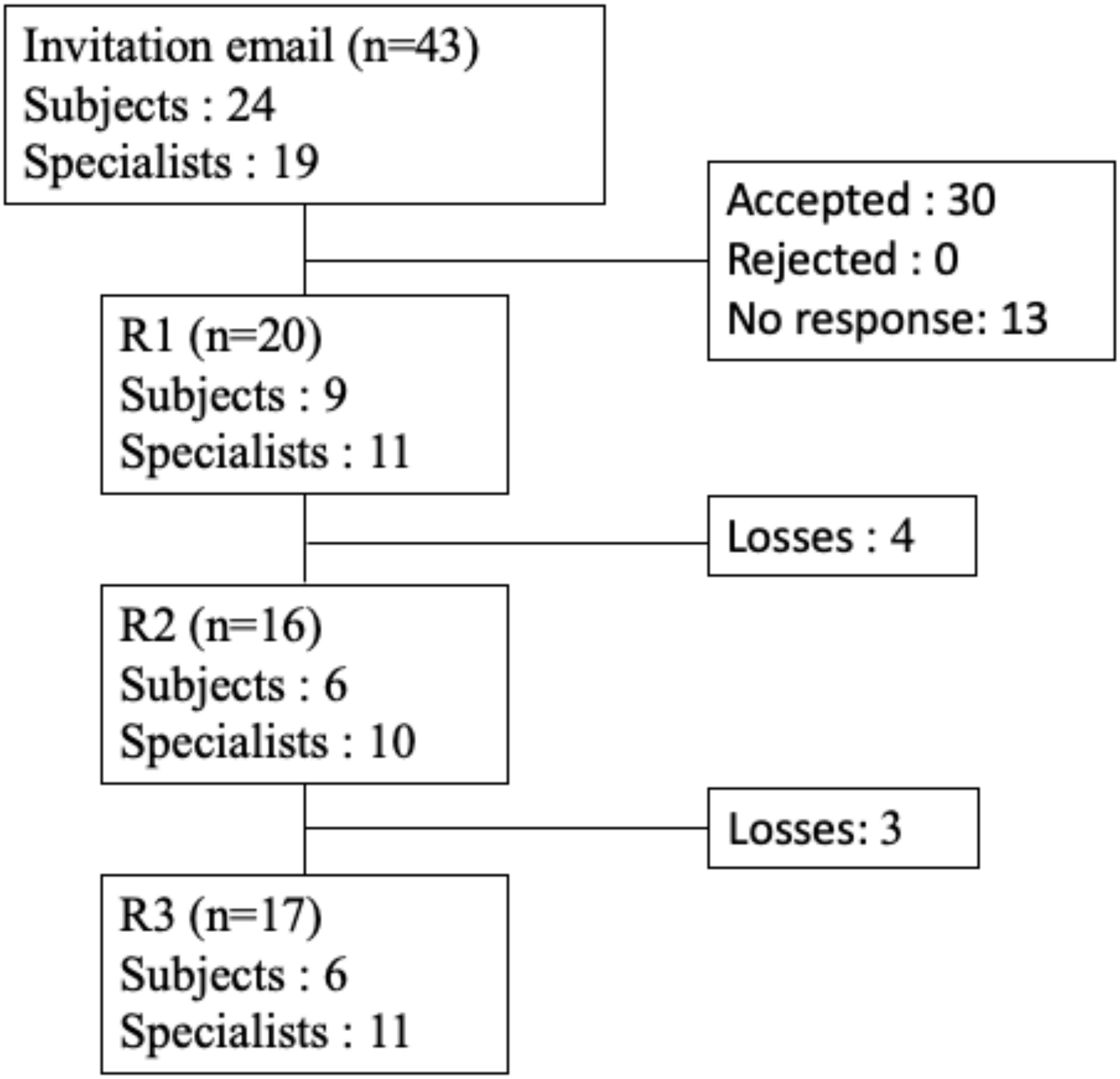
Invitation email (n=43)
Subjects : 24
Specialists : 19
Accepted : 30
Rejected : 0
No response: 13
R1 (n=20)
Subjects : 9
Specialists : 11
Losses : 4
R2 (n=16)
Subjects : 6
Specialists : 10
Losses: 3
R3 (n=17)
Subjects : 6
Specialists : 11

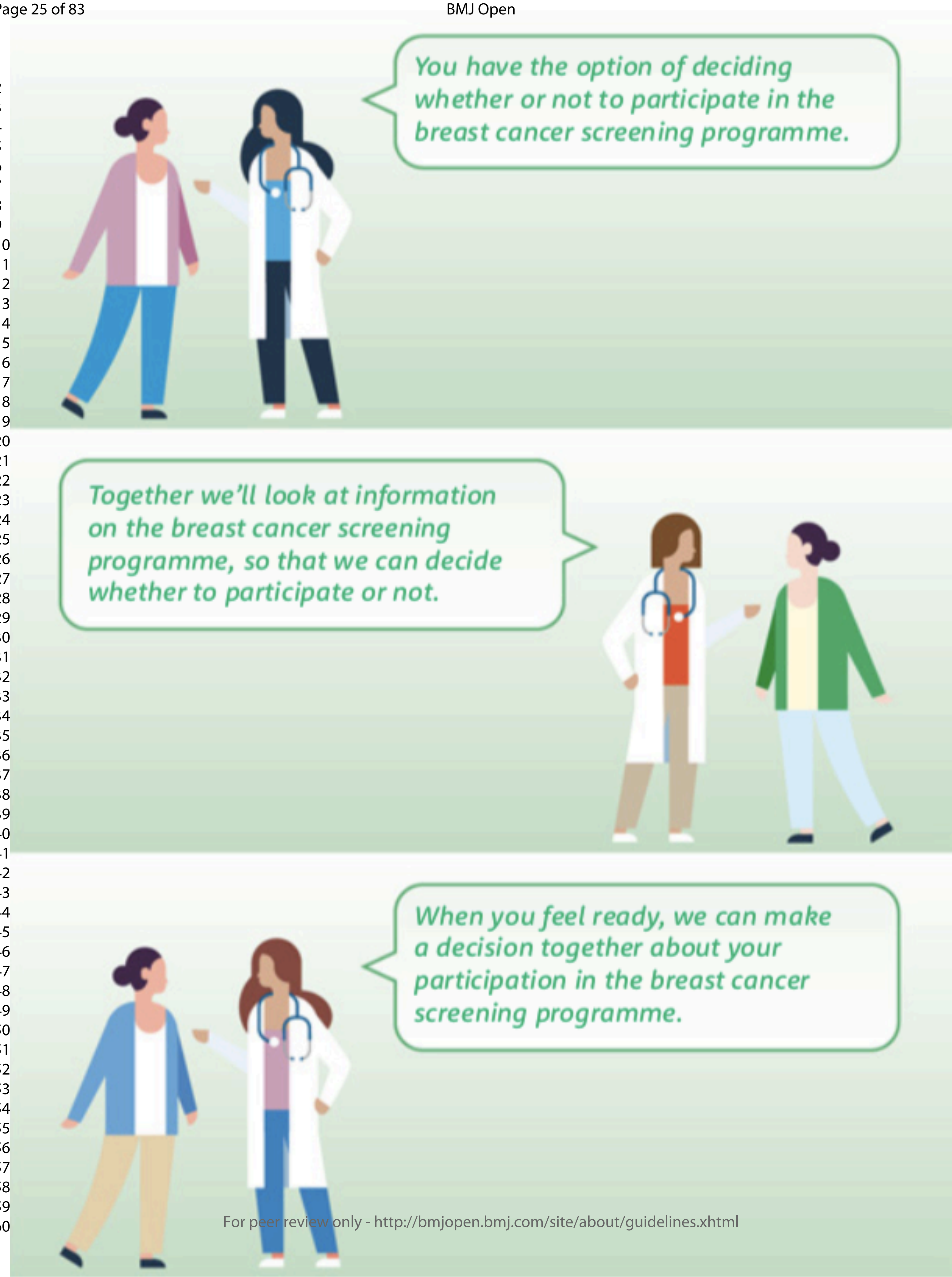
You have the option of deciding whether or not to participate in the breast cancer screening programme.
Together we'll look at information on the breast cancer screening programme, so that we can decide whether to participate or not.
When you feel ready, we can make a decision together about your participation in the breast cancer screening programme.

The Participation of Health Professionals in Shared Decision-Making on Breast Cancer Screening

A Handbook to Support the Implementation of Shared Decision-Making

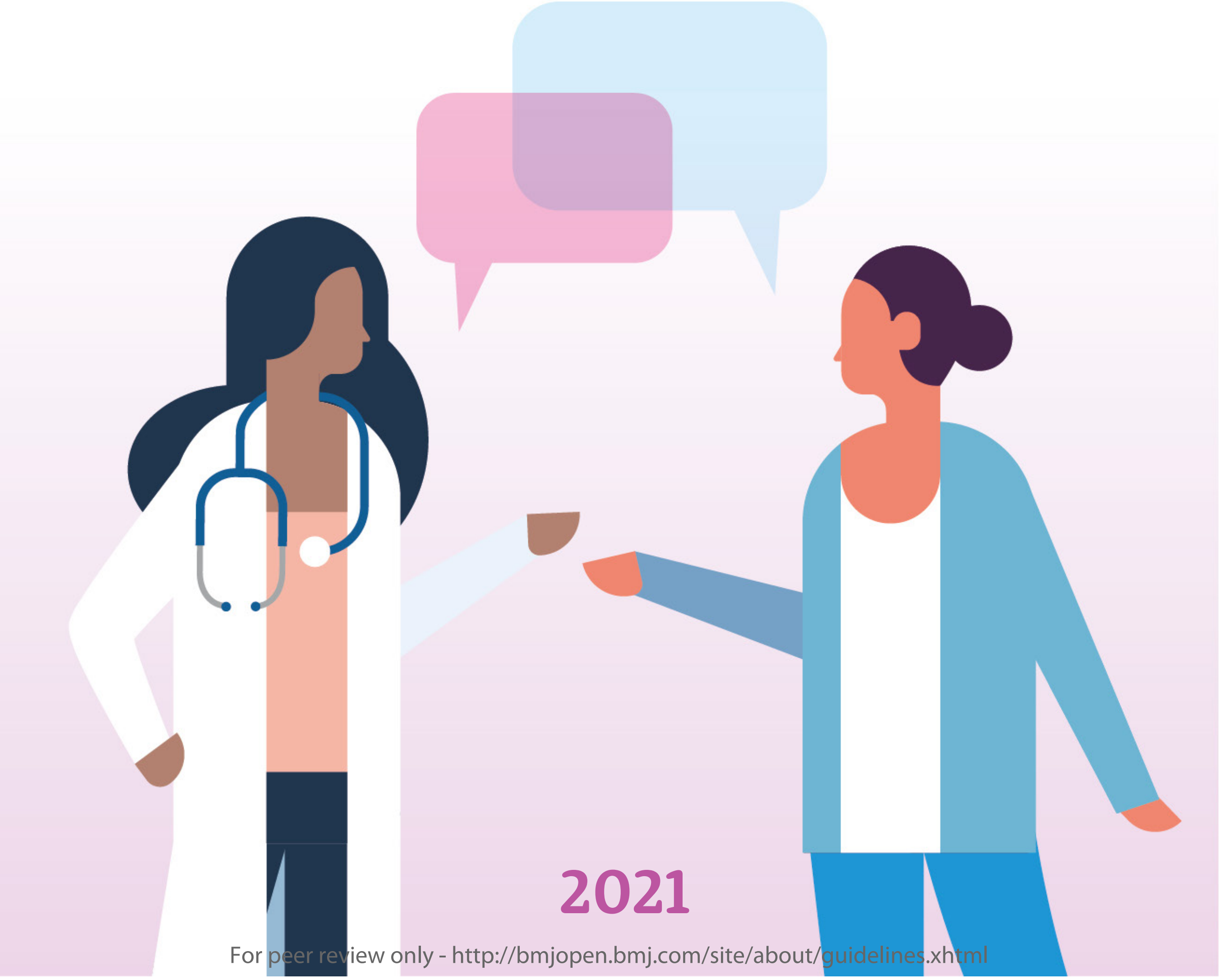

2021

For peer review only - http://bmjopen.bmj.com/site/about/guidelines.xhtml

For peer review only

A Handbook to Support the Implementation of SDM

2

AUTHORS

María José Hernández-Leal[1,a,b,c], María José Pérez-Lacasta[2,a,c] and Misericòrdia Carles-Lavila[3,a,b,c] on behalf of the ProShare Group.

1. PhD student, Msc and Bsc Nursing. Mail: mariajose.hernandez@urv.cat
2. PhD, Mail: mariajose.perez@urv.cat
3. PhD. Mail: misericordia.carles@urv.cat

a. Department of Economics, Universitat Rovira i Virgili.
b. Research Centre on Economics and Sustainability (ECO-SOS).
c. Research Group on Statistical and Economic Analysis in Health (GRAEES).

ProShare Research Group:

Misericòrdia Carles Lavila
Núria Codern-Bové
María José Hernández Leal
María José Pérez Lacasta
Roger Pla
Vanesa Ramos García
Carmen Vidal

Cómo citar este documento:

Hernández-Leal MJ, Carles-Lavila M, Pérez-Lacasta M. The Participation of Health Professionals in Shared Decision-Making on Breast Cancer Screening: A Handbook to Support the Implementation of SDM. Spain: María José Hernández, editor; 2021.

EXTERNAL REVIEWERS

Dra. Lilisbeth Perestelo-Pérez
Evaluation and Planning Service.
Dirección del Servicio Canario de Salud, Spain.
Research Network on Health Services in Chronic Diseases (REDISSEC).

Dr. Victor Montori
Knowledge and Evaluation Research (KER) Unit.
Mayo Clinic - Minnesota, USA.

Dra. Montserrat Rué
Institut de Recerca Biomèdica de Lleida (IRBLLEIDA).
Department of Basic Medical Sciences.
Universitat de Lleida, España.

DESIGN

Miquel A. Fernández
www.mafsdisseny.com

FUNDING

- The European Regional Development Fund (ERDF). European Union's Horizon 2020 research and innovation programme under the Marie Skłodowska-Curie grant agreement No. 713679 from the Universitat Rovira i Virgili (URV).
- Project PI18/00773 "The collaboration of health professionals in incorporating shared decision-making into the breast cancer screening programme", funded by Instituto de Salud Carlos III and cofunded by the European Union (FEDER) "A way to make Europe".

For peer review only

For peer review only

CONTENTS

THE OBJECTIVE OF THIS HANDBOOK 6

WHO IS IT FOR? 6

Introduction 7

Shared Decision-Making 11

1. What is it? 12

2. Why is it important? 15

3. The limitations of the model 17

4. What skills or competencies do health professionals need? 18

5. What do patients think? 22

Shared Decision-Making in Breast Cancer Screening 25

1. The screening programme 26

2. Implementing SDM in breast cancer screening 30

3. Self-assessment of the SDM process 39

Bibliographic References 43

For peer review only

THE OBJECTIVE OF THIS HANDBOOK

The aim of this handbook is to act as a guide on how to develop and implement **Shared Decision-Making (SDM)** in breast cancer screening.

WHO IS IT FOR?

Health professionals who are involved in breast cancer screening and have direct contact with women that are advised to participate in the programme.

For peer review only

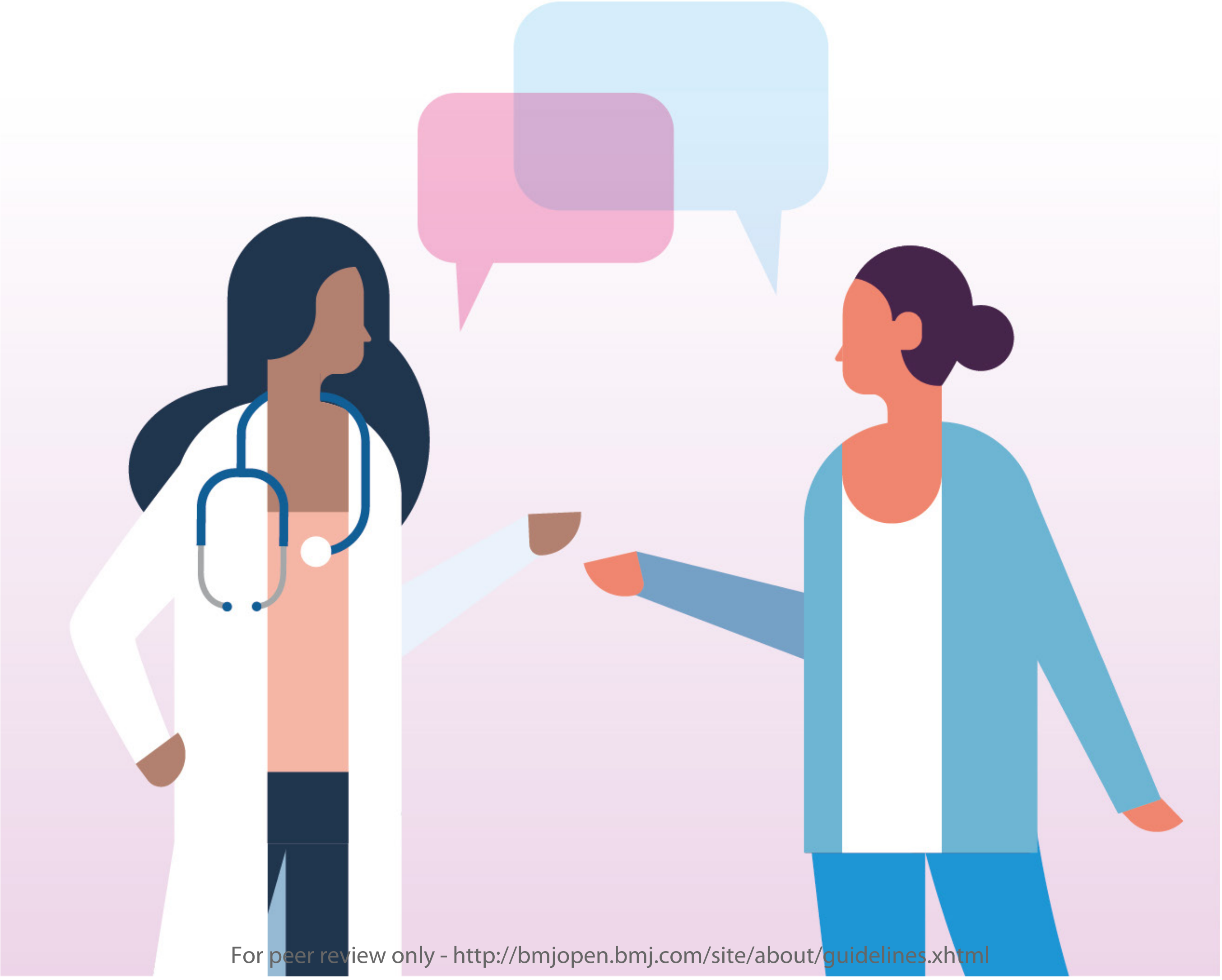

For peer review only - http://bmjopen.bmj.com/site/about/guidelines.xhtml

The objective of **breast cancer screening** is to detect tumours early, at a preclinical stage. This means that better treatment options can be used and reduces mortality[1,2]. Despite these benefits, screening may also cause adverse effects: false negatives, false positives, overdiagnosis and overtreatment[2,3,4]. When making a decision about screening, how much these positive and negative effects will affect each woman is unknown. The **Shared Decision-Making** (SDM) model enables patients and health professionals to reduce the uncertainty surrounding this decision[5,6].

SDM has mainly been used in Western countries to improve decisions on health, drawing on patients' preferences and scientific evidence[7]. In 2012, the European Patients' Forum launched the *"Nothing about me, without me"* campaign[8] to involve people in decisions about their health[9].

In this context, some studies have explored how SDM is being implemented. For example, only 24% of patients in Spain said they had made a shared decision with their health professional, taking into account their personal or social characteristics and preferencess[10]. This demonstrates the failure of

For peer review only

strategies aimed at involving people in their health, due to potential shortcomings in communication skills and the lack of channels for productive dialogue between the various actors in the clinical encounter[11].

To improve these strategies, the **Health Quality and Assessment Agency of Catalonia** describes Patient decision aids (PtDAs) as a key element in SDM, although it does not currently provide one for breast cancer screening[12]. However, a recent study developed a PtDAs for this purpose[13].

While there has recently been an increase in the amount of materials developed for patients[14], there are few resources to help professionals incorporate people's preferences and values into decision-making about health. This document offers health professionals scientific evidence on SDM, so that it can be applied to the breast cancer screening process.

For peer review only

For peer review only

Shared Decision-Making

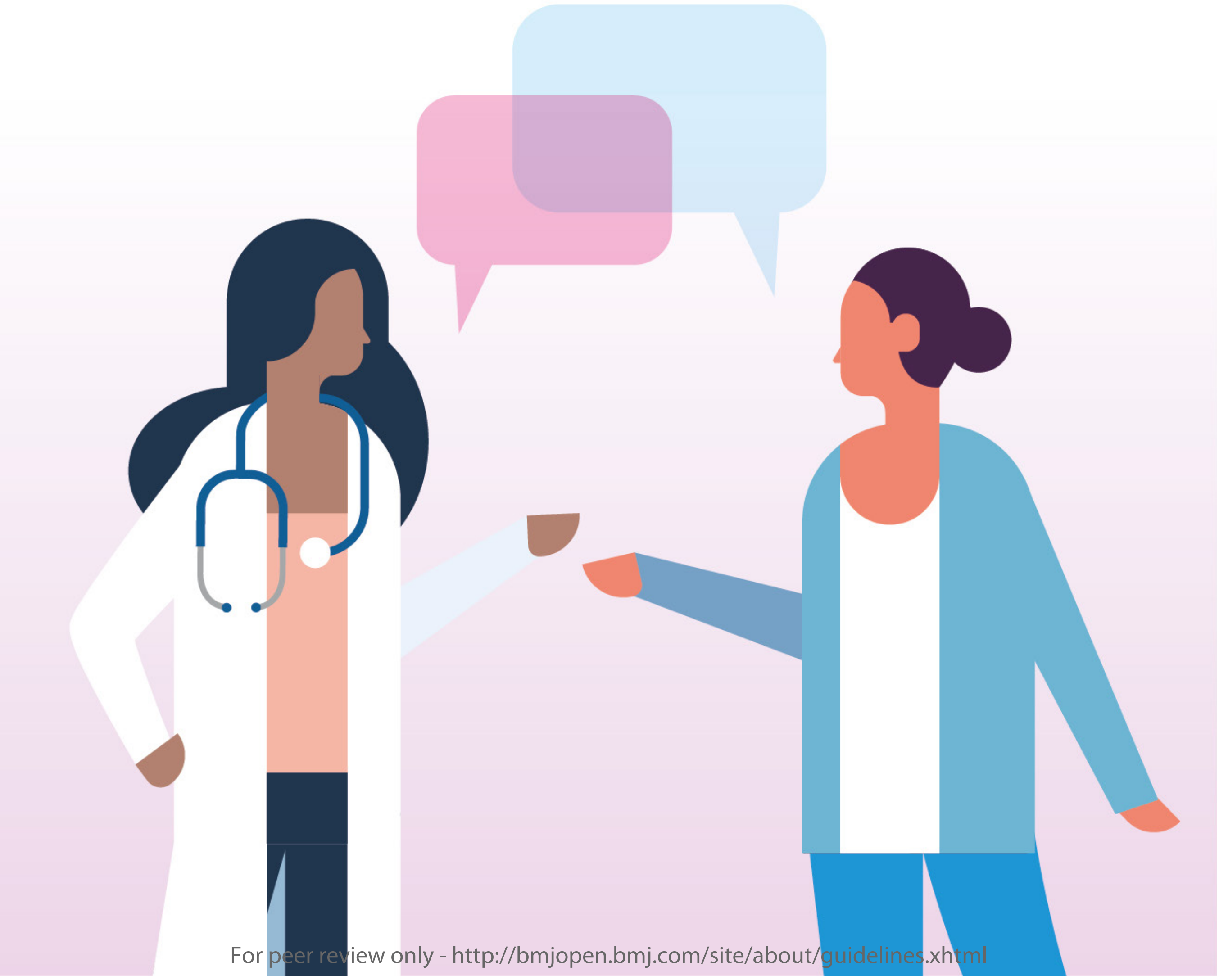

For peer review only - http://bmjopen.bmj.com/site/about/guidelines.xhtml

1. What is it?

Shared Decision-Making (SDM) was developed in the 1960s-1970s as part of a participatory care model that was situated between the paternalistic and the informative styles of care[15,16].

Figure 1: Healthcare models

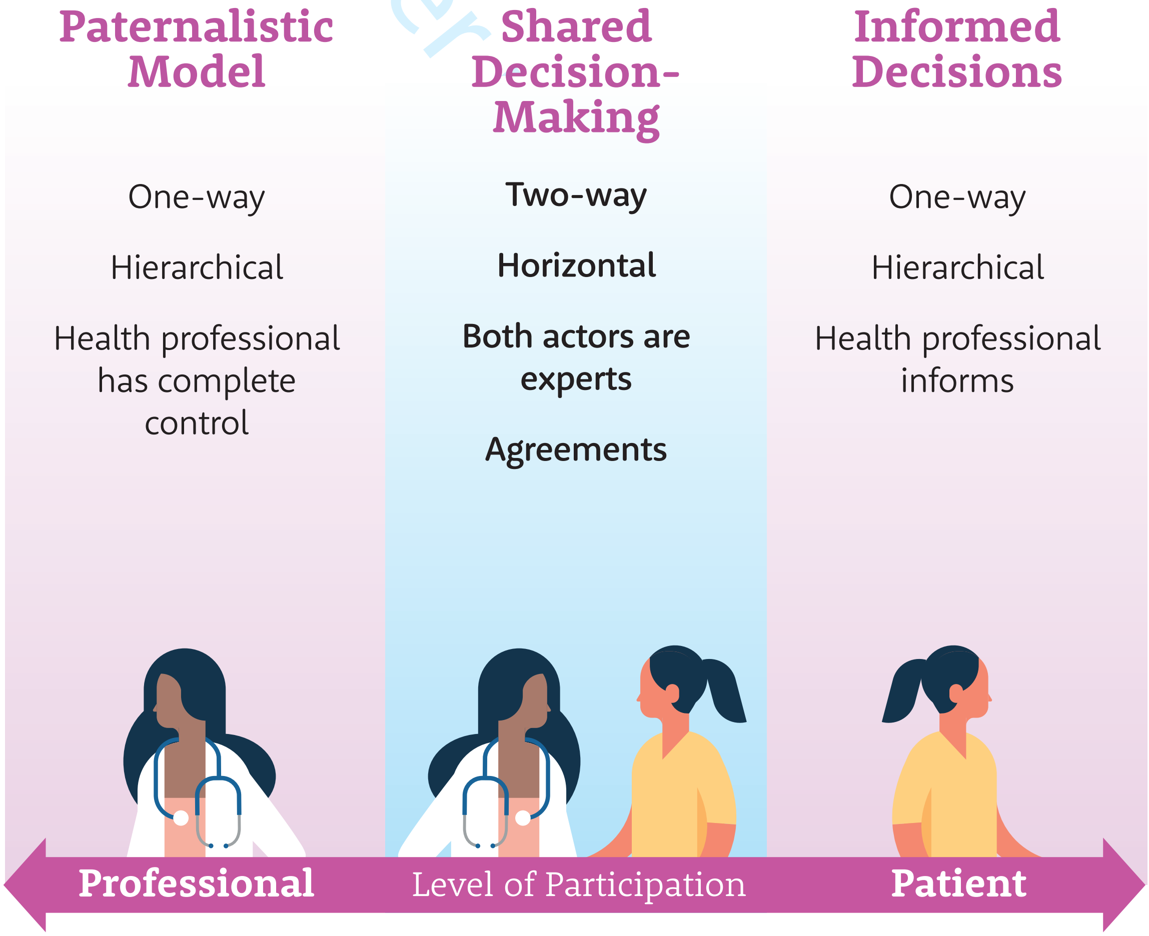

Source: Developed by the ProShare group

For peer

SDM promotes the participation of patients[17] in making a shared decision with their health professional on changes to their life style, diagnostic tests, treatments and therapeutic interventions in which there may be a degree of uncertainty[18,19]. SDM is carried out during the clinical encounter, and both actors are considered experts: the patient on his or her health, values, beliefs and preferences; and the health professional on the scientific evidence and information about the available therapeutic options[20].

Figure 2: Role of the participants in the clinical encounter

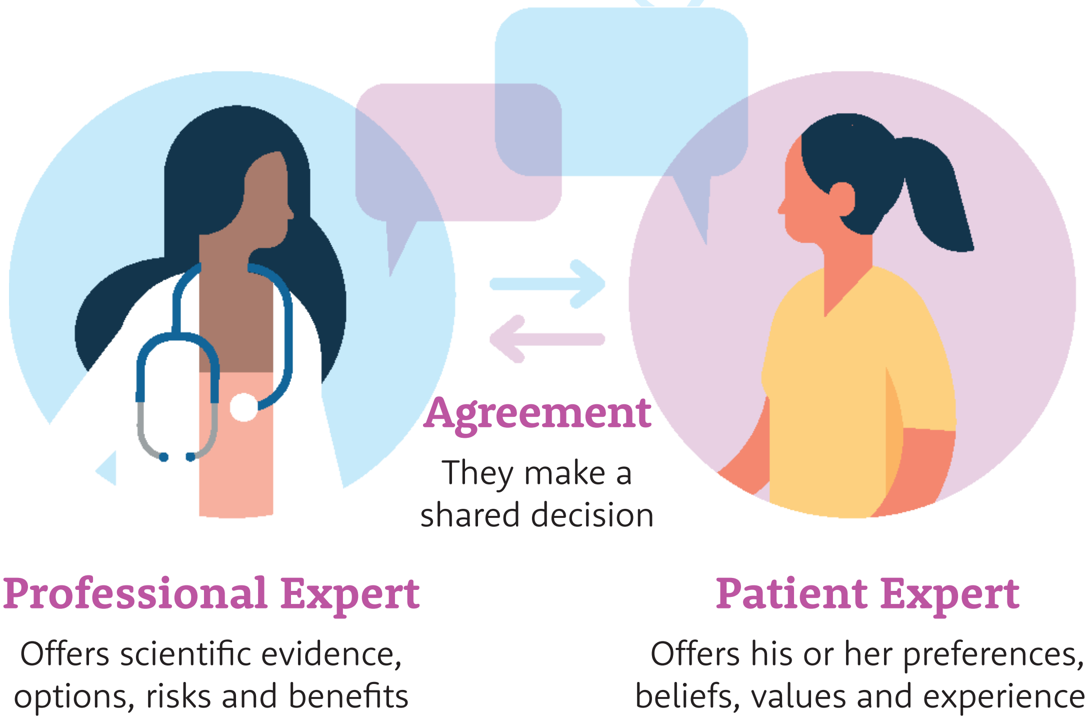

Source: Developed by the ProShare group

Therefore, the main objective of SDM is to guarantee that people are sufficiently informed when they make decisions about their health[21]. Achieving this goal requires continuous negotiation between the two experts, with the dialogue focusing on the values, preferences and circumstances of the patient, and the benefits, harms, risks and therapeutic options put forward by the health professional. As a result, this discussion creates more autonomous people with a greater sense of commitment and responsibility towards their health[20,22–24].

For peer review

Figure 3: Components of Shared Decision-Making

A) Exchange of information between the patient and health professional	B) Deliberation about the different options	**C) A shared decision is made**

Source: Adaptation of Elwyn et al. 2012[20]

2. Why is it important?

SDM is based upon the principle of patient autonomy. Spanish Law 21/2000 on Rights to Information about Health, Patient Autonomy and Clinical Documentation protects patients' right to decide freely between the available clinical options after having received appropriate information on them[25]. Health professionals are therefore legally bound to comply with this principle, and may not be guided solely by the professional's intentions.

Additionally, implementing SDM has been proven to create a number of benefits for patients, professionals and health services[10, 24, 26, 27]:

- Increases patient participation.
- Improves communication between the patient and health professional.
- Improves adherence to treatment.
- Improves biometric health results.
- Increases patient satisfaction with healthcare.

For peer review only - http://bmjopen.bmj.com/site/about/guidelines.xhtml

- Reduces patients' level of worry and anxiety.
- Reduces the amount of conflict patients feel when they have to make a diagnostic and/or therapeutic decision.
- Improves patients' knowledge of the disease, and the diagnostic and therapeutic options.
- Increases the precision of risk perception.
- Increases the number of patients choosing more beneficial options.
- Reduces the use of highly invasive and costly treatment.
- Reduces unjustified variability in care practice.
- Contributes to streamlining the use of health service resources.

only

3. The limitations of the model

There are still few studies that have monitored patients over an extended period to clearly determine the long-term impact of using the model. Additionally, there is a belief among health professionals that they are already applying SDM[28]. However, some studies have demonstrated that this assumption is not reflected in practice[29,30]. Finally, although patients tend to choose the same options as when SDM is not used, in a breast cancer screening context it has been shown that women value the reduction of mortality almost 5 times more than the risk of overdiagnosis[31], and this difference results from their increased knowledge, a sense of affinity with what has been decided, and feeling less conflicted about the decision.

For peer review only

4. What skills or competencies do health professionals need?

Communicating the risks and benefits of a therapeutic option in a balanced way is no easy task[26]. To achieve this, two types of competencies that health professionals should apply to SDM have been identified[32]:

4.1 Relationship-building competencies

These skills create a comfortable atmosphere, helping patients to share their concerns. To achieve this, the health professional must have a genuine desire to engage with the process, understand the patient's point of view and use simple language.

Some of the most important professional competencies include:

For peer review only

- Engaging in active listening.
- Respecting the decisions made by the patient.
- Asking open questions.
- Maintaining eye contact throughout.
- Letting the patient set the pace.
- Recognising the patient's emotional and verbal cues.
- Using communication skills, such as summarising, clarification, reflection and empathy[33].

4.2 Risk Communication Competencies

These are the skills that enable professionals to discuss uncertainties with the patient and communicate the risks and benefits of the various options effectively. The evidence should be assessed in relation to each unique context, taking into account the patient's personal background: the family history, medical history, risk factors and protective factors that could increase or decrease the benefits/harms caused by each option[32].

It is recommended that professionals avoid technical language, adapt the amount of information to the patient's current needs, use diagrams, check understanding of the information given, incorporate the patient's values into the evidence offered, provide objective information, promote participation and evaluate the information the patient already has[34].

In summary, the following skills are key for developing an optimum relationship with the patient[33] (Figure 4).

Figure 4: Communication skills

Listening	Language	Non-verbal	Cultural	Attitudinal
General and active listening	**Verbal:** an appropriate tone, adapted to the person's level of education<br>**Written:** clear communication and use of educational material	**Expressive:** body language and eye contact<br>**Receptive:** responding to body language and emotions	Adapting communication to the person's culture, age and disease	Respecting the patient's opinions and right to decide

Source: Adaptation of Laughlin T, Wetmore S, Allen T, Brailovsky C, Crichton T, Bethune C, Donoff M, Lawrence K. 2012[34].

For peer review

A Handbook to Support the Implementation of SDM

21

5. What do patients think?

A study conducted in Spain in 2012 found that 60% of patients would have liked the health professional to seek their opinion, but were not asked. Most of them would also have liked to receive more information than they were given[35]. Other studies of breast cancer screening have shown that only 8% to 10% of women received information about overdiagnosis[36].

Patients think that health professionals should engage with their patients' decisions, rather than leaving them to go through the decision process alone[37]. In 2013, another study identified which aspects of SDM patients' value most. The most important are: the health professional's communicative role; feeling that the professional is engaging in empathetic listening and showing real concern for their health and needs; that the conversation fits the context, and seeing that the professional has a good command of the information[38].

For peer review only

SDM strengthens the health professional-patient relationship and the therapeutic alliance because patients' active participation in decisions about their health reduces uncertainty, increases knowledge and improves their ability to manage their disease[37]. In short, patients gain greater peace of mind.

For peer review only

For peer review only

Shared Decision-Making in Breast Cancer Screening

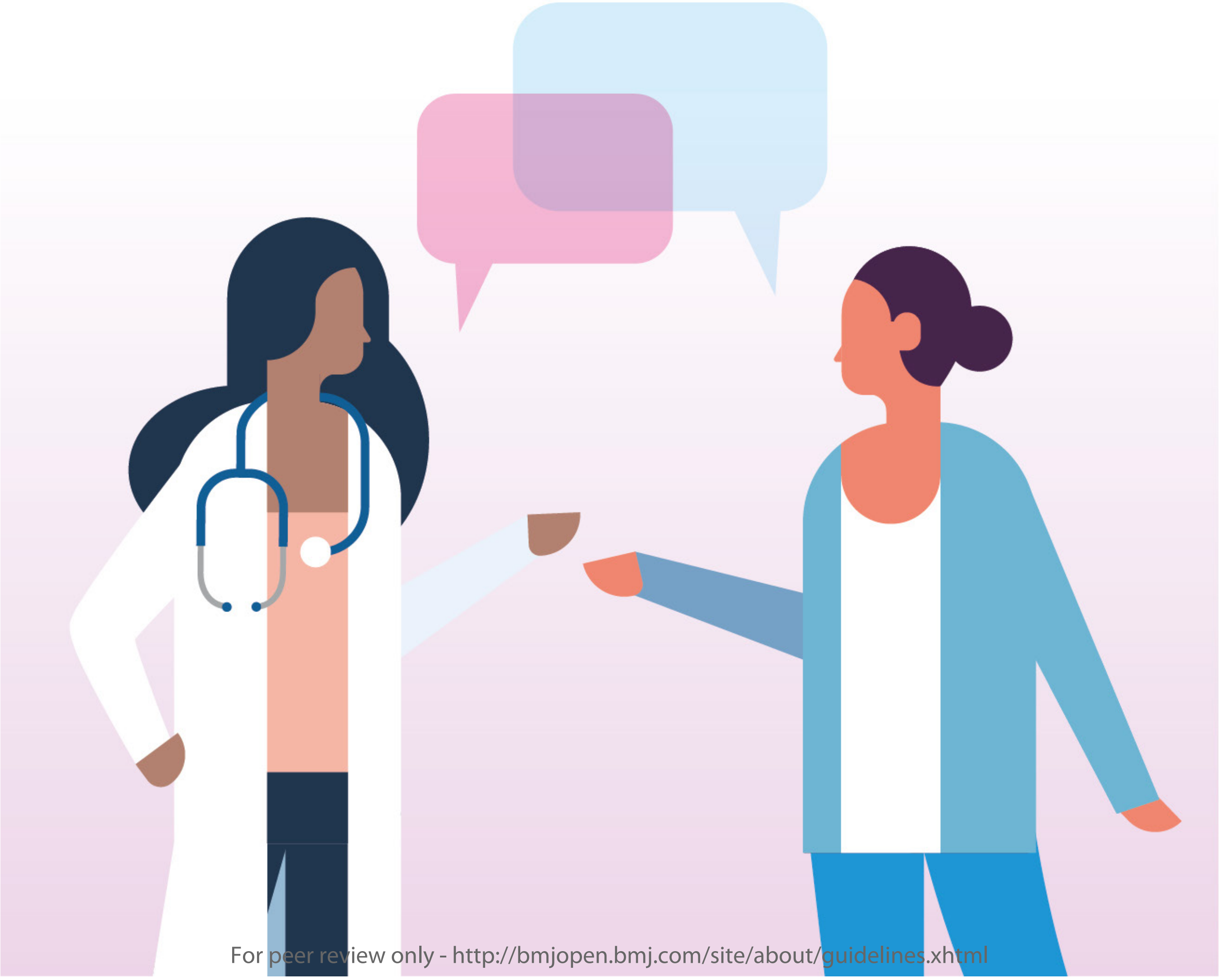

For peer review only - http://bmjopen.bmj.com/site/about/guidelines.xhtml

1. The screening programme

1.1 Population screening in Catalonia, Spain

The Strategic Plan contained within *the Regional Government of Catalonia's Oncology Master Plan* mentions the objective of reducing the impact of breast cancer on the population through screening[39]. However, it makes no reference to how women will be involved in decisions made about their health, despite the fact that their participation is one of the cornerstones of the Health Plan of Catalonia 2016-2020[40].

Currently, each screening programme set out in the Oncology Master Plan is conducted every two years. Using the Central Registry of Insured Individuals (RCA), women between 50 and 69 years of age are recruited via a letter sent to their home address. This letter invites them to have a free mammogram at a preassigned health centre[41]. This system does not provide a setting in which the health professional and the woman can meet for her to resolve any doubts or concerns, nor does it enable enough information to

For peer review only

https://www.youtube.com/watch?v=qwyx7yAP5zA&t=4s

- SHARE Approach — Shared Decisionmaking Demonstration. Agency for Healthcare Research and Quality: *https://www.youtube.com/watch?v=zpZ8JgE8DZc*

1.2 Why apply SDM to breast cancer screening?

Diagnostic tests for the early detection of breast cancer have become established as the standard in public health due to the demonstrated reduction in mortality. For every 200 women aged between 50 and 69 who have screening mammograms every two years, 1 woman is saved thanks to early detection of a tumour, and 40 need further tests to rule out cancer[13]. However, recent studies show that there is little or no awareness of the harms or adverse effects of these tests.

The main risks attributed to breast cancer screening are false positives, false negatives and overdiagnosis[19]. This last concept is defined as tumours which grow so slowly that they would

For peer review only - http://bmjopen.bmj.com/site/about/guidelines.xhtml

never become a health problem and would even disappear of their own accord, without the need for treatment. Currently, it is not known which types of lesions will progress and which will not, so treatment is offered to all women diagnosed with breast cancer (this is known as overtreatment)[13]. This creates a sense of fragility, vulnerability and intolerance of uncertainty in women, is linked to higher-risk procedures such as biopsies[43] and translates into increased public health spending[8].

In a context of uncertainty about the benefits and adverse effects, using SDM is recommended in order to reach a decision about whether to participate or not, on the basis of current scientific evidence and women's values.

You can find more information about definitions, incidence and statistics on the risks, benefits and adverse effects of breast cancer screening in the PtDAs developed in 2016[13].

For peer review only

2. Implementing SDM in breast cancer screening

Before beginning the SDM process, a relationship of trust that is based on empathy should be established. This will facilitate fluid, high-quality communication[23,44]. The process should be deliberative, meaning that women understand they will be making a decision and that more than one clinical encounter may be necessary for this[21]. It should also be dynamic, as the stages must be adapted to the needs, concerns and priorities of each woman[45].

Once the information has been provided, there should be an explicit exploration of whether the woman wants to take an active or passive role in the decision[23,46]; not doing so may lead her to take a passive role[17,23,46]. Nevertheless, professionals may still check which role she wishes to take throughout the clinical encounter, given that this may change from active to passive or vice versa over the course of the conversation.

For peer review only

Factors that promote participation of the professional and the woman include: health professionals' motivation to involve the woman in her decision; the perception that SDM will have a positive impact on the clinical process; the woman having a high level of literacy, or her own desire to take an active part in decisions that affect her health[26,47]. In contrast, the following factors can impede SDM: time limitations in the clinical encounter; the woman being of an advanced age, having difficulty communicating in the language of the professional, being from an underprivileged socioeconomic background, or having a low level of literacy[23,26,46,47]; and the existence of mental health conditions[23,43].

2.1 The "Three-talk" model and steps

The three steps involved in this model are[48]:

STEP 1
Team talk

The main objective of this step is to communicate the need to make a decision as a team, whose members are the health professional and the woman. This involves communicating the objectives of the decision, why it needs to be made (existence of personal risk factors) and the options available on the basis of the evidence. Professionals should (a) stress that the woman may decide not to make a decision at that time and request the support of other actors, such as relatives or other specialists, and (b) be receptive to the reactions the woman may have upon facing this decision. They should therefore underline that they will support the woman through the process until she feels confident enough to make the decision.

For peer review only

Figure 5: Team talk

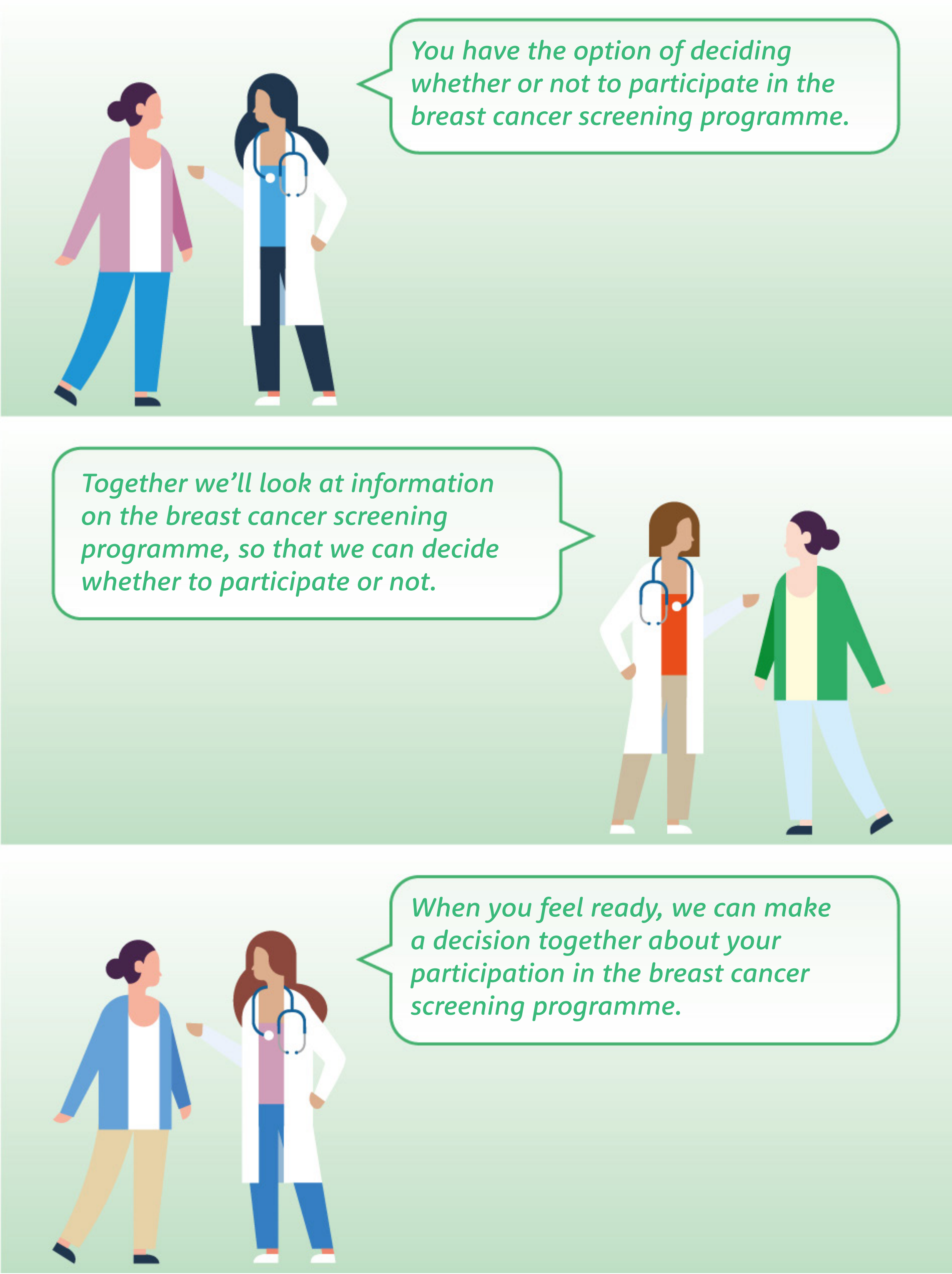

Source: Developed by the ProShare group.

A Handbook to Support the Implementation of SDM

33

STEP 2

Option talk and exploring preferences

The main objective of this step is to provide clear information, adapted to each woman's characteristics, about the adverse effects and benefits of screening. This should involve exploring her values, concerns, expectations and initial preferences (priorities based on pre-existing knowledge or preconceived ideas about screening[21]). The conversation should also explore the options in detail, considering risks and benefits. Through this process, the woman's initial preferences will become informed preferences (personal preferences based on her values, after her understanding of the most important risks and benefits of screening has been ensured[21]).

Using a PtDAs to explain the specific risks is recommended, as this will improve understanding of the information, even for women with a low level of literacy[13].

For peer review only

Figure 6: Option talk and exploring preferences

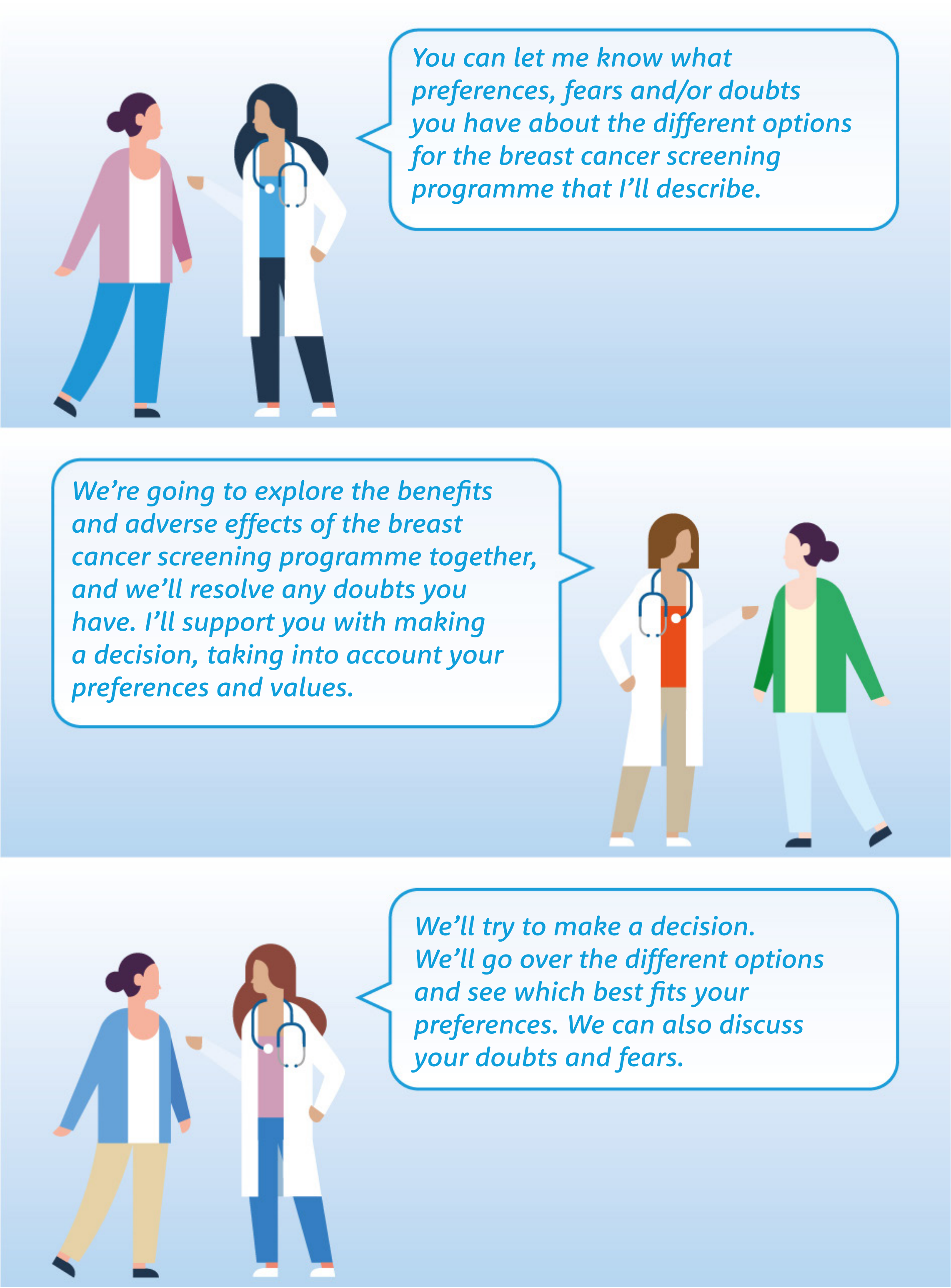

Source: Developed by the ProShare group.

STEP 3
Decision talk

At this step, the participants discuss the various options and make a decision on participation in breast cancer screening[48]. Professionals should underline the fact that they will support the woman in making the decision. They should also provide enough time for her to reflect on her priorities, even bringing up the option of delaying the decision to a later date or delegating it to the professional. In the latter case, it is recommended that participants identify the factors that are stopping her from making the decision by herself. Finally, they should confirm the decision, then agree on an action and follow-up plan[23,46] that enables the professional and woman to exchange ongoing feedback[45,46].

Figure 7: Decision talk

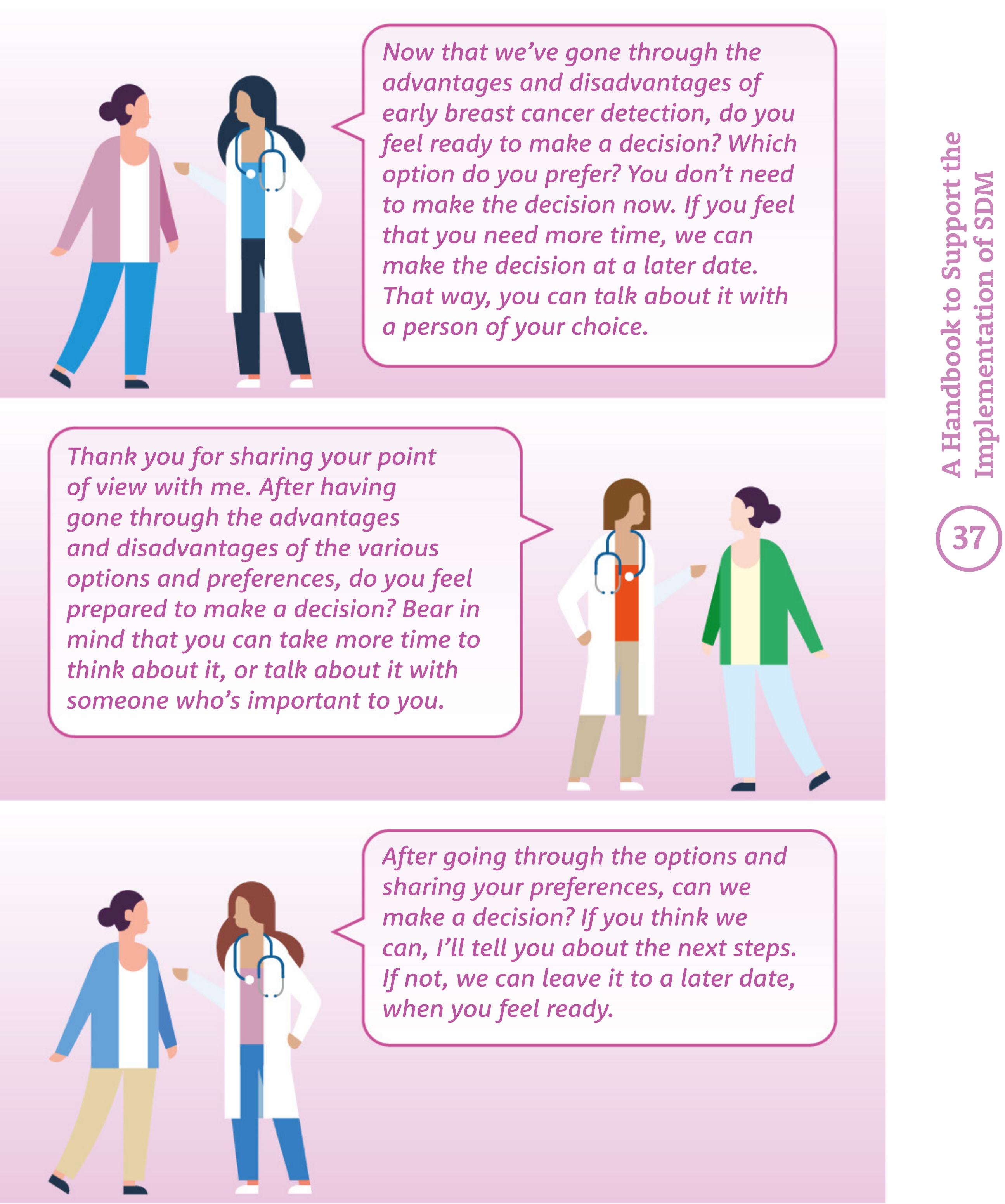

Source: Developed by the ProShare group.

Figure 8: The steps of Shared Decision-Making

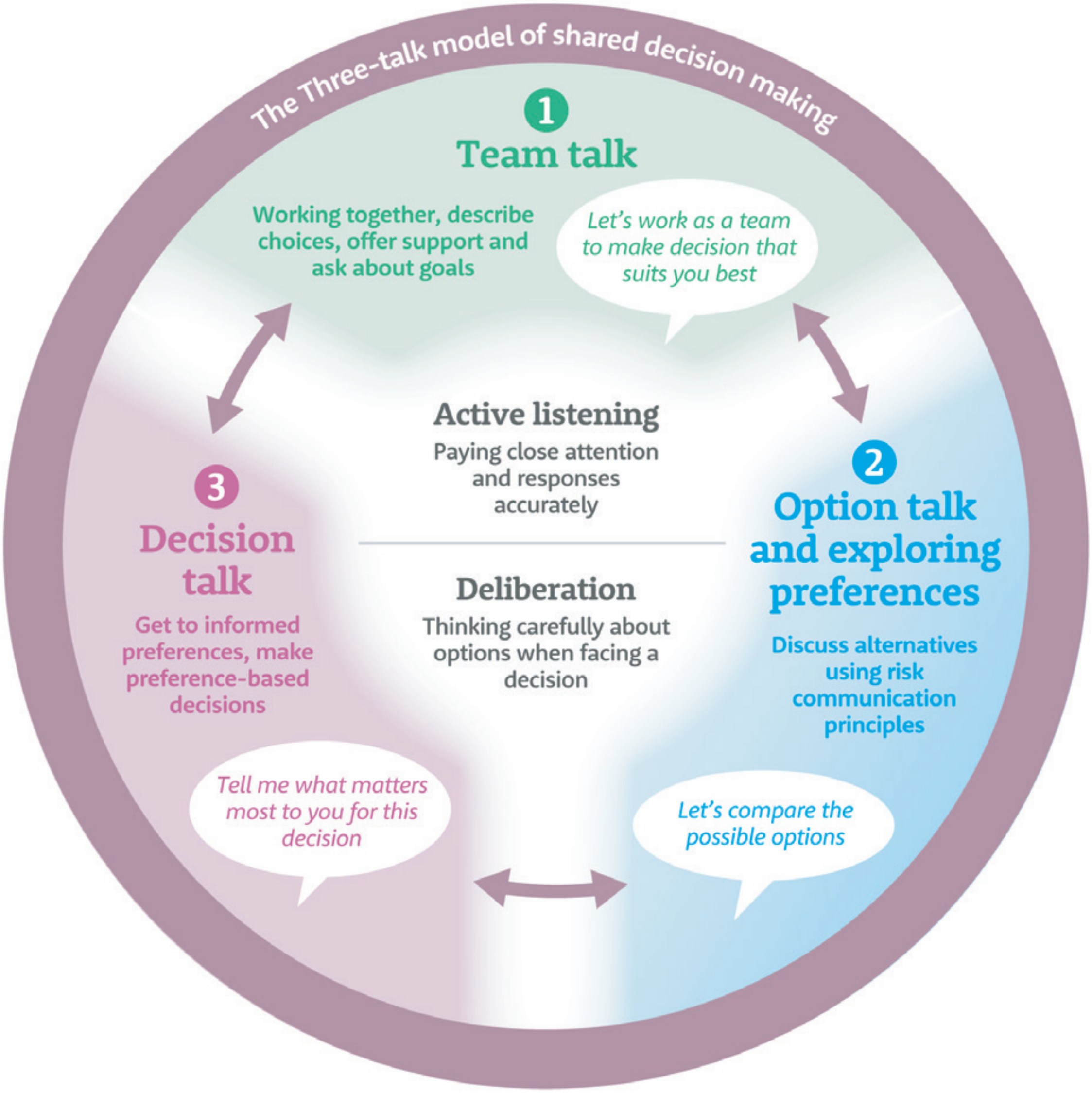

Source: Adaptation of A three-talk model for shared decision making. Elwyn G, et al 2017. Use authorised by the author[48].

3. Self-assessment of the SDM process

The SDM-Q-doc[49] instrument is a self-assessment questionnaire for health professionals that enables them to measure the level of participation the woman has been offered when making decisions. This instrument consists of nine items that the professional has to rate on a scale of one to six: from completely disagree (1) to completely agree (6).

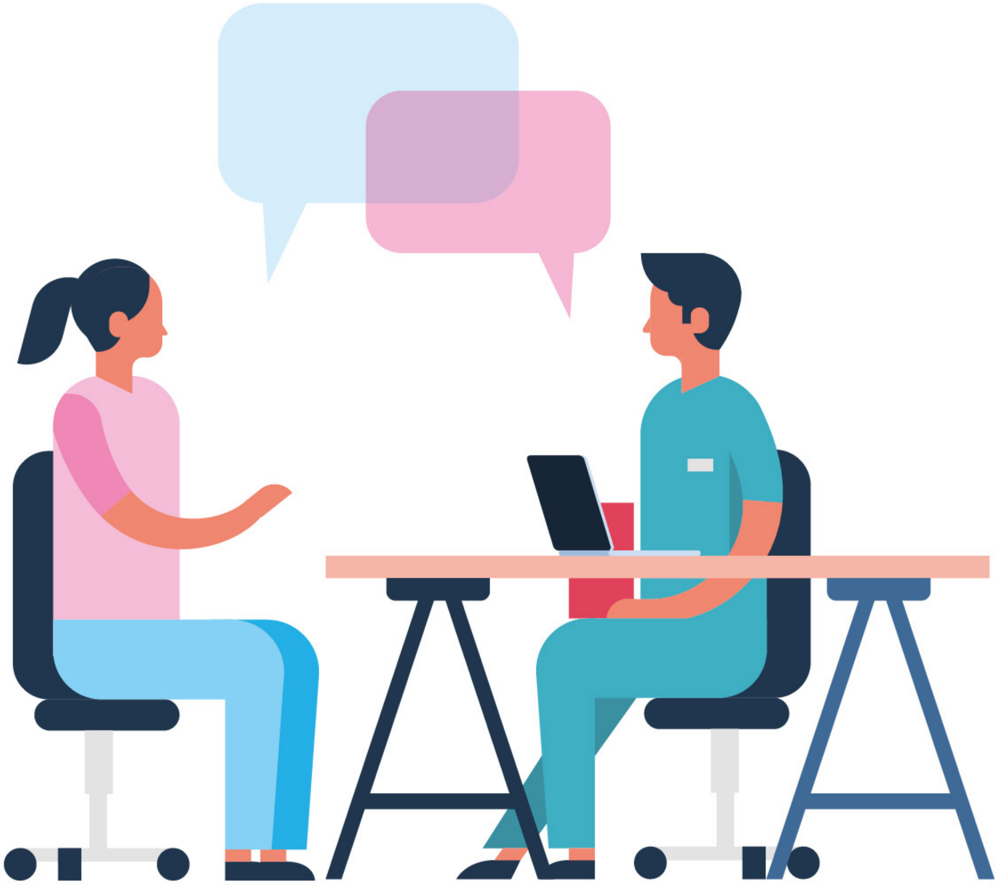

Criteria	Points*					
	1	2	3	4	5	6
I made it clear to the woman that a decision needs to be made on her participation in breast cancer screening	☐	☐	☐	☐	☐	☐
I asked the woman exactly how she wants to be involved in making the decision	☐	☐	☐	☐	☐	☐
I informed the woman that she has the option to participate or not in screening	☐	☐	☐	☐	☐	☐
I precisely explained the advantages and disadvantages of each option to the woman	☐	☐	☐	☐	☐	☐
I helped the woman understand all the information about benefits and adverse effects	☐	☐	☐	☐	☐	☐
I asked the woman which option she prefers	☐	☐	☐	☐	☐	☐
The woman and I thoroughly weighed all the options	☐	☐	☐	☐	☐	☐
The woman and I selected an option together	☐	☐	☐	☐	☐	☐
The woman and I have come to an agreement about how to proceed with her subsequent healthcare	☐	☐	☐	☐	☐	☐

* The scale ranges from: “Completely disagree” (1) to “Completely agree” (6)

Source: SDM-Q-doc adapted to breast cancer screening.

For peer review only

The interpretation and score were based on the evidence presented by Pollard, Bansback and Bryan (2015)[5]. The total number of available points (54 points) was divided according to the following percentages: >80% "Strong support in favor of SDM"; 60-80% "Mild support for SDM"; 40-60% "Indifference from the professional towards SDM" and <40% "Lack of support from the professional for SDM". The score boundaries for each stage define those who score over 60% as showing "support" for SDM and the rest as showing a "lack of support" for SDM, out of a total of 15 points (3 to 18). Finally, this division into stages is an adaptation of the SDM-Q-doc scale, cross-referencing it with the characteristics of the Three-talk model[48].

Completing this self-assessment regularly is recommended, as it enables health professionals to identify strengths and weaknesses in how tahree ypromoting women's involvement in their ohweanlth. This helps them to focus their efforts on improving their weakest points through education and training, and to monitor their progress.

Bibliographic References

1. World Health Organization. (WHO) Breast Cancer Control; 2020 [accessed 30 June 2020]. Available from: https://www.who.int/cancer/detection/breastcancer/en/
2. Febles G. Revisión de los beneficios del screening del cáncer de mama y análisis de las controversias (Review of the benefits of breast cancer screening and analysis of the controversies). *Rev. Imagenol.* [Internet] 2015;19 (1)9-18. Available from: https://www.webcir.org/revistavirtual/articulos/2016/4_noviembre/ur/febles_esp.pdf
3. Day N. Overdiagnosis and breast cancer screening. *Breast Cancer Res* [Internet]. 2005;7(5):228–9. Available from: http://www.ncbi.nlm.nih.gov/pubmed/16168144
4. Nehmat H. Overdiagnosis of breast cancer in population screening: does it make breast screening worthless? *Cancer Biol Med* [Internet]. 2017;14(1):1–8. Available from: http://www.cancerbiomed.org/index.php/cocr/article/view/1003
5. Pollard S, Bansback N, Bryan S. Physician attitudes toward shared decision making: A systematic review. *Patient Educ Couns* [Internet]. 2015; 98(9):1046-57. Available from: https://www.sciencedirect.com/science/article/abs/pii/S0738399115002256?via%3Dihub
6. Munthe C, Sandman L, Cutas D. Person Centred Care and Shared Decision Making: Implications for Ethics, Public Health and Research. *Health Care Anal* [Internet]. 2012;20(3):231–49. Available from: http://link.springer.com/10.1007/s10728-011-0183-y
7. Barry M, Edgman-Levitan S. Shared Decision Making — The Pinnacle of Patient-Centered Care. *N Engl J Med* [Internet]. 2012;366(9):780–1. Available from: http://www.nejm.org/doi/10.1056/NEJMp1109283
8. Coulter A, Collins A. Making shared decision-making a reality. No decision about me, without me [Internet]. The King's Fund. Edwina Rowling, editor. London; 2011. Available from: www.kingsfund.org.uk

For peer review only

9. European patients' forum. 150 Million reasons to act; *EPF's Patients' Manifesto for the European Parliament and Commission*. [Internet]. 2009. Available from: https://www.eu-patient.eu/globalassets/events/old/manifesto/epf_manifesto.pdf

10. Confederación Crohn y Colitis Ulcerosa. Se incumple el modelo de decisiones compartidas con los pacientes (The model of shared decisions involving patients is not fulfilled) [Internet]. Available from: https://www.accuesp.com/se-incumple-el-modelo-de-decisiones-compartidas-con-los-pacientes

11. Ministerio de Sanidad, Consumo y Bienestar Social de España. Marco Estratégico para la Atención Primaria y Comunitaria (Strategic Framework for Primary and Community Care) [Internet]. 2019. Available from: https://www.mscbs.gob.es/profesionales/proyectosActividades/docs/Marco_Estrategico_APS_25Abril_2019.pdf

12. Agencia de Calidad y Evaluación Sanitarias de Cataluña (AQuAs). Estado de situación de las herramientas de ayuda a la toma de decisiones clínicas en Cataluña (State of the art of clinical decision support tools in Catalonia). [Internet]. 2020 [accessed 30 June 2020]. Available from: http://aquas.gencat.cat/ca/ambits/avaluacio-tecnologies-qualitat/guies/eines_ajut_presa_decisions_catalunya/

13. Toledo-Chavarri A, Rué M, Codern-Bove N, Carles-Lavila M, Perestelo-Perez L, Perez-Lacasta MJ, and Feijoo-Cid M. A Qualitative Study on a Decision Aid for Breast Cancer Screening: Views from Women and Health Professionals. *Eur J Cancer Care (Engl)* [Internet]. 2017. 26(3). Available from: https://onlinelibrary.wiley.com/doi/abs/10.1111/ecc.12660

14. Grupo Español de Investigación en Cáncer de Mama. Guía GEICAM de Práctica Clínica: Para el Diagnóstico y Tratamiento del Cáncer de Mama Metastásico (GEICAM Clinical Practice Guidelines: For the diagnosis and treatment of metastatic breast cancer). Published by: Comuniland S.L. Madrid; 2015. Available from: https://portal.guiasalud.es/wp-content/uploads/2019/06/gpc_538_geicam_cancer_mama_resumida.pdf

15. Charles C, Gafni A, Whelan T. Self-reported use of shared decision-making among breast cancer specialists and perceived barriers and facilitators to implementing this approach. *Health Expect* [Internet]. 2004 Dec;7(4):338–48. Available from: http://www.ncbi.nlm.nih.gov/pubmed/15544686

For peer review only

16. Barr P, Scholl I, Bravo P, Faber M, Elwyn G, McAllister M. Assessment of Patient Empowerment - A Systematic Review of Measures. *PLoS One* [Internet]. 2015;10(5). Available from: http://journals.plos.org/plosone/article/file?id=10.1371/journal.pone.0126553&type=printable

17. Elwyn G, Edwards A, Kinnersley P, Grol R. Shared decision making and the concept of equipoise: the competences of involving patients in healthcare choice. *Br J Gen Pract* [Internet]. 2000;50:892–7. Available from: https://bjgp.org/content/bjgp/50/460/892.full.pdf

18. Participa y decide sobre tu salud. ¿Qué es la toma de decisiones compartida? (Participate and decide on your health: What is shared decision making?) [Internet]. 2020 [access ed 30 June 2020]. Available from: https://www.pydesalud.com/toma-de-decisiones-compartidas/

19. Marmot M, Altman D, Cameron D, Dewar J, Thompson S, Wilcox M. The benefits and harms of breast cancer screening: An independent review. *Br J Cancer* [Internet]. 2013;108(11): 2205–2240. Available from: https://www.ncbi.nlm.nih.gov/pmc/articles/PMC3693450/

20. Reyes A. Toma de decisiones compartidas: por una medicina cuidadosa y amable. Parte 1 (Shared decision making: A case for caring, kind medicine. Part 1) [Internet]. *Por una Medicina Interna de Alto Valor* (A Case for High-quality Internal Medicine). 2018. Available from: https://medicinainternaaltovalor.fesemi.org/el-universo-del-paciente/toma-de-decisiones-compartidas-por-una-medicina-cuidadosa-y-amable-parte-1/

21. Elwyn G, Frosch D, Thomson R, Joseph-Williams N, Lloyd A, Kinnersley P, et al. Shared decision making: a model for clinical practice. *J Gen Intern Med* [Internet]. 2012;27(10):1361–7. Available from: http://www.ncbi.nlm.nih.gov/pubmed/22618581

22. Peralta L, Ruiz J, Castillo R. La participación del paciente en la toma de decisiones en las consultas de atención primaria (Patient participation in decision-making in primary care practices). *Universidad de Cantabria*. 2010; Available from: http://www.tesisenred.net/bitstream/handle/10803/10705/TesisLPM.pdf?sequence=1

For peer review only

23. Información farmacoterapéutica de la Comarca. Participación del paciente en la toma de decisiones (Patient participation in decision-making). *INFAC* [Internet]. 2014;22(3):12–7. Available from: https://www.euskadi.eus/contenidos/informacion/cevime_infac_2014a/es_def/adjuntos/INFAC_Vol_22_n_3_Decisiones_compartidas.pdf

24. Bravo P, Contreras A. Perestelo-Pérez L, Pérez-Ramos J, Málaga G. En busca de una salud más participativa: Compartiendo decisiones de salud (In search of more participatory health: Sharing decisions on health). *Rev Peru Med Exp Salud Publica*. 2013;30(4):691-7.

25. Boletín Oficial del Estado. Ley 14/1986 de 2 de febrero, General de Sanidad (General Law 14/1986 of 2 February on Health). BOE de 21/2000. [Internet]. Available from: https://www.boe.es/boe/dias/2001/02/02/pdfs/A04121-04125.pdf

26. Gravel K, Légaré F, Graham I. Barriers and facilitators to implementing shared decision-making in clinical practice: a systematic review of health professionals' perceptions. *Implementation Sci*. 2006;1(16). Available from: http://www.implementationscience.com/content/1/1/16

27. Légaré F, Ratté S, Stacey D, Kryworuchko J, Gravel K, Graham ID, et al. Interventions for improving the adoption of shared decision making by healthcare professionals. *Cochrane Database of Systematic Reviews* [Internet]. Chichester, UK: John Wiley & Sons, Ltd; 2010. p. CD006732. Available from: http://www.ncbi.nlm.nih.gov/pubmed/20464744

28. Stiggelbout AM, Pieterse AH, De Haes JC. Shared decision making: Concepts, evidence, and practice. *Patient Educ Couns.* 2015;(10):1172-9. doi: 10.1016/j.pec.2015.06.022. Epub 2015 Jul 15. PMID: 26215573.

29. Hernández-Leal M, Perestelo-Pérez L, Bravo P. Percepción de los profesionales de salud para la Toma de Decisiones Compartida en Atención Primaria (Health professionals' perception of Shared Decision Making in primary care). *Revista chilena de medicina familiar*. Forthcoming.

30. Couët N, Desroches S, Robitaille H, Vaillancourt H, Leblanc A, Turcotte S, Elwyn G, Légaré F. Assessments of the extent to which health-care providers involve patients in decision making: a systematic review of studies using the OPTION instrument. *Health Expect.* 2015;18(4):542-61. doi: 10.1111/hex.12054.

For peer review only

31. Pérez-Lacastra, Carles-Lavila M, Codern-Bové N, Cardona-Cardona A, Rué-Monné M. Eliciting women's preferences for the design of a breast cancer screening program. *Patient Educ Couns.* Forthcoming.

32. Légaré F, Moumjid-Ferdjaoui N, Drolet R, Stacey D, Härter M, Bastian H, et al. Core competencies for shared decision making training programs: insights from an international, interdisciplinary working group. *J Contin Educ Health Prof* [Internet]. 2013;33(4):267–73. Available from: http://www.ncbi.nlm.nih.gov/pubmed/24347105

33. Ford S, Schofield T, Hope T. What are the ingredients for a successful evidence-based patient choice consultation? A qualitative study. *Soc Sci Med* [Internet]. 2003;56:589–602. Available from: https://pubmed.ncbi.nlm.nih.gov/12570976/

34. Laughlin T, Wetmore S, Allen T, et al. Defining competency-based evaluation objectives in family medicine: communication skills. *Can Fam Physician* [Internet]. 2012;58(4):e217–e224. Available from: https://www.ncbi.nlm.nih.gov/pmc/articles/PMC3325474/.

35. Ruiz R, Peralta L, Pérula L, Olloqui Mundet J, Carrión T, Sobrino A, et al. Opiniones y percepciones de los pacientes sobre su participación en la toma de decisiones en las consultas de medicina de familia (Patient opinions and perceptions about their participation in decision-making in family medicine practices). *Aten Primaria* [Internet]. 2012;44(1):5–10. Available from: https://www.sciencedirect.com/science/article/pii/S0212656711001545?via%3Dihub

36. Hersch, J. Jansen & K. McCaffery (2018) Decision-making about mammographic screening: pursuing informed choice, Climacteric, 21:3, 209-213, DOI: 10.1080/13697137.2017.1406912

37. Moreau A, Carol L, Dedianne M, Dupraz C, Perdrix C, Lainé X, et al. What perceptions do patients have of decision making (DM)? Toward an integrative patient-centered care model. A qualitative study using focus-group interviews. *Patient Educ Couns* [Internet]. 2012:1;87(2):206–11. Available from: https://www.sciencedirect.com/science/article/abs/pii/S0738399111004599

For peer review only

38. Thorne S, Oliffe JL, Stajduhar KI. Communicating shared decision-making: Cancer patient perspectives. Patient Educ Couns [Internet]. 2013;90(3):291–6. Available from: https://www.sciencedirect.com/science/article/abs/pii/S0738399112000912

39. Generalitat de Catalunya. Departament de Salut. Pla director 2017 (Master plan 2017). [Internet]. Available from: http://salutweb.gencat.cat/ca/ambits_actuacio/linies_dactuacio/estrategies_salut/cancer/pla_director/

40. Generalitat de Catalunya. Departament de Salut. Pla director D'Oncologia 2016-2020: Un sistema centrat en la persona: públic, universal i just (Oncology master plan 2016-2020: A person-centred system: public, universal and fair). 2016. [Internet]. Available from: http://salutweb.gencat.cat/web/.content/_departament/pla-de-salut/Pla-de-salut-2016-2020/documents/Pla_salut_Catalunya_2016_2020.pdf

41. Institut Català d'Oncologia. Detecció Precoç de Càncer de Mama (Early detection of breast cancer). [Internet]. 2020 [accessed 30 June 2020]. Available from: http://ico.gencat.cat/ca/el-cancer/programes_de_deteccio_precoc/programa_de_deteccio_precoc_de_cancer_de_mama/index.html

42. Servicio de epidemiología y Prevención del cáncer (SEPC). Esquema funcional del Programa de Detección Precoz del Cáncer de Mama (Functional outline of the Early Breast Cancer Detection Programme). [Internet]. 2020 [accessed 30 June 2020]. Available from: https://epicancer.cat/prevencion_cancer_mama.

43. Coll-Benejam T, Bravo-Toledo R , Marcos-Calvo M, Astier-Peña M. Impacto del sobrediagnóstico y sobretratamiento en el paciente, el sistema sanitario y la Sociedad (The impact of overdiagnosis and overtreatment on the patient, health service and society). *Aten Primaria.* [Internet]. 2018;50(S2):86-95. Available from: https://www.elsevier.es/es-revista-atencion-primaria-27-articulo-impacto-del-sobrediagnostico-sobretratamiento-el-S0212656718305158

44. Rowland K, Politi M. Shared Decision-Making and the Patient-Provider Relationship. In: Handbook of Health Decision Science [Internet]. New York, NY: Springer New York; 2016. p. 181–92. Available from: http://link.springer.com/10.1007/978-1-4939-3486-7_14

For peer review only - http://bmjopen.bmj.com/site/about/guidelines.xhtml

45. Elwyn G, Petronella Catharina Anna Vermunt N. Goal-Based Shared Decision-Making: Developing an Integrated Model. *Journal of Patient Experience*. 2019;1-9. Available from: https://journals.sagepub.com/doi/full/10.1177/2374373519878604

46. Barani M, Kopitowski K. Toma de decisiones compartidas: centrando los cuidados médicos realmente en nuestros pacientes (Shared decision making: Genuinely centring medical care around our patients). *Rev Hosp Ital BAires* [Internet]. 2013;33(2):60–4. Available from: https://www.hospitalitaliano.org.ar/multimedia/archivos/noticias_attachs/47/documentos/14745_60-64-HI-33-2-2BARANI_A.pdf

47. Hernández-Leal, MJ, Pérez-Lacasta MJ, Feijoo-Cid M, Ramos-García V, Carles-Lavila M. Healthcare professionals' behaviour regarding the implementation of shared decision-making in screening programmes: A systematic review. Patient Educ Couns [Internet]. 2021. https://doi.org/10.1016/j.pec.2021.01.032

48. Elwyn G, Duran M, Song J, Aarts J, J Barr P, Berger Z, etc al. A three-talk model for shared decision making: multistage consultation process. *BMJ*. 2017;359. [Internet]. Available from: https://www.bmj.com/content/359/bmj.j4891.long

49. De las Cuevas C, Perestelo-Perez L, Rivero-Santana A, Cebolla-Martí A, Scholl I, Härter M. Validation of the Spanish version of the 9-item Shared Decision-Making Questionnaire. *Health Expect* [Internet]. 2015;18(6):2143–53. Available from: https://pubmed.ncbi.nlm.nih.gov/24593044/

For peer review only

For peer review only

A Practical Guide to Implementing SDM for Health Professionals

1 Team talk

Communicate the need to make a decision

- Introduce the idea that the woman can make decisions on her health
- Talk about the risk factors and those that particularly affect her
- Stress that you will help her through the whole process and that she can also get support from relatives or other professionals

"Usted tiene la opción de decidir si participar o no en el programa de cribado de cáncer de mama."

"We're going to explore the benefits and adverse effects of the breast cancer screening programme together, and we'll resolve any doubts you have. I'll support you with making a decision, taking into account your preferences and values".

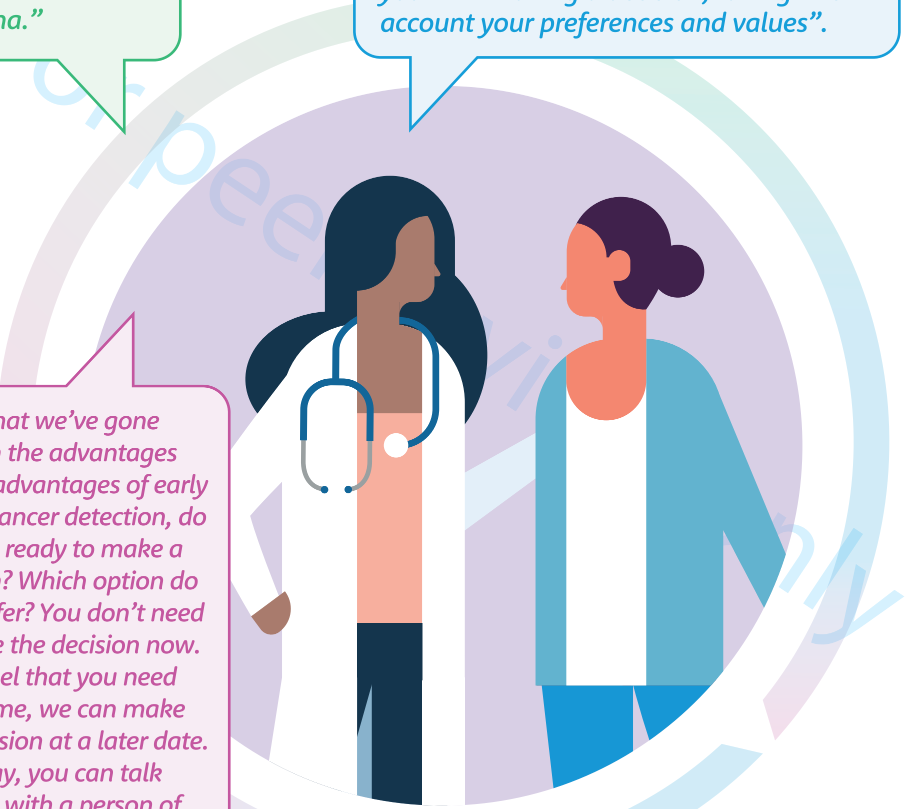

2 Option talk and exploring preferences

Inform the woman that she has the option of going for a mammogram or not

- Explore what the woman knows about the mammogram
- Introduce the adverse effects and benefits of the mammogram using a Patients Decision Aids (PtDAs)
- Consider the woman's preferences, beliefs, values and fears concerning the mammogram
- Summarise the options and check that the woman has understood the new information

3 Decision talk

Make a shared decision on the mammogram

- Provide the time she needs to think things through
- Clear up any doubts and evaluate her preference
- Design a plan for how to follow up on the decision

"Now that we've gone through the advantages and disadvantages of early breast cancer detection, do you feel ready to make a decision? Which option do you prefer? You don't need to make the decision now. If you feel that you need more time, we can make the decision at a later date. That way, you can talk about it with a person of your choice".

ProShare
SHARED DECISION MAKING IN HEALTH

Crosscutting Relationship-building Competencies

Empathy | Active listening | Assertiveness | Feedback | Adapting language | Eye contact

A Practical Guide to Implementing SDM for Health Professionals

ProShare
SHARED DECISION MAKING IN HEALTH

Results

Steps of SDM	Points	Interpretation
Step 1 **"Team talk"**	☐	3 to 12 points: lack of support for SDM<br>13 to 18 points: support for SDM
Step 2 **"Option talk and exploring preferences"**	☐	3 to 12 points: lack of support for SDM<br>13 to 18 points: support for SDM
Step 3 **"Decision talk"**	☐	3 to 12 points: lack of support for SDM<br>13 to 18 points: support for SDM
**Total score:**	☐	**9 to 27:** Lack of support for SDM<br>**28 to 36:** Indifference towards SDM<br>**37 to 45:** Mild support for SDM<br>**46 to 54:** Strong support in favor of SDM

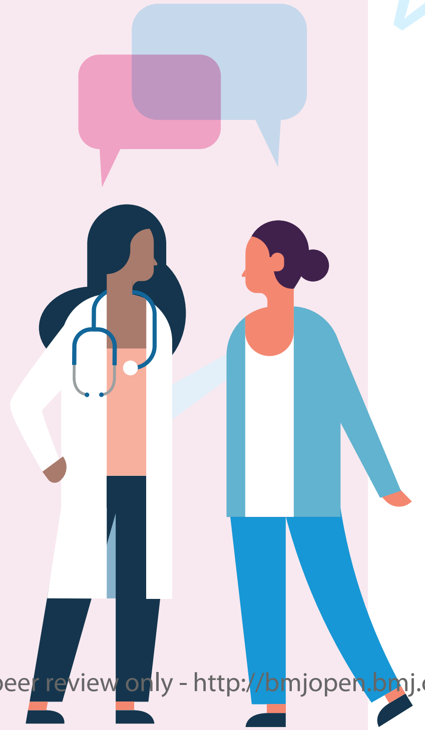

Criteria	Points* 1	2	3	4	5	6
I made it clear to the woman that a decision needs to be made on her participation in breast cancer screening	☐	☐	☐	☐	☐	☐
I asked the woman exactly how she wants to be involved in making the decision	☐	☐	☐	☐	☐	☐
I informed the woman that she has the option to participate or not in screening	☐	☐	☐	☐	☐	☐
I precisely explained the advantages and disadvantages of each option to the woman	☐	☐	☐	☐	☐	☐
I helped the woman understand all the information about benefits and adverse effects	☐	☐	☐	☐	☐	☐
I asked the woman which option she prefers	☐	☐	☐	☐	☐	☐
The woman and I thoroughly weighed all the options	☐	☐	☐	☐	☐	☐
The woman and I selected an option together	☐	☐	☐	☐	☐	☐
The woman and I have come to an agreement about how to proceed with her subsequent healthcare	☐	☐	☐	☐	☐	☐

* The scale ranges from: "Completely disagree" (1) to "Completely agree" (6)

From: De las Cuevas C, Perestelo-Perez L, Rivero-Santana A, Cebolla-Martí A, Scholl I, Härter M. Validation of the Spanish version of the 9-item Shared Decision-Making Questionnaire. Health Expect [Internet]. 2015;18(6):2143–53. Available from: http://www.ncbi.nlm.nih.gov/pubmed/24593044

For peer review only - http://bmjopen.bmj.com/site/about/guidelines.xhtml

NO OLVIDES QUE...

La mamografía no evita que tengas cáncer de mama. Además, no es un método perfecto; algunos tumores son muy difíciles de ver en una mamografía.

Puede ser que no tengas cáncer. Pero si lo tuvieras, el diagnóstico y tratamiento en una fase inicial del tumor puede suponer una mayor probabilidad de supervivencia.

Aunque te hayas hecho una mamografía recientemente, es importante que si notas algún cambio en el pecho vayas al médico.

La información presentada en este folleto se ha basado en artículos científicos y materiales desarrollados por el Programa de Cribado de Cáncer de Mama del National Health Service en Inglaterra, por la Colaboración Cochrane y por programas de cribado de Cataluña.

GOBIERNO DE ESPAÑA · MINISTERIO DE ECONOMÍA Y COMPETITIVIDAD · Instituto de Salud Carlos III

Unión Europea
Fondo Europeo de Desarrollo Regional
"Una manera de hacer Europa"

Estudio PI14/00113 Participación de las mujeres en las decisiones y estrategias de detección precoz del cáncer de mama. Cofinanciado por el Instituto de Salud Carlos III y fondos FEDER de la Unión Europea. Participan: Institut de Recerca Biomèdica de Lleida-Universitat de Lleida, Universitat Rovira i Virgili, Institut Català d'Oncologia, Hospital del Mar y Servicio Canario de Salud.

BENEFICIOS Y EFECTOS ADVERSOS A LARGO PLAZO DE LA DETECCIÓN PRECOZ DEL CÁNCER DE MAMA

Si un grupo de 200 mujeres entre 50 y 69 años se hacen mamografías de cribado cada 2 años, cuando cumplan 80 años...

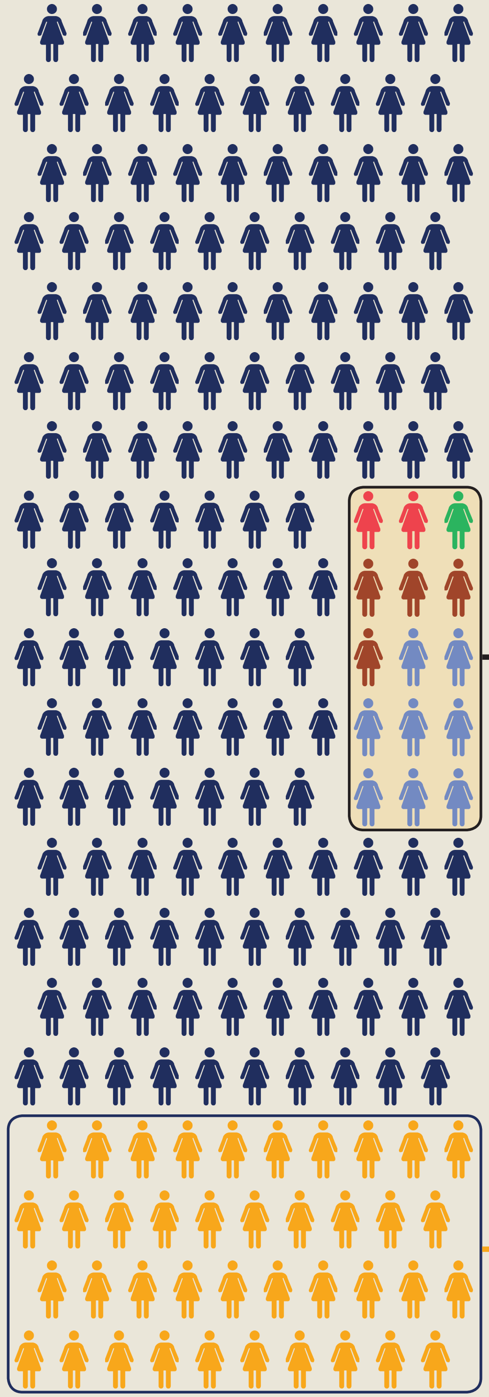

15 mujeres habrán sido diagnosticadas de cáncer de mama

BENEFICIOS DEL CRIBADO

1 sobrevivirá al cáncer de mama
Habría fallecido sin el cribado
Los tratamientos de algunas mujeres pueden ser menos agresivos

CON O SIN CRIBADO

4 habrán muerto de cáncer de mama a pesar de haber participado en el cribado

8 habrán sido diagnosticadas y sobrevivirán
Habrían sobrevivido aunque no se hubieran cribado

EFECTOS ADVERSOS DEL CRIBADO

2 serán diagnosticadas y tratadas de un cáncer de mama
Este cáncer nunca habría sido una amenaza para su vida

40 habrán necesitado pruebas adicionales
Posteriormente se descartará que hubieran tenido cáncer de mama (falsos positivos)

Por cada muerte evitada por el programa de cribado, 2 mujeres son diagnosticadas y tratadas de un cáncer que nunca hubiera puesto en riesgo su vida.

LA DETECCIÓN PRECOZ DEL CÁNCER DE MAMA

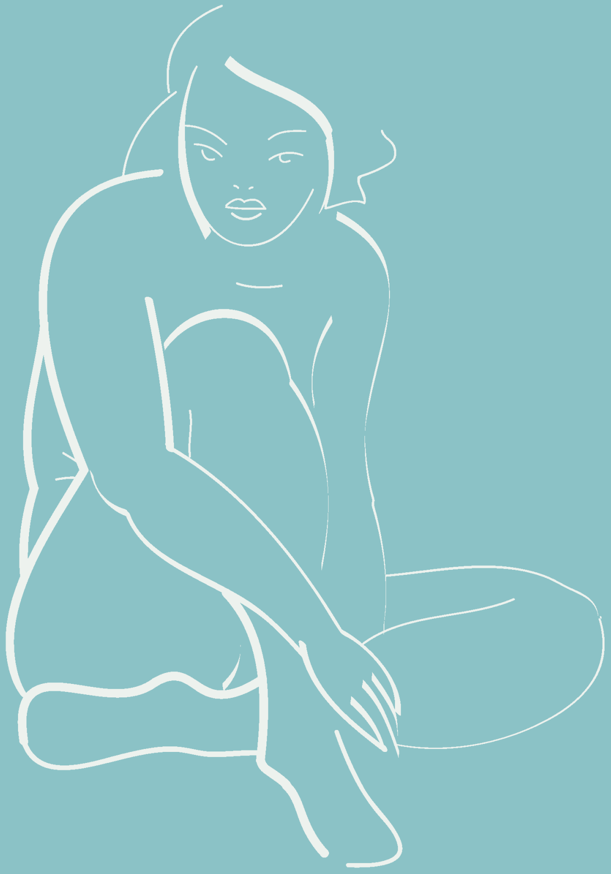

AYUDÁNDOTE A DECIDIR

PARTICIPAR O NO PARTICIPAR EN EL CRIBADO DEL CÁNCER DE MAMA: ESTA ES LA CUESTIÓN

Estudios científicos recientes han identificado efectos adversos, antes desconocidos, de la detección precoz de cáncer de mama mediante mamografía. Por esta razón, este folleto tiene como objetivo informar sobre los beneficios y efectos adversos de participar en la detección precoz de cáncer de mama.

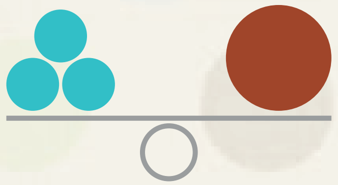

Este material informativo pretende ayudarte a sopesar pros y contras para que puedas tomar una decisión personal sobre si deseas participar o no en la detección precoz del cáncer de mama, en función de tus valores y preferencias.

¿QUÉ ES EL CÁNCER DE MAMA?

El cáncer de mama se desarrolla cuando algunas células empiezan a crecer de forma descontrolada, formando un tumor. A medida que el tumor crece las células malignas se pueden desplazar a otras partes del cuerpo y poner en peligro la vida de la persona afectada.

En Cataluña se diagnostican unos 4.000 casos nuevos de cáncer de mama al año. Las estadísticas nos dicen que 1 de cada 9 mujeres padecerá cáncer de mama a lo largo de su vida y que el 83% de las mujeres afectadas sobrevivirán a esta enfermedad.

¿QUÉ ES LA DETECCIÓN PRECOZ DEL CÁNCER DE MAMA?

La detección precoz del cáncer de mama, también denominada cribado, tiene por objetivo detectar un cáncer de mama en una etapa muy inicial, antes de que aparezcan síntomas. En su etapa inicial, el cáncer es más fácil de tratar y las oportunidades de sobrevivir son superiores.

El sistema sanitario público ofrece la posibilidad de participar en la detección precoz del cáncer de mama con el objetivo de reducir la mortalidad causada por este tumor. El programa de cribado se dirige a las mujeres entre 50 y 69 años y consiste en realizar una mamografía cada dos años.

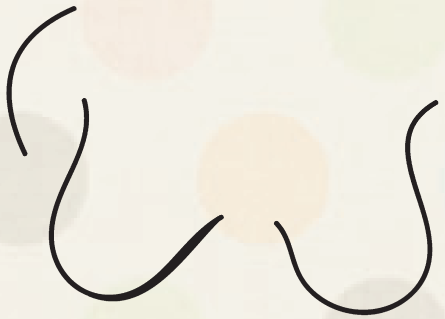

La mamografía es una radiografía de la mama. Es la prueba más eficaz para detectar el cáncer de mama en mujeres que no presentan síntomas. El riesgo de algún daño por la exposición a esta radiación es muy pequeño.

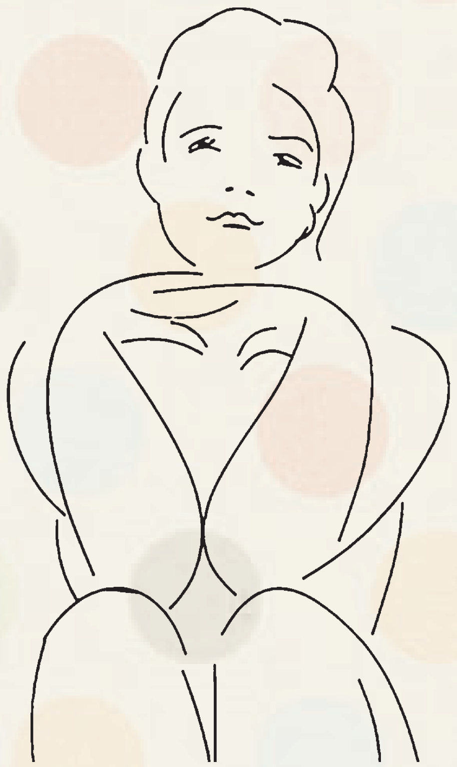

BENEFICIOS DEL CRIBADO

El cribado reduce el riesgo de morir por cáncer de mama

La detección precoz puede salvar la vida a algunas mujeres porque se diagnostican y tratan antes de lo que se habría hecho sin cribado.

De cada 200 mujeres que se realizan mamografías de cribado cada dos años, entre los 50 y los 69 años, 1 mujer se salva gracias a la detección precoz del tumor.

El cribado detecta el cáncer en estadios más iniciales

Un cáncer detectado en estadios iniciales no necesita tratamientos tan agresivos como cuando está más avanzado; estos tratamientos tienen menos efectos secundarios y la probabilidad de recuperación es más alta.

EFECTOS ADVERSOS DEL CRIBADO

Errores en el diagnóstico: falsos positivos y falsos negativos

Los falsos positivos se producen cuando los resultados de la mamografía hacen sospechar de un posible cáncer de mama que en realidad no existe. Esto conlleva exploraciones adicionales que no serían necesarias.

De cada 200 mujeres que se realizan mamografías de cribado cada dos años entre los 50 y los 69 años, 40 tendrán un resultado falso positivo.

La situación contraria, el falso negativo, es mucho menos frecuente y se puede producir cuando la mamografía no muestra ninguna señal de cáncer de mama, aunque la mujer lo padezca.

El cribado puede detectar tumores inofensivos

Algunos tipos de cáncer que se detectan mediante la mamografía de cribado crecen tan lentamente que nunca hubieran llegado a ser un problema de salud. Algunos, incluso, habrían desaparecido de forma espontánea, sin tratamiento.

Actualmente no se puede saber qué lesiones progresarían y cuáles no, y por tanto, se ofrece tratamiento a todas las mujeres diagnosticadas. Algunas mujeres pueden recibir tratamientos que tienen efectos secundarios importantes, sin necesitarlos. Esto se conoce como **sobrediagnóstico** y **sobretratamiento**.

De cada 200 mujeres que se realizan mamografías de cribado cada dos años, entre los 50 y los 69 años, 2 serán tratadas de cáncer sin necesidad.

Development of support material for health professionals who are implementing Shared Decision-Making in breast cancer screening: Validation using Delphi methodology

Revised Standards for Quality Improvement Reporting Excellence (SQUIRE 2.0)

Text Section and Item Name	Section or Item Description	Chek
**Title and Abstract**		
1. Title	Indicate that the manuscript concerns an initiative to improve healthcare (broadly defined to include the quality, safety, effectiveness, patient- centeredness, timeliness, cost, efficiency, and equity of healthcare)	X
2. Abstract	a. Provide adequate information to aid in searching and indexing<br>b. Summarize all key information from various sections of the text using the abstract format of the intended publication or a structured summary such as: background, local problem, methods, interventions, results, conclusions	X
**Introduction**	**Why did you start?**	
3. Problem Description	Nature and significance of the local problem	X
4. Available knowledge	Summary of what is currently known about the problem, including relevant previous studies	X
5. Rationale	Informal or formal frameworks, models, concepts, and/or theories used to explain the problem, any reasons or assumptions that were used to develop the intervention(s), and reasons why the intervention(s) was expected to work	X
6. Specific aims	Purpose of the project and of this report	X
**Methods**	**What did you do?**	

For peer review only

Development of support material for health professionals who are implementing Shared Decision-Making in breast cancer screening: Validation using Delphi methodology

7. Context	Contextual elements considered important at the outset of introducing the intervention(s)	**X**
8. Intervention(s)	a. Description of the intervention(s) in sufficient detail that others could reproduce it<br>b. Specifics of the team involved in the work	**X**
9. Study of the Intervention(s)	a. Approach chosen for assessing the impact of the intervention(s)<br>b. Approach used to establish whether the observed outcomes were due to the intervention(s)	**X**
10. Measures	a. Measures chosen for studying processes and outcomes of the intervention(s), including rationale for choosing them, their operational definitions, and their validity and reliability<br>b. Description of the approach to the ongoing assessment of contextual elements that contributed to the success, failure, efficiency, and cost<br>c. Methods employed for assessing completeness and accuracy of data.	**X**
11. Analysis	a. Qualitative and quantitative methods used to draw inferences from the data<br>b. Methods for understanding variation within the data, including the effects of time as a variable	**X**
12. Ethical Considerations	Ethical aspects of implementing and studying the intervention(s) and how they were addressed, including, but not limited to, formal ethics review and potential conflict(s) of interest	**X**
**Results**	**What did you find?**	
13. Results	a. Initial steps of the intervention(s) and their evolution over time (e.g., time-line diagram, flow chart, or table), including modifications made to the intervention during the project<br>b. Details of the process measures and outcome<br>c. Contextual elements that interacted with the intervention(s)<br>d. Observed associations between outcomes, interventions, and relevant contextual elements<br>e. Unintended consequences such as unexpected benefits, problems, failures, or costs associated with the intervention(s).<br>f. Details about missing data	X

Development of support material for health professionals who are implementing Shared Decision-Making in breast cancer screening: Validation using Delphi methodology

Discussion	What does it mean?	
14. Summary	a. Key findings, including relevance to the rationale and specific aims<br>b. Particular strengths of the project	X
15. Interpretation	a. Nature of the association between the intervention(s) and the outcomes<br>b. Comparison of results with findings from other publications<br>c. Impact of the project on people and systems	X
16. Limitations	a. Limits to the generalizability of the work<br>b. Factors that might have limited internal validity such as confounding, bias, or imprecision in the design, methods, measurement, or analysis c. Efforts made to minimize and adjust for limitations.	X
17. Conclusions	a. Usefulness of the work<br>b. Sustainability<br>c. Potential for spread to other contexts<br>d. Implications for practice and for further study in the field<br>e. Suggested next steps	X
	**Other information**	
18. Funding	Sources of funding that supported this work. Role, if any, of the funding organization in the design, implementation, interpretation, and reporting	X

For peer review only

BMJ Open

Title: Development of support material for health professionals who are implementing Shared Decision-Making in breast cancer screening: Validation using Delphi technique

Journal:	*BMJ Open*
Manuscript ID	bmjopen-2021-052566.R1
Article Type:	Original research
Date Submitted by the Author:	21-Oct-2021
Complete List of Authors:	Hernández Leal, María José; Universitat Rovira i Virgili, Economic<br>Codern-Bové, Núria; Universitat Autònoma de Barcelona<br>Pérez-Lacasta, María José; University Rovira i Virgili, Economica<br>Cardona, Angels; Area Q , Evaluation and Research in the Field of Social Sciences and Health.<br>Vidal, Carmen; Catalan Institute of Oncology, Cancer Prevention and Control Programme<br>Carles-Lavila, Misericòrdia; Universitat Rovira i Virgili, Department of Economics
**Primary Subject Heading**:	Medical education and training
Secondary Subject Heading:	Patient-centred medicine, Qualitative research, General practice / Family practice, Medical education and training, Oncology
Keywords:	MEDICAL EDUCATION & TRAINING, Breast tumours < ONCOLOGY, PREVENTIVE MEDICINE, PRIMARY CARE, PUBLIC HEALTH, QUALITATIVE RESEARCH

SCHOLARONE™
Manuscripts

I, the Submitting Author has the right to grant and does grant on behalf of all authors of the Work (as defined in the below author licence), an exclusive licence and/or a non-exclusive licence for contributions from authors who are: i) UK Crown employees; ii) where BMJ has agreed a CC-BY licence shall apply, and/or iii) in accordance with the terms applicable for US Federal Government officers or employees acting as part of their official duties; on a worldwide, perpetual, irrevocable, royalty-free basis to BMJ Publishing Group Ltd ("BMJ") its licensees and where the relevant Journal is co-owned by BMJ to the co-owners of the Journal, to publish the Work in this journal and any other BMJ products and to exploit all rights, as set out in our licence.

The Submitting Author accepts and understands that any supply made under these terms is made by BMJ to the Submitting Author unless you are acting as an employee on behalf of your employer or a postgraduate student of an affiliated institution which is paying any applicable article publishing charge ("APC") for Open Access articles. Where the Submitting Author wishes to make the Work available on an Open Access basis (and intends to pay the relevant APC), the terms of reuse of such Open Access shall be governed by a Creative Commons licence – details of these licences and which Creative Commons licence will apply to this Work are set out in our licence referred to above.

Other than as permitted in any relevant BMJ Author's Self Archiving Policies, I confirm this Work has not been accepted for publication elsewhere, is not being considered for publication elsewhere and does not duplicate material already published. I confirm all authors consent to publication of this Work and authorise the granting of this licence.

For peer review only

Original Research Article

Title: Development of support material for health professionals who are implementing Shared Decision-Making in breast cancer screening: Validation using Delphi technique

María José Hernández-Leal[1,a,b,c], Núria Codern-Bové[2,d], María José Pérez-Lacasta[3,a,c], Angels Cardona[4,e], Carmen Vidal-Lancis[5,f], Misericòrdia Carles-Lavila[6,a,b,c] on behalf of the ProShare Group

1. PhD student, MSc and BSc Nursing. Email: mariajose.hernandez@urv.cat
2. PhD. Email: nuriacodern@euit.fdsll.cat
3. PhD. Email: mariajose.perez@urv.cat
4. MSc. Email: angels.cardona@areaq.net
5. MPH. Email: cvidal@iconcologia.net
6. PhD. Email: misericordia.carles@urv.cat

a. Department of Economics, Universitat Rovira i Virgili.
b. Research Centre on Economics and Sustainability (ECO-SOS).
c. Research Group on Statistics, Economic Evaluation and Health (GRAEES).
d. Terrassa University School of Nursing and Occupational Therapy (EUIT), Universitat Autònoma de Barcelona, Terrassa, Spain.
e. Area Q: Evaluation and Research in the Field of Social Sciences and Health.
f. Cancer Prevention and Control Program, Catalan Institute of Oncology-IDIBELL, L'Hospitalet de Llobregat, Spain,

ProShare group: Misericòrdia Carles-Lavila, Núria Codern-Bové, María José Hernández-Leal, María José Pérez, Roger Pla, Vanesa Ramos, Carmen Vidal.

Corresponding author:
Misericòrdia Carles-Lavila, Department of Economics, Universitat Rovira i Virgili, Avenida Universitat 1, 43204, Reus, Spain. Email: misericordia.carles@urv.cat

Word count: 5090

For peer review only

ABSTRACT

Background: Literature not reported support material about Shared Decision-Making (SDM) to breast cancer screening for Health professional in Spain. The researcher created a handbook and guide for this topic using an adaption of the Three-talk model by Elwyn, et al.

Objective: The experts to determine the usefulness and relevance of the contents and design provided in a handbook and a guide to support the application of Shared Decision-Making to breast cancer screening intended to healthcare professionals.

Design: The Delphi technique is a qualitative study; it was discussing with 20 experts about the content and design of the handbook and the guide. Delphi was online mode between July and October 2020 and researchers used Google forms in three rounds with open and close questions. The criterion established for consensus was a coefficient of concordance (Cc) above 75, for questions using a Likert scale of 1 to 6 - in which 1 was 'completely disagree' and 6 was 'completely agree'- with a cut-off point equal to or greater than 4.

Results: Participants considered the Three-talk model suitable for the screening context. The handbook's sections and level of detail were considered satisfactory (Cc=90). The summary provided by the clinical practice guide was considered necessary (Cc=75), as was the self-assessment tool for professionals (Cc=85). Content was added: addressing the limitations of the SDM model; extending the number of example dialogues for health professionals to three; providing supplementary resources on using Patients Decisions Aids (PtDAs) and adding references on communication skills.

Conclusions and applications: The first handbook and clinical practice guide providing unique SDM support material for health professionals have been developed. The handbook and guide are useful and innovative in support material for health professionals, but to facilitate it implementation is requisite training strategies for SDM and a plan for piloting for use of material.

Keywords: Shared Decision-Making; breast cancer screening; health professionals.

STRENGTHS AND LIMITATIONS OF THIS STUDY

- Development of a handbook and a clinical practice guide to Shared Decision-Making for breast cancer screening.
- Adaptation of the Three-talk model to breast cancer screening.
- Participation of professionals in validating the design of the support materials.
- Facilitating the application of a person-centred model to the screening context.

For peer review only - http://bmjopen.bmj.com/site/about/guidelines.xhtml

BACKGROUND

Shared Decision-Making (SDM) is recommended for uncertainty context -among others- where is necessary discus about risks and benefits in health topics (1)-. SDM is a relationship model between doctor-patient, and both collaborate to deliberate over the best choice on the basis of scientific evidence, and women's preferences and values (2,3). Thus, the SDM invites you to change the paternalistic health model for a more participatory one, seeks a greater involvement of patients in their health and not the aim a greater adherence to treatments, procedures or medicines, although it has also been associated as a result of its application (4).

In Spain there is no evidence that explicitly recommends in which type of women SDM should be performed, however its use through PtDAs has been extended to all women who must decide to have mammography because they are integrated into breast cancer screening programmes in each Autonomous Community. However, PtDAs are not commonly used in clinical appointments (5).

The breast cancer screening (BC) programme currently falls under the Oncology Master Plan (*Plan Director de Oncología*) in Catalonia (6). However, while there are strategies for incorporating women's values and preferences into the decision on whether or not to have the examination, there is no associated framework on how to put them into practice (7). The current situation in Catalonia is the Breast Cancer Detection Programme (*Programa de Detección del Cáncer de Mama*) sends -each two years- women between 50 and 69 years a letter informing them of the time and date at which they should attend their local centre to have a mammogram (8). The programme achieves a high level of coverage, but it fails to incorporate an opportunity for the woman and the professional to exchange information and enter into dialogue on the decision. In order to promote the women's participation, several research teams have developed projects that involve women in making the decision on screening. In 2017, Toledo-Chavari and colleagues created a PtDAs (5) (Annex 1), consisting of a trifold leaflet that provides balanced information on the benefits and adverse effects, for the professionals and women to use during the clinical appointment. However, based on the barriers and enabling factors cited in the literature (9-11), the researchers decided that it was not enough to use the PtDAs alone, concluding that SDM training material aimed at health professionals was also needed. A training material are the manuals, as these are a useful tool to transmit knowledge and provide quick and simple information on how to operationalize new practices, turning novices on a theme into an advanced users in order to using it as they use it (12). Considering that SDM is not a common practice, a manual could to some extent fill knowledge gaps on this model.

The ProShare Study, our research team has therefore developed a handbook-manual- (Annex 2) and guide (13) (Annex3) aimed at health professionals who have direct relationships with women. These documents should be used as reference material by health professionals when facing the decision with women on -or not- to perform mammography, taking into consideration key elements are providing the patient with information and education, interpersonal communication between doctor and patient, finally a decision (14). To facilitate the implementation of SDM the model was used as a reference the Three-talk model, adapting sus three steps to BC screening: 1) Team talk; 2) Option talk and exploring preferences; 3) Decision talk (15). A self-assessment of the SDM was included in the manual, which should be applied at the end of the appointment so professionals can identify strengths and weaknesses in the implementation of the SDM. Finally, the guide provides a summary of the handbook to be used in the same appointment as a reminder of the three steps.

For peer review only - http://bmjopen.bmj.com/site/about/guidelines.xhtml

The objective of this study is the experts to determine the usefulness and relevance of the contents and design provided in a handbook -manual- and a guide to support the application of Shared Decision-Making to breast cancer screening intended to healthcare professionals

METHODS

Delphi Technique

The Delphi technique has the main objective of reaching consensus among experts on specific topics. For this reason, it was decided to use it since when you want developing training competencies, tools to support clinical practice or a response to a professional issue, seeking the opinion of experts is a common approach (16) in this case experts are required for the development of a manual and guide because there are no materials of this nature on the subject of SDM in breast cancer screening, and therefore it is an area without previous references. Another feature of a Delphi is that participants undergo a series of virtual question rounds, which are formulated with elements that were not agreed upon in the previous round (17-18). This process is repeated continuously until one of the completion criteria is met. (19). A further requirement for the formulation of the Delphi is that the responses of all experts be shared in each round, thus allowing experts to reassess their responses in the light of the views of other experts. Finally, all the rounds must be carried out anonymously and thus ensure that they do not influence others just because of the reputation that one of the experts in the topic could have. One of the limitation Delphi techniques is that this provides expert opinion, but could also be considered other complementary techniques to determine a final position on the subject of study (16-18). The experts participating in a virtual way can overcome barriers related to circumstances economic, geographic or time-related constraints (17,18). The experts, according to the literature, can be grouped into two broad categories: *Subjects (Su)* – people who would use the instrument in their profession; and *Specialists (Sp)* – people who have knowledge about the subject due to their academic and/or professional experience (17,18).

Participants

The handbook and clinical practice guide, entitled 'The participation of health professionals in Shared Decision-Making in breast cancer screening' (*La participación de los profesionales de la salud en la Toma de Decisiones Compartida en el cribado de cáncer de mama*) (Appendix 2) (13), were developed by the ProShare research team. The first version was produced with the participation of three researchers with experience in Shared Decision-Making and BC screening, who acted as external reviewers, and two health professionals, who produced the plan for piloting the questionnaire online (Google form).

The inclusion criteria for participants were as follow*s:*

- *Subjects*: a) health professionals, preferably from primary care services, who provide direct care to women through breast cancer prevention activities, and b) who have at least five years' experience (20) in the Spanish health system.
- *Specialists*: a) international-level researchers whose research career has focused on the Shared Decision-Making model, and b) who are proficient in Spanish (given that the handbook has been produced in this language). Preference was given to individuals who had developed educational support material for professionals (20).

To determine the size of the sample, literature was consulted, which mentions that large numbers (more than 50 people) could imply an impediment in so many people reaching

For peer review only - http://bmjopen.bmj.com/site/about/guidelines.xhtml

agreement in a limited time. Moreover, it depends on the heterogeneity of the experts, if they are of various subjects and international enrich the opinions formulated (18). Therefore, a limit between 7 and 30 (18) was decided, with the most common being a total of 15 to 20 experts (18).

Procedure and Data collection

The researcher use two sampling strategies were used to recruit participants: convenience sampling for the specialists and snowball sampling for the health professionals. For the specialist, the researcher looking for published articles about SDM and contact the authors by e-mail (MJH, MC, MJP). For the health professionals, the researchers sent an e-mail to invitations (NC, AC), and they could be resent to other collages. Finally, researchers (NC-AC) sent invitations by email to 43 potentials experts for participate in a Delphi, 30 whom accepted. The aim was determinate the usefulness of the topics, relevance of the content and document design of the material for the SDM on BC screening. The Delphi was making on Google form between July and October 2020.

For round 1, open and closed questions were considered with topics of relevance to the research objective "*The sections of the handbook are effective for understanding the application of SDM to breast cancer screening* " or "*Do you think that a guide concisely summarizing the SDM steps is necessary?"*. Participants should mark the degree of agreement to the questions using a Likert scale of 1 to 6, in which 1 was 'completely disagree' and 6 was 'completely agree'. Later, when all experts finish the questionary research (MJH-MC-MJP) sent The experts received a report with the answers so that the participants could consider the views of the other participants -remaining anonymous-, especially in those questions where no agreement was reached in the group (Cc = 75), where these questions -disagreement- were raised again in the following rounds and so on until reaching the necessary agreement in the most transversal aspects. This was finally achieved in round 3.

Data analysis

The researchers (MJH-MC-MJP-NC-AC) analysed participants' responses at the end of each round, considering responses whose score on the Likert scale was 4 or above to be positive. Agreement was determined to be reached when the coefficient of concordance (Cc) was greater than 75 (21) The Coefficient of Concordance (Cc) >75 was used. For calculate consider the next formula:

$$Cc = \left(1 - \frac{Vn}{Vt}\right) x100$$

Vn = Number of negative votes (score of less than 4); Vt = Total number of votes (n=6) (21)

For R3, the criteria established by Martínez (2003) were considered to bring the Delphi close (19).

This research was approved by the Medicinal Product Research Ethics Committee (CEIm) of the Institut d'Investigació Sanitària Pere Virgili (Pere Virgili Health Research Institute). Informed consent was secured, which stated that participants accepted the conditions of participation upon agreeing to respond to the questionnaire. These conditions specified that responses were confidential and would only be used for the purposes of this research.

For peer review only - http://bmjopen.bmj.com/site/about/guidelines.xhtml

RESULTS

Of the 30 professionals who initially agreed to participate, 20 (66.6%) went on to respond in the first round (R1), 16 (53.3%) in the second (R2) and 17 (56.6%) in the third (R3) (Figure 1). In R1, the mean age of the experts was 46.6 years (SD 10.25), 75% were female, 65% were doctors, 70% worked in the public sector and they had an average of 19 years' (SD 9.69) experience (Table 1).

Figure 1: Flow diagram of participation in each round
Table 1: Characteristics of the participants

Round 1

R1 was designed to achieve two objectives: determinate the utility and clarify of content and the design of the support material. For this purpose, participants were asked 33 Likert-scale questions, 1 multiple-choice question and 6 open questions on the handbook and were given 2 Likert-scale questions and 4 open questions on the clinical practice guide (Table 2).

Table 2: R1 responses

A Cc greater than 75 was recorded for 32 of the Likert-scale questions and the minimum Cc was not reached for only 3 of them, In other words, no agreement was reached. These questions concluded that Figure 5, entitled 'Flow diagram of the Early Detection of Breast Cancer programme', was clear (Cc=60) and useful (Cc=70) (Figure 2). The same applied to the question that determined Figure 6 – Team talk (page 34) – to be clear (Cc=75). These questions were incorporated into R2.

Figure 2: Flow diagram of the Early Detection of Breast Cancer Programme

In the multiple-choice question, participants were asked which section of the handbook should be edited: 10 responded 'none'; 5 chose the section entitled 'Which skills or competencies do health professionals need?'; 3 selected the 'Screening programme' section, and 2 chose the 'Introduction' (Figure 3).

Figure 3: Changes made to the index

In their open responses, most participants considered the initiative to be positive and thought that it would enable health professionals to access information on SDM through use of the Three-talk model in BC screening (Box 1). However, one of the participants suggested using the Agency for Healthcare Research and Quality model.

Box 1: Response to the question: Are the steps based on "Three-talk" suitable for the application of SDM in breast cancer screening? Please explain briefly

For peer review only - http://bmjopen.bmj.com/site/about/guidelines.xhtml

P3 (R1): *Yes, it shows how the health professional can implement SDM in a three-step process in a brief, practical and easy-to-read way. It describes the characteristics that differentiate each step, and specific examples of implementation in breast cancer screening.*

The participants also provided some suggestions to modify the handbook. The most frequently cited concerned the length of the handbook, recommending reducing the content (Box 2) and incorporating example dialogues, communication skills (Box 3) and instructions for using the PtDAs. The comments were incorporated into the questions in R2.

Box 2: Response to the question: How would you improve the elements selected in the previous question?

P7: *I think that the handbook is very long, which may reduce motivation to read it.*
P6: *Very long and it doesn't show how to use the tool.*

Box 3: Response to the question: What other content would you include in the clinical practice guide?

P3: *Provide more information or example dialogues on how to use communication skills. This last [point] if the health professionals don't have a grounding or training in active listening, motivational interviewing, empathy, reflection, etc.*
P10: *I'd go into greater depth on relationship-building skills and give a few links to where they can find exercises to train themselves [in this].*

Finally, in response to the question on whether the dialogues in each step represent their objective, most participants agreed ('Team talk' step, n=10; 'option talk' step, n=7; 'Decision talk' step, n=12) and made suggestions on the wording of the dialogues. Suggestions were also made to adapt the name of the original *the Three-talk* steps to one that was more representative of the screening context. All the suggestions were incorporated into R2 to be approved or rejected by the other participants.

Only one of the questions evaluating the clinical practice guide did not reach the minimum Cc established: 'Do you consider a guide that concisely summarises the SDM steps to be necessary?' (*¿Cree necesaria una guía que resuma de forma rápida las fases de la TDC?*) (Cc=75). This question was incorporated into R2. In the open questions, the participants suggested changing the wording of the step 1 dialogues (n=3) and incorporating a review of communicative skills (Box 4); the same applied to step 2, but participants added a comment about using relative risks instead of absolute ones (n=1) (Box 5).

Box 4: Response to the question: What elements would you change in step 1: 'Team talk'?

P3: *I'd include a few reviews, such as [on] active listening and deliberation. Perhaps using a phrase like 'Remember to pay close attention and give assertive responses (active listening), and to think the options through carefully for the decision (deliberation)'.*

For peer review only - http://bmjopen.bmj.com/site/about/guidelines.xhtml

Box 5: Response to the question: What elements would you change in step 2: 'Option talk?

P15: *Change relative risks to absolute risks.*

They also proposed: eliminating the definition of SDM for step 3 in the guide (n=4), incorporating a brief clarification noting that women may also consult other people for support with making the decision (n=3) and mentioning the possibility of reversing the decision (n=4) (Box 6). Between 6 and 8 people stated that they would not makes any change to steps 1, 2 or 3.

Box 6: Response to the question: What elements would you change in step 3: 'Decision talk'?

P11: *I'd add the possibility of reversing the decision; I'd take out the explanation about SDM.*

Finally, in the last question – 'What other content would you include in the clinical practice guide?' – the participants reiterated the need to include a review of communication skills (n=3) and one of them proposed changing the self-assessment to use either the ASQ3 or the CollaboRATE instrument.

Round 2

R2 was structured around the open-question responses and included the elements about which agreement had not been reached in the previous round. Thirteen Likert-scale questions, 5 multiple-choice question and 6 open questions were produced on the handbook. For the clinical practice guide, 2 Likert-scale questions and 5 open questions were included (Table 3).

Table 3: R2 responses

Of the 13 Likert-scale questions, only 3 reached a score of Cc>75. These underlined the need to: reduce the length of the handbook (Cc=81.3), create a clinical practice guide to accompany the handbook (Cc=81.3), and mention the possibility of reversing the decision in the follow-up plan (Cc=87.6).

The closed questions included the following – 'Which elements of the handbook would you shorten?' (*¿Qué elementos reducirían del Manual?*) – to which the two most significant answers were the 'Introduction' (50%) and 'None' (31.3%). Following the comments made in the previous round, alternative formulations of the example phrases for the dialogues in each *the Three-talk* steps were given, as well as a change of name for step 2 to 'Option talk and exploring preferences' (*Plantear opciones y explorar preferencias*), on which consensus was reached (81,3%).

In their responses to the open questions, those who considered the proposed dialogues not to be representative of the steps had the opportunity to suggest how they could be reworded. Finally, participants were able to include their final comments on the handbook and the guide (Figure 4).

For peer review only - http://bmjopen.bmj.com/site/about/guidelines.xhtml

Figure 4: Changes made to the guide

Most had no further suggestions for either document, but some participants included comments about shortening the handbook (Box 7) and including this material in clinical practice guides, in order to improve implementation (Box 8).

Box 7: Response to 'Provide your final comments on the handbook'

P10: *None, the idea of including appendices on communication skills for the health professional, and on the screening tests for the women, seems like an excellent idea to me, to avoid making the handbook longer but offer additional tools for those health workers and women who would like more information.*

Box 8: Response to 'Provide your final comments on the guide'

P10: *Clinical practice guidelines on the preventive approach to breast cancer that includes these points on shared decision-making would be very useful to support implementation. In any case, I don't think that it is a prerequisite to be able to produce the handbook that you are working on. This handbook could be incorporated into future Clinical Practice Guidelines (CPG).*

Round 3
R3 was structured around the 10 elements about which no agreement was reached in R2. Six questions with closed, dichotomous answers were posed in the section evaluating the handbook, and 1 in the section evaluating the clinical practice guide, in addition to an open question. Of these, only those proposing an improvement to the organisation of the clinical practice guide, a change of colours and a review of cross-cutting communication skills in SDM reached a Cc of over 75% (Table 4).

Table 4: R3 responses

Given that agreement was not reached on the Flow diagram for the Early Detection of Breast Cancer Programme, this figure was removed from the handbook, in light of the fact that it only applies to the region of Catalonia. The other elements about which no agreement was reached were: the need to incorporate more examples of professional dialogues (64.7%); incorporating information about joint responsibility for the decision (41.2%); adding information on the limitations of the SDM model (58.8%), as well as adding supplementary resources on using the DST (52.9%) and on communication skills and competencies (58.8%). The researchers believed that the additional content would not entail substantial changes to the handbook and would provide more information to professionals who are not familiar with the model, so all these elements were incorporated into the handbook.

The texts included were developed in line with the proposals submitted by the participants in previous rounds. For example, the following elements were highlighted in the professional dialogues: the possibility of reversing the decision, needing more time, and accessing support from a third person to make the decision (Figure 5).

Figure 5: Example of dialogues for the professionals to "Team talk" step

For peer review only - http://bmjopen.bmj.com/site/about/guidelines.xhtml

The Delphi was brought to close in R3, taking into account the criteria cited by Martínez regarding the elements about which agreement was not reached (27): a) the limited number of items for which Cc>75 was not achieved (6 of the 61 Likert-scale and closed questions); b) limited resources and time; c) the possibility that participants would abandon the study in a subsequent round, which would affect the external validity of the study. The latter two criteria applied in the context of the COVID-19 pandemic, given that half of the participants are health professionals who work in health centres.

DISCUSSION

The literature mentions certain barriers to applying SDM in BC screening, including limited time in clinical appointments and health professionals' lack of training in providing more participatory care (19). This was the motivation for producing the first handbook and clinical practice guide on this subject, aimed at supporting health professionals by providing them with the essential elements for implementing SDM with women in a BC screening context.

The most relevant results included validation of the support materials using Delphi techniquel, taking into account the opinion of experts to reach agreements on editing the design and content, as well as their recommendation to incorporate these materials into the clinical practice guide. This validation included the possibility of adapting a generic model –*Three Talk* – to one designed specifically for the BC screening context.

Of the 43 participants who were invited to respond to the Delphi questionnaires, more than half expressed interest in the topic of the research and collaborating in it. However, only 20 of these went on to participate in the study. This may be related to the timetabling of the questionnaires, which coincided with the end of the first wave of the COVID-19 pandemic and the resurgence of cases at the beginning of the second wave. In spite of this, the professionals who decided to participate at the beginning of the process fulfilled their commitment, illustrated by the fact that participant numbers only decreased by three between rounds, these having been lost from the Subjects category (n=3).

Discussion between the participants

It was easy to reach an agreement on the main content elements in the first round. Regarding the structure and development of SDM using the *Three-talk* model (15), which was considered suitable for BC screening, one of the participants initially suggested using the model created by the Agency for Healthcare Research and Quality (22). However, this alternative model contains five steps and the model proposed by the authors, which involves fewer steps, met all the requirements of SDM. Regarding the set of nine figures in the handbook, only one was eliminated, and the wording of three was edited.

The participants also easily came to an agreement that the initial version of the handbook was very long, at 56 pages. The length was due to the fact that it will be published in a pocket edition, which is equivalent to 23 pages in a larger textbook edition. The researchers decided to maintain the smaller format because it is more transportable, although they cut down the content elements agreed by the participants.

For six of the items, it was not possible to reach an agreement. While agreement should ideally be reached for all items, when a new round will not provide any more information or is unlikely to achieve a better result, the rounds of questions may be brought to a close despite a small

For peer review only - http://bmjopen.bmj.com/site/about/guidelines.xhtml

number of disagreements remaining (19). The change in the formulation of the responses between R2 (Likert scale) and R3 (dichotomous) meant that participants had to opt for one of the options rather than rating their level of agreement with the statements, which undoubtedly made it more difficult to arrive at an agreement.

Certain responses to the open questions were analysed in depth by the researchers. One of the participants in R1 suggested that the professional self-assessment method could be changed from SDM-Q-doc (23) to Ask 3Q (24) or CollaboRATE (25). However, Ask 3Q is a methodology for applying SDM, making it an equivalent to the Three-talk model. Given that the Three-talk model received a positive evaluation from the participants, the change was not made. The other tool, CollaboRATE, is designed for the patient to evaluate the professional, which was not the purpose of this questionnaire (26). Our objective was for the professional to be able to evaluate how he or she performs SDM, resulting in self-guided learning of this methodology. The researchers therefore kept the original version, SDM-Q-doc, and adapted it for screening.

The decision on the flow diagram was affected by whether participants came from the region of Catalonia (of those living in Catalonia, 5/6 wanted to keep it, albeit improving its resolution; in contrast, the specialists originating outside Spain (7/11) opted to remove it). Given that the objective of the handbook is to be used in other territories, the research group decided to eliminate the flow diagram.

The example dialogues suggesting how professionals should conduct SDM at each point in the process were widely accepted as a fundamental part of the handbook, although no consensus was reached on whether to include more example dialogues for each steps (Su=4/6; Sp=7/11). While Cc>75 was not reached, a larger proportion of both groups advocated providing more examples. This may be directly related to the fact that both groups believed that SDM training for health professionals is still incomplete. Some of these participants therefore called for the handbook to provide more support, giving professionals greater confidence in implementation through use of the aforementioned dialogues. The same conclusion can be made regarding the decision to include more bibliographic references on communication skills and relationship-building competencies (Su=3/6; Sp=7/11) and on including information about PtDas (Su=5/6; Sp=4/11). In the latter case, the results differed between the two groups: most of the Subject participants wanted to add information on these tools, perhaps highlighting their lack of knowledge about them or training in their use, while the Specialists did not consider their inclusion to be as relevant, due to their familiarity with the tools.

How to improve the application of SDM to screening

While 83% of the health professionals expressed a high level of interest in promoting Shared Decision-Making during the clinical encounter (26), they recognised their lack of training in the SDM model as one of the most significant barriers to its implementation in the screening context (11).

A review of the training that health professionals receive confirmed our belief that there is a lack of strategies able to familiarise health professionals with this model. In Spain, the topic has been introduced into medicine and health-related degree programmes (27-30). However, it is not framed precisely within a SDM model, but is closer to communication or clinical communication skills, which have been used interchangeably as equivalents to the model. The level of detail and the strategies used in this training are also unknown. Most training in SDM

For peer review only - http://bmjopen.bmj.com/site/about/guidelines.xhtml

is acquired in postgraduate-level study aimed at doctors and nurses (31), whereas particular attention should also be paid to health workers in primary care (including support and technical staff, as well as clinicians), who provide person-centred healthcare in a holistic manner (32).

Experts in SDM have argued that it is necessary to prioritise adapting curricula to consolidate this training, by emphasising education in communication skills and the accreditation of these competencies (33), within the framework of a horizontal care model. Additionally, experts highlight the need to create partnerships between universities and interdisciplinary research groups to develop this material (33).

Experts also recommend training methodology based on practical activities such as role plays, as well as working in small teams of six people, training of over a day in length, and providing constructive feedback on students' capacity to express empathy, give assertive responses, engage in active listening, and other skills (34). This handbook and clinical practice guide therefore include dialogues and specific examples of how to apply them. This will serve as reference material supporting an initial grounding in SDM for professionals who have not received formal training in this subject, and as supplementary material for those who have, enabling them to apply the skills and competencies acquired in the specific context of BC screening.

The final structure of our document responds to the need described in the preceding paragraph and highlighted by the participants in the study.

Given the change of paradigm that SDM entails, all measures that help familiarise professionals with SDM are important. For example, adding a section into Clinical Practice Guidelines (CPG) on how to include the patient in decision-making, thereby coordinating evidence-based practice with SDM (35), may be useful. Patients may even participate to some degree in its development, as is current practice in such organisations as the National Institute for Health and Clinical Excellence (NICE) and the Scottish Intercollegiate Guidelines Network (36). In this sense, our proposed handbook and clinical practice guide, as well as the PtDas whose quality has already been evaluated and certified by international organisations such as The Ottawa Hospital (37), may be considered complementary materials.

LIMITATIONS

The main limitation of the study was participant recruitment, which is a typical constraint. It was a particular problem in this case, given that the empirical work coincided with the successive waves of the COVID-19 pandemic, hindering the active participation of some professionals who had initially agreed to participate in the study. Despite this, there were fewer withdrawals from Round 2 onwards than might have been expected in the circumstances.

The change in the formulation of the R2 (Likert scale) and R3 (dichotomous) responses may have made it more difficult to reach the established minimum Cc for agreement. Nevertheless, with reference to Martínez (2003) (19), the research team determined that one more round would not have provided any added value to the results, for the reasons described in the preceding sections. Nevertheless, the decision made regarding those elements about which no agreement had been reached did not significantly affect the participants' opinions regarding the basic concepts on which the initial questionnaire was based.

For peer review only - http://bmjopen.bmj.com/site/about/guidelines.xhtml

Finally, it should be noted that a systematic literature review (2018) showed that more research is still needed to determine the real impact that training interventions have on health professionals regarding SDM, given that the level of certainty of the studies was low or very low. In this research, professionals who had received standard training were compared with those who had been trained in SDM; from the 15 studies, it was concluded that the results for patients' satisfaction, knowledge, decision-related conflict, regret, level of health and quality of life differed little or not at all (31). In spite of this, the demand for information and training expressed by this study's participants leads us to believe that this first handbook aimed at health professionals for implementation in a BC screening context will help bring clarity to the healthcare model centred on patients and their needs and preferences. However, we have also noted the need to expand training in SDM and develop empirical strategies to facilitate its implementation.

CONCLUSION

A horizontal relationship between patients and health professionals enables person-centred care to be delivered, in which that person is considered a protagonist in the decisions made about his or her health. This has been recognised by several governmental organisations and incorporated into discourse and strategies. However, the practical application of this model is an area in which progress has yet to be made. The handbook and clinical practice guide therefore aim to familiarise professionals with the model, helping them to engage women in the decision of whether have BC screening or not. The results obtained enable us to conclude that, in order to be applied as public policy, a pilot study with health professionals is needed, which should be supplemented by formal training in SDM.

ACKNOWLEDGEMENTS

We would like to express our gratitude to the three expert reviewers in Shared Decision-Making and breast cancer: Victor Montori, Lilisbeth Perestelo-Pérez and Montserrat Rué; as well as the external reviewers, Lluís Colomés Figuera and Josep Maria Sabaté. We would also like to thank the 20 participants in the study who put their time, effort and perseverance into answering all the rounds of questions that the research team posed them.

FUNDING

Financial support for this study was provided entirely by a grant from Instituto de Salud Carlos III through the project PI18/00773 (co-funded by the European Regional Development Fund), and by the European Union's Horizon 2020 research and innovation programme, under Marie Skłodowska-Curie grant agreement No 713679 from Universitat Rovira i Virgili (URV). The funding agreement ensured the authors' independence in designing the study, interpreting the data, and writing and publishing the report.

DECLARATION OF CONFLICTS OF INTEREST

The authors declare that they have no conflict of interest.

CONTRIBUTORSHIP STATEMENT

For peer review only

For peer review only - http://bmjopen.bmj.com/site/about/guidelines.xhtml

-MJH: Conception and design of work, Data analysis and interpretation, Manuscript writing, Critical review of the manuscript, Adoption of the final version, Contribution of patients or study material, Obtaining funding.

NC: Conception and design of work, Collection/achievement of results, Data analysis and interpretation, Critical review of the manuscript, Adoption of the final version, Contribution of patients or study material.

MJP: Conception and design of work, Data analysis and interpretation, Manuscript writing, Critical review of the manuscript, Adoption of the final version, Contribution of patients or study material.

AC: Conception and design of work, Collection/achievement of results, Data analysis and interpretation, Critical review of the manuscript, Adoption of the final version, Contribution of patients or study material.

CV:Collection/achievement of results, Data analysis and interpretation, Critical review of the manuscript, Adoption of the final version, Contribution of patients or study material.

MC: Conception and design of work, Data analysis and interpretation, Manuscript writing, Critical review of the manuscript, Adoption of the final version, Contribution of patients or study material, Obtaining funding.

REFERENCES

1. Hargraves IG, Montori VM, Brito JP, Kunneman M, Shaw K, LaVecchia C, Wilson M, Walker L, Thorsteinsdottir B. Purposeful SDM: A problem-based approach to caring for patients with shared decision making. Patient Educ Couns. 2019;102(10):1786-1792. doi: 10.1016/j.pec.2019.07.020. Epub 2019 Jul 19. PMID: 31353170; PMCID: PMC6717012.
2. Munthe C, Sandman L, Cutas D. Person Centred Care and Shared Decision Making: Implications for Ethics, Public Health and Research. Health Care Anal [Internet]. 2012;20(3):231–49. Available from: http://link. springer.com/10.1007/s10728-011-0183-y
3. Charles C, Gafni A, Whelan T. Decision-making in the physician-patient encounter: revisiting the shared treatment decision-making model. Soc Sci Med [Internet]. 1999;49(5):651–61. Available from: http://www.ncbi.nlm.nih.gov/pubmed/10452420
4. Sandman L, Granger BB, Ekman I, Munthe C. Adherence, shared decision-making and patient autonomy. Med Health Care Philos. 2012;15(2):115-27. doi: 10.1007/s11019-011-9336-x. PMID: 21678125.
5. Toledo-Chavarri A, Rué M, Codern-Bove N, Carles-Lavila M, Perestelo-Perez L, Perez-Lacasta MJ, and Feijoo-Cid M. A Qualitative Study on a Decision Aid for Breast Cancer Screening: Views from Women and Health Professionals. Eur J Cancer Care (Engl) [Internet]. 2017:26(3). Available from: https://onlinelibrary.wiley.com/doi/abs/10.1111/ecc.12660
6. Generalitat de Catalunya. Departament de Salut. Pla director 2017. [Master plan 2017]. Available from:http://salutweb.gencat.cat/ca/ambits_actuacio/linies_dactuacio/estrategies_salut/cancer/pla_director/

For peer review only - http://bmjopen.bmj.com/site/about/guidelines.xhtml

7. Generalitat de Catalunya. Departament de Salut. Pla director D'Oncologia 2016-2020: Un sistema centrat en la persona: públic, universal i just [Master Oncology Plan 2016-2020: A person-centred system: Public, universal and fair]. 2016. [Internet]. Available from: http://salutweb.gencat.cat/web/.con-tent/_departament/pla-de-salut/Pla-de-salut-2016-2020/documents/ Pla_salut_Catalunya_2016_2020.pdf
8. Institut Català d`Oncologia. Detecció Precoç de Càncer de Mama [Early detection of breast cancer]. [Internet]. 2020 [cited 30 June 2020]. Available from: http://ico.gencat.cat/ca/el-cancer/programes_de_deteccio_precoc/programa_de_deteccio_precoc_de_cancer_de_mama/index.html
9. Légaré F, Witteman H. Shared decision making: examining key elements and barriers to adoption into routine clinical practice. Health Aff (Mill- wood). [Internet]. 2013;32(2):276-84 Available from: https://www.healthaffairs.org/doi/pdf/10.1377/hlthaff.2012.1078
10. Charles C, Gafni A, Whelan T. Self-reported use of shared decision-making among breast cancer specialists and perceived barriers and facilitators to implementing this approach. Health Expect [Internet]. 2004;7(4):338–48. Available from: http://www.ncbi.nlm.nih.gov/pubmed/15544686
11. Hernández-Leal MJ, Pérez-Lacasta M, Feijoo-Cid M, Ramos-García V, Carles-Lavila M. Healthcare professionals behaviour to implement in shared decision making in screening programs: A systematic review. Patient Educ Couns. 2021. doi: https://doi.org/10.1016/j.pec.2021.01.032
12. Álvarez-Torres G. Capítulo 2 Los Manuales [Chapter 2: The Manuals]. En: Manual para elaborar manuales de políticas y procedimientos [Manual for developing policy and procedures manuals]. Mexico D.F.: Panorama editorial, S.A; 1996. p. 23-26.
13. Hernández-Leal MJ, Carles-Lavila M, Pérez-Lacasta M. La participación de los profesionales de la salud en la Toma de Decisiones Compartida en el cribado de cáncer de mama: Manual de apoyo para implementación de la TDC [The participation of health professionals in Shared Decision-Making in breast cancer screening: A handbook to support the implementation of SDM]. Spain: María José Hernández editor; 2020.
14. Croes KD, Jones NR, DuBenske LL, Schrager SB, Mahoney JE, Little TA, Burnside ES. Core Elements of Shared Decision-making for Women Considering Breast Cancer Screening: Results of a Modified Delphi Survey. J Gen Intern Med. 2020;35(6):1668-1677. doi: 10.1007/s11606-019-05298-8. Epub 2020 Mar 19. PMID: 32193817; PMCID: PMC7280383.
15. Elwyn G, Duran M, Song J, Aarts J, J Barr P, Berger Z, etc al. A three-talk model for shared decision making: multistage consultation process. BMJ. 2017;359. [Internet]. Available from:https://www.bmj.com/con-tent/359/bmj.j4891.long
16. Barrett D, Heale R. What are Delphi studies? Evid Based Nurs. 2020 Jul;23(3):68-69. doi: 10.1136/ebnurs-2020-103303. Epub 2020:19. PMID: 32430290.
17. Reguant-Álvarez M. y Torrado-Fonseca M. El método Delphi [The Delphi method]. Revista d'Innovació i Recerca en Educació. Universidad de Barcelona. Institut de Ciéncies de P´Educació. 2016:9(1). Available from: https://revistes.ub.edu/index.php/REIRE/article/view/reire2016.9.1916
18. López-Gómez, E. El método Delphi en la investigación actual en educación: una revisión teórica y metodológica [The Delphi method in current education research: A theoretical and methodological review]. Educación XX1. 2018;21(1):17-40
19. Martínez-Piñeiro M. La técnica Delphi como estrategia de consulta a los implicados en la evaluación de programas [The Delphi technique as a strategy for consulting

For peer review only - http://bmjopen.bmj.com/site/about/guidelines.xhtml

participants in the evaluation of programmes]. RIE. 2003; 21(2): 449-463. Available from: https://revistas.um.es/rie/article/view/99311/94911

20. Benner P, Lee P, Stannard D. (1999). Clinical wisdom in critical care: a thinking- in-action approach. Saunders.
21. García-Ruiz M, Lena-Acebo F. Aplicación del metodo delphi en el diseño de una investigación cuantitativa sobre el fenómeno FABLAB [Application of the Delphi method to the design of quantitative research on the FABLAB phenomenon]. Empiria. Revista de metodología de ciencias sociales, 2018:40;129-166. doi:https://doi.org/10.5944/empiria.40.2018.22014.
22. The SHARE Approach—Essential Steps of Shared Decision Making: Quick Reference Guide. 2014. [Internet]. Available from: https://www.ahrq.gov/health-literacy/curriculum-tools/shareddecisionmaking/tools/tool-1/index.html
23. De las Cuevas C, Perestelo-Perez L, Rivero-Santana A, Cebolla-Martí A, Scholl I, Härter M. Validation of the Spanish version of the 9-item Shared Decision-Making Questionnaire. Health Expect. 2015; 18(6):2143-53. doi: 10.1111/hex.12183. Epub 2014 Mar 5. PMID: 24593044; PMCID: PMC5810694.
24. Shepherd HL, Barratt A, Trevena LJ, McGeechan K, Carey K, Epstein RM, Butow PN, Del Mar CB, Entwistle V, Tattersall MH. Three questions that patients can ask to improve the quality of information physicians give about treatment options: a cross-over trial. Patient Educ Couns. 2011;84(3):379-85. doi: 10.1016/j.pec.2011.07.022. Epub 2011 Aug 9. PMID: 21831558.
25. Elwyn G, Barr PJ, Grande SW, Thompson R, Walsh T, Ozanne EM. Developing CollaboRATE: a fast and frugal patient-reported measure of shared decision making in clinical encounters. Patient Educ Couns. 2013;93(1):102-7. doi: 10.1016/j.pec.2013.05.009. Epub 2013 Jun 12. PMID: 23768763.
26. Martínez KA, Deshpande A, Ruff AL, Bolen SD, Teng K, Rothberg MB. Are Providers Prepared to Engage Younger Women in Shared Decision-Making for Mammography? J Womens Health (Larchmt). 2018;27(1):24-31. DOI: 10.1089/jwh.2016.6047. Epub 2017 Jun 28. PMID: 28657820.
27. Universidad Complutense de Madrid. Guía Docente 2015-2016 [Teaching guide 2015-2016]. Facultad de Medicina. Available from: https://medicina.ucm.es/data/cont/media/www/pag-17227/Gu%C3%ADa%20Medicina%202015-2016.pdf
28. Universitat Autónoma de Barcelona. Programa Bioética y Comunicación 2020/2021 [Bioethics and Communication Programme 2020/2021]. Available from: https://guies.uab.cat/guies_docents/public/portal/html/2020/assignatura/102950/es
29. Complejo Hospitalario Universitario de Badajoz (CHUB). Guía Docente Medicina Intensiva CHUB [CHUB Intensive Care Medicine Teaching Guide]. 2020. Available from: https://www.areasaludbadajoz.com/docencia_investigacion/comision_docencia/Guias_e_Itinerarios_Formativos/Guia_formativa_UCI.pdf
30. Ministerio de Ciencia e Innovación de España. Orden CIN/2134/2008, de 3 de julio, por la que establecen los requisitos para la verificación de los títulos universitarios oficiales que habilitan para el ejercicio de la profesión de Enfermero [Order CIN/2134/2008 of 3 July, establishing the requirements for verification of the official university certificates that act as qualifications for practising the profession of Nurse]. Agencia Estatal Boletín Oficial del Estado. 2008 (174). Available from: https://www.boe.es/eli/es/o/2008/07/03/cin2134
31. Légaré F, Adekpedjou R, Stacey D, Turcotte S, Kryworuchko J, Graham ID, et. al. Interventions for increasing the use of shared decision making by healthcare

For peer review only

For peer review only - http://bmjopen.bmj.com/site/about/guidelines.xhtml

professionals. Cochrane Database Syst Rev. 2018;7(7):CD006732. DOI: 10.1002/14651858.CD006732.pub4. PMID: 30025154; PMCID: PMC6513543.
32. Dois A, Bravo P, Contreras A, Soto MG, Mora I. Formación y competencias para los equipos de atención primaria desde la mirada de expertos chilenos [Training and competencies for primary care teams from the perspective of Chilean experts; Treinamento e competências para equipes de atenção primária na perspectiva de especialistas chilenos]. Rev Panam Salud Publica. 2018 Sep 24;42:e147. Spanish. doi: 10.26633/RPSP.2018.147. PMID: 31093175; PMCID: PMC6386070.
33. García-Altés A, Peiró M, Artells JJ. Priorización de medidas para la consolidación de la toma de decisiones compartidas en las prestaciones del Sistema Nacional de Salud español [Prioritising measures for consolidating shared decision-making in the services rendered by the Spanish National Health System]. Gac Sanit. 2019;33(5):408-414. Spanish. doi: 10.1016/j.gaceta.2018.04.017. Epub 2018 Jul 20. PMID: 30033094.
34. Berkhof M, van Rijssen HJ, Schellart AJ, Anema JR, van der Beek AJ. Effective training strategies for teaching communication skills to physicians: an overview of systematic reviews. Patient Educ Couns. 2011;84(2):152-62. doi: 10.1016/j.pec.2010.06.010. Epub 2010 Jul 29. PMID: 20673620.
35. Perestelo-Perez L, Rivero-Santana A, Perez-Ramos J, Gonzalez-Lorenzo M, Roman JG, Serrano-Aguilar P. Shared decision making in Spain: current state and future perspectives. Z Evid Fortbild Qual Gesundhwes. 2011;105(4):289-95. doi: 10.1016/j.zefq.2011.04.013. Epub 2011 Apr 30. PMID: 21620323.
36. Grupo de trabajo de implicación de pacientes en el desarrollo de GPC. Implicación de Pacientes en el Desarrollo de Guías de Práctica Clínica: Manual Metodológico [Involvement of Patients in the Development of Clinical Practice Guides: Methodological Handbook]. Ministerio de Sanidad, Servicios Sociales e Igualdad. Instituto Aragonés de Ciencias de la Salud-IACS. Guías de Práctica Clínica en el SNS: IACS No 2010/01.
37. The Ottawa Hospital. Patient Decision Aids. Available from: https://decisionaid.ohri.ca/AZsearch.php?criteria=screening

For peer review only

For peer review only - http://bmjopen.bmj.com/site/about/guidelines.xhtml

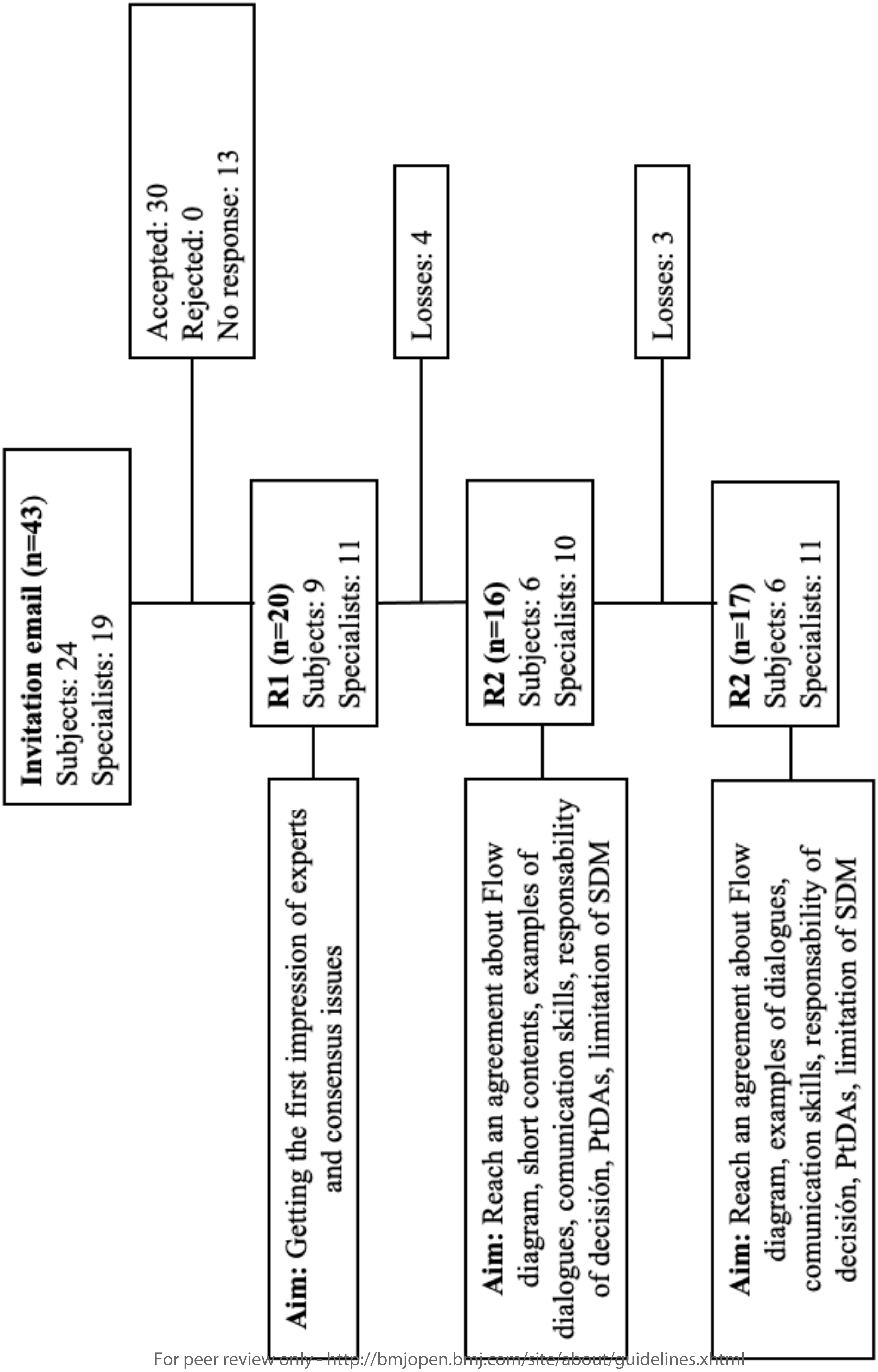
Invitation email (n=43)
Subjects: 24
Specialists: 19
Accepted: 30
Rejected: 0
No response: 13
R1 (n=20)
Subjects: 9
Specialists: 11
Aim: Getting the first impression of experts and consensus issues
Losses: 4
R2 (n=16)
Subjects: 6
Specialists: 10
Aim: Reach an agreement about Flow diagram, short contents, examples of dialogues, comunication skills, responsability of decisión, PtDAs, limitation of SDM
Losses: 3
R2 (n=17)
Subjects: 6
Specialists: 11
Aim: Reach an agreement about Flow diagram, examples of dialogues, comunication skills, responsability of decisión, PtDAs, limitation of SDM

Figura 5: Flujograma del programa de Detección Precoz del Cáncer de Mama

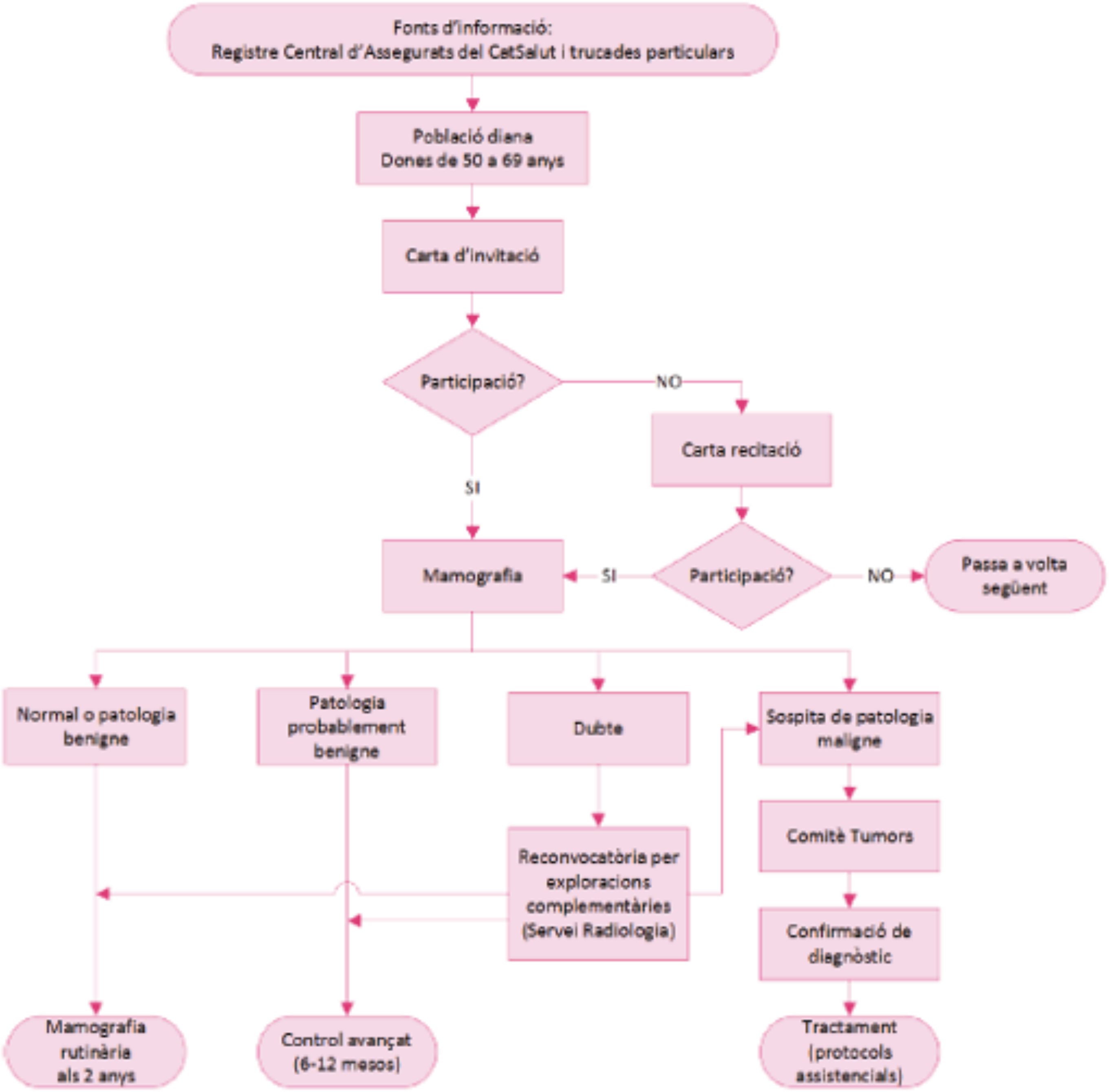

Figure 2 (Annex 4): the clarity and usefulness of this figure was not agreed; therefore, it will be eliminated according to the results of the R3.

First version: Prior to discussion with experts (Annex 4)

ÍNDICE

OBJETIVO DEL MANUAL 6
¿A QUIÉN VA DIRIGIDO? 6
Introducción **7**
Toma de decisiones compartida **13**
1. ¿Qué es? 14
2. ¿Por qué es importante? 17
3. ¿Qué habilidades o competencias necesitan los profesionales sanitarios? 19
4. ¿Qué opinan los pacientes? 22
La toma de decisiones compartida en el cribado de cáncer de mama **25**
1. El Programa de cribado 26
2. Implementación de la TDC en cribado de cáncer de mama 31
3. Autoevaluación del proceso de TDC 40
Referencias bibliográficas **45**

Final version: With the suggestion by the experts (Annex 2)

ÍNDICE

OBJETIVO DEL MANUAL 6
¿A QUIÉN VA DIRIGIDO? 6
Introducción **7**
Toma de decisiones compartida **11**
1. ¿Qué es? 12
2. ¿Por qué es importante? 15
3. Las limitaciones del modelo 17
4. ¿Qué habilidades o competencias necesitan los profesionales sanitarios? 18
5. ¿Qué opinan los pacientes? 22
La toma de decisiones compartida en el cribado de cáncer de mama **25**
1. El Programa de cribado 26
2. Implementación de la TDC en cribado de cáncer de mama 30
3. Autoevaluación del proceso de TDC 39
Referencias bibliográficas **43**

First version: Prior to discussion with experts (Annex 5)	Final version: With the suggestion by the experts (Annex 3)
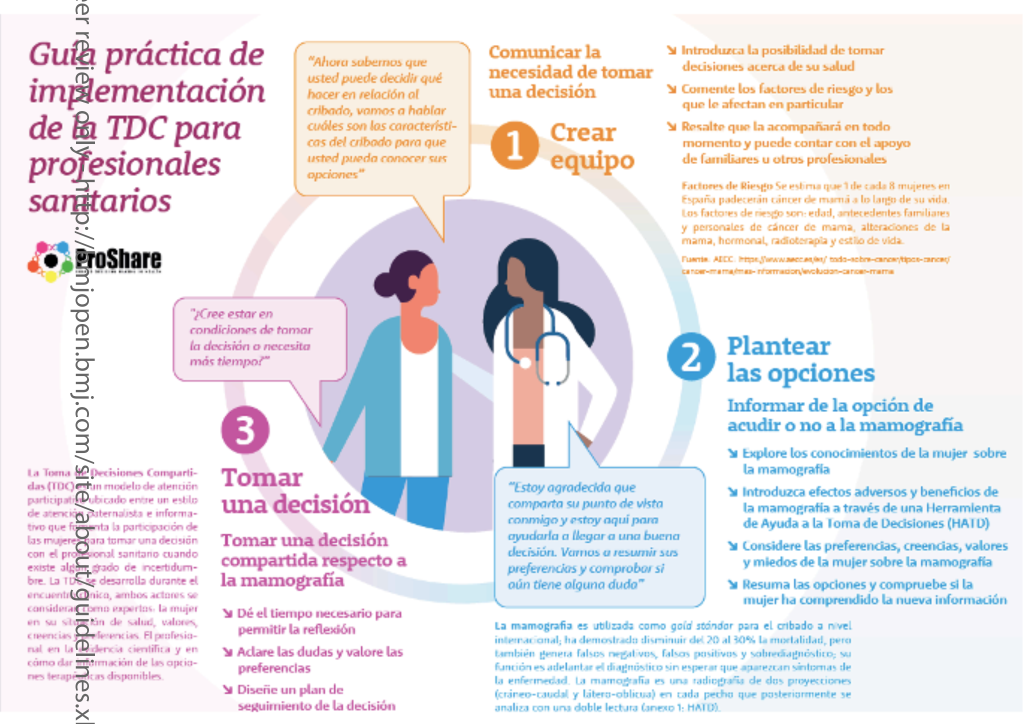	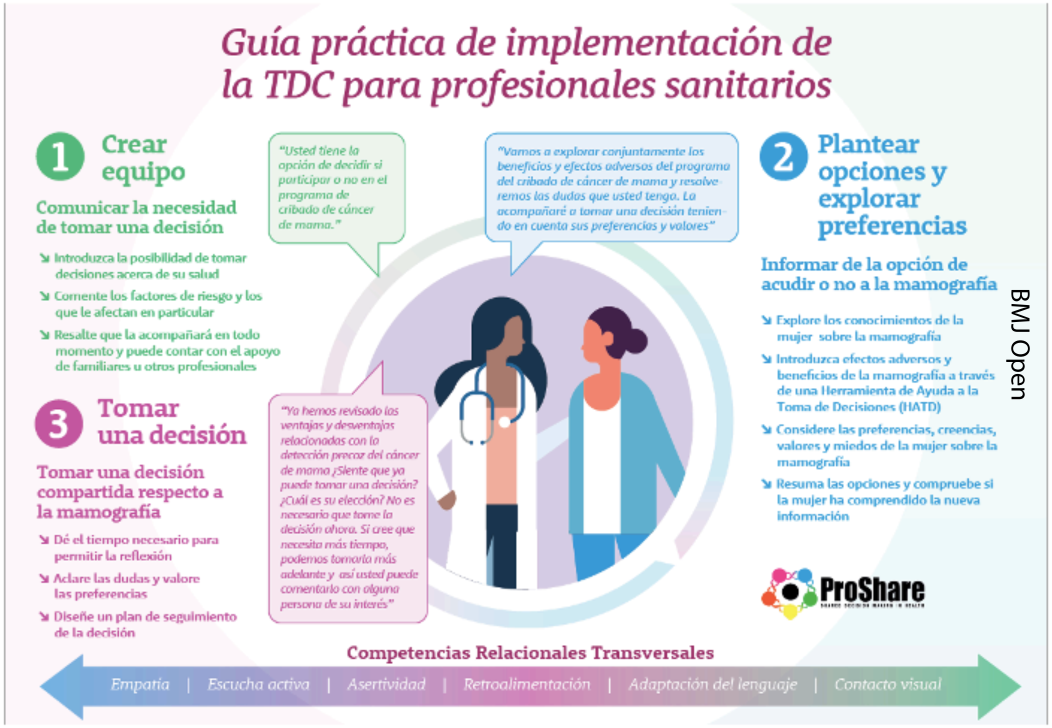

For peer review only - http://bmjopen.bmj.com/site/about/guidelines.xhtml

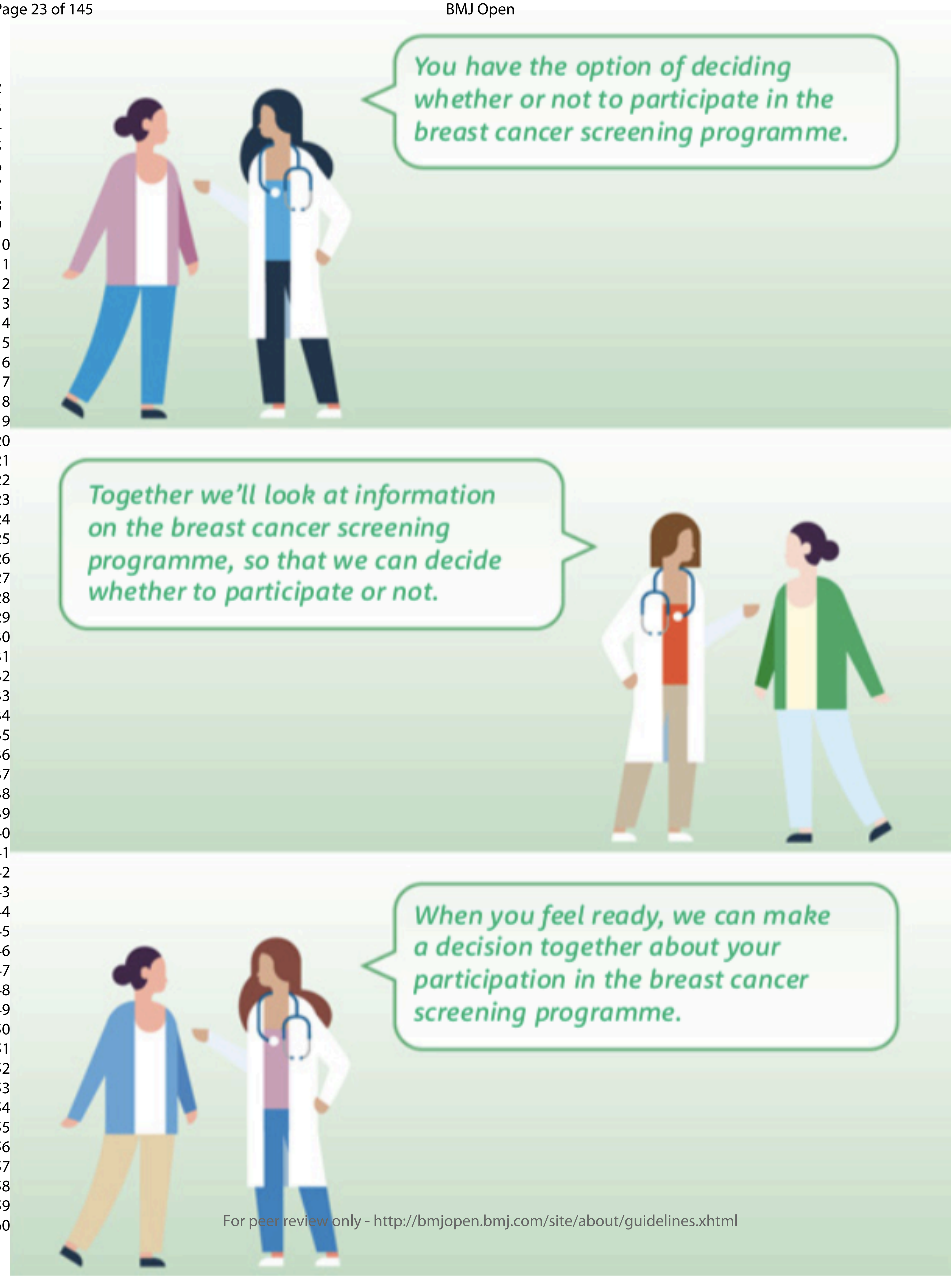
You have the option of deciding whether or not to participate in the breast cancer screening programme.
Together we'll look at information on the breast cancer screening programme, so that we can decide whether to participate or not.
When you feel ready, we can make a decision together about your participation in the breast cancer screening programme.

NO OLVIDES QUE...

La mamografía no evita que tengas cáncer de mama. Además, no es un método perfecto; algunos tumores son muy difíciles de ver en una mamografía.

Puede ser que no tengas cáncer. Pero si lo tuvieras, el diagnóstico y tratamiento en una fase inicial del tumor puede suponer una mayor probabilidad de supervivencia.

Aunque te hayas hecho una mamografía recientemente, es importante que si notas algún cambio en el pecho vayas al médico.

La información presentada en este folleto se ha basado en artículos científicos y materiales desarrollados por el Programa de Cribado de Cáncer de Mama del National Health Service en Inglaterra, por la Colaboración Cochrane y por programas de cribado de Cataluña.

GOBIERNO DE ESPAÑA
MINISTERIO DE ECONOMÍA Y COMPETITIVIDAD
Instituto de Salud Carlos III
Unión Europea
Fondo Europeo de Desarrollo Regional
"Una manera de hacer Europa"

Estudio PI14/00113 Participación de las mujeres en las decisiones y estrategias de detección precoz del cáncer de mama. Cofinanciado por el Instituto de Salud Carlos III y fondos FEDER de la Unión Europea. Participan: Institut de Recerca Biomèdica de Lleida-Universitat de Lleida, Universitat Rovira i Virgili, Institut Català d'Oncologia, Hospital del Mar y Servicio Canario de Salud.

BENEFICIOS Y EFECTOS ADVERSOS A LARGO PLAZO DE LA DETECCIÓN PRECOZ DEL CÁNCER DE MAMA

Si un grupo de 200 mujeres entre 50 y 69 años se hacen mamografías de cribado cada 2 años, cuando cumplan 80 años...

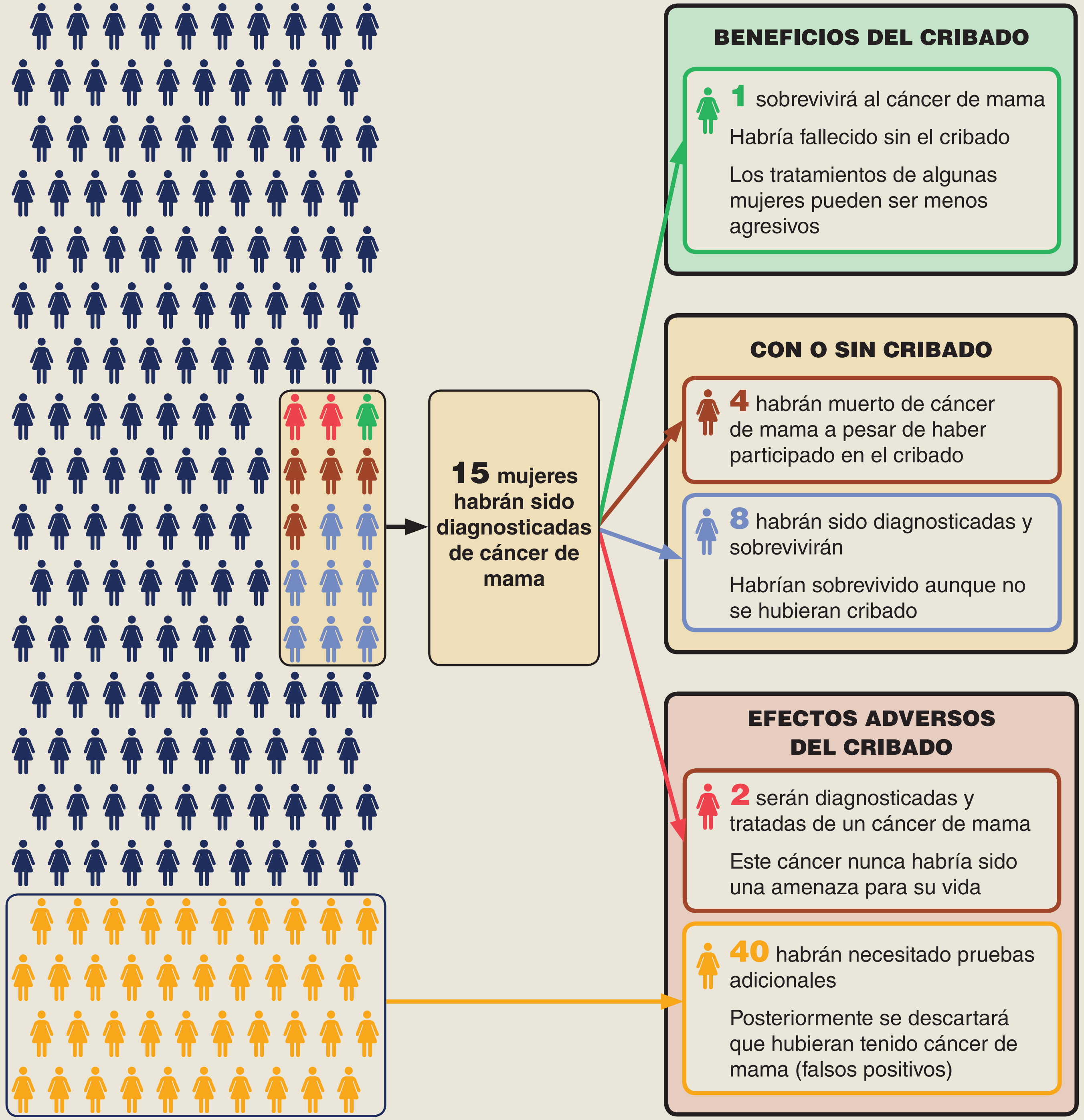

Por cada muerte evitada por el programa de cribado, 2 mujeres son diagnosticadas y tratadas de un cáncer que nunca hubiera puesto en riesgo su vida.

LA DETECCIÓN PRECOZ DEL CÁNCER DE MAMA

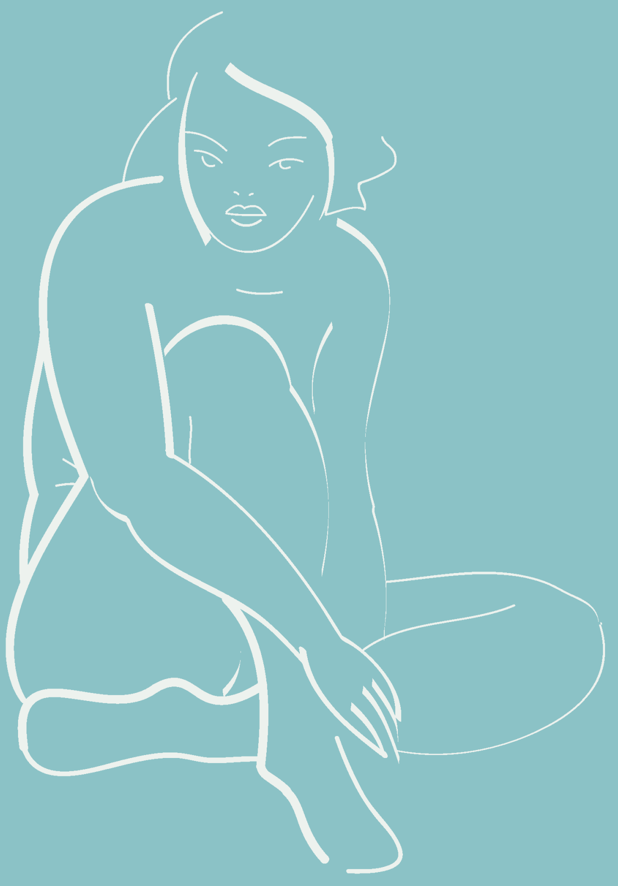

AYUDÁNDOTE A DECIDIR

PARTICIPAR O NO PARTICIPAR EN EL CRIBADO DEL CÁNCER DE MAMA: ESTA ES LA CUESTIÓN

Estudios científicos recientes han identificado efectos adversos, antes desconocidos, de la detección precoz de cáncer de mama mediante mamografía. Por esta razón, este folleto tiene como objetivo informar sobre los beneficios y efectos adversos de participar en la detección precoz de cáncer de mama.

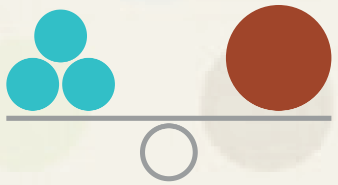

Este material informativo pretende ayudarte a sopesar pros y contras para que puedas tomar una decisión personal sobre si deseas participar o no en la detección precoz del cáncer de mama, en función de tus valores y preferencias.

¿QUÉ ES EL CÁNCER DE MAMA?

El cáncer de mama se desarrolla cuando algunas células empiezan a crecer de forma descontrolada, formando un tumor. A medida que el tumor crece las células malignas se pueden desplazar a otras partes del cuerpo y poner en peligro la vida de la persona afectada.

En Cataluña se diagnostican unos 4.000 casos nuevos de cáncer de mama al año. Las estadísticas nos dicen que 1 de cada 9 mujeres padecerá cáncer de mama a lo largo de su vida y que el 83% de las mujeres afectadas sobrevivirán a esta enfermedad.

¿QUÉ ES LA DETECCIÓN PRECOZ DEL CÁNCER DE MAMA?

La detección precoz del cáncer de mama, también denominada cribado, tiene por objetivo detectar un cáncer de mama en una etapa muy inicial, antes de que aparezcan síntomas. En su etapa inicial, el cáncer es más fácil de tratar y las oportunidades de sobrevivir son superiores.

El sistema sanitario público ofrece la posibilidad de participar en la detección precoz del cáncer de mama con el objetivo de reducir la mortalidad causada por este tumor. El programa de cribado se dirige a las mujeres entre 50 y 69 años y consiste en realizar una mamografía cada dos años.

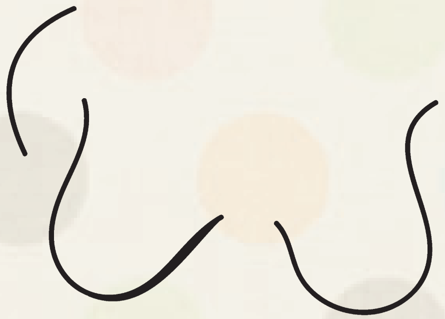

La mamografía es una radiografía de la mama. Es la prueba más eficaz para detectar el cáncer de mama en mujeres que no presentan síntomas. El riesgo de algún daño por la exposición a esta radiación es muy pequeño.

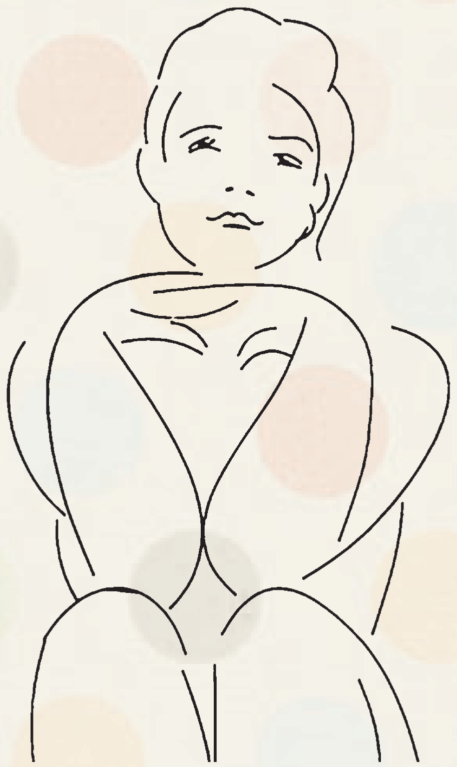

BENEFICIOS DEL CRIBADO

El cribado reduce el riesgo de morir por cáncer de mama

La detección precoz puede salvar la vida a algunas mujeres porque se diagnostican y tratan antes de lo que se habría hecho sin cribado.

De cada 200 mujeres que se realizan mamografías de cribado cada dos años, entre los 50 y los 69 años, 1 mujer se salva gracias a la detección precoz del tumor.

El cribado detecta el cáncer en estadios más iniciales

Un cáncer detectado en estadios iniciales no necesita tratamientos tan agresivos como cuando está más avanzado; estos tratamientos tienen menos efectos secundarios y la probabilidad de recuperación es más alta.

EFECTOS ADVERSOS DEL CRIBADO

Errores en el diagnóstico: falsos positivos y falsos negativos

Los falsos positivos se producen cuando los resultados de la mamografía hacen sospechar de un posible cáncer de mama que en realidad no existe. Esto conlleva exploraciones adicionales que no serían necesarias.

De cada 200 mujeres que se realizan mamografías de cribado cada dos años entre los 50 y los 69 años, 40 tendrán un resultado falso positivo.

La situación contraria, el falso negativo, es mucho menos frecuente y se puede producir cuando la mamografía no muestra ninguna señal de cáncer de mama, aunque la mujer lo padezca.

El cribado puede detectar tumores inofensivos

Algunos tipos de cáncer que se detectan mediante la mamografía de cribado crecen tan lentamente que nunca hubieran llegado a ser un problema de salud. Algunos, incluso, habrían desaparecido de forma espontánea, sin tratamiento.

Actualmente no se puede saber qué lesiones progresarían y cuáles no, y por tanto, se ofrece tratamiento a todas las mujeres diagnosticadas. Algunas mujeres pueden recibir tratamientos que tienen efectos secundarios importantes, sin necesitarlos. Esto se conoce como **sobrediagnóstico** y **sobretratamiento**.

De cada 200 mujeres que se realizan mamografías de cribado cada dos años, entre los 50 y los 69 años, 2 serán tratadas de cáncer sin necesidad.

La participación de los profesionales de la salud en la Toma de Decisiones Compartida en el cribado de cáncer de mama

Manual de apoyo para implementación de la Toma de Decisiones Compartida

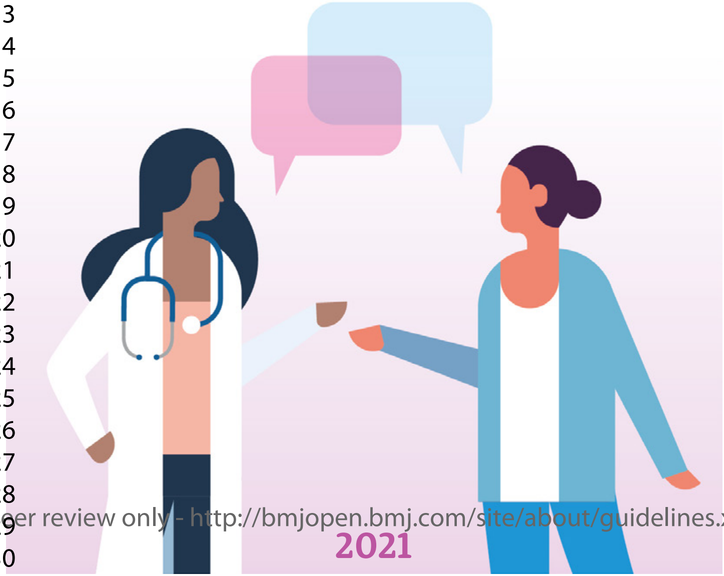

r peer review only - http://bmjopen.bmj.com/site/about/guidelines.xht

2021

For peer review only

La participación de los profesionales de la salud en la Toma de Decisiones Compartida en el cribado de cáncer de mama

Manual de apoyo para implementación de la Toma de Decisiones Compartida

2021

For peer review only - http://bmjopen.bmj.com/site/about/guidelines.xhtml

AUTORES

María José Hernández-Leal[1,a,b,c], María José Pérez-Lacasta[2,a,c], Misericòrdia Carles-Lavila[3,a,b,c] on behalf of the ProShare Group.

1. PhD student, Msc and Bsc Nursing. Mail: mariajose.hernandez@urv.cat
2. PhD, Mail: mariajose.perez@urv.cat
3. PhD. Mail: misericordia.carles@urv.cat

a. Department of Economics, Universitat Rovira i Virgili.
b. Centre de Recerca en Economia i Sostenibilitat (ECO-SOS).
c. Grup de Recerca en Anàlisi Estadístic i Econòmic en Salut (GRAEES).

Equipo de investigación ProShare:

Misericòrdia Carles Lavila
Núria Codern-Bové
María José Hernández Leal
María José Pérez Lacasta
Roger Pla
Vanesa Ramos García
Carmen Vidal

Cómo citar este documento:

Hernández-Leal MJ, Carles-Lavila M, Pérez-Lacasta M. La participación de los profesionales de la salud en la Toma de Decisiones Compartida en el cribado de cáncer de mama: Manual de apoyo para implementación de la TDC. España: María José Hernández editor; 2021.

For peer review only - http://bmjopen.bmj.com/site/about/guidelines.xhtml

For peer review only

EVALUADORES EXTERNOS

Dra. Lilisbeth Perestelo-Pérez
Servicio de Evaluación y Planificación.
Dirección del Servicio Canario de la Salud, España.
Red de Investigación en Servicios de Salud en Enfermedades Crónicas (REDISSEC).

Dr. Victor Montori
Knowledge and Evaluation Research (KER) Unit.
Mayo Clinic - Minnesota, USA.

Dra. Montserrat Rué
Institut de Recerca Biomèdica de Lleida (IRBLLEIDA).
Departament de Ciències Mèdiques Bàsiques.
Universitat de Lleida, España.

DISEÑO

Miquel A. Fernández
www.mafsdisseny.com

FINANCIACIÓN

- The European Regional Development Fund (ERDF). European Union's Horizon 2020 research and innovation programme under the Marie Skłodowska-Curie grant agreement No. 713679 from the Universitat Rovira i Virgili (URV).
- Proyecto PI18/00773 “La colaboración de los profesionales sanitarios para incluir la toma de decisiones compartida en el programa de cribado de cáncer de mama” financiado por el Instituto de Salud Carlos III y cofinanciado por la Unión Europea (FEDER) “Una manera de hacer Europa”

For peer review only - http://bmjopen.bmj.com/site/about/guidelines.xhtml

For peer review only

ÍNDICE

OBJETIVO DEL MANUAL 6

¿A QUIÉN VA DIRIGIDO? 6

Introducción 7

Toma de decisiones compartida 11

1. ¿Qué es? 12
2. ¿Por qué es importante? 15
3. Las limitaciones del modelo 17
4. ¿Qué habilidades o competencias necesitan los profesionales sanitarios? 18
5. ¿Qué opinan los pacientes? 22

La toma de decisiones compartida en el cribado de cáncer de mama 25

1. El Programa de cribado 26
2. Implementación de la TDC en cribado de cáncer de mama 30
3. Autoevaluación del proceso de TDC 39

Referencias bibliográficas 43

For peer review only

For peer review only - http://bmjopen.bmj.com/site/about/guidelines.xhtml

OBJETIVO DEL MANUAL

Este manual pretende ser un documento de referencia que sirva de guía para el desarrollo e implementación de la **Toma de Decisiones Compartida (TDC)** en el cribado del cáncer de mama.

¿A QUIÉN VA DIRIGIDO?

A profesionales sanitarios de la comunidad autónoma de Catalunya relacionados con el cribado de cáncer de mama y que tengan contacto directo con las mujeres que deben participar en el programa.

For peer review only - http://bmjopen.bmj.com/site/about/guidelines.xhtml

Introducción

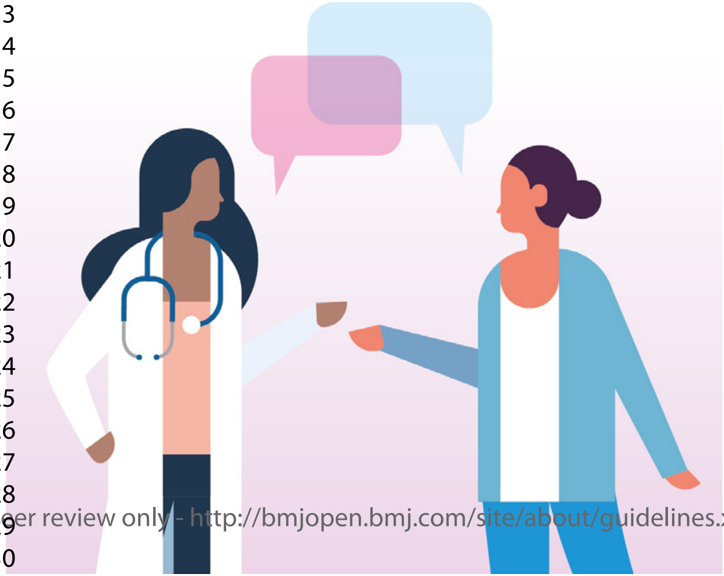

r peer review only - http://bmjopen.bmj.com/site/about/guidelines.xht

El objetivo del **cribado de cáncer de mama** es detectar tempranamente un tumor, en una fase preclínica. Esto permite mejorar las opciones de tratamiento y disminuir la mortalidad[1,2]. A pesar de estos beneficios, el cribado puede producir también efectos adversos: falsos negativos, falsos positivos, sobrediagnóstico y sobretratamiento[2,3,4]. Ante el desconocimiento del grado en que afectarán los efectos positivos y negativos a cada mujer en la decisión del cribado, el modelo de **Toma de Decisiones Compartida** (TDC) permite que pacientes y profesionales de la salud disminuyan la incertidumbre de la decisión[5,6].

La TDC ha sido utilizada principalmente en los países occidentales para mejorar las decisiones en salud de acuerdo a las preferencias de los pacientes y la evidencia científica[7]. Así, en 2012 The European Patients' Forum inició la campaña *"nothing about me, without me"* (nada sobre mí, sin mí)[8] para involucrar a las personas en las decisiones sobre su salud[9].

En este contexto, algunas investigaciones han explorado cómo se desarrolla la TDC, así, por ejemplo, en España solo el 24% de los pacientes afirma haber tomado la decisión conjunta con su profe-

For peer review only

For peer review only - http://bmjopen.bmj.com/site/about/guidelines.xhtml

sional sanitario considerando sus características y preferencias personales o sociales[10]. Esto evidencia la falta de estrategias para involucrar a las personas en su salud, debido a posibles deficiencias en las habilidades comunicativas y la inexistencia de vías de diálogo productivas entre los distintos actores del encuentro clínico[11].

Para mejorar las estrategias, la **Agencia de Calidad y Evaluación Sanitarias de Cataluña** (AQuAs) describe a las Herramienta de Ayuda a la Toma de Decisiones (HATD) como un elemento central para la TDC, aunque actualmente no dispone una de ellas para el cribado de cáncer de mama[12]. Sin embargo, un estudio reciente desarrolló una HATD[13].

A pesar de que en la actualidad ha habido un incremento de materiales destinados a las pacientes[14], son escasos aquellos que ayudan a los profesionales para incorporar las preferencias y los valores de las personas en la toma de decisiones en salud. En este sentido, este documento ofrece a los profesionales sanitarios evidencias científicas sobre la TDC para que sean aplicables al proceso de cribado de cáncer de mama.

For peer review only - http://bmjopen.bmj.com/site/about/guidelines.xhtml

For peer review only

Toma de decisiones compartida

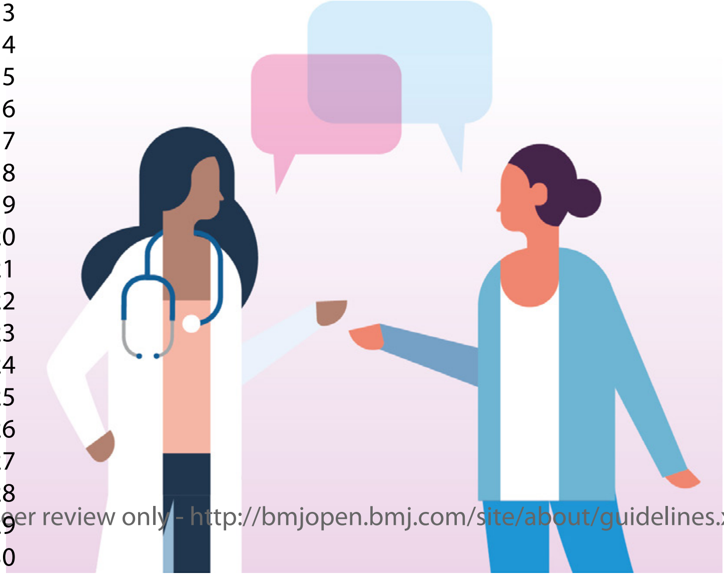

For peer review only - http://bmjopen.bmj.com/site/about/guidelines.xhtml

1. ¿Qué es?

La Toma de Decisiones Compartida (TDC) surge en la década de los 60-70 y corresponde a un modelo de atención participativo ubicado entre el estilo de atención paternalista y el informativo[15,16].

Figura 1: Modelos de atención en salud

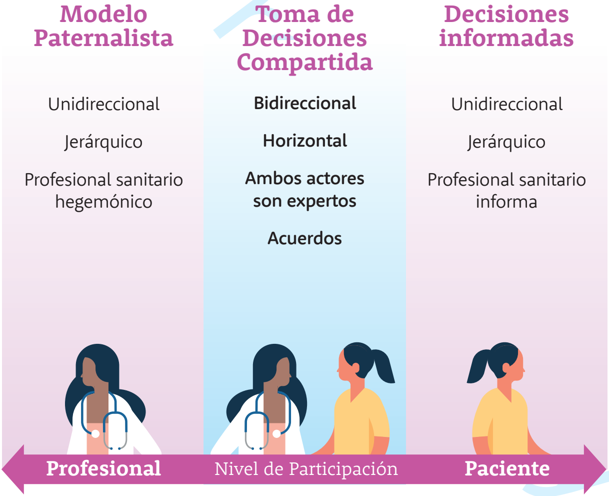

Fuente: Elaboración propia del grupo ProShare

For peer review only

For peer review only

La TDC fomenta la participación del paciente[17] para tomar una decisión conjunta con el profesional sanitario en relación con cambios en el estilo de vida, pruebas diagnósticas, tratamientos y acciones terapéuticas donde pueda existir algún grado de incertidumbre[18,19]. La TDC se desarrolla durante el encuentro clínico y ambos actores son considerados expertos: el paciente en su situación de salud, valores, creencias y preferencias; y el profesional sanitario en la evidencia científica e información de las opciones terapéuticas disponibles[20].

Figura 2: Rol de los participantes en el encuentro clínico

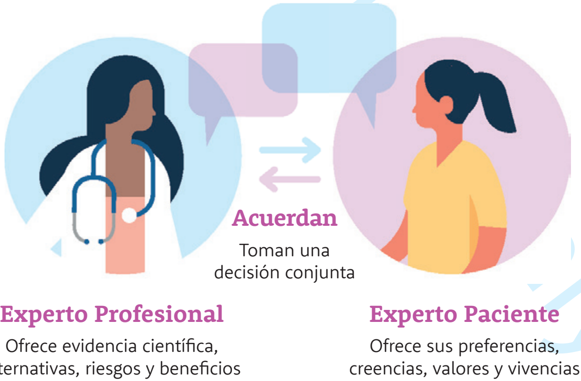

Fuente: Elaboración propia del grupo [illegible]

Por tanto, el objetivo de la TDC se centra en garantizar que las personas tomen decisiones sobre su salud cuando están suficientemente informadas[21]. Para lograrlo se requiere de una negociación continua entre ambos expertos centrando el diálogo en los valores, preferencias, circunstancias del paciente, así cómo en los beneficios, daños, riesgos y opciones terapéuticas ofrecidas por el profesional sanitario. Como resultado final de esta discusión se consigue personas más autónomas, un mayor nivel de compromiso y responsabilidad en su salud[20,22–24].

Figura 3: Elementos de la Toma de Decisiones Compartida

A) Intercambio de información entre el paciente y el profesional sanitario	B) Deliberación sobre las distintas opciones	**C) Tomar una decisión consensuada**

Fuente: Adaptación de Elwyn et al. 2012[20]

For peer review only - http://bmjopen.bmj.com/site/about/guidelines.xhtml

For peer review only

2. ¿Por qué es importante?

La TDC se sustenta en el principio de autonomía de los pacientes. La Ley 21/2000 Derechos de Información relativos a la Salud, Autonomía del Paciente y Documentación Clínica protege su derecho a decidir libremente después de recibir la información adecuada entre las opciones clínicas disponibles[25]. Por tanto, los profesionales están legalmente sujetos al cumplimiento de este principio, y no puede limitarse a la voluntad del profesional.

Junto con lo anterior, la implementación de la TDC ha evidenciado una serie de beneficios en los pacientes, en los profesionales y también en los sistemas sanitarios[10, 24, 26, 27]:

- ✔ Aumenta la participación de los pacientes.
- ✔ Mejora la comunicación paciente-profesional sanitario.
- ✔ Mayor adherencia a tratamientos.
- ✔ Mejora los resultados biométricos en salud.
- ✔ Aumenta la satisfacción de los pacientes en la atención de salud.

For peer review only - http://bmjopen.bmj.com/site/about/guidelines.xhtml

- Disminuye la preocupación y la ansiedad en los pacientes.
- Reduce el conflicto decisional de los pacientes cuando se enfrentan a tomar una decisión diagnóstica y/o terapéutica.
- Mejora el conocimiento de la enfermedad, las opciones diagnósticas y terapéuticas en los pacientes.
- Aumenta la precisión en la percepción del riesgo.
- Aumenta la elección de opciones más beneficiosas.
- Reduce el uso de tratamientos muy invasivos y costosos.
- Reduce la variabilidad injustificada en la práctica asistencial.
- Contribuye a la racionalización del uso de recursos del sistema sanitario.

For peer review only - http://bmjopen.bmj.com/site/about/guidelines.xhtml

3. Las limitaciones del modelo

Aun existen pocos estudios que realicen un seguimiento prolongado a los pacientes para determinar con certeza cuál es el impacto de su aplicación a largo plazo. Por otro lado, existe la creencia entre los profesionales sanitarios que ellos ya aplican la SDM[28]. Sin embargo, algunos estudios han demostrado que esta presunción no se refleja en la práctica[29,30]. Finalmente, aunque los pacientes tienden a elegir las mismas opciones que si no se hubiese aplicado una SDM, se ha demostrado que, en el caso del cribado para el cáncer de mama, las mujeres valoran casi 5 veces más la reducción de la mortalidad que el riesgo de un sobrediagnóstico[31] y esta diferencia radica en un mayor conocimiento, en la adherencia al acuerdo, y la disminución del conflicto decisional.

Manual de apoyo para implementación de la TDC

17

For peer review only - http://bmjopen.bmj.com/site/about/guidelines.xhtml

4. ¿Qué habilidades o competencias necesitan los profesionales sanitarios?

Comunicar de forma equilibrada los riesgos y beneficios de cualquier opción terapéutica no es tarea fácil[26], para conseguirlo se han identificado dos tipos de competencias que deben desarrollar los profesionales sanitarios para aplicarlas en la TDC[32]:

4.1 Competencias relacionales

Son las habilidades que proporcionan un ambiente cómodo para que el paciente comparta sus preocupaciones. Para lograrlo el profesional debe tener un interés genuino en querer involucrarse, comprender el punto de vista del paciente y utilizar un lenguaje sencillo.

For peer review only

Entre las competencias del profesional destacan:

- Realizar una escucha activa.
- Respetar las decisiones tomadas por el paciente.
- Realizar preguntas abiertas.
- Generar en todo momento contacto visual.
- Dejar que los tiempos sean pautados por el paciente.
- Reconocer sus señales emocionales o verbales.
- Usar habilidades comunicacionales: el resumen, la clarificación, el reflejo, la empatía, entre otras[33].

4.2 Competencias de comunicación de riesgo

Son las habilidades que sirven para discutir con el paciente la incertidumbres y comunicar de forma efectiva los riesgos y beneficios de las diferentes opciones. Se debe evaluar la evidencia en relación a cada contexto particular, es decir, considerar los antecedentes personales: historia familiar, historia clínica y factores de riesgo o protectores que podrían aumentar o disminuir los beneficios/daños de las opciones[32].

For peer review only - http://bmjopen.bmj.com/site/about/guidelines.xhtml

Para esto se recomienda evitar el lenguaje técnico, adaptar la cantidad de información a las necesidades actuales del paciente, utilizar diagramas, comprobar la comprensión de la información ofrecida, incorporar valores del paciente a la evidencia, transmitir información objetiva, facilitar la participación y evaluar la información que ya dispone el paciente[34].

En resumen, para poseer una óptima relación con el paciente se resumen en las siguientes habilidades[33] (Figura 4).

Figura 4: Habilidades comunicativas

Escucha	Lenguaje	No verbal	Cultural	Actitudinal
Escucha general y activa	**Verbal:** tono apropiado y adaptado al nivel educativo<br><br>**Escrita:** comunicación clara y uso de material educativo	**Expresivo:** lenguaje corporal y contacto visual<br><br>**Receptivo:** responde a lenguaje corporal y emociones	Adaptar comunicación a la cultura, edad y enfermedad	Respetar las opiniones y derecho de decidir del paciente

Fuente: Adaptación de Laughlin T, Wetmore S, Allen T, Brailovsky C, Crichton T, Bethune C, Donoff M, Lawrence K. 2012[34].

For peer review only - http://bmjopen.bmj.com/site/about/guidelines.xhtml

Si quieres **profundizar en habilidades comunicacionales** revisa los siguientes enlaces:

- Shared decision making and the concept of equipoise: the competences of involving patients in healthcare choices.

  https://bjgp.org/content/bjgp/50/460/892.full.pdf

- The role of physician–patient communication in promoting patient–participatory decision making.

  https://www.ncbi.nlm.nih.gov/pmc/articles/PMC5060521/

- La comunicación médico-paciente: ¿Cuáles son las habilidades efectivas?

  https://scielo.conicyt.cl/pdf/rmc/v138n8/art16.pdf

- Video Decisions Compartides, Generalitat de Catalunya.

  http://decisionscompartides.gencat.cat/ca/inici

For peer review only - http://bmjopen.bmj.com/site/about/guidelines.xhtml

5. ¿Qué opinan los pacientes?

Un estudio desarrollado en España del año 2012 identificó que al 60% de los pacientes les hubiese gustado que el profesional sanitario les pidiese su opinión, aunque no se les animó a hacerlo. Además, la mayoría hubieran deseado recibir más información de la que se les entregó[35]. Otros estudios en el cribado de cáncer de mama han demostrado que sólo entre un 8% a 10% de las mujeres recibieron información respecto al sobrediagnóstico[36].

Los pacientes consideran que el profesional sanitario debe involucrarse en las decisiones de sus pacientes, es decir, no abandonarlos en el proceso de decisión[37]. En 2013, otro estudio determinó cuáles son los elementos que más valoran los pacientes en la TDC, siendo los más significativos el rol comunicativo del profesional sanitario, la percepción de una escucha comprensiva, la sensación de una preocupación real por su salud y por sus necesidades, una conversación acorde al contexto y la constatación de un dominio de la información[38].

For peer review only - http://bmjopen.bmj.com/site/about/guidelines.xhtml

La TDC fortalece la relación profesional sanitario-paciente y la alianza terapéutica porque el hecho de participar activamente en las decisiones de su salud, disminuye la incertidumbre, aumenta el conocimiento y la posibiidad de manejar mejor su enfermedad[37]. En definitiva, los pacientes sienten mayor tranquilidad.

For peer review only

For peer review only - http://bmjopen.bmj.com/site/about/guidelines.xhtml

For peer review only

La toma de decisiones compartida en el cribado de cáncer de mama

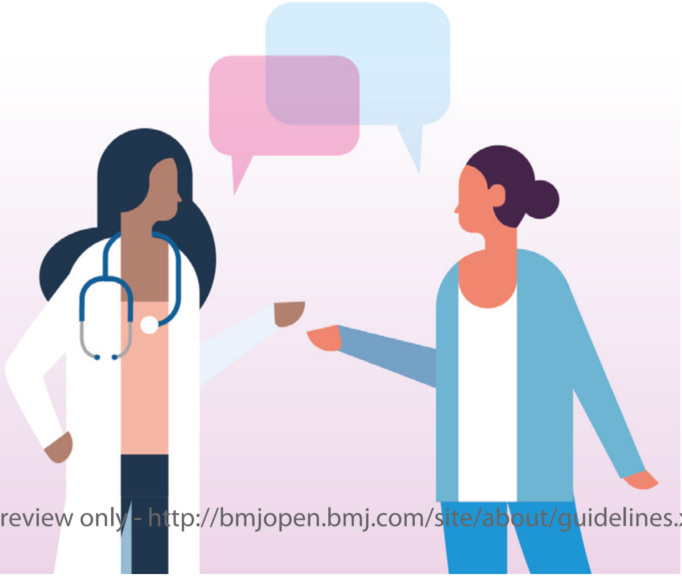

r peer review only - http://bmjopen.bmj.com/site/about/guidelines.xhtr

1. El Programa de cribado

1.1 Cribado poblacional en Cataluña

El Plan Estratégico del *Pla Director d'Oncologia* de la Generalitat de Catalunya menciona como objetivo disminuir el impacto del cáncer de mama en la población mediante el cribado[39]. Sin embargo, no hace referencia a la forma de incorporar a la mujer en las decisiones de su salud, a pesar de que su participación es uno de los pilares del Plan de Salud de Cataluña 2016-2020[40].

Actualmente, cada Programa dependiente del *Pla Director d'Oncologia*, se realiza cada dos años y en base al Registro Central de Asegurados (RCA), la captación de las mujeres entre los 50 y los 69 años mediante una carta dirigida a su domicilio particular. En ella, se les invita a realizar, de forma gratuita, una mamografía en un centro de salud previamente asignado[41]. Este mecanismo no incorpora un espacio de contacto entre el profesional sanitario y la mujer, donde ella pueda resolver sus dudas o inquietudes, ni permite ofrecerle información su-

For peer review only - http://bmjopen.bmj.com/site/about/guidelines.xhtml

ficiente para hacerla partícipe en la decisión sobre su participación -o no- en el programa de cribado.

Para subsanar esta falta de contacto se requiere un cambio en la organización y en los medios de información a las mujeres[42]. Así, el uso de HATD ha demostrado ser un apoyo para el profesional sanitario y para las mujeres en el momento de tomar una decisión conjunta respecto al cribado.

Si quieres **profundizar en HATD** revisa los siguientes enlaces:

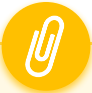

- Documento *Desarrollo de Herramientas de Ayuda para la Toma de Decisiones Compartida derivadas de las recomendaciones de las Guías de Práctica Clínica.*
- Sitios web PyDeSalud: *https://pydesalud.com/toma-de-decisiones-compartidas/*
- Sitio web de The Ottawa Hospital: *https://decisionaid.ohri.ca/AZsearch.php?criteria=screening*
- Video demostrativo *Una Demostración - Toma de decisiones compartidas - Mayo Clinic:* *https://www.youtube.com/watch?v=qwyx7yAP5zA&t=4s*

For peer review only - http://bmjopen.bmj.com/site/about/guidelines.xhtml

1.2 ¿Por qué aplicar la TDC al cribado de cáncer de mama?

Las pruebas diagnósticas para la detección precoz del cáncer de mama han tomado fuerza como estándar de salud pública al reconocer la reducción de la mortalidad, así de cada 200 mujeres que se realizan mamografías de cribado cada dos años, entre los 50 y los 69 años, 1 mujer se salva gracias a la detección precoz del tumor y 40 necesitarán pruebas adicionales para descartar el cáncer[13]. Sin embargo, estudios recientes demuestran que existe una escasa o nula percepción de sus daños o efectos adversos.

Los principales riesgos atribuidos al cribado de cáncer de mama son los falsos positivos, falsos negativos y el sobrediagnóstico[19]. Este último concepto se define como aquellos tumores que crecen tan lentamente que nunca llegarían a ser un problema de salud e incluso desaparecerían de forma espontánea, sin necesidad de tratamiento. Actualmente, se desconoce qué tipo de lesiones progresarán y cuáles no, por tanto, se ofrece tratamiento

For peer review only - http://bmjopen.bmj.com/site/about/guidelines.xhtml

a todas las mujeres diagnosticadas de cáncer de mama (lo que se conoce como sobretratamiento)[13] que provoca una sensación de fragilidad y vulnerabilidad en la mujer, intolerancia a la incertidumbre, vinculación a procedimientos de mayor riesgo, como son las biopsias[43] y, finalmente, se traduce en un aumento del gasto sanitario[8].

Ante la incertidumbre entre los beneficios y efectos adversos, se recomienda el uso de TDC con el fin de definir en base a la evidencia científica actual y los valores de las mujeres la decisión de participar o no en el cribado.

Para mayor información sobre las definiciones, incidencia, estadísticas de riesgo, beneficios y efectos adversos del cribado de cáncer de mamas se pueden encontrar en la HATD desarrollada el año 2016[13].

For peer review only

2. Implementación de la TDC en cribado de cáncer de mama

Antes de iniciar el proceso de TDC se debe establecer una relación de confianza basada en la empatía, facilitar una comunicación fluida y de calidad[23,44]. El proceso debe ser deliberativo[21], es decir, las mujeres toman conciencia que tomarán una decisión y que puede requerir más de un encuentro clínico[21]. Además, este debe ser dinámico ya que las fases deben adaptarse a las necesidades, inquietudes y prioridades de cada mujer[45].

Una vez proporcionada la información, se debe explorar explícitamente si la mujer desea desempeñar un rol activo o pasivo en la decisión[23,46] de lo contrario puede inducir a un rol pasivo en las mujeres[17,23,46]. Sin embargo, no es impedimento para corroborar durante todo el encuentro clínico el rol que desean desempeñar, ya que éste puede cambiar de uno activo a uno pasivo o viceversa en el transcurso de la conversación.

For peer review only

Existen algunos factores que promueven la participación, entre el profesional y la mujer, la motivación de los profesionales de la salud para involucrar a la persona en sus decisiones, la percepción de que la TDC producirá un impacto positivo en el proceso clínico, la alta alfabetización de la mujer o el propio deseo de ella de ser parte activa en las decisiones que afectan a su salud[26,47]. Sin embargo, la falta del tiempo en el encuentro clínico, la edad avanzada de las mujeres, personas con dificultad de comunicarse en el idioma del profesional, el bajo nivel socioeconómico de las mujeres, su baja alfabetización[23,26,46,47] y la presencia de patologías de salud mental[23,43] limitan la TDC.

For peer review only - http://bmjopen.bmj.com/site/about/guidelines.xhtml

2.1 Fases y modelo *"Three-talk"*

Las tres fases que componen este modelo son[48]:

FASE 1
Crear equipo

El principal objetivo de esta fase es comunicar la necesidad de tomar una decisión en equipo, cuyos integrantes son el profesional sanitario y la mujer. Aquí se comunican los objetivos de la decisión, por qué se debe tomar (presencia de factores de riesgo personal) y las alternativas disponibles basadas en la evidencia. El profesional debe enfatizar en que (a) la mujer pude decidir no tomar una decisión en ese momento y solicitar el apoyo de otros actores como familiares u otros especialistas y (b) debe ser receptivo a las reacciones que puede generar en la mujer el enfrentarse a esta decisión. Por tanto, debe recalcar que la acompañará en el proceso hasta que se sienta segura para llevar a cabo la decisión.

For peer review only - http://bmjopen.bmj.com/site/about/guidelines.xhtml

Figura 5: Crear equipo

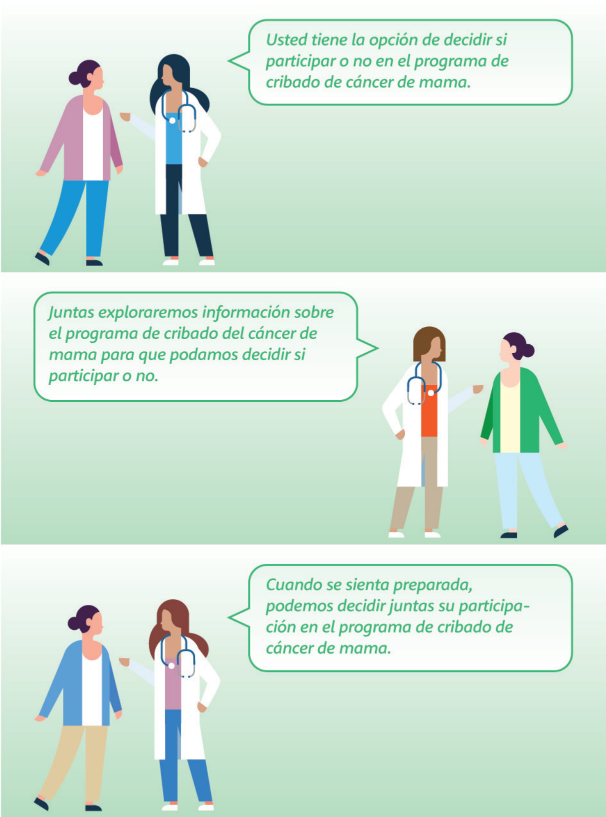

Fuente: Elaboración propia del grupo [illegible]

Manual de apoyo para implementación de la TDC

33

or peer review only - http://bmjopen.bmj.com/site/about/guidelines.xhtr

FASE 2

Plantear las opciones y explorar preferencias

El principal objetivo de esta fase es informar claramente, según las características de cada mujer, los efectos adversos y beneficios del cribado. Para esto debe explorar sus valores, sus preocupaciones, expectativas y preferencias iniciales (prioridades basadas en los conocimientos preexistentes o ideas preconcebidas respecto al cribado[21]). Además ampliar en detalle las opciones, considerando riesgos y beneficios. De esta forma, las preferencias iniciales pasarán a ser preferencias informadas (preferencias personales basadas en los valores una vez que se ha asegurado la comprensión de los riesgos y beneficios más relevantes del cribado[21]).

Para explicar los riesgos específicos se recomienda utilizar alguna HATD, ya que mejorará la comprensión de la información incluso en mujeres con baja alfabetización[13].

For peer review only - http://bmjopen.bmj.com/site/about/guidelines.xhtml

Figura 6: Plantear las opciones y explorar preferencias

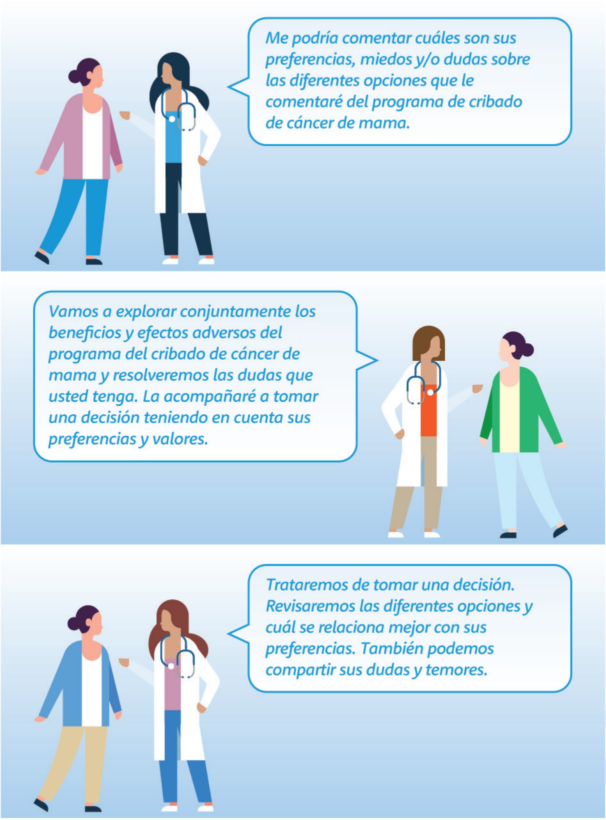

Fuente: Elaboración propia del grupo ProShare.

For peer review only - http://bmjopen.bmj.com/site/about/guidelines.xhtml

FASE 3

Tomar una decisión compartida

En esta fase se argumentan las alternativas y se toma una decisión respecto a la participación en el cribado de cáncer de mama[48]. El profesional debe reforzar la idea de acompañamiento en la decisión. Además, debe dar el tiempo suficiente que permita a la mujer reflexionar en torno a sus prioridades, incluso incluyendo la idea de diferirla para otro momento o delegarla en el profesional; en este último caso se recomienda identificar los elementos que le impiden hacerlo por ella misma. Finalmente, debe confirmar la decisión y acordar un plan de acción[23,46] y seguimiento que permita un circuito de retroalimentación entre el profesional y la mujer[45,46].

For peer review only

Figura 7: Tomar una decisión compartida

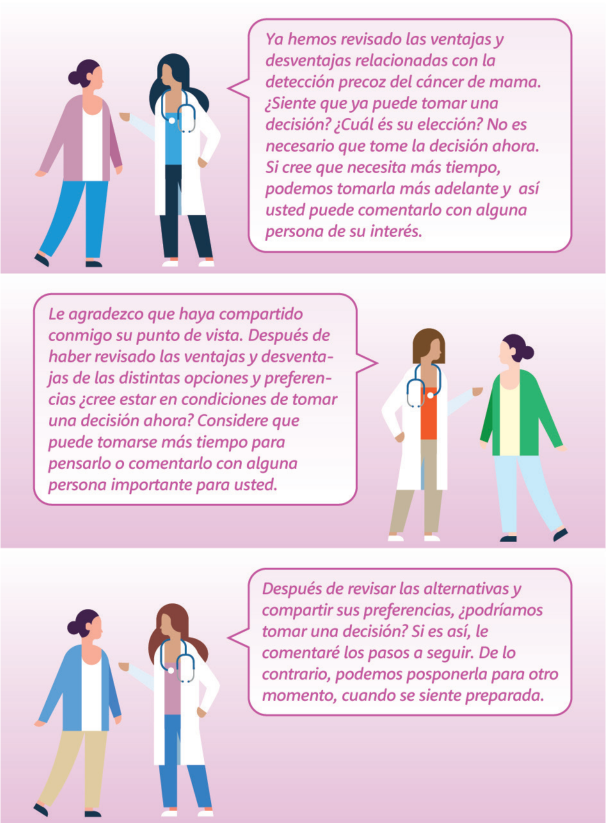

Manual de apoyo para implementación de la TDC

37

Fuente: Elaboración propia del grupo [illegible]

For peer review only - http://bmjopen.bmj.com/site/about/guidelines.xhtml

Figura 8: Fases de la Toma de Decisiones Compartida

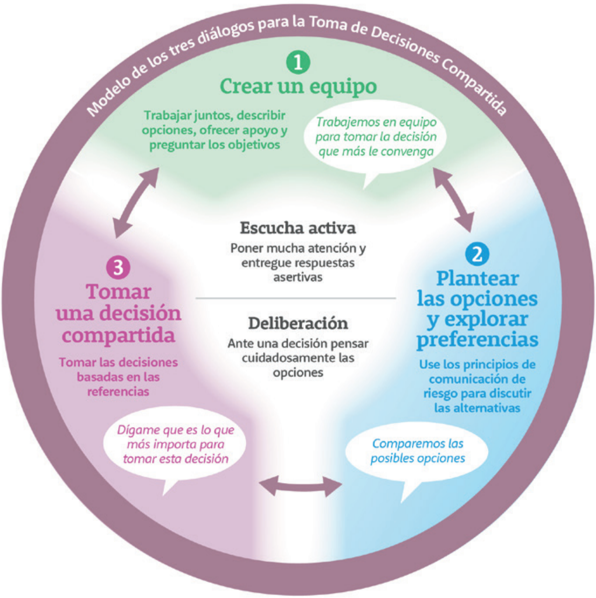

Fuente: Adaptación de Three-talk model of shared decision making. Elwyn G, et al 2017. Uso autorizado por el autor[48].

For peer review only - http://bmjopen.bmj.com/site/about/guidelines.xhtml

3. Autoevaluación del proceso de TDC

El instrumento SDM-Q-doc[49] es una encuesta de autoevaluación para profesionales sanitarios que permite medir el nivel de participación que se le ha ofrecido a la mujer para tomar decisiones. Este instrumento está compuesto por nueve ítems que deben ser valorados por el profesional dentro de seis alternativas; desde totalmente en desacuerdo (valor 1) a totalmente de acuerdo (valor 6).

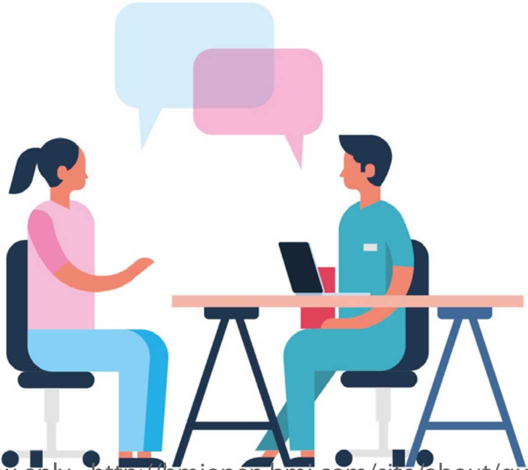

For peer review only - http://bmjopen.bmj.com/site/about/guidelines.xhtml

Criterio	Puntos* 1	2	3	4	5	6
Informé claramente a la mujer de la necesidad de tomar una decisión sobre su participación en el cribado de cáncer de mama	☐	☐	☐	☐	☐	☐
Pregunté a la mujer de forma precisa cómo le gustaría participar en la toma de decisiones	☐	☐	☐	☐	☐	☐
Informé a la mujer que existe la opción de participar o no en el cribado	☐	☐	☐	☐	☐	☐
Expliqué claramente a la mujer las ventajas y desventajas de cada opción	☐	☐	☐	☐	☐	☐
Ayudé a la mujer a entender toda la información sobre beneficios y efectos adversos	☐	☐	☐	☐	☐	☐
Pregunté a la mujer qué opción prefería	☐	☐	☐	☐	☐	☐
La mujer y yo hemos valorado ampliamente todas las opciones	☐	☐	☐	☐	☐	☐
La mujer y yo hemos escogido conjuntamente una opción	☐	☐	☐	☐	☐	☐
La mujer y yo nos hemos puesto de acuerdo sobre el seguimiento de su atención sanitaria posterior	☐	☐	☐	☐	☐	☐

* La puntuación va de: "Totalmente en desacuerdo" (1) a "Totalmente de acuerdo" (6)

Fuente: SDM-Q-doc adaptado al cribado de cáncer de mama.

For peer review only - http://bmjopen.bmj.com/site/about/guidelines.xhtml

Procedimiento y Resultados de la autoevaluación

Para conocer el resultado se deben sumar los puntos obtenidos en cada sección, identificando así las fases del modelo "*Three-talk*".

Fase de la TDC	Puntos	Interpretación
Fase 1 **"Crear equipo"**		3 a 12 puntos: sin adherencia a la TDC<br>13 a 18 puntos: adherente a la TDC
Fase 2 **"Plantear opciones y explorar preferencias"**		3 a 12 puntos: sin adherencia a la TDC<br>13 a 18 puntos: adherente a la TDC
Fase 3 **"Tomar una decisión"**		3 a 12 puntos: sin adherencia a la TDC<br>13 a 18 puntos: adherente a la TDC
**Puntuación total:**		**9 a 27:** Falta de adherencia a la TDC<br>**28 a 36:** Indiferencia a la TDC<br>**37 a 45:** Leve adherencia a la TDC<br>**46 a 54:** Fuerte adherencia a favor de la TDC

For peer review only - http://bmjopen.bmj.com/site/about/guidelines.xhtml

La interpretación y puntos de corte se basaron en la evidencia de Pollard, Bansback y Bryan (2015)[5]. Se dividieron los puntos totales (54 puntos) según los porcentajes de corte descritos; >80% "Fuerte adherencia del profesional a favor de la TDC"; 60-80% "Leve adherencia del profesional a la TDC"; 40-60% "Indiferencia del profesional a la TDC" y <40% "Falta de adherencia del profesional a la TDC". Por otro lado, los puntos de corte por cada fase definen como "adherente" aquellos superiores al 60% y "sin adherencia" a la TDC el resto, de un total de 15 puntos (3 al 18). Finalmente, la división por fases corresponde a una adaptación de la escala SDM-Q-doc contrastándola con las características del modelo "*Three-talk*"[48].

Se recomienda realizar esta autoevaluación con periodicidad ya que permite a los profesionales sanitarios identificar los puntos fuertes y débiles en cuanto a la forma en que incorporan la participación en salud de las mujeres. De este modo, se facilita focalizar los esfuerzos en mejorar los aspectos más débiles con formación y entrenamiento, y finalmente hacer un seguimiento de los avances conseguidos.

For peer review only

For peer review only

Referencias bibliográficas

1. Organización Mundial de la Salud. (WHO) Control del cáncer de mama; 2020 [citado 30 de junio de 2020]. Available from: https://www.who.int/topics/cancer/breastcancer/es/index3.html
2. Febles G. Revisión de los beneficios del screening del cáncer de mama y análisis de las controversias. *Rev. Imagenol.* [Internet] 2015;19 (1)9-18. Available from: https://www.webcir.org/revistavirtual/articulos/2016/4_noviembre/ur/febles_esp.pdf
3. Day N. Overdiagnosis and breast cancer screening. *Breast Cancer Res* [Internet]. 2005;7(5):228–9. Available from: http://www.ncbi.nlm.nih.gov/pubmed/16168144
4. Nehmat H. Overdiagnosis of breast cancer in population screening: does it make breast screening worthless? *Cancer Biol Med* [Internet]. 2017;14(1):1–8. Available from: http://www.cancerbiomed.org/index.php/cocr/article/view/1003
5. Pollard S, Bansback N, Bryan S. Physician attitudes toward shared decision making: A systematic review. *Patient Educ Couns* [Internet]. 2015; 98(9):1046-57. Available from: https://www.sciencedirect.com/science/article/abs/pii/S0738399115002256?via%3Dihub
6. Munthe C, Sandman L, Cutas D. Person Centred Care and Shared Decision Making: Implications for Ethics, Public Health and Research. *Health Care Anal* [Internet]. 2012;20(3):231–49. Available from: http://link.springer.com/10.1007/s10728-011-0183-y
7. Barry M, Edgman-Levitan S. Shared Decision Making — The Pinnacle of Patient-Centered Care. *N Engl J Med* [Internet]. 2012;366(9):780–1. Available from: http://www.nejm.org/doi/10.1056/NEJMp1109283
8. Coulter A, Collins A. Making shared decision-making a reality. No decision about me, without me [Internet]. The King's Fund. Edwina Rowling, editor. London; 2011. Available from: www.kingsfund.org.uk
9. European patients' forum. 150 Million reasons to act; *EPF's Patients' Manifesto for the European Parliament and Commission*. [Internet]. 2009. Available from: https://www.eu-patient.eu/globalassets/events/old/manifesto/epf_manifesto.pdf

For peer review only - http://bmjopen.bmj.com/site/about/guidelines.xhtml

10. Confederación Crohn y Colitis Ulcerosa. Se incumple el modelo de decisiones compartidas con los pacientes [Internet]. Available from: https://www.accuesp.com/se-incumple-el-modelo-de-decisiones-compartidas-con-los-pacientes

11. Ministerio de Sanidad, Consumo y Bienestar Social de España. Marco Estratégico para la Atención Primaria y Comunitaria [Internet]. 2019. Available from: https://www.mscbs.gob.es/profesionales/proyectosActividades/docs/Marco_Estrategico_APS_25Abril_2019.pdf

12. Agencia de Calidad y Evaluación Sanitarias de Cataluña (AQuAs). Estado de situación de las herramientas de ayuda a la toma de decisiones clínicas en Cataluña. [Internet]. 2020 [citado 30 de junio de 2020]. Available from: http://aquas.gencat.cat/ca/ambits/avaluacio-tecnologies-qualitat/guies/eines_ajut_presa_decisions_catalunya/

13. Toledo-Chavarri A, Rué M, Codern-Bove N, Carles-Lavila M, Perestelo-Perez L, Perez-Lacasta MJ, and Feijoo-Cid M. A Qualitative Study on a Decision Aid for Breast Cancer Screening: Views from Women and Health Professionals. *Eur J Cancer Care (Engl)* [Internet]. 2017. 26(3). Available from: https://onlinelibrary.wiley.com/doi/abs/10.1111/ecc.12660

14. Grupo Español de Investigación en Cáncer de Mama. Guía GEICAM de Práctica Clínica: Para el Diagnóstico y Tratamiento del Cáncer de Mama Metastásico. Edición: Comuniland S.L. Madrid; 2015. Available from: https://portal.guiasalud.es/wp-content/uploads/2019/06/gpc_538_geicam_cancer_mama_resumida.pdf

15. Charles C, Gafni A, Whelan T. Self-reported use of shared decision-making among breast cancer specialists and perceived barriers and facilitators to implementing this approach. *Health Expect* [Internet]. 2004 Dec;7(4):338–48. Available from: http://www.ncbi.nlm.nih.gov/pubmed/15544686

16. Barr P, Scholl I, Bravo P, Faber M, Elwyn G, McAllister M. Assessment of Patient Empowerment - A Systematic Review of Measures. *PLoS One* [Internet]. 2015;10(5). Available from: http://journals.plos.org/plosone/article/file?id=10.1371/journal.pone.0126553&type=printable

For peer review only

For peer review only - http://bmjopen.bmj.com/site/about/guidelines.xhtml

17. Elwyn G, Edwards A, Kinnersley P, Grol R. Shared decision making and the concept of equipoise: the competences of involving patients in healthcare choice. *Br J Gen Pract* [Internet]. 2000;50:892–7. Available from: https://bjgp.org/content/bjgp/50/460/892.full.pdf

18. Participa y decide sobre tu salud. ¿Qué es la toma de decisiones compartida? [Internet]. 2020 [citado 30 de junio de 2020]. Available from: https://www.pydesalud.com/toma-de-decisiones-compartidas/

19. Marmot M, Altman D, Cameron D, Dewar J, Thompson S, Wilcox M. The benefits and harms of breast cancer screening: An independent review. *Br J Cancer* [Internet]. 2013;108(11): 2205–2240. Available from: https://www.ncbi.nlm.nih.gov/pmc/articles/PMC3693450/

20. Reyes A. Toma de decisiones compartidas: por una medicina cuidadosa y amable [Internet]. Parte 1. *Por una Medicina Interna de Alto Valor*. 2018. Available from: https://medicinainternaaltovalor.fesemi.org/el-universo-del-paciente/toma-de-decisiones-compartidas-por-una-medicina-cuidadosa-y-amable-parte-1/

21. Elwyn G, Frosch D, Thomson R, Joseph-Williams N, Lloyd A, Kinnersley P, et al. Shared decision making: a model for clinical practice. *J Gen Intern Med* [Internet]. 2012;27(10):1361–7. Available from: http://www.ncbi.nlm.nih.gov/pubmed/22618581

22. Peralta L, Ruiz J, Castillo R. La participación del paciente en la toma de decisiones en las consultas de atención primaria. *Universidad de Cantabria*. 2010; Available from: http://www.tesisenred.net/bitstream/handle/10803/10705/TesisLPM.pdf?sequence=1

23. Información farmacoterapéutica de la Comarca. Participación del paciente en la toma de decisiones. *INFAC* [Internet]. 2014;22(3):12–7. Available from: https://www.euskadi.eus/contenidos/informacion/cevime_infac_2014a/es_def/adjuntos/INFAC_Vol_22_n_3_Decisiones_compartidas.pdf

24. Bravo P, Contreras A. Perestelo-Pérez L, Pérez-Ramos J, Málaga G. En busca de una salud más participativa: Compartiendo decisiones de salud. *Rev Peru Med Exp Salud Publica*. 2013;30(4):691-7.

25. Boletín Oficial del Estado. Ley 14/1986 de 2 de febrero, General de Sanidad. BOE de 21/2000. [Internet]. Available from: https://www.boe.es/boe/dias/2001/02/02/pdfs/A04121-04125.pdf

Manual de apoyo para implementación de la TDC

45

For peer review only

26. Gravel K, Légaré F, Graham I. Barriers and facilitators to implementing shared decision-making in clinical practice: a systematic review of health professionals' perceptions. *Implementation Sci*. 2006;1(16). Available from: http://www.implementationscience.com/content/1/1/16

27. Légaré F, Ratté S, Stacey D, Kryworuchko J, Gravel K, Graham ID, et al. Interventions for improving the adoption of shared decision making by health-care professionals. *Cochrane Database of Systematic Reviews* [Internet]. Chichester, UK: John Wiley & Sons, Ltd; 2010. p. CD006732. Available from: http://www.ncbi.nlm.nih.gov/pubmed/20464744

28. Stiggelbout AM, Pieterse AH, De Haes JC. Shared decision making: Concepts, evidence, and practice. *Patient Educ Couns.* 2015;(10):1172-9. doi: 10.1016/j.pec.2015.06.022. Epub 2015 Jul 15. PMID: 26215573.

29. Hernández-Leal M, Perestelo-Pérez L, Bravo P. Percepción de los profesionales de salud para la Toma de Decisiones Compartida en Atención Primaria. *Revista chilena de medicina familiar*. forthcoming.

30. Couët N, Desroches S, Robitaille H, Vaillancourt H, Leblanc A, Turcotte S, Elwyn G, Légaré F. Assessments of the extent to which health-care providers involve patients in decision making: a systematic review of studies using the OPTION instrument. *Health Expect.* 2015;18(4):542-61. doi: 10.1111/hex.12054.

31. Pérez-Lacastra, Carles-Lavila M, Codern-Bové N, Cardona-Cardona A, Rué-Monné M. Eliciting women's preferences for the design of a breast cancer screening program. *Patient Educ Couns.* forthcoming.

32. Légaré F, Moumjid-Ferdjaoui N, Drolet R, Stacey D, Härter M, Bastian H, et al. Core competencies for shared decision making training programs: insights from an international, interdisciplinary working group. *J Contin Educ Health Prof* [Internet]. 2013;33(4):267–73. Available from: http://www.ncbi.nlm.nih.gov/pubmed/24347105

33. Ford S, Schofield T, Hope T. What are the ingredients for a successful evidence-based patient choice consultation? A qualitative study. *Soc Sci Med* [Internet]. 2003;56:589–602. Available from: https://pubmed.ncbi.nlm.nih.gov/12570976/

For peer review only

34. Laughlin T, Wetmore S, Allen T, et al. Defining competency-based evaluation objectives in family medicine: communication skills. *Can Fam Physician* [Internet]. 2012;58(4):e217–e224. Available from: https://www.ncbi.nlm.nih.gov/pmc/articles/PMC3325474/.

35. Ruiz R, Peralta L, Pérula L, Olloqui Mundet J, Carrión T, Sobrino A, et al. Opiniones y percepciones de los pacientes sobre su participación en la toma de decisiones en las consultas de medicina de familia. *Aten Primaria* [Internet]. 2012;44(1):5–10. Available from: https://www.sciencedirect.com/science/article/pii/S0212656711001545?via%3Dihub

36. Hersch, J. Jansen & K. McCaffery (2018) Decision-making about mammographic screening: pursuing informed choice, Climacteric, 21:3, 209-213, DOI: 10.1080/13697137.2017.1406912

37. Moreau A, Carol L, Dedianne M, Dupraz C, Perdrix C, Lainé X, et al. What perceptions do patients have of decision making (DM)? Toward an integrative patient-centered care model. A qualitative study using focus-group interviews. *Patient Educ Couns* [Internet]. 2012:1;87(2):206–11. Available from: https://www.sciencedirect.com/science/article/abs/pii/S0738399111004599

38. Thorne S, Oliffe JL, Stajduhar KI. Communicating shared decision-making: Cancer patient perspectives. Patient Educ Couns [Internet]. 2013;90(3):291–6. Available from: https://www.sciencedirect.com/science/article/abs/pii/S0738399112000912

39. Generalitat de Catalunya. Departament de Salut. Pla director 2017. [Internet]. Available from: http://salutweb.gencat.cat/ca/ambits_actuacio/linies_dactuacio/estrategies_salut/cancer/pla_director/

40. Generalitat de Catalunya. Departament de Salut. Pla director D'Oncologia 2016-2020: Un sistema centrat en la persona: públic, universal i just. 2016. [Internet]. Available from: http://salutweb.gencat.cat/web/.content/_departament/pla-de-salut/Pla-de-salut-2016-2020/documents/Pla_salut_Catalunya_2016_2020.pdf

41. Institut Català d'Oncologia. Detecció Precoç de Càncer de Mama. [Internet]. 2020 [citado 30 de junio de 2020]. Available from: http://ico.gencat.cat/ca/el-cancer/programes_de_deteccio_precoc/programa_de_deteccio_precoc_de_cancer_de_mama/index.html

For peer review only - http://bmjopen.bmj.com/site/about/guidelines.xhtml

42. Servicio de epidemiología y Prevención del cáncer (SEPC). Esquema funcional del Programa de Detección Precoz del Cáncer de Mama. [Internet]. 2020 [citado 30 de junio de 2020]. Available from: https://epicancer.cat/prevencion_cancer_mama.

43. Coll-Benejam T, Bravo-Toledo R , Marcos-Calvo M, Astier-Peña M. Impacto del sobrediagnóstico y sobretratamiento en el paciente, el sistema sanitario y la Sociedad. *Aten Primaria.* [Internet]. 2018;50(S2):86-95. Available from: https://www.elsevier.es/es-revista-atencion-primaria-27-articulo-impacto-del-sobrediagnostico-sobretratamiento-el-S0212656718305158

44. Rowland K, Politi M. Shared Decision-Making and the Patient-Provider Relationship. In: Handbook of Health Decision Science [Internet]. New York, NY: Springer New York; 2016. p. 181–92. Available from: http://link.springer.com/10.1007/978-1-4939-3486-7_14

45. Elwyn G, Petronella Catharina Anna Vermunt N. Goal-Based Shared Decision-Making: Developing an Integrated Model. *Journal of Patient Experience*. 2019;1-9. Available from: https://journals.sagepub.com/doi/full/10.1177/2374373519878604

46. Barani M, Kopitowski K. Toma de decisiones compartidas: centrando los cuidados médicos realmente en nuestros pacientes. *Rev Hosp Ital BAires* [Internet]. 2013;33(2):60–4. Available from: https://www.hospitalitaliano.org.ar/multimedia/archivos/noticias_attachs/47/documentos/14745_60-64-HI-33-2-2BARANI_A.pdf

47. Hernández-Leal, MJ, Pérez-Lacasta MJ, Feijoo-Cid M, Ramos-García V, Carles-Lavila M. Healthcare professionals' behaviour regarding the implementation of shared decision-making in screening programmes: A systematic review. Patient Educ Couns [Internet]. 2021. https://doi.org/10.1016/j.pec.2021.01.032

48. Elwyn G, Duran M, Song J, Aarts J, J Barr P, Berger Z, etc al. A three-talk model for shared decision making: multistage consultation process. *BMJ*. 2017;359. [Internet]. Available from: https://www.bmj.com/content/359/bmj.j4891.long

49. De las Cuevas C, Perestelo-Perez L, Rivero-Santana A, Cebolla-Martí A, Scholl I, Härter M. Validation of the Spanish version of the 9-item Shared Decision-Making Questionnaire. *Health Expect* [Internet]. 2015;18(6):2143–53. Available from: https://pubmed.ncbi.nlm.nih.gov/24593044/

For peer review only

For peer review only

La participación de los profesionales de la salud en la Toma de Decisiones Compartida en el cribado de cáncer de mama

Manual de apoyo para implementación de la Toma de Decisiones Compartida

2021

Unión Europea
Fondo Europeo de Desarrollo Regional
"Una manera de hacer Europa"

MINISTERIO DE CIENCIA, INNOVACIÓN Y UNIVERSIDADES

Instituto de Salud Carlos III

UNIVERSITAT ROVIRA i VIRGILI

ProShare

For peer review only - http://bmjopen.bmj.com/site/about/guidelines.xhtml

CC BY NC

Guía práctica de implementación de la TDC para profesionales sanitarios

1 Crear equipo

Comunicar la necesidad de tomar una decisión

- ↘ Introduzca la posibilidad de tomar decisiones acerca de su salud
- ↘ Comente los factores de riesgo y los que le afectan en particular
- ↘ Resalte que la acompañará en todo momento y puede contar con el apoyo de familiares u otros profesionales

"Usted tiene la opción de decidir si participar o no en el programa de cribado de cáncer de mama."

"Vamos a explorar conjuntamente los beneficios y efectos adversos del programa del cribado de cáncer de mama y resolveremos las dudas que usted tenga. La acompañaré a tomar una decisión teniendo en cuenta sus preferencias y valores"

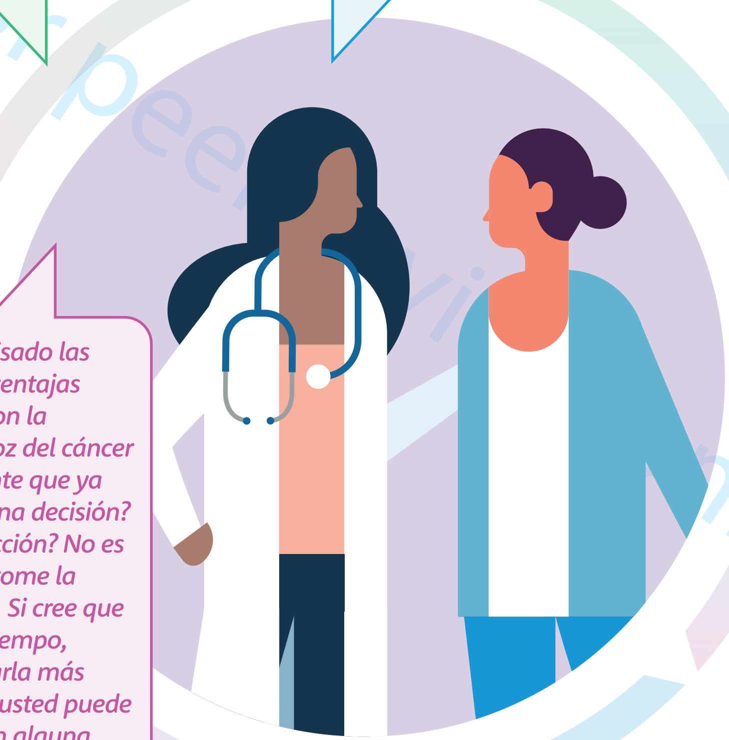

2 Plantear opciones y explorar preferencias

Informar de la opción de acudir o no a la mamografía

- ↘ Explore los conocimientos de la mujer sobre la mamografía
- ↘ Introduzca efectos adversos y beneficios de la mamografía a través de una Herramienta de Ayuda a la Toma de Decisiones (HATD)
- ↘ Considere las preferencias, creencias, valores y miedos de la mujer sobre la mamografía
- ↘ Resuma las opciones y compruebe si la mujer ha comprendido la nueva información

3 Tomar una decisión

Tomar una decisión compartida respecto a la mamografía

- ↘ Dé el tiempo necesario para permitir la reflexión
- ↘ Aclare las dudas y valore las preferencias
- ↘ Diseñe un plan de seguimiento de la decisión

"Ya hemos revisado las ventajas y desventajas relacionadas con la detección precoz del cáncer de mama ¿Siente que ya puede tomar una decisión? ¿Cuál es su elección? No es necesario que tome la decisión ahora. Si cree que necesita más tiempo, podemos tomarla más adelante y así usted puede comentarlo con alguna persona de su interés"

ProShare
SHARED DECISION MAKING IN HEALTH

Competencias Relacionales Transversales

Empatía | Escucha activa | Asertividad | Retroalimentación | Adaptación del lenguaje | Contacto visual

For peer review only - http://bmjopen.bmj.com/site/about/guidelines.xhtml

Guía práctica de implementación de la TDC para profesionales sanitarios

ProShare
SHARED DECISION MAKING IN HEALTH

Resultados

Fase de la TDC	Puntos	Interpretación
Fase 1 **"Crear equipo"**	☐	3 a 12 puntos: sin adherencia a la TDC<br>13 a 18 puntos: adherente a la TDC
Fase 2 **"Plantear opciones y explorar preferencias"**	☐	3 a 12 puntos: sin adherencia a la TDC<br>13 a 18 puntos: adherente a la TDC
Fase 3 **"Tomar una decisión"**	☐	3 a 12 puntos: sin adherencia a la TDC<br>13 a 18 puntos: adherente a la TDC
**Puntuación total:**	☐	**9 a 27:** Falta de adherencia a la TDC<br>**28 a 36:** Indiferencia a la TDC<br>**37 a 45:** Leve adherencia a la TDC<br>**46 a 54:** Fuerte adherencia a favor de la TDC

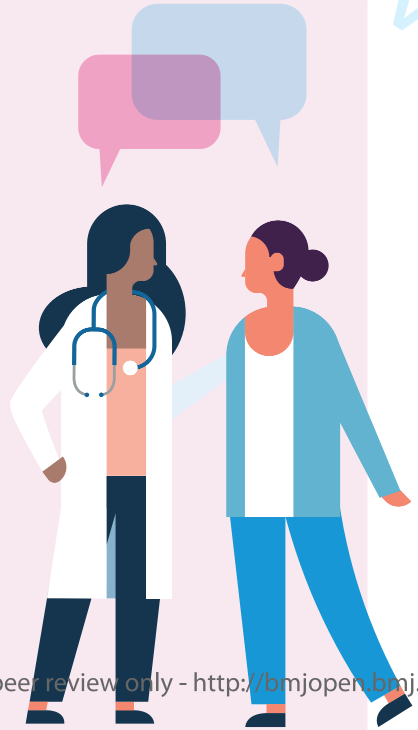

Criterio	Puntos* 1	2	3	4	5	6
Informé claramente a la mujer de la necesidad de tomar una decisión sobre su participación en el cribado de cáncer de mama	☐	☐	☐	☐	☐	☐
Pregunté a la mujer de forma precisa cómo le gustaría participar en la toma de decisiones	☐	☐	☐	☐	☐	☐
Informé a la mujer que existe la opción de participar o no en el cribado	☐	☐	☐	☐	☐	☐
Expliqué claramente a la mujer las ventajas y desventajas de cada opción	☐	☐	☐	☐	☐	☐
Ayudé a la mujer a entender toda la información sobre beneficios y efectos adversos	☐	☐	☐	☐	☐	☐
Pregunté a la mujer qué opción prefería	☐	☐	☐	☐	☐	☐
La mujer y yo hemos valorado ampliamente todas las opciones	☐	☐	☐	☐	☐	☐
La mujer y yo hemos escogido conjuntamente una opción	☐	☐	☐	☐	☐	☐
La mujer y yo nos hemos puesto de acuerdo sobre el seguimiento de su atención sanitaria posterior	☐	☐	☐	☐	☐	☐

* La puntuación va de: "Totalmente en desacuerdo" (1) a "Totalmente de acuerdo" (6)

De las Cuevas C, Perestelo-Perez L, Rivero-Santana A, Cebolla-Martí A, Scholl I, Härter M. Validation of the Spanish version of the 9-item Shared Decision-Making Questionnaire. Health Expect [Internet]. 2015;18(6):2143–53. Available from: http://www.ncbi.nlm.nih.gov/pubmed/24593044

CC BY NC

La participación de los profesionales de la salud en la Toma de Decisiones Compartida en el cribado de cáncer de mama

Manual de apoyo para implementación de la Toma de Decisiones Compartida

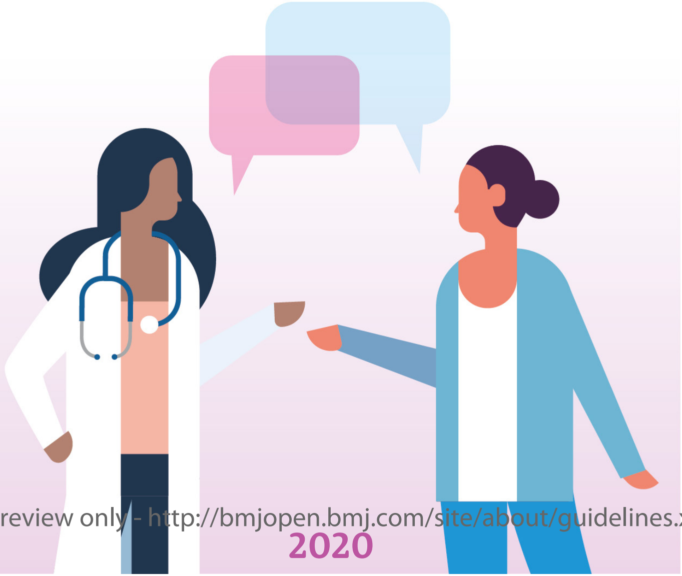

r peer review only - http://bmjopen.bmj.com/site/about/guidelines.xhtr

2020

For peer review only

La participación de los profesionales de la salud en la Toma de Decisiones Compartida en el cribado de cáncer de mama

Manual de apoyo para implementación de la Toma de Decisiones Compartida

2020

Unión Europea
Fondo Europeo de Desarrollo Regional
"Una manera de hacer Europa"
MINISTERIO DE CIENCIA, INNOVACIÓN Y UNIVERSIDADES
Instituto de Salud Carlos III
UNIVERSITAT ROVIRA i VIRGILI
ProShare
SHARED DECISION MAKING IN HEALTH

For peer review only - http://bmjopen.bmj.com/site/about/guidelines.xhtml

AUTORES

María José Hernández-Leal[1,a,b], María José Pérez-Lacasta[2,a,c], Misericòrdia Carles-Lavila[3,a,b,c] on behalf of the ProShare Group.

1. PhD student, Msc and Bsc Nursing. Mail: mariajose.hernandez@urv.cat
2. PhD, Mail: mariajose.perez@urv.cat
3. PhD. Mail: misericordia.carles@urv.cat

a. Department of Economics, Universitat Rovira i Virgili.
b. Centre de Recerca en Economia i Sostenibilitat (ECO-SOS).
c. Grup de Recerca en Anàlisi Estadístic i Econòmic en Salut (GRAEES).

Equipo de investigación ProShare:

Misericòrdia Carles Lavila
Núria Codern-Bové
María Feijoo
María José Hernández Leal
María José Pérez Lacasta
Roger Pla
Vanesa Ramos García
Carmen Vidal

Cómo citar este documento:

Hernández-Leal MJ, Carles-Lavila M, Pérez-Lacasta M. La participación de los profesionales de la salud en la Toma de Decisiones Compartida en el cribado de cáncer de mama: Manual de apoyo para implementación de la TDC. España: María José Hernández editor; 2020.

For peer review only - http://bmjopen.bmj.com/site/about/guidelines.xhtml

For peer review only

EVALUADORES EXTERNOS

Dra. Lilisbeth Perestelo-Pérez
Servicio de Evaluación y Planificación.
Dirección del Servicio Canario de la Salud, España.
Red de Investigación en Servicios de Salud en Enfermedades Crónicas (REDISSEC).

Dr. Victor Montori
Knowledge and Evaluation Research (KER) Unit.
Mayo Clinic - Minnesota, USA.

Dra. Montserrat Rué
Institut de Recerca Biomèdica de Lleida (IRBLLEIDA).
Departament de Ciències Mèdiques Bàsiques.
Universitat de Lleida, España.

DISEÑO

Miquel A. Fernández
www.mafsdisseny.com

FINANCIACIÓN

- The European Regional Development Fund (ERDF). European Union's Horizon 2020 research and innovation programme under the Marie Skłodowska-Curie grant agreement No. 713679 from the Universitat Rovira i Virgili (URV).
- Proyecto PI18/00773 “La colaboración de los profesionales sanitarios para incluir la toma de decisiones compartida en el programa de cribado de cáncer de mama” financiado por el Instituto de Salud Carlos III y cofinanciado por la Unión Europea (FEDER) “Una manera de hacer Europa”

For peer review only - http://bmjopen.bmj.com/site/about/guidelines.xhtml

For peer review only

ÍNDICE

OBJETIVO DEL MANUAL 6

¿A QUIÉN VA DIRIGIDO? 6

Introducción 7

Toma de decisiones compartida 13

1. ¿Qué es? 14

2. ¿Por qué es importante? 17

3. ¿Qué habilidades o competencias necesitan los profesionales sanitarios? 19

4. ¿Qué opinan los pacientes? 22

La toma de decisiones compartida en el cribado de cáncer de mama 25

1. El Programa de cribado 26

2. Implementación de la TDC en cribado de cáncer de mama 31

3. Autoevaluación del proceso de TDC 40

Referencias bibliográficas 45

For peer review only - http://bmjopen.bmj.com/site/about/guidelines.xhtml

OBJETIVO DEL MANUAL

Este manual pretende ser un documento de referencia que sirva de guía para el desarrollo e implementación de la **Toma de Decisiones Compartida (TDC)** en el cribado del cáncer de mama.

¿A QUIÉN VA DIRIGIDO?

A profesionales sanitarios de la comunidad autónoma de Catalunya relacionados con el cribado de cáncer de mama y que tengan contacto directo con las mujeres que deben participar en el programa.

For peer review only

Introducción

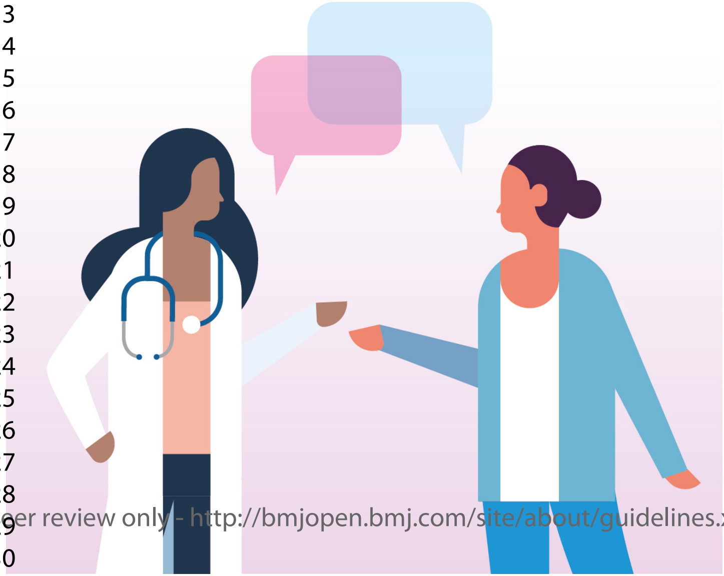

For peer review only - http://bmjopen.bmj.com/site/about/guidelines.xhtml

El objetivo del **cribado de cáncer de mama** es detectar tempranamente el tumor, en una fase preclínica. Esto permite mejorar las opciones de tratamiento y disminuir la mortalidad [1,2]. A pesar de estos beneficios, el cribado puede producir también efectos adversos: falsos negativos, falsos positivos, sobrediagnóstico y sobretratamiento [2,3,4]. Ante el desconocimiento del grado en que afectará los efectos positivos y negativos a cada mujer en la decisión del cribado, el modelo de **Toma de Decisiones Compartida** (TDC) permite que pacientes y profesionales de la salud disminuyan la incertidumbre de la decisión [3,5,6].

La TDC ha sido utilizada principalmente en los países occidentales para mejorar las decisiones en salud[7]. Así, en 2012 The European Patients' Forum inició la campaña *"nothing about me, without me"* (nada sobre mí, sin mí) [8] para involucrar a las personas en las decisiones sobre su salud [9].

Por otro lado, la **Medicina Personalizada** (MP) se ha consolidado desde la segunda mitad del siglo XX[10,11,12], centrando la atención sanitaria en la combi-

For peer review only - http://bmjopen.bmj.com/site/about/guidelines.xhtml
For peer review only

nación de la información clínica, genética y ambiental de cada persona. Al individualizar la atención, los profesionales sanitarios acceden a un enfoque integrado y basado en la evidencia para la medición de riesgo, diagnósticos, aplicación de terapias farmacológicas y manejo clínico. Estas medidas han permitido optimizar la atención clínica según las características de cada individuo[13].

La MP es un modelo de salud que está tomando cada vez más relevancia en las estrategias sanitarias en Europa, por lo que prepararse para su implementación forma parte de los desafíos actuales. La MP se complementa con la TDC ya que ambos modelos buscan posicionar a la persona en el centro de la atención, mejorar su participación en las decisiones de salud y priorizar un enfoque de atención preventiva sobre la curativa. Es decir, mientras que en la MP se utilizan los antecedentes médicos, ambientales y genéticos para estimar resultados clínicos individuales, en la TDC se consideran los valores, preferencias y vivencias de los pacientes[13].

For peer review only - http://bmjopen.bmj.com/site/about/guidelines.xhtml

La aplicación de la MP en los programas de cribado permite [10-12,14]:

- Reducir los efectos adversos (falsos positivos, falsos negativos, sobrediagnóstico y sobretratamiento.
- Aumentar la eficiencia del cribado en todos los subgrupos de riesgo de cáncer de mama.
- Ampliar el periodo y la frecuencia del cribado en las mujeres con riesgo elevado de cáncer de mama y reducir la frecuencia en mujeres con bajo riesgo.
- Informar a las mujeres sobre la detección precoz para que las decisiones que adopten estén mejor sustentadas.
- Ajustar la percepción del riesgo de padecer un cáncer de mama al riesgo real.

For peer review only

For peer review only

En este contexto, algunas investigaciones han explorado cómo se desarrolla la TDC. Martínez, et al. (2018)[15] identificaron que el 83% de los profesionales creían que las decisiones de detección deberían ser compartidas o centradas en el paciente. Sin embargo, el 77% dedicó menos de 5 minutos para discutir con ellos los beneficios y los riesgos relacionados con la mamografía[16]. Por otro lado, en España solo el 24% de los pacientes afirma haber tomado la decisión conjunta con su profesional sanitario teniendo en cuenta sus características y preferencias personales y sociales[17]. Esto evidencia la falta de estrategias para involucrar a las personas en su salud, posibles deficiencias en las habilidades comunicativas y la inexistencia de vías de diálogo productivas entre los distintos actores del encuentro clínico del cribado de mama[18].

En este sentido, la literatura ha reportado algunas barreras para la implementación de la TDC[19]: falta de tiempo en los encuentros clínicos, falta de conocimientos del profesional sanitario acerca del modelo TDC y de los efectos adversos del cribado, así como la dificultad de los pacientes para tener

For peer review only - http://bmjopen.bmj.com/site/about/guidelines.xhtml

una actuación proactiva[20,21]. La **Agencia de Calidad y Evaluación Sanitarias de Cataluña** (AQuAs) describe las Herramienta de Ayuda a la Toma de Decisiones (HATD) como un elemento central para la TDC, aunque actualmente no dispone de una para el cribado de cáncer de mama[22]. Un estudio reciente desarrolló una HATD en el cribado de cáncer de mama (anexo 1). Su evaluación posterior puso de manifiesto que los profesionales sanitarios y las mujeres valoraron positivamente el intercambio de información equilibrada del cribado mediante una HATD con el fin de mejorar la toma de decisiones en salud[23].

Sin embargo, a pesar de que en la actualidad ha habido un incremento de materiales destinados a las pacientes[24], son escasos aquellos que ayudan a los profesionales para incorporar las preferencias y los valores de las personas en la toma de decisiones en salud. En este sentido, este documento y sus anexos ofrece a los profesionales sanitarios evidencias científicas sobre la TDC para que sea aplicable al proceso de cribado de cáncer de mama.

For peer review only - http://bmjopen.bmj.com/site/about/guidelines.xhtml

Toma de decisiones compartida

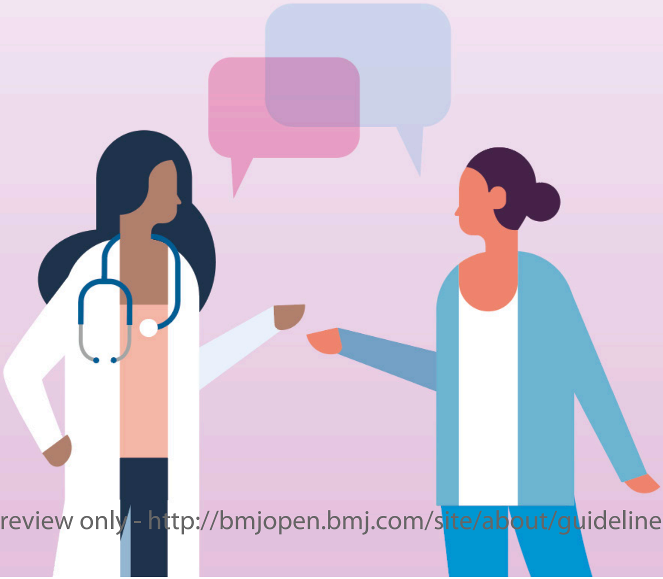

or peer review only - http://bmjopen.bmj.com/site/about/guidelines.xhtr

1. ¿Qué es?

La Toma de Decisiones Compartida (TDC) surge en la década de los 60-70 desarrollándose en mayor medida a partir de los 90. Corresponde a un modelo de atención participativa ubicada entre el estilo de atención paternalista y el informativo [25,26].

Figura 1: Modelos de atención en salud

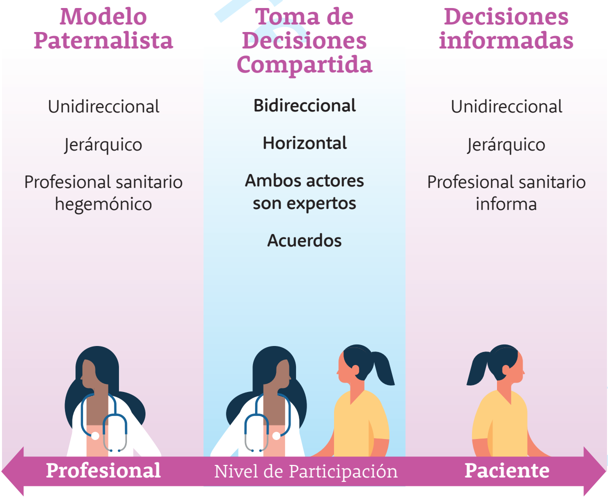

Fuente: Elaboración propia del grupo ProShare

For peer review only - http://bmjopen.bmj.com/site/about/guidelines.xhtml

La TDC fomenta la participación del paciente[27] para tomar una decisión conjunta con el profesional sanitario en relación con cambios en el estilo de vida, pruebas diagnósticas, tratamientos y acciones terapéuticas donde pueda existir algún grado de incertidumbre[28,29]. La TDC se desarrolla durante el encuentro clínico y ambos actores son considerados expertos: el paciente en su situación de salud, valores, creencias y preferencias; y el profesional sanitario en la evidencia científica e información de las opciones terapéuticas disponibles[30].

Figura 2: Rol de los participantes en el encuentro clínico

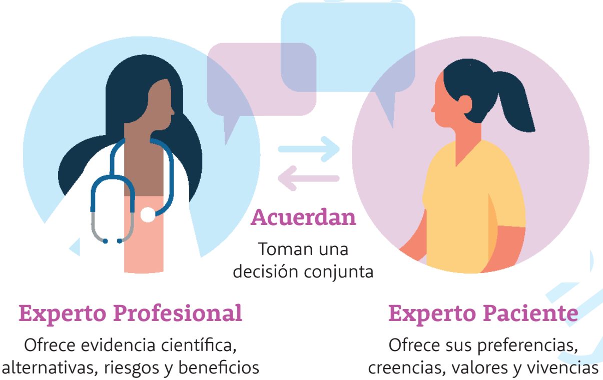

Fuente: Elaboración propia del grupo ProShare

For peer review only - http://bmjopen.bmj.com/site/about/guidelines.xhtml

Por tanto, el objetivo de la TDC se centra en garantizar que las personas tomen decisiones sobre su salud cuando están suficientemente informadas [31]. Para lograrlo se requiere de una negociación continua mediante la discusión entre ambos expertos (paciente y profesional) centrando el diálogo en los valores, preferencias, circunstancias del paciente, así cómo en los beneficios, daños, riesgos y opciones terapéuticas ofrecidas por el profesional sanitario. Como resultado final de esta discusión se consigue que las personas sean más autónomas y presenten un mayor nivel de compromiso y responsabilidad en su salud [(30,32–34)].

Figura 3: Elementos de la Toma de Decisiones Compartida

A) Intercambio de información entre el paciente y el profesional sanitario	B) Deliberación sobre las distintas opciones	**C) Tomar una decisión consensuada**

Fuente: Adaptación de Elwyn et al. 2012 [30]

For peer review only - http://bmjopen.bmj.com/site/about/guidelines.xhtml

2. ¿Por qué es importante?

La TDC se sustenta en el principio de autonomía de los pacientes. La Ley 21/2000 Derechos de Información relativos a la Salud, Autonomía del Paciente y Documentación Clínica protege su derecho a decidir libremente después de recibir la información adecuada entre las opciones clínicas disponibles [35]. Por tanto, los profesionales están sujetos jurídicamente al cumplimiento de este principio, que no puede limitarse al deseo o voluntariedad del profesional.

Asimismo, en Cataluña, el **Modelo Asistencial del Instituto Catalán de Oncología 2019-2022** promueve intervenciones centradas en la persona, teniendo como primer objetivo considerar las necesidades de las personas, para luego planificar el cuidado y atención en salud [36].

Junto con lo anterior, la implementación de la TDC ha evidenciado una serie de beneficios en los pacientes, en los profesionales y también en los sistemas sanitarios [8, 17, 34, 37, 38]:

For peer review only - http://bmjopen.bmj.com/site/about/guidelines.xhtml

- Aumenta la participación de los pacientes.
- Mejora la comunicación paciente-profesional sanitario.
- Mayor adherencia a tratamientos.
- Mejora los resultados biométricos en salud.
- Aumenta la satisfacción de los pacientes en la atención de salud.
- Disminuye la preocupación y la ansiedad en los pacientes.
- Reduce el conflicto decisional de los pacientes cuando se enfrentan a tomar una decisión diagnóstica y/o terapéutica.
- Mejora el conocimiento de la enfermedad, las opciones diagnósticas y terapéuticas en los pacientes.
- Aumenta la precisión en la percepción del riesgo.
- Aumenta la elección de opciones más beneficiosas.
- Reduce el uso de tratamientos muy invasivos y costosos.
- Reduce la variabilidad injustificada en la práctica asistencial.
- Contribuye a la racionalización del uso de recursos del sistema sanitario.

For peer review only - http://bmjopen.bmj.com/site/about/guidelines.xhtml

3. ¿Qué habilidades o competencias necesitan los profesionales sanitarios?

Comunicar de forma equilibrada los riesgos y beneficios de cualquier opción terapéutica no es tarea fácil, pero es necesario ayudar a los pacientes a estar mejor informados [37]; para conseguirlo se han identificado dos tipos de competencias que deben desarrollar los profesionales sanitarios para aplicarlas en la TDC [39]:

3.1 Competencias relacionales

Son las habilidades que favorecen una buena comunicación durante el encuentro clínico y proporcionan un ambiente cómodo que permite al paciente compartir sus preocupaciones. Para lograrlo el profesional debe tener un interés genuino en querer involucrarse y comprender el punto de vista del usuario y utilizar el tipo de lenguaje más adecuado.

For peer review only - http://bmjopen.bmj.com/site/about/guidelines.xhtml

Entre las competencias del profesional destacan:

- Realizar una escucha activa.
- Respetar las decisiones tomadas por el paciente.
- Realizar preguntas abiertas.
- Generar en todo momento contacto visual.
- Dejar que los tiempos sean pautados por el paciente.
- Reconocer sus señales emocionales o verbales.
- Usar habilidades comunicacionales: el resumen, la clarificación, el reflejo, la empatía, entre otras [40].

3.2 Competencias de comunicación de riesgo

Son las habilidades de los profesionales sanitarios que sirven para discutir con el paciente la incertidumbre que existe sobre las opciones terapéuticas y comunicar de forma efectiva los riesgos y beneficios de las diferentes opciones. Se debe evaluar la evidencia en relación con cada contexto en particular, es decir, considerar los antecedentes personales: historia familiar, historia clínica y factores de riesgo o protectores que podrían aumentar o disminuir los beneficios/daños de algunas opciones [19].

For peer review only - http://bmjopen.bmj.com/site/about/guidelines.xhtml

For peer review only

Para esto se recomienda evitar el lenguaje técnico, adaptar la cantidad de información a las necesidades actuales del paciente, utilizar diagramas, comprobar la comprensión de la información ofrecida, incorporar valores del paciente a la evidencia, transmitir información objetiva, facilitar la participación y evaluar la información de la que ya dispone el paciente [41].

Como resumen, las habilidades comunicativas necesarias para poseer una óptima relación con el paciente se pueden resumir en cinco categorías [40] (Figura 4).

Figura 4: Habilidades comunicativas

Escucha	Lenguaje	No verbal	Cultural	Actitudinal
Escucha general y activa	**Verbal:** tono apropiado y adaptado al nivel educativo<br><br>**Escrita:** comunicación clara y uso de material educativo	**Expresivo:** lenguaje corporal y contacto visual<br><br>**Receptivo:** responde a lenguaje corporal y emociones	Adaptar comunicación a la cultura, edad y enfermedad	Respetar las opiniones y derecho de decidir del paciente

Fuente: Adaptación de Laughlin T, Wetmore S, Allen T, Brailovsky C, Crichton T, Bethune C, Donoff M, Lawrence K. 2012 [illegible].

For peer review only - http://bmjopen.bmj.com/site/about/guidelines.xhtml

For peer review only

4. ¿Qué opinan los pacientes?

En un estudio desarrollado en España el 2012, usuarios de Atención Primaria con distintas patologías, valoraron una serie de elementos como positivos en la atención clínica: entrega de información, comunicación de los efectos adversos y beneficios, recomendaciones por parte del profesional, posibilidad de participar activamente en la decisión y la percepción de habilidades comunicativas básicas de los profesionales sanitarios. En este mismo estudio se identificó que al 60% de los pacientes les hubiese gustado que el profesional sanitario les pidiese su opinión, aunque no se les animó a hacerlo. Además, la mayoría hubieran deseado recibir más información de la que se les entregó [42]. En relación con la entrega de información, estudios internacionales sobre cribado de cáncer de mama han demostrado que solo entre un 8% a 10% de las mujeres recibieron información respecto al sobrediagnóstico [43].

Los pacientes sienten mayor tranquilidad, se fortalece la relación profesional sanitario-paciente

y la alianza terapéutica al participar activamente en las decisiones de su salud, ya que aumentan sus conocimientos, disminuye la incertidumbre y aumenta la posibilidad de manejar mejor su enfermedad. Además, las personas consideran que el profesional sanitario debe involucrarse en las decisiones de sus pacientes, es decir, no abandonarlos en el proceso de decisión, presentarles las opciones disponibles y brindarles asesoramiento, a pesar de que la última decisión sea la del paciente [44].

En 2013, otro estudio determinó cuáles son los elementos que más valoran los pacientes en la TDC, siendo los más significativos el rol comunicativo del profesional sanitario, la percepción de una escucha comprensiva, la sensación de una preocupación real por su salud y por sus necesidades, una conversación acorde al contexto y la constatación de un dominio de la información [45]. Finalmente, la evidencia también concluye que aún falta un largo camino de empoderamiento por el cual las personas fortalezcan su capacidad de ejercer autonomía en el autocuidado de su salud [42].

For peer review only - http://bmjopen.bmj.com/site/about/guidelines.xhtml

For peer review only

La toma de decisiones compartida en el cribado de cáncer de mama

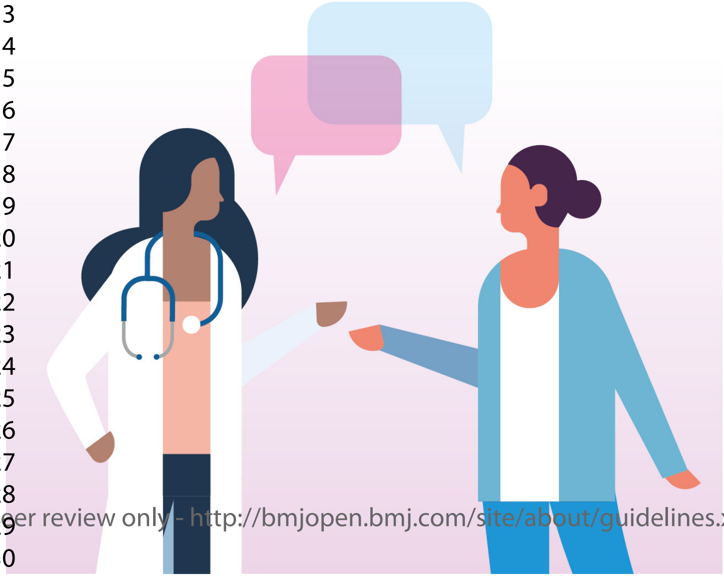

r peer review only - http://bmjopen.bmj.com/site/about/guidelines.xht

1. El Programa de cribado

1.1 Cribado poblacional en Cataluña

El Plan Estratégico del *Pla Director d'Oncologia* de la Generalitat de Catalunya menciona como objetivo disminuir el impacto del cáncer de mama en la población mediante el cribado [46]. Sin embargo, no se hace referencia a la forma de incorporar a la mujer en las decisiones de su salud, a pesar de que su participación es uno de los pilares del Plan de Salud de Cataluña 2016-2020 [47].

Actualmente, cada Programa dependiente del *Pla Director d'Oncologia* realiza, cada dos años y en base al Registro Central de Asegurados (RCA), la captación de las mujeres entre los 50 y los 69 años mediante una carta dirigida a su domicilio particular. En ella, se las invita a realizar, de forma gratuita, una mamografía en un centro de salud previamente asignado [48]. Este mecanismo no incorpora un espacio de contacto entre el profesional sanitario y la mujer, en el que ella pueda resolver sus dudas o inquietudes, ni permite

For peer review only - http://bmjopen.bmj.com/site/about/guidelines.xhtml

Figura 5: Flujograma del programa de Detección Precoz del Cáncer de Mama

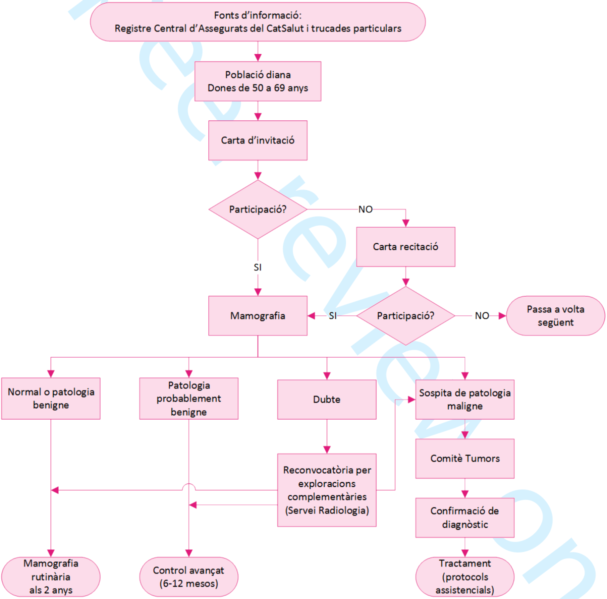

Fuente: Elaborado por Servicio de Epidemiología y Prevención del cáncer (SEPC) [49].

For peer review only - http://bmjopen.bmj.com/site/about/guidelines.xhtml

For peer review only

ofrecerle información suficiente para hacerla partícipe en la decisión sobre su participación -o no- en el programa de cribado.

Para subsanar esta falta de contacto se requiere un cambio en la organización y en los medios de información a las mujeres [49]. Así, el uso de Herramientas de Ayuda a la Toma de Decisiones (HATD) ha demostrado ser un apoyo para el profesional sanitario y para las mujeres en el momento de tomar una decisión conjunta respecto al cribado.

1.2 ¿Por qué aplicar la TDC al cribado de cáncer de mama?

Las pruebas diagnósticas para la detección precoz del cáncer de mama han tomado fuerza como estándar de salud pública al reconocerse, tanto por la comunidad científica como por las mujeres, su influencia sobre la reducción de la mortalidad en un 20% [50] y un incremento de la supervivencia al cáncer de mama que alcanza el 80% a los cinco años [24]. Sin embargo, estudios recientes demues-

or peer review only - http://bmjopen.bmj.com/site/about/guidelines.xhtr

tran que existe una escasa o nula percepción de sus daños o efectos adversos [27]. Resultados, que al menos vienen condicionados por la sensibilidad de la prueba, que pueden variar entre un 61% y el 95%, y su especificidad que varía entre el 80% al 90% [42,51].

Los principales riesgos atribuidos al cribado de cáncer de mama son los falsos positivos, falsos negativos y el sobrediagnóstico [29]. Este último concepto se define como aquellos tumores que crecen tan lentamente que nunca llegarían a ser un problema de salud e incluso desaparecerían de forma espontánea, sin necesidad de tratamiento. Actualmente, se desconoce qué tipo de lesiones progresarán y cuáles no, por tanto, se ofrece tratamiento a todas las mujeres diagnosticadas de cáncer de mama (lo que se conoce como sobretratamiento) [23] que provoca una sensación de fragilidad y vulnerabilidad en la mujer, intolerancia a la incertidumbre, grados de estrés en áreas personal-social-familiar-laboral, vinculación a procedimientos de mayor riesgo, como es el caso de las biopsias [52] y, finalmente, un aumento del gasto

For peer review only - http://bmjopen.bmj.com/site/about/guidelines.xhtml

sanitario[8]. Ante la incertidumbre entre los beneficios y efectos adversos en el cribado de cáncer de mama, se recomienda el uso de TDC con el fin de definir en base a la evidencia científica actual y los valores de las mujeres la decisión de participar o no en el cribado.

La información referente al cribado de cáncer de mama se encuentra resumida en la HATD elaborada el año 2016 (anexo 1). Allí se presenta información obtenida del *United Kingdom National Health Service (NHS)*, respecto a definiciones, incidencia, estadísticas de riesgo, beneficios y efectos adversos de una forma sencilla de comprender para el profesional sanitario y la mujer.

For peer review only

2. Implementación de la TDC en cribado de cáncer de mama

Antes de iniciar el proceso de TDC es importante construir un ambiente que permita una conversación cercana entre el profesional sanitario y la mujer. En este sentido, se debe establecer una relación de confianza basada en la empatía, para facilitar una comunicación fluida y de calidad [33,53]. El proceso debe ser deliberativo [31], es decir, las mujeres en el cribado de cáncer de mama toman conciencia de que pueden tomar una decisión, lo cual puede requerir más de un encuentro clínico [54]. Además este proceso debe ser dinámico ya que las fases deben adaptarse a las necesidades, inquietudes y prioridades de cada mujer [55].

Una vez proporcionada la información, se debe explorar explícitamente si la mujer desea desempeñar un rol activo o pasivo en la decisión [33,56] ya que si no se hace de esta forma puede inducir a un rol pasivo en las mujeres [27,33,56]. Sin embargo, no es impedimento para corroborar durante todo el encuentro clínico el rol que desean desempeñar, ya que éste

For peer review only - http://bmjopen.bmj.com/site/about/guidelines.xhtml

puede cambiar de uno activo a uno pasivo o viceversa en el transcurso de la conversación. Además, se debe considerar que existen factores que pueden aumentar o disminuir la participación de las mujeres [33]. Algunos factores, entre otros, que promueven su participación son la motivación de los profesionales de la salud para involucrar a la persona en sus decisiones, la percepción de que la TDC producirá un impacto positivo en el proceso clínico, la alta alfabetización de la mujer [57,58] o el propio deseo de ella de ser parte activa en las decisiones que afectan a su salud [37,57,59-61]. Sin embargo, la falta del tiempo en el encuentro clínico, la edad avanzada de las mujeres, personas inmigrantes y/o con dificultad de comunicarse en el idioma del profesional, el bajo nivel socioeconómico de las mujeres, su baja alfabetización [33,37,56] y la presencia de patologías de salud mental [33,52] limitan la TDC.

For peer review only

2.1 Fases y modelo *"The three-talk"*

Las tres fases que componen este modelo son [62]:

FASE 1
Crear equipo

El principal objetivo de esta fase es comunicar la necesidad de tomar una decisión en equipo, cuyos integrantes son el profesional sanitario y la mujer. Aquí se comunican los objetivos de la decisión, por qué se debe tomar (presencia de factores de riesgo personal) y las alternativas disponibles basadas en la evidencia que se deberán tener en cuenta. El profesional debe enfatizar en que (a) la mujer pude decidir no tomar una decisión en ese momento y solicitar el apoyo de otros actores como familiares u otros especialistas y (b) debe ser receptivo a las reacciones que puede generar en la mujer el enfrentarse a esta decisión. Por tanto, debe recalcar que la acompañará en el proceso hasta que se sienta segura para llevar a cabo la decisión.

For peer review only - http://bmjopen.bmj.com/site/about/guidelines.xhtml

Figura 6: Crear equipo

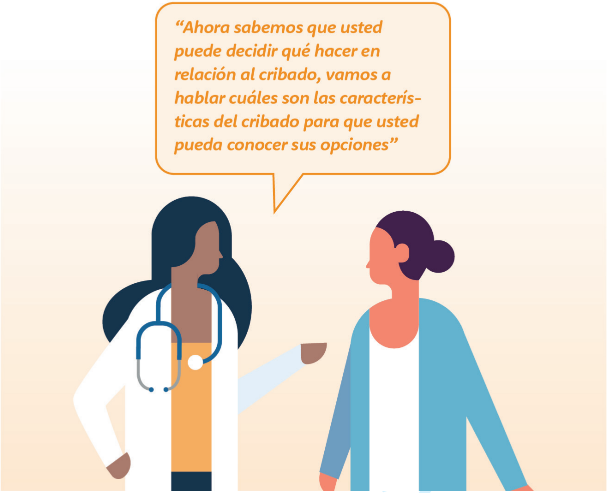

or peer review only - http://bmjopen.bmj.com/site/about/guidelines.xht

FASE 2

Plantear las opciones

El principal objetivo de esta fase es informar claramente, y según las características de cada mujer, sobre los efectos adversos y beneficios del cribado. Para esto debe explorar sus preferencias iniciales (prioridades basadas en los conocimientos preexistentes o ideas preconcebidas respecto al cribado[31]), sus valores, sus preocupaciones y expectativas y ampliar en detalle las opciones, considerando riesgos y beneficios. De esta forma, las preferencias iniciales pasarán a ser preferencias informadas (preferencias personales basadas en los valores una vez que se ha asegurado la comprensión de los riesgos y beneficios más relevantes del cribado[31]).

Para explicar los riesgos específicos se recomienda utilizar alguna HATD (anexo 1), ya que mejorarán la comprensión de la información incluso en mujeres con baja alfabetización[23]. En el contexto general, existen dos grandes categorías de HATD; las que se utilizan durante el encuentro clínico de forma con-

For peer review only - http://bmjopen.bmj.com/site/about/guidelines.xhtml

junta profesional sanitario-mujer suelen ser concisas, como los folletos (la que se proporciona en este manual). Las más extensas pueden ser utilizadas antes o después del encuentro clínico, como por ejemplo los documentos, páginas web, etc.[31]

Figura 7: Plantear las opciones

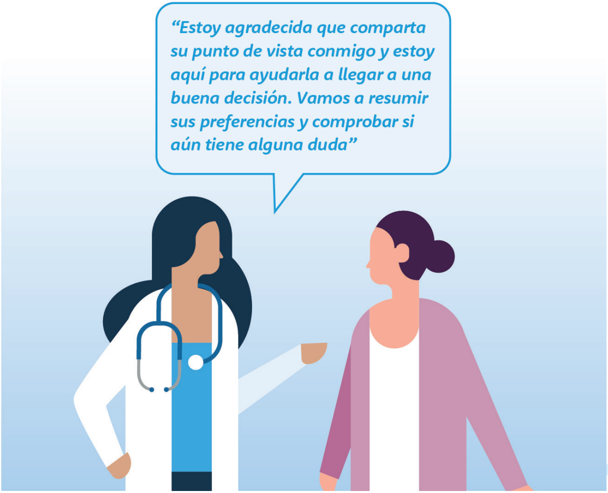

For peer review only - http://bmjopen.bmj.com/site/about/guidelines.xhtml

FASE 3

Tomar una decisión compartida

En esta fase se argumentan las alternativas y se toma una decisión respecto a la participación en el cribado de cáncer de mama [62]. El profesional debe reforzar la idea de acompañamiento en la decisión. Además, debe dar el tiempo suficiente que permita a la mujer reflexionar en torno a sus prioridades, incluso incluyendo la idea de diferirla para otro momento o delegarla en el profesional; en este último caso se recomienda identificar los elementos que le impiden hacerlo por ella misma. Finalmente, debe confirmar la decisión y acordar un plan de acción [33,56] y seguimiento que permita un circuito de retroalimentación entre el profesional y la mujer [55,56].

For peer review only - http://bmjopen.bmj.com/site/about/guidelines.xhtml

Figura 8: Tomar una decisión informada

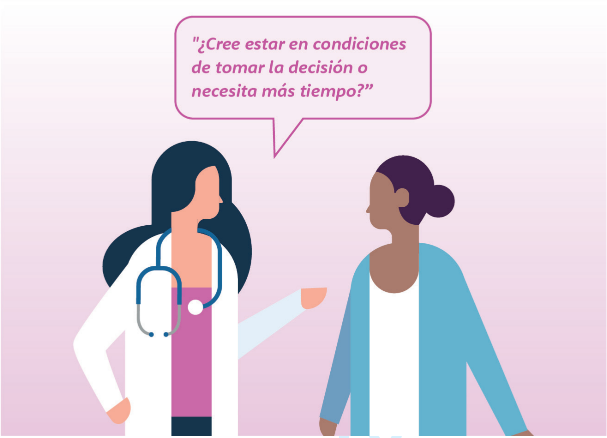

Manual de apoyo para implementación de la TDC

38

Figura 9: Fases de la Toma de Decisiones Compartida

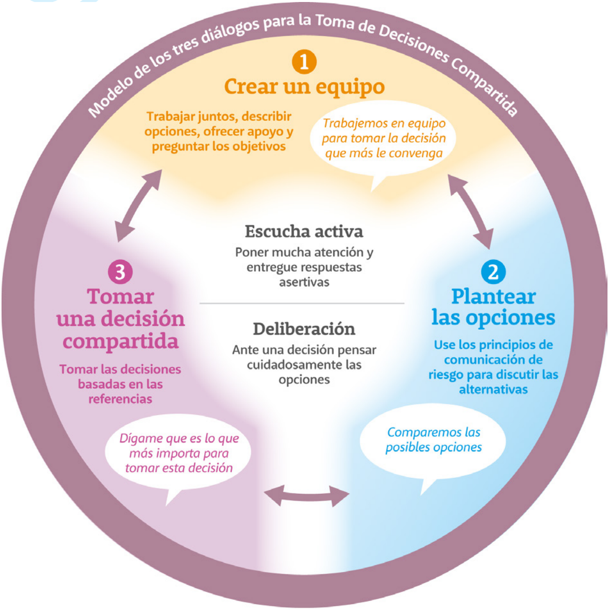

Manual de apoyo para implementación de la TDC

39

Fuente: Adaptación de Three-talk model of shared decision making. Elwyn G, et al 2017. Uso autorizado por autor[62].

3. Autoevaluación del proceso de TDC

El instrumento SDM-Q-doc [63] es una encuesta de autoevaluación para profesionales sanitarios que permite medir el nivel de participación que se le ha ofrecido a la mujer para tomar decisiones. Este instrumento está compuesto por nueve ítems que deben ser valorados por el profesional dentro de seis alternativas; desde totalmente en desacuerdo (valor 1) a totalmente de acuerdo (valor 6).

For peer review only

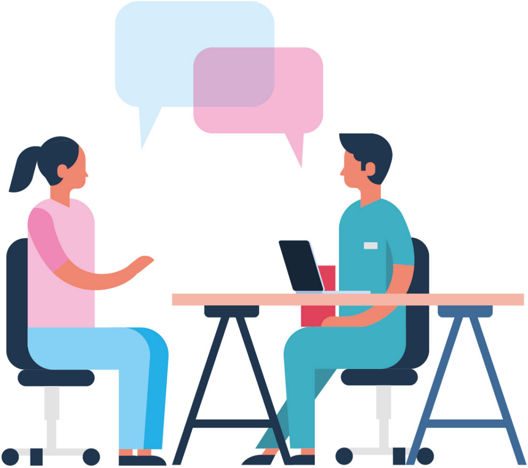

For peer review only - http://bmjopen.bmj.com/site/about/guidelines.xhtml

Criterio	Puntos* 1	2	3	4	5	6
Informé claramente a la mujer de la necesidad de tomar una decisión sobre su participación en el cribado de cáncer de mama	☐	☐	☐	☐	☐	☐
Pregunté a la mujer de forma precisa cómo le gustaría participar en la toma de decisiones	☐	☐	☐	☐	☐	☐
Informé a la mujer que existe la opción de participar o no en el cribado	☐	☐	☐	☐	☐	☐
Expliqué claramente a la mujer las ventajas y desventajas de cada opción	☐	☐	☐	☐	☐	☐
Ayudé a la mujer a entender toda la información sobre beneficios y efectos adversos	☐	☐	☐	☐	☐	☐
Pregunté a la mujer qué opción prefería	☐	☐	☐	☐	☐	☐
La mujer y yo hemos valorado ampliamente todas las opciones	☐	☐	☐	☐	☐	☐
La mujer y yo hemos escogido conjuntamente una opción	☐	☐	☐	☐	☐	☐
La mujer y yo nos hemos puesto de acuerdo sobre el seguimiento de su atención sanitaria posterior	☐	☐	☐	☐	☐	☐

* La puntuación va de: "Totalmente en desacuerdo" (1) a "Totalmente de acuerdo" (6)

Fuente: SDM-Q-doc adaptado al cribado de cáncer de mama.

For peer review only - http://bmjopen.bmj.com/site/about/guidelines.xhtml

For peer review only

Procedimiento y Resultados de la autoevaluación

Para conocer el resultado se deben sumar los puntos obtenidos en cada sección, identificando así las fases del modelo "*The three-talk*".

Fase de la TDC	Puntos	Interpretación
Fase 1 **"Crear equipo"**		13 a 18 puntos: adherente a la TDC<br>3 a 12 puntos: sin adherencia a la TDC
Fase 2 **"Plantear opciones"**		13 a 18 puntos: adherente a la TDC<br>3 a 12 puntos: sin adherencia a la TDC
Fase 3 **"Tomar una decisión"**		13 a 18 puntos: adherente a la TDC<br>3 a 12 puntos: sin adherencia a la TDC
**Puntuación total:**		**46 a 54:** Fuerte adherencia a favor de la TDC<br>**37 a 45:** Leve adherencia a la TDC<br>**28 a 36:** Indiferencia a la TDC<br>**9 a 27:** Falta de adherencia a la TDC

For peer review only - http://bmjopen.bmj.com/site/about/guidelines.xhtml

La interpretación y puntos de corte se basaron en la evidencia de Pollard, Bansback y Bryan (2015) [64]. Se dividieron los puntos totales (54 puntos) según los porcentajes de corte descritos; >80% "Fuerte adherencia del profesional a favor de la TDC"; 60-80% "Leve adherencia del profesional a la TDC"; 40-60% "Indiferencia del profesional a la TDC" y <40% "Falta de adherencia del profesional a la TDC". Por otro lado, los puntos de corte por cada fase definen como "adherente" aquellos superiores al 60% y "sin adherencia" a la TDC el resto, de un total de 15 puntos (3 al 18). Finalmente, la división por fases corresponde a una adaptación de la escala SDM-Q-doc contrastándola con las características del modelo "*Three-talk*" [61].

Se recomienda realizar esta autoevaluación con cierta periodicidad ya que permite a los profesionales sanitarios identificar los puntos fuertes y deficientes en cuanto a la forma en que incorporan la participación en salud de las mujeres. De este modo, se facilita focalizar los esfuerzos en mejorar los aspectos más débiles con formación y entrenamiento, y finalmente hacer un seguimiento de los avances conseguidos.

For peer review only - http://bmjopen.bmj.com/site/about/guidelines.xhtml

For peer review only

Referencias bibliográficas

1. Organización Mundial de la Salud. (WHO) Control del cáncer de mama; 2020 [citado 30 de junio de 2020]. Available from: https://www.who.int/topics/cancer/breastcancer/es/index3.html

2. Febles G. Revisión de los beneficios del screening del cáncer de mama y análisis de las controversias. *Rev. Imagenol.* [Internet] 2015;19 (1)9-18. Available from: https://www.webcir.org/revistavirtual/articulos/2016/4_noviembre/ur/febles_esp.pdf

3. Day N. Overdiagnosis and breast cancer screening. *Breast Cancer Res* [Internet]. 2005;7(5):228–9. Available from: http://www.ncbi.nlm.nih.gov/pubmed/16168144

4. Nehmat H. Overdiagnosis of breast cancer in population screening: does it make breast screening worthless? *Cancer Biol Med* [Internet]. 2017;14(1):1–8. Available from: http://www.cancerbiomed.org/index.php/cocr/article/view/1003

5. Pollard S, Bansback N, Bryan S. Physician attitudes toward shared decision making: A systematic review. *Patient Educ Couns* [Internet]. 2015; 98(9):1046-57. Available from: https://www.sciencedirect.com/science/article/abs/pii/S0738399115002256?via%3Dihub

6. Munthe C, Sandman L, Cutas D. Person Centred Care and Shared Decision Making: Implications for Ethics, Public Health and Research. *Health Care Anal* [Internet]. 2012;20(3):231–49. Available from: http://link.springer.com/10.1007/s10728-011-0183-y

7. Barry M, Edgman-Levitan S. Shared Decision Making — The Pinnacle of Patient-Centered Care. *N Engl J Med* [Internet]. 2012;366(9):780–1. Available from: http://www.nejm.org/doi/10.1056/NEJMp1109283

8. Coulter A, Collins A. Making shared decision-making a reality. No decision about me, without me [Internet]. The King's Fund. Edwina Rowling, editor. London; 2011. Available from: www.kingsfund.org.uk

For peer review only - http://bmjopen.bmj.com/site/about/guidelines.xhtml

9. European patients`forum. 150 Million reasons to act; *EPF's Patients' Manifesto for the European Parliament and Commission*. [Internet]. 2009. Available from: https://www.eu-patient.eu/globalassets/events/old/manifesto/epf_manifesto.pdf

10. Hernández J, Serrano O. La medicina personalizada, la revolución genómica y el Sistema Nacional de Salud. *Rev Cuba Salud Pública* [Internet]. 2014;40(4):379–91. Available from: http://scielo.sld.cu/scielo.php?pid=S0864-34662014000400012&script=sci_arttext&tlng=pt

11. Chan I, Ginsburg G. Personalized Medicine: Progress and Promise. *Annu Rev Genomics Hum Genet* [Internet]. 2011;12:217–61. Available from: https://pubmed.ncbi.nlm.nih.gov/21721939/

12. Medrano-Hernández A. Medicina personalizada: hacia un nuevo modelo en la práctica médica. *Arch Neurocien* [Internet]. 2012;17(2):129–31. Available from: https://www.medigraphic.com/pdfs/arcneu/ane-2012/ane122h.pdf

13. Sala M, Domingo L, Rué M, Comas M, Torá-Rocamora I, Macià F, et al. Investigación en cribado de cáncer de mama: camino hacia estrategias personalizadas y decisiones compartidas. *Rev Senol y Patol Mamar* [Internet]. 2014;27(4):176–82. Available from: https://linkinghub.elsevier.com/retrieve/pii/S0214158214000589

14. Edwards A, Naik G, Ahmed H, Elwyn G, Pickles T, Hood K, et al. Personalised risk communication for informed decision making about taking screening tests. *Cochrane Database Syst Rev* [Internet]. 2013:28;(2). Available from: http://doi.wiley.com/10.1002/14651858.CD001865.pub3

15. Martinez K, Deshpande A, Ruff AL, Bolen S, Teng K, Rothberg M. Are Providers Prepared to Engage Younger Women in Shared Decision-Making for Mammography? *J Womens Health* (Larchmt). 2018;27(1):24–31. Available from: http://www.liebertpub.com/doi/10.1089/jwh.2016.6047

16. Confederación Crohn y Colitis Ulcerosa. Se incumple el modelo de decisiones compartidas con los pacientes [Internet]. Available from: https://www.accuesp.com/se-incumple-el-modelo-de-decisiones-comparti-das-con-los-pacientes

For peer review only

For peer review only - http://bmjopen.bmj.com/site/about/guidelines.xhtml

17. Confederación Crohn y Colitis Ulcerosa. Se incumple el modelo de decisiones compartidas con los pacientes [Internet]. 2020 [citado 30 de junio de 2020]. Available from: https://accuesp.com/se-incumple-el-modelo-de-decisiones-compartidas-con-los-pacientes

18. Ministerio de Sanidad, Consumo y Bienestar Social de España. Marco Estratégico para la Atención Primaria y Comunitaria [Internet]. 2019. Available from: https://www.mscbs.gob.es/profesionales/proyectosActividades/docs/Marco_Estrategico_APS_25Abril_2019.pdf

19. Légaré F, Witteman H. Shared decision making: examining key elements and barriers to adoption into routine clinical practice. *Health Aff (Millwood)*. [Internet]. 2013;32(2):276-84 Available from: https://www.healthaffairs.org/doi/pdf/10.1377/hlthaff.2012.1078

20. Légaré F, Ratté S, Gravel K, Graham I. Barriers and facilitators to implementing shared decision-making in clinical practice: Update of a systematic review of health professionals' perceptions. *Patient Educ Couns* [Internet]. 2008;73(3):526–35. Available from: http://www.ncbi.nlm.nih.gov/pubmed/18752915

21. Hernández-Leal MJ, Pérez-Lacasta M, Feijoo-Cid M, Ramos-García V, Carles-Lavila M. Healthcare professionals´ behaviour to implement in shared decision making in screening programs: A systematic review. *Patient Educ Couns*. forthcoming. 2020.

22. Agencia de Calidad y Evaluación Sanitarias de Cataluña (AQuAs). Estado de situación de las herramientas de ayuda a la toma de decisiones clínicas en Cataluña. [Internet]. 2020 [citado 30 de junio de 2020]. Available from: http://aquas.gencat.cat/ca/ambits/avaluacio-tecnologies-qualitat/guies/eines_ajut_presa_decisions_catalunya/

23. Toledo-Chavarri A, Rué M, Codern-Bove N, Carles-Lavila M, Perestelo-Perez L, Perez-Lacasta MJ, and Feijoo-Cid M. A Qualitative Study on a Decision Aid for Breast Cancer Screening: Views from Women and Health Professionals. *Eur J Cancer Care (Engl)* [Internet]. 2017. 26(3). Available from: https://onlinelibrary.wiley.com/doi/abs/10.1111/ecc.12660

For peer review only - http://bmjopen.bmj.com/site/about/guidelines.xhtml

For peer review only

24. Grupo Español de Investigación en Cáncer de Mama. Guía GEICAM de Práctica Clínica: Para el Diagnóstico y Tratamiento del Cáncer de Mama Metastásico. Edición: Comuniland S.L. Madrid; 2015. Available from: https://portal.guiasalud.es/wp-content/uploads/2019/06/gpc_538_geicam_cancer_mama_resumida.pdf

25. Charles C, Gafni A, Whelan T. Self-reported use of shared decision-making among breast cancer specialists and perceived barriers and facilitators to implementing this approach. *Health Expect* [Internet]. 2004 Dec;7(4):338–48. Available from: http://www.ncbi.nlm.nih.gov/pubmed/15544686

26. Barr P, Scholl I, Bravo P, Faber M, Elwyn G, McAllister M. Assessment of Patient Empowerment - A Systematic Review of Measures. *PLoS One* [Internet]. 2015;10(5). Available from: http://journals.plos.org/plosone/article/file?id=10.1371/journal.pone.0126553&type=printable

27. Elwyn G, Edwards A, Kinnersley P, Grol R. Shared decision making and the concept of equipoise: the competences of involving patients in healthcare choice. *Br J Gen Pract* [Internet]. 2000;50:892–7. Available from: https://bjgp.org/content/bjgp/50/460/892.full.pdf

28. Participa y decide sobre tu salud. ¿Qué es la toma de decisiones compartida? [Internet]. 2020 [citado 30 de junio de 2020]. Available from: https://www.pydesalud.com/toma-de-decisiones-compartidas/

29. Marmot M, Altman D, Cameron D, Dewar J, Thompson S, Wilcox M. The benefits and harms of breast cancer screening: An independent review. *Br J Cancer* [Internet]. 2013;108(11): 2205–2240. Available from: https://www.ncbi.nlm.nih.gov/pmc/articles/PMC3693450/

30. Reyes A. Toma de decisiones compartidas: por una medicina cuidadosa y amable [Internet]. Parte 1. *Por una Medicina Interna de Alto Valor*. 2018. Available from: https://medicinainternaaltovalor.fesemi.org/el-universo-del-paciente/toma-de-decisiones-compartidas-por-una-medicina-cuidadosa-y-amable-parte-1/

31. Elwyn G, Frosch D, Thomson R, Joseph-Williams N, Lloyd A, Kinnersley P, et al. Shared decision making: a model for clinical practice. *J Gen Intern Med* [Internet]. 2012;27(10):1361–7. Available from: http://www.ncbi.nlm.nih.gov/pubmed/22618581

For peer review only - http://bmjopen.bmj.com/site/about/guidelines.xhtml

32. Peralta L, Ruiz J, Castillo R. La participación del paciente en la toma de decisiones en las consultas de atención primaria. *Universidad de Cantabria*. 2010; Available from: http://www.tesisenred.net/bitstream/handle/10803/10705/TesisLPM.pdf?sequence=1

33. Información farmacoterapéutica de la Comarca. Participación del paciente en la toma de decisiones. *INFAC* [Internet]. 2014;22(3):12–7. Available from: https://www.euskadi.eus/contenidos/informacion/cevime_infac_2014a/es_def/adjuntos/INFAC_Vol_22_n_3_Decisiones_compartidas.pdf

34. Bravo P, Contreras A. Perestelo-Pérez L, Pérez-Ramos J, Málaga G. En busca de una salud más participativa: Compartiendo decisiones de salud. *Rev Peru Med Exp Salud Publica*. 2013;30(4):691-7.

35. Boletín Oficial del Estado. Ley 14/1986 de 2 de febrero, General de Sanidad. BOE de 21/2000. [Internet]. Available from: https://www.boe.es/boe/dias/2001/02/02/pdfs/A04121-04125.pdf

36. Instituto Catalán de Oncología. 2019. Plan Estratégico 2019-2022. [Internet]. Available from: http://ico.gencat.cat/web/.content/minisite/ico/lico/documents/arxius/PLA-ESTRATEGIC-2019-2022-Original.pdf

37. Gravel K, Légaré F, Graham I. Barriers and facilitators to implementing shared decision-making in clinical practice: a systematic review of health professionals' perceptions. *Implementation Sci*. 2006;1(16). Available from: http://www.implementationscience.com/content/1/1/16

38. Légaré F, Ratté S, Stacey D, Kryworuchko J, Gravel K, Graham ID, et al. Interventions for improving the adoption of shared decision making by healthcare professionals. *Cochrane Database of Systematic Reviews* [Internet]. Chichester, UK: John Wiley & Sons, Ltd; 2010. p. CD006732. Available from: http://www.ncbi.nlm.nih.gov/pubmed/20464744

39. Légaré F, Moumjid-Ferdjaoui N, Drolet R, Stacey D, Härter M, Bastian H, et al. Core competencies for shared decision making training programs: insights from an international, interdisciplinary working group. *J Contin Educ Health Prof* [Internet]. 2013;33(4):267–73. Available from: http://www.ncbi.nlm.nih.gov/pubmed/24347105

For peer review only - http://bmjopen.bmj.com/site/about/guidelines.xhtml

40. Ford S, Schofield T, Hope T. What are the ingredients for a successful evidence-based patient choice consultation? A qualitative study. *Soc Sci Med* [Internet]. 2003;56:589–602. Available from: https://pubmed.ncbi.nlm.nih.gov/12570976/

41. Laughlin T, Wetmore S, Allen T, et al. Defining competency-based evaluation objectives in family medicine: communication skills. *Can Fam Physician* [Internet]. 2012;58(4):e217–e224. Available from: https://www.ncbi.nlm.nih.gov/pmc/articles/PMC3325474/.

42. Ruiz R, Peralta L, Pérula L, Olloqui Mundet J, Carrión T, Sobrino A, et al. Opiniones y percepciones de los pacientes sobre su participación en la toma de decisiones en las consultas de medicina de familia. *Aten Primaria* [Internet]. 2012;44(1):5–10. Available from: https://www.sciencedirect.com/science/article/pii/S0212656711001545?via%3Dihub

43. Hersch, J. Jansen & K. McCaffery (2018) Decision-making about mammographic screening: pursuing informed choice, Climacteric, 21:3, 209-213, DOI: 10.1080/13697137.2017.1406912

44. Moreau A, Carol L, Dedianne M, Dupraz C, Perdrix C, Lainé X, et al. What perceptions do patients have of decision making (DM)? Toward an integrative patient-centered care model. A qualitative study using focus-group interviews. *Patient Educ Couns* [Internet]. 2012:1;87(2):206–11. Available from: https://www.sciencedirect.com/science/article/abs/pii/S0738399111004599

45. Thorne S, Oliffe JL, Stajduhar KI. Communicating shared decision-making: Cancer patient perspectives. Patient Educ Couns [Internet]. 2013;90(3):291–6. Available from: https://www.sciencedirect.com/science/article/abs/pii/S0738399112000912

46. Generalitat de Catalunya. Departament de Salut. Pla director 2017. [Internet]. Available from: http://salutweb.gencat.cat/ca/ambits_actuacio/linies_dactuacio/estrategies_salut/cancer/pla_director/

47. Generalitat de Catalunya. Departament de Salut. Pla director D'Oncologia 2016-2020: Un sistema centrat en la persona: públic, universal i just. 2016. [Internet]. Available from: http://salutweb.gencat.cat/web/.content/_departament/pla-de-salut/Pla-de-salut-2016-2020/documents/Pla_salut_Catalunya_2016_2020.pdf

For peer review only - http://bmjopen.bmj.com/site/about/guidelines.xhtml

48. Institut Català d`Oncologia. Detecció Precoç de Càncer de Mama. [Internet]. 2020 [citado 30 de junio de 2020]. Available from: http://ico.gencat.cat/ca/el-cancer/programes_de_deteccio_precoc/programa_de_deteccio_precoc_de_cancer_de_mama/index.html

49. Servicio de epidemiología y Prevención del cáncer (SEPC). Esquema funcional del Programa de Detección Precoz del Cáncer de Mama. [Internet]. 2020 [citado 30 de junio de 2020]. Available from: https://epicancer.cat/prevencion_cancer_mama.

50. Pérez-Lacasta MJ, Martínez-Alonso M, Garcia M, Sala M, Perestelo-Pérez L, Vidal C, Corden-Bové N, Feijoo-Cid M, Toledo-Chávarri A, Cardona Á, Pons A, Carles-Lacastra M, Rué M. Effect of information about the benefits and harms of mammography on women's decision making: The InforMa randomised controlled trial. *Plos One*. [Internet]. 2019; 14(3). Available from: https://journals.plos.org/plosone/article?id=10.1371/journal.pone.0214057

51. Álvarez M, Cara M. Radiología básica de mama. Capítulo 1. Introducción al diagnóstico por la imagen de la mama. [Internet] 2015. Available from: http://www.sedim.es/nueva/wp-content/uploads/2015/01/Cap%C3%ADtulo_1_Introduccion.pdf . Córdoba, España.

52. Coll-Benejam T, Bravo-Toledo R , Marcos-Calvo M, Astier-Peña M. Impacto del sobrediagnóstico y sobretratamiento en el paciente, el sistema sanitario y la Sociedad. *Aten Primaria*. [Internet]. 2018;50(S2):86-95. Available from: https://www.elsevier.es/es-revista-atencion-primaria-27-articulo-impacto-del-sobrediagnostico-sobretratamiento-el-S0212656718305158

53. Rowland K, Politi M. Shared Decision-Making and the Patient-Provider Relationship. In: Handbook of Health Decision Science [Internet]. New York, NY: Springer New York; 2016. p. 181–92. Available from: http://link.springer.com/10.1007/978-1-4939-3486-7_14

54. Elwyn G, Frosch D, Thomson R, Joseph-Williams N, Lloyd A, Kinnersley P, et al. Shared decision making: a model for clinical practice. *J Gen Intern Med* [Internet]. 2012;27(10):1361–7. Available from: http://www.ncbi.nlm.nih.gov/pubmed/22618581

For peer review only - http://bmjopen.bmj.com/site/about/guidelines.xhtml

55. Elwyn G, Petronella Catharina Anna Vermunt N. Goal-Based Shared Decision-Making: Developing an Integrated Model. *Journal of Patient Experience*. 2019;1-9. Available from: https://journals.sagepub.com/doi/full/10.1177/2374373519878604

56. Barani M, Kopitowski K. Toma de decisiones compartidas: centrando los cuidados médicos realmente en nuestros pacientes. *Rev Hosp Ital BAires* [Internet]. 2013;33(2):60–4. Available from: https://www.hospitalitaliano.org.ar/multimedia/archivos/noticias_attachs/47/documentos/14745_60-64-HI-33-2-2BARANI_A.pdf

57. Guerra C, Jacobs S, Holmes J, Shea J. Are Physicians Discussing Prostate Cancer Screening with Their Patients and Why or Why Not? A Pilot Study. *J Gen Intern Med* [Internet]. 2007;22(7):901–7. Available from: http://www.ncbi.nlm.nih.gov/pubmed/17549576

58. Volk R, Linder S, Kallen M, Galliher J, Spano M, Mullen P, et al. Primary care physicians' use of an informed decision-making process for prostate cancer screening. *Ann Fam Med*. [Internet]. 2013;11(1):67-74. Available from: https://pubmed.ncbi.nlm.nih.gov/23319508/

59. Malli G. [Early detection of prostate cancer by PSA testing: the results of a qualitative study on barriers caused by physicians in Austria implementing informed decision making]. Gesundheitswesen [Internet]. 2013:75(1);22–8. Available from: https://www.thieme-connect.de/products/ejournals/abstract/10.1055/s-0032-1309017

60. Davis K, Haisfield L, Dorfman C, Krist A, Taylor KL. Physicians' attitudes about shared decision making for prostate cancer screening. *Fam Med* [Internet]. 2011;43(4):206-6. Available from: https://pubmed.ncbi.nlm.nih.gov/21499999/

61. Legaré F, St-Jacques S, Gagnon S, Njoya M, Brisson M,Frémont P, Rousseau F. Prenatal screening for Down syndrome: a survey of willingness in women and family physicians to engage in shared decision-making. *Prenat Diagn* [Internet]. 2011; 31:319–26. Available from: https://obgyn.onlinelibrary.wiley.com/doi/10.1002/pd.2624

For peer review only - http://bmjopen.bmj.com/site/about/guidelines.xhtml

62. Elwyn G, Duran M, Song J, Aarts J, J Barr P, Berger Z, etc al. A three-talk model for shared decision making: multistage consultation process. *BMJ*. 2017;359. [Internet]. Available from: https://www.bmj.com/content/359/bmj.j4891.long

63. De las Cuevas C, Perestelo-Perez L, Rivero-Santana A, Cebolla-Martí A, Scholl I, Härter M. Validation of the Spanish version of the 9-item Shared Decision-Making Questionnaire. *Health Expect* [Internet]. 2015;18(6):2143–53. Available from: https://pubmed.ncbi.nlm.nih.gov/24593044/

64. Pollard S, Bansback N, Bryan S. Physician attitudes toward shared decision making: A systematic review. *Patient Educ Couns* [Internet]. 2015; 98(9):1046-57. Available from: https://www.sciencedirect.com/science/article/abs/pii/S0738399115002256?via%3Dihub

For peer review only

For peer review only - http://bmjopen.bmj.com/site/about/guidelines.xhtml

La participación de los profesionales de la salud en la Toma de Decisiones Compartida en el cribado de cáncer de mama

Manual de apoyo para implementación de la Toma de Decisiones Compartida

2020

Unión Europea
Fondo Europeo de Desarrollo Regional
"Una manera de hacer Europa"
MINISTERIO DE CIENCIA, INNOVACIÓN Y UNIVERSIDADES
Instituto de Salud Carlos III
UNIVERSITAT ROVIRA i VIRGILI
ProShare
SHARED DECISION MAKING IN HEALTH

For peer review only - http://bmjopen.bmj.com/site/about/guidelines.xhtml

Guía práctica de implementación de la TDC para profesionales sanitarios

ProShare
SHARED DECISION MAKING IN HEALTH

"Ahora sabemos que usted puede decidir qué hacer en relación al cribado, vamos a hablar cuáles son las característi-cas del cribado para que usted pueda conocer sus opciones"

Comunicar la necesidad de tomar una decisión

1 Crear equipo

- Introduzca la posibilidad de tomar decisiones acerca de su salud
- Comente los factores de riesgo y los que le afectan en particular
- Resalte que la acompañará en todo momento y puede contar con el apoyo de familiares u otros profesionales

Factores de Riesgo Se estima que 1 de cada 8 mujeres en España padecerán cáncer de mamá a lo largo de su vida. Los factores de riesgo son: edad, antecedentes familiares y personales de cáncer de mama, alteraciones de la mama, hormonal, radioterapia y estilo de vida.

Fuente: AECC: https://www.aecc.es/es/ todo-sobre-cancer/tipos-cancer/cancer-mama/mas-informacion/evolucion-cancer-mama

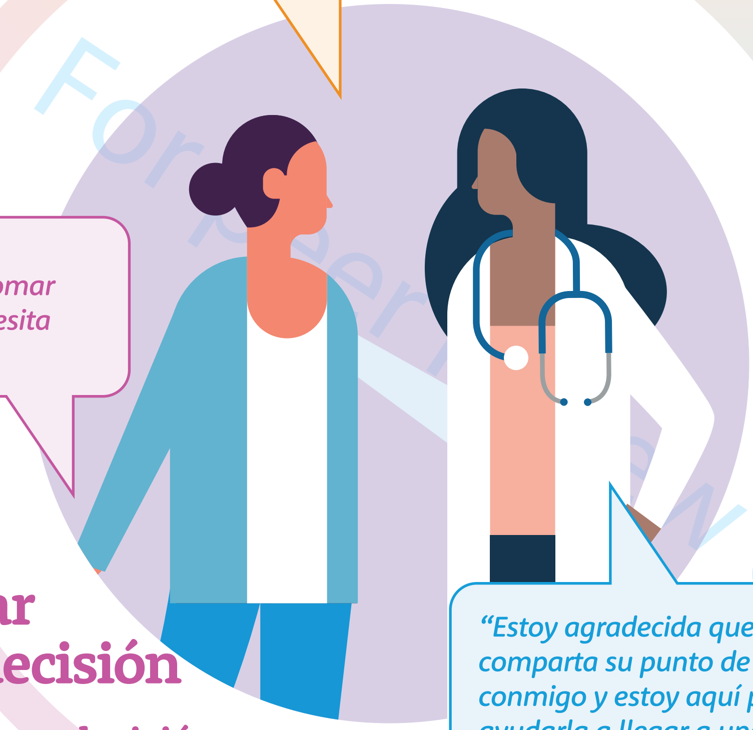

"¿Cree estar en condiciones de tomar la decisión o necesita más tiempo?"

2 Plantear las opciones

Informar de la opción de acudir o no a la mamografía

- Explore los conocimientos de la mujer sobre la mamografía
- Introduzca efectos adversos y beneficios de la mamografía a través de una Herramienta de Ayuda a la Toma de Decisiones (HATD)
- Considere las preferencias, creencias, valores y miedos de la mujer sobre la mamografía
- Resuma las opciones y compruebe si la mujer ha comprendido la nueva información

"Estoy agradecida que comparta su punto de vista conmigo y estoy aquí para ayudarla a llegar a una buena decisión. Vamos a resumir sus preferencias y comprobar si aún tiene alguna duda"

3 Tomar una decisión

Tomar una decisión compartida respecto a la mamografía

- Dé el tiempo necesario para permitir la reflexión
- Aclare las dudas y valore las preferencias
- Diseñe un plan de seguimiento de la decisión

La Toma de Decisiones Comparti-das (TDC) es un modelo de atención participativo ubicado entre un estilo de atención paternalista e informa-tivo que fomenta la participación de las mujeres para tomar una decisión con el profesional sanitario cuando existe algún grado de incertidum-bre. La TDC se desarrolla durante el encuentro clínico, ambos actores se consideran como expertos: la mujer en su situación de salud, valores, creencias y preferencias. El profesio-nal en la evidencia científica y en cómo dar información de las opcio-nes terapéuticas disponibles.

La mamografía es utilizada como *gold stándar* para el cribado a nivel internacional; ha demostrado disminuir del 20 al 30% la mortalidad, pero también genera falsos negativos, falsos positivos y sobrediagnóstico; su función es adelantar el diagnóstico sin esperar que aparezcan síntomas de la enfermedad. La mamografía es una radiografía de dos proyecciones (cráneo-caudal y látero-oblicua) en cada pecho que posteriormente se analiza con una doble lectura (anexo 1: HATD).

For peer review only - http://bmjopen.bmj.com/site/about/guidelines.xhtml

Guía práctica de implementación de la TDC para profesionales sanitarios

ProShare
SHARED DECISION MAKING IN HEALTH

Resultados

Fase de la TDC	Puntos	Interpretación
Fase 1 **“Crear equipo”**	☐	13 a 18 puntos: adherente a la TDC<br>3 a 12 puntos: sin adherencia a la TDC
Fase 2 **“Plantear opciones”**	☐	13 a 18 puntos: adherente a la TDC<br>3 a 12 puntos: sin adherencia a la TDC
Fase 3 **“Tomar una decisión”**	☐	13 a 18 puntos: adherente a la TDC<br>3 a 12 puntos: sin adherencia a la TDC
**Puntuación total:**	☐	**46 a 54:** Fuerte adherencia a favor de la TDC<br>**37 a 45:** Leve adherencia a la TDC<br>**28 a 36:** Indiferencia a la TDC<br>**9 a 27:** Falta de adherencia a la TDC

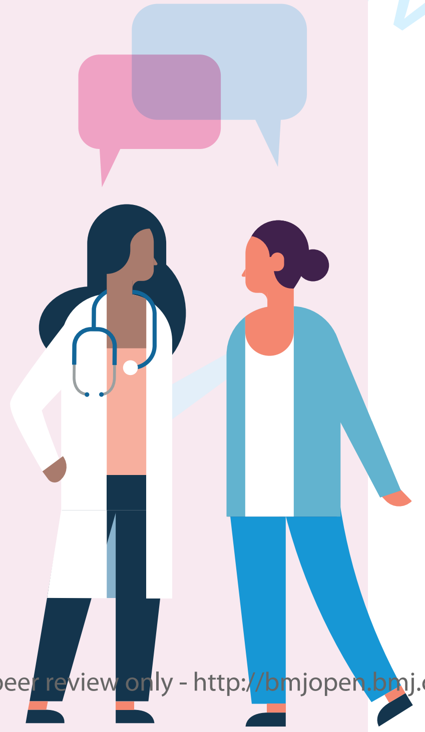

Criterio	Puntos* 1	2	3	4	5	6
Informé claramente a la mujer de la necesidad de tomar una decisión sobre su participación en el cribado de cáncer de mama	☐	☐	☐	☐	☐	☐
Pregunté a la mujer de forma precisa cómo le gustaría participar en la toma de decisiones	☐	☐	☐	☐	☐	☐
Informé a la mujer que existe la opción de participar o no en el cribado	☐	☐	☐	☐	☐	☐
Expliqué claramente a la mujer las ventajas y desventajas de cada opción	☐	☐	☐	☐	☐	☐
Ayudé a la mujer a entender toda la información sobre beneficios y efectos adversos	☐	☐	☐	☐	☐	☐
Pregunté a la mujer qué opción prefería	☐	☐	☐	☐	☐	☐
La mujer y yo hemos valorado ampliamente todas las opciones	☐	☐	☐	☐	☐	☐
La mujer y yo hemos escogido conjuntamente una opción	☐	☐	☐	☐	☐	☐
La mujer y yo nos hemos puesto de acuerdo sobre el seguimiento de su atención sanitaria posterior	☐	☐	☐	☐	☐	☐

* La puntuación va de: “Totalmente en desacuerdo” (1) a “Totalmente de acuerdo” (6)

De las Cuevas C, Perestelo-Perez L, Rivero-Santana A, Cebolla-Martí A, Scholl I, Härter M. Validation of the Spanish version of the 9-item Shared Decision-Making Questionnaire. Health Expect [Internet]. 2015;18(6):2143–53. Available from: http://www.ncbi.nlm.nih.gov/pubmed/24593044

For peer review only - http://bmjopen.bmj.com/site/about/guidelines.xhtml

TABLES

Table 1: Characteristics of the participants

Variable		Round 1		Round 2		Round 3	
		N	%	N	%	N	%
Sex	Female	15	75	12	75	13	76.47
	Male	5	25	4	25	4	23.52
	Total	20	100	16	100	17	100
Age range (years)	31-40	7	35	7	43.75	7	41.17
	41-50	6	30	4	25	5	29.41
	51-60	5	25	4	25	4	23.52
	61-70	2	10	1	6.25	1	5.88
	Total	20	100	16	100	17	100
Ownership of the affiliated institute, health centre or research site	Public sector	14	70	11	68.75	11	64.7
	Private sector	6	30	5	31.25	6	35.29
	Total	20	100	16	100	17	100
Profession	Nursing	4	20	2	12.5	3	17.64
	Medicine	13	65	11	68.75	11	64.7
	Psychology	1	5	1	6.25	1	5.88
	Other	2	10	2	12.5	2	11.76
	Total	20	100	16	100	17	100
Specialty	Family and community medicine or nursing	14	70	11	68.75	12	70.58
	Public health	1	5	1	6.25	1	5.88
	Gynaecology	1	5	1	6.25	1	5.88
	Endocrinology	1	5	1	6.25	1	5.88
	Research in health services	1	5	1	6.25	1	5.88
	Content development for Decision Support Systems for Healthcare	1	5	1	6.25	1	5.88
	None	1	5	0	0	0	0
	Total	20	100	16	100	17	100
Experience (years)	6-10	6	30	6	37.5	6	35.29
	11-20	6	30	5	31.25	6	35.29
	21-30	6	30	5	31.25	5	29.41
	31-40	2	10	0	0	0	0
	Total	20	100	16	100	17	100

For peer review only

Development of support material for health professionals who are implementing Shared Decision-Making in breast cancer screening: Validation using Delphi methodology

Support material for SDM in breast cancer screening

Table 2: R1 responses

Section	Questions using a Likert scale of 1 (Completely disagree) to 6 (Completely agree)	1	2	3	4	5	6	Cc*
Evaluation of the Handbook on Shared Decision-Making in Breast Cancer Screening	1. The sections of the handbook are effective for understanding the application of SDM to breast cancer screening	0	5	5	10	55	25	90
	4. The 'Contents' section is suitable for this handbook	0	5	5	5	35	50	90
	5. The 'Objective of the material' section is suitable for this handbook	0	5	0	5	30	60	95
	6. The 'Who is it aimed at?' section is suitable for this handbook	0	5	0	10	35	50	95
	7. The 'Introduction' section is suitable for this handbook	5	10	0	10	45	30	85
	8. The 'Shared Decision-Making: What is it?' section is suitable for this handbook	0	5	5	10	40	40	90
	9. The 'Shared Decision-Making: Why is it important?' section is suitable for this handbook	0	5	0	5	45	45	95
	10. The 'Shared Decision-Making: 'What skills or competencies do health professionals need?' section is suitable for this handbook	0	5	5	35	35	20	90
	11. The 'Shared Decision-Making: What do patients think?' section is suitable for this handbook	0	10	0	10	35	45	90
	12. The 'Shared Decision-Making in breast cancer screening: The screening programme' section is suitable for this handbook	0	10	10	5	30	45	80
	13. The 'Shared Decision-Making in breast cancer screening: Implementation of SDM in breast cancer screening' section is suitable for this handbook	0	5	0	5	45	45	95
	14. The 'Shared Decision-Making in breast cancer screening: Self-assessment of the SDM process' section is suitable for this handbook	0	10	10	10	35	35	80
	15. The handbook provides the minimum content on SDM in breast cancer screening that health professionals should be familiar with	0	5	0	20	50	25	95
	16. The content of the handbook is sufficiently detailed	0	5	5	5	35	50	90
	19.a. Figure 1: Models of healthcare (page 14) is useful	0	0	10	15	30	45	90
	19.b. Figure 1: Models of healthcare (page 14) is clear	0	0	15	10	20	55	85
	20.a. Figure 2: Role of the participants in the clinical encounter (page 15) is useful	0	5	5	10	35	45	90
	20.b. Figure 2: Role of the participants in the clinical encounter (page 15) is clear	0	5	5	15	30	45	90
	21.a. Figure 3: Elements of Shared Decision-Making (page 16) is useful	0	0	10	20	25	45	90
	21.b. Figure 3: Elements of Shared Decision-Making (page 16) is clear	0	0	5	20	25	50	95
	22.a. Figure 4: Communication skills (page 21) is useful	5	0	10	30	15	40	85
	22.b. Figure 4: Communication skills (page 21) is clear	0	0	10	20	25	45	90
	23.a. Figure 5: Flow diagram of the Early Detection of Breast Cancer programme (page 27) is useful	5	5	20	15	20	35	70
	23.b. Figure 5: Flow diagram of the Early Detection of Breast Cancer programme (page 27) is clear	10	10	20	15	15	30	60
	24.a. Figure 6: Team talk (page 34) is useful	10	5	0	30	25	30	85
	24.b. Figure 6: Team talk (page 34) is clear	10	0	15	20	25	30	75
	26.a. Figure 7: Option talk (page 36) is useful	5	5	0	30	40	20	90
	26.b. Figure 7: Option talk (page 36) is clear	5	0	10	45	15	25	85
	28.a. Figure 8: Decision talk (page 38) is useful	0	5	10	5	35	45	80

Development of support material for health professionals who are implementing Shared Decision-Making in breast cancer screening: Validation using Delphi methodology

Support material for SDM in breast cancer screening

<table>
<tr><td rowspan="19"></td><td colspan="2">28.b. Figure 8: Decision talk (page 38) is clear</td><td>0</td><td>0</td><td>5</td><td>15</td><td>30</td><td>50</td><td>95</td></tr>
<tr><td colspan="2">30.a. Figure 9: Shared Decision-Making steps (page 39) is useful</td><td>0</td><td>0</td><td>5</td><td>20</td><td>25</td><td>50</td><td>95</td></tr>
<tr><td colspan="2">30.b. Figure 9: Shared Decision-Making steps (page 39) is clear</td><td>0</td><td>0</td><td>10</td><td>35</td><td>10</td><td>45</td><td>90</td></tr>
<tr><td colspan="2">31. Does its design (colours, images) make the handbook easier to read for an SDM professional?</td><td>0</td><td>0</td><td>5</td><td>20</td><td>35</td><td>40</td><td>95</td></tr>
<tr><th>Closed questions</th><th colspan="6">Options</th><th colspan="2">Percentage (%)</th></tr>
<tr><td rowspan="14">2. Which section of the handbook do you think should be changed?</td><td colspan="6">a) Front cover</td><td colspan="2">0</td></tr>
<tr><td colspan="6">b) Objective of the material</td><td colspan="2">0</td></tr>
<tr><td colspan="6">c) Who is it aimed at?</td><td colspan="2">0</td></tr>
<tr><td colspan="6">d) Introduction</td><td colspan="2">10</td></tr>
<tr><td colspan="6">e) Shared Decision-Making: What is it?</td><td colspan="2">0</td></tr>
<tr><td colspan="6">f) Shared Decision-Making: Why is it important?</td><td colspan="2">0</td></tr>
<tr><td colspan="6">g) Shared Decision-Making: What skills or competencies do health professionals need?</td><td colspan="2">25</td></tr>
<tr><td colspan="6">h) Shared Decision-Making: What do patients think?</td><td colspan="2">0</td></tr>
<tr><td colspan="6">i) Shared Decision-Making in breast cancer screening: The screening programme</td><td colspan="2">15</td></tr>
<tr><td colspan="6">j) Shared Decision-Making in breast cancer screening: Implementation of SDM in breast cancer screening</td><td colspan="2">0</td></tr>
<tr><td colspan="6">k) Shared Decision-Making in breast cancer screening: Self-assessment of the SDM process</td><td colspan="2">0</td></tr>
<tr><td colspan="6">l) None</td><td colspan="2">50</td></tr>
<tr><td colspan="6">Total</td><td colspan="2">100</td></tr>
<tr><td rowspan="3">Evaluation of the Clinical Practice Guide: Implementation of SDM for Health Professionals</td><th colspan="2">Questions using a Likert scale of 1 (Completely disagree) to 6 (Completely agree)</th><th>1</th><th>2</th><th>3</th><th>4</th><th>5</th><th>6</th><th>Cc*</th></tr>
<tr><td colspan="2">1. Do you think that a clinical practice guide concisely summarising the SDM steps is necessary?</td><td>0</td><td>15</td><td>10</td><td>5</td><td>5</td><td>65</td><td>75</td></tr>
<tr><td colspan="2">6. Is it useful to incorporate the Self-assessment section in the clinical practice guide?</td><td>0</td><td>5</td><td>10</td><td>25</td><td>25</td><td>35</td><td>85</td></tr>
</table>

$*Cc = \left(1 - \frac{Vn}{Vt}\right)x100$

For consensus, a Coefficient of Concordance (Cc) >75 was used. Vn = Number of negative votes (score of less than 4); Vt = Total number of votes (n=6)[26].

For peer review only

For peer review only - http://bmjopen.bmj.com/site/about/guidelines.xhtml

Development of support material for health professionals who are implementing Shared Decision-Making in breast cancer screening: Validation using Delphi methodology

Support material for SDM in breast cancer screening

Table 3: R2 responses

Section	Questions using a Likert scale of 1 (Completely disagree) to 6 (Completely agree)	1	2	3	4	5	6	CC*
Evaluation of the Handbook on Shared Decision-Making in Breast Cancer Screening	1. Eliminate Figure 5: Flow diagram of the Early Detection of Breast Cancer Programme (page 27)	6.3	18.8	18.8	6.3	43.8	6.3	56.4
	2. Shorten content: the handbook format is very long	0	12.5	6.3	18.8	25	37.5	81.3
	4. Incorporate more examples of dialogues between the professional and the woman into each phase	18.8	6.3	6.3	31.3	31.3	6.3	68.9
	5. Add information on communication skills and competencies resources	0	12.5	25	12.5	43.8	6.3	62.6
	6. Add information on joint responsibility for the shared decision-making agreement	6.3	31.3	12.5	0	37.5	12.5	50
	7. Add information about resources on using the Patients Decision Aids (PtDAs). Note that this tool is intended to be used with the women	0	18.8	12.5	18.8	18.8	31.3	68.9
	8. Add information on the limitations of the SDM model	6.3	18.8	25	12.5	25	12.5	50
	9. Provide example dialogues on exploring the women's values, beliefs and preferences	0	18.8	12.5	18.8	31.3	18.8	68.9

Section	Closed questions	Options	Percentage (%)
Evaluation of the Handbook on Shared Decision-Making in Breast Cancer Screening	3. Which element of the handbook would you shorten?	a) Objective of the material	0
		b) Who is it aimed at?	0
		c) Introduction	50
		d) Shared Decision-Making: What is it?	0
		e) Shared Decision-Making: Why is it important?	0
		f) Shared Decision-Making: What skills or competencies do health professionals need?	0
		g) Shared Decision-Making: What do patients think?	6.3
		h) Shared Decision-Making in breast cancer screening: The screening programme	6.3
		i) Shared Decision-Making in breast cancer screening: Implementation of SDM in breast cancer screening	6.3
		j) Shared Decision-Making in breast cancer screening: Self-assessment of the SDM process	0
		k) None	31.3
		Total	100
	10. Change the name of phase 2	a) Option talk (current name)	18.8
		b) Option talk and exploring preferences (proposal)	81.3
		c) Other	0
		Total	100
	12. Phase 1 dialogue: Team Talk (page 34):	a) Now that we know that you can decide what to do about screening, we're going to talk about the characteristics of screening, so that you know what your options are (current dialogue).	12.5
		a) You have the option of deciding whether or not to have early-detection tests for breast cancer. If you're happy to, we can explore together what risks and benefits the test involves for you (proposal).	81.3
		c) Other	6.2
		Total	100

	14. Phase 2 dialogue: Option Talk (page 36)	a) I appreciate you sharing your views with me and I'm here to help you come to a good decision. Let's do a recap of your preferences and check whether you have any more questions (current dialogue).	18.8
		B) I'm here to help you make a decision. Let's look at what your preferences are and the various options available, and we'll check whether you have any questions about them (proposal).	75
		c) Other	6.2
		Total	100
	16. Phase 3 dialogue: Decision Talk (page 38):	a) Do you think that you're ready to make the decision or do you need more time? (current dialogue).	12.5
		b) Now that we've gone over the advantages and disadvantages of early detection, do you think that you can make the decision? Bear in mind that this can be delayed if you need more time or to talk about it with someone of your choice (proposal).	81.3
		c) Other	6.2
		Total	100

	Questions using a Likert scale of 1 (Completely disagree) to 6 (Completely agree)	1	2	3	4	5	6	Cc*
Evaluation of the Clinical Practice Guide: Implementation of SDM for Health Professionals	1. A clinical practice guide is necessary for this handbook	6.3	0	12.5	25	31.3	25	81.3
	2. Improve the design of the clinical practice guide to improve understanding (colour, structure, etc.)	6.3	0	18.8	37.5	25	12.5	75
	3. Eliminate additional information (definitions of Risk factors, Mammography, Shared Decision-Making)	6.3	18.8	18.8	6.3	18.8	31.3	56.4
	4. Mention the possibility of reversing the decision in the follow-up plan	6.3	0	6.3	18.8	25	43.8	87.6
	5. Mention relationship-building competencies: active listening, showing empathy, clarification, etc.	12.5	6.3	12.5	6.3	43.8	18.8	68.9

$*Cc = \left(1 - \frac{Vn}{Vt}\right)x100$

For consensus, a Coefficient of Concordance (Cc) >75 was used. Vn = Number of negative votes (score of less than 4); Vt = Total number of votes (n=6)[21].

For peer review only

Table 4: R3 responses

Section	Closed questions	Options	Percentage (%)
Evaluation of the Handbook on Shared Decision-Making in Breast Cancer Screening	1. Given that no consensus has been reached (56.4 %) on whether or not to eliminate Figure 5: Flow diagram of the Early Detection of Breast Cancer programme (page 27), please select one of the following options:	a) Eliminate. It does not add relevant information to this handbook	47.1
		b) Keep. Translate to Spanish and improve the image resolution	52.9
		Total	
	2. Given that there is no consensus (68.9 %) about whether to add more examples of dialogues between the professional and the women for each phase, please select one of the following options:	a) One example per phase (current format)	35.3
		b) Three examples per phase (proposed new format) The image will be adapted to a more readable size for the handbook	64.7
		Total	100
	3. Given that there is no consensus (62.6 %) about whether to add information on communication skills and competencies resources to the handbook, please select one of the following options:	a) Yes, it is necessary to incorporate bibliographic references into the handbook for those who would like to find out more about this topic.	58.8
		b) No, the handbook is already too long to add more information.	64.7
		Total	100
	4. Given that there is no consensus (50 %) about whether to include information on joint responsibility for the SDM agreement, please select one of the following options:	a) Yes, it should be included because the information is not clear	41.2
		b) It is not necessary, it is already clear that the responsibility is shared	58.8
		Total	100
	5. Given that there is no consensus (68.9 %) about whether bibliographic references should be added on the PtDAs– note that the PtDAs is an appendix to the handbook, to be used by the woman and health professional – please select one of the following options:	a) Yes, they should be added	52.9
		b) No, this is not necessary	47.1
		Total	100
	6. Given that there is no consensus (50 %) about whether to add information on the limitations of the model, please select one of the following options:	a) Yes, this is necessary because not doing so would mean producing one-sided material	58.8
		b) No, it is not necessary because the objective of the handbook is to show the advantages of implementing it	41.2
		Total	100
Evaluation of the Clinical Practice Guide: Implementation of SDM for Health Professionals	1. Given that there is no consensus about the design and content of the guide, please select one of the following options. The infographic will be adapted to a more readable size for the guide.	a) Current format	23.5
		b) Proposed new format	76.5
		Total	100

For peer review only

Development of support material for health professionals who are implementing Shared Decision-Making in breast cancer screening: Validation using Delphi methodology

Revised Standards for Quality Improvement Reporting Excellence (SQUIRE 2.0)

Text Section and Item Name	Section or Item Description	Chek
	**Title and Abstract**	
1. Title	Indicate that the manuscript concerns an initiative to improve healthcare (broadly defined to include the quality, safety, effectiveness, patient- centeredness, timeliness, cost, efficiency, and equity of healthcare)	X
2. Abstract	a. Provide adequate information to aid in searching and indexing<br>b. Summarize all key information from various sections of the text using the abstract format of the intended publication or a structured summary such as: background, local problem, methods, interventions, results, conclusions	X
**Introduction**	**Why did you start?**	
3. Problem Description	Nature and significance of the local problem	X
4. Available knowledge	Summary of what is currently known about the problem, including relevant previous studies	X
5. Rationale	Informal or formal frameworks, models, concepts, and/or theories used to explain the problem, any reasons or assumptions that were used to develop the intervention(s), and reasons why the intervention(s) was expected to work	X
6. Specific aims	Purpose of the project and of this report	X
**Methods**	**What did you do?**	

Development of support material for health professionals who are implementing Shared Decision-Making in breast cancer screening: Validation using Delphi methodology

7. Context	Contextual elements considered important at the outset of introducing the intervention(s)	**X**
8. Intervention(s)	a. Description of the intervention(s) in sufficient detail that others could reproduce it<br>b. Specifics of the team involved in the work	**X**
9. Study of the Intervention(s)	a. Approach chosen for assessing the impact of the intervention(s)<br>b. Approach used to establish whether the observed outcomes were due to the intervention(s)	**X**
10. Measures	a. Measures chosen for studying processes and outcomes of the intervention(s), including rationale for choosing them, their operational definitions, and their validity and reliability<br>b. Description of the approach to the ongoing assessment of contextual elements that contributed to the success, failure, efficiency, and cost<br>c. Methods employed for assessing completeness and accuracy of data.	**X**
11. Analysis	a. Qualitative and quantitative methods used to draw inferences from the data<br>b. Methods for understanding variation within the data, including the effects of time as a variable	**X**
12. Ethical Considerations	Ethical aspects of implementing and studying the intervention(s) and how they were addressed, including, but not limited to, formal ethics review and potential conflict(s) of interest	**X**
**Results**	**What did you find?**	
13. Results	a. Initial steps of the intervention(s) and their evolution over time (e.g., time-line diagram, flow chart, or table), including modifications made to the intervention during the project<br>b. Details of the process measures and outcome<br>c. Contextual elements that interacted with the intervention(s)<br>d. Observed associations between outcomes, interventions, and relevant contextual elements<br>e. Unintended consequences such as unexpected benefits, problems, failures, or costs associated with the intervention(s).<br>f. Details about missing data	X

Development of support material for health professionals who are implementing Shared Decision-Making in breast cancer screening: Validation using Delphi methodology

**Discussion**	**What does it mean?**	
14. Summary	a. Key findings, including relevance to the rationale and specific aims<br>b. Particular strengths of the project	**X**
15. Interpretation	a. Nature of the association between the intervention(s) and the outcomes<br>b. Comparison of results with findings from other publications<br>c. Impact of the project on people and systems	**X**
16. Limitations	a. Limits to the generalizability of the work<br>b. Factors that might have limited internal validity such as confounding, bias, or imprecision in the design, methods, measurement, or analysis c. Efforts made to minimize and adjust for limitations.	**X**
17. Conclusions	a. Usefulness of the work<br>b. Sustainability<br>c. Potential for spread to other contexts<br>d. Implications for practice and for further study in the field<br>e. Suggested next steps	**X**
	**Other information**	
18. Funding	Sources of funding that supported this work. Role, if any, of the funding organization in the design, implementation, interpretation, and reporting	**X**

For peer review only

BMJ Open

Title: Development of support material for health professionals who are implementing Shared Decision-Making in breast cancer screening: Validation using Delphi technique

Journal:	*BMJ Open*
Manuscript ID	bmjopen-2021-052566.R2
Article Type:	Original research
Date Submitted by the Author:	17-Dec-2021
Complete List of Authors:	Hernández Leal, María José; Universitat Rovira i Virgili, Economic<br>Codern-Bové, Núria; Universitat Autònoma de Barcelona<br>Pérez-Lacasta, María José; University Rovira i Virgili, Economica<br>Cardona, Angels; Area Q , Evaluation and Research in the Field of Social Sciences and Health.<br>Vidal, Carmen; Catalan Institute of Oncology, Cancer Prevention and Control Programme<br>Carles-Lavila, Misericòrdia; Universitat Rovira i Virgili, Department of Economics
**Primary Subject Heading**:	Medical education and training
Secondary Subject Heading:	Patient-centred medicine, Qualitative research, General practice / Family practice, Medical education and training, Oncology
Keywords:	MEDICAL EDUCATION & TRAINING, Breast tumours < ONCOLOGY, PREVENTIVE MEDICINE, PRIMARY CARE, PUBLIC HEALTH, QUALITATIVE RESEARCH

SCHOLARONE™
Manuscripts

I, the Submitting Author has the right to grant and does grant on behalf of all authors of the Work (as defined in the below author licence), an exclusive licence and/or a non-exclusive licence for contributions from authors who are: i) UK Crown employees; ii) where BMJ has agreed a CC-BY licence shall apply, and/or iii) in accordance with the terms applicable for US Federal Government officers or employees acting as part of their official duties; on a worldwide, perpetual, irrevocable, royalty-free basis to BMJ Publishing Group Ltd ("BMJ") its licensees and where the relevant Journal is co-owned by BMJ to the co-owners of the Journal, to publish the Work in this journal and any other BMJ products and to exploit all rights, as set out in our licence.

The Submitting Author accepts and understands that any supply made under these terms is made by BMJ to the Submitting Author unless you are acting as an employee on behalf of your employer or a postgraduate student of an affiliated institution which is paying any applicable article publishing charge ("APC") for Open Access articles. Where the Submitting Author wishes to make the Work available on an Open Access basis (and intends to pay the relevant APC), the terms of reuse of such Open Access shall be governed by a Creative Commons licence – details of these licences and which Creative Commons licence will apply to this Work are set out in our licence referred to above.

Other than as permitted in any relevant BMJ Author's Self Archiving Policies, I confirm this Work has not been accepted for publication elsewhere, is not being considered for publication elsewhere and does not duplicate material already published. I confirm all authors consent to publication of this Work and authorise the granting of this licence.

For peer review only

Original Research Article

Title: Development of support material for health professionals who are implementing Shared Decision-Making in breast cancer screening: Validation using Delphi technique.

María José Hernández-Leal[1,a,b,c], Núria Codern-Bové[2,d], María José Pérez-Lacasta[3,a,c], Angels Cardona[4,e], Carmen Vidal-Lancis[5,f], Misericòrdia Carles-Lavila[6,a,b,c] on behalf of the ProShare Group

1. PhD student, MSc, BSc Nursing. Email: mariajose.hernandez@urv.cat
2. PhD. Email: nuriacodern@euit.fdsll.cat
3. PhD. Email: mariajose.perez@urv.cat
4. MSc. Email: angels.cardona@areaq.net
5. MPH. Email: cvidal@iconcologia.net
6. PhD. Email: misericordia.carles@urv.cat

a. Department of Economics, Universitat Rovira i Virgili.
b. Research Centre on Economics and Sustainability (ECO-SOS).
c. Research Group on Statistics, Economic Evaluation and Health (GRAEES).
d. Terrassa University School of Nursing and Occupational Therapy (EUIT), Universitat Autònoma de Barcelona, Terrassa, Spain.
e. Area Q: Evaluation and Research in the Field of Social Sciences and Health.
f. Cancer Prevention and Control Program, Catalan Institute of Oncology-IDIBELL, L'Hospitalet de Llobregat, Spain.

ProShare group: Misericòrdia Carles-Lavila, Núria Codern-Bové, María José Hernández-Leal, María José Pérez, Roger Pla, Vanesa Ramos, Carmen Vidal.

Corresponding author:
Misericòrdia Carles-Lavila, Department of Economics, Universitat Rovira i Virgili, Avenida Universitat 1, 43204, Reus, Spain. Email: misericordia.carles@urv.cat

Word count: 5090

For peer review only

ABSTRACT

Background: Literature on non-reported support material about Shared Decision-Making (SDM) to breast cancer screening for Health professionals in Spain. The researcher created both a handbook and guide for this topic using an adaption of The Three-talk Model.

Objective: *A Delphi method will be used to reach an agreement among experts on the contents and design of a manual and guide, designed by the research team, and to be used by health professionals in the application of SDM in breast cancer screening.*

Design: A qualitative study; the content and design of the handbook and the guide was discussed by 20 experts. Delphi was an online mode between July and October 2020 and researchers used Google forms in three rounds with open and close questions. The criterion established for consensus was a coefficient of concordance (Cc) above 75, for questions using a Likert scale of 1 to 6 - in which 1 meant 'completely disagree' and 6 'completely agree'- with a cut-off point equal to or higher than 4.

Results: Participants considered The Three-talk Model suitable for the screening context. The handbook sections and level of detail were considered satisfactory (Cc=90). The summary provided by the clinical practice guide was considered necessary (Cc=75), as it was the self-assessment tool for professionals (Cc=85). Content was added: addressing the limitations of the SDM model; extending the number of example dialogues for health professionals; providing supplementary resources on using Patients Decisions Aids (PtDAs) and adding references on communication skills.

Conclusions and applications: The first handbook and clinical practice guide providing unique SDM support material for health professionals have been developed. The handbook and guide are useful and innovative as supporting material for health professionals, but to facilitate its implementation, training strategies for SDM and a piloting plan for the use of materials are requested.

Keywords: Shared Decision-Making; breast cancer screening; health professionals.

STRENGTHS AND LIMITATIONS OF THIS STUDY

- Development of a handbook and a clinical practice guide to Shared Decision-Making for breast cancer screening.
- Adaptation of The Three-talk Model to breast cancer screening.
- Participation of professionals in validating the design of the support materials.
- Facilitating the application of a person-centred model to the screening context.

For peer review only - http://bmjopen.bmj.com/site/about/guidelines.xhtml

BACKGROUND

Shared Decision-Making (SDM) is recommended in an uncertainty context -among others- in which it is necessary to argue on risks and benefits in health topics (1)-. SDM is a relationship doctor-patient model, and both collaborate to deliberate over the best choice based not only on scientific evidence but also on women's preferences and values (2,3). Thus, the SDM invites you to change the paternalistic health model for a more participatory one, seeking patients' greater involvement in their health, instead of aiming at a greater adherence to treatments, procedures, or medicines, even though it has also been associated as a result of its application (4).

In Spain, Law 21/2000 Health Information Rights, Patient Autonomy and Clinical Documentation (5) protects the right to decide freely. However, SDM is not explicitly recommended for screening programs. And the scientific community are making efforts to create PtDAs (6,7) to be integrated in the Early Detection Programmes of Autonomous Communities, but, at the moment, its use is not widespread."

The breast cancer screening (BC) programme currently falls under the Oncology Master Plan (*Plan Director de Oncología*) in Catalonia (8). However, while there are strategies for incorporating women's values and preferences into the decision on whether having the examination or not, there is no associated framework on how to put them into practice (9). The current situation in Catalonia is the this: the Breast Cancer Detection Programme (*Programa de Detección del Cáncer de Mama*) sends -every two years- women between 50 and 69 years old a letter informing them of the time and date when they should attend their local health centre to have a mammogram (10). The programme achieves a high level of coverage, but it fails to incorporate an opportunity for women and professionals to exchange information and have a dialogue on her decision. To promote women's participation, several research teams have developed projects that involve women in making their decision on screening. In 2017, Toledo-Chavari and their colleagues created a PtDAs (5) (Annex 1), consisting of a trifold leaflet that provided balanced information on either the benefits or adverse effects, for both professionals and women to use it during the clinical appointment. However, based on the barriers and enabling factors cited in the literature (11-13), the researchers decided not to use the PtDAs alone, for it was not enough, and concluded that SDM training material aimed at health professionals was also needed. The manual is training material, since they are a useful tool to transmit knowledge and provide quick and simple information on how to operationalize new practices, introducing beginners into the theme on how to use it the same way advanced users do (14). Considering that SDM is not a common practice, a manual could, to some extent, fill knowledge gaps on this model.

The ProShare Study. Our research team has therefore developed a handbook-manual- (Annex 2) and guide (15) (Annex 3) aimed at health professionals who have a direct relationship with women. These documents should be used as reference material by health professionals when facing the decision with women on whether to perform or -not to- a mammography, taking into consideration key elements and providing the patient with: information and education, and interpersonal communication between doctor and patient for a final decision (16). To facilitate the implementation of SDM, the model used as a reference was The Three-talk model. The model was created so that three key steps (1- Team Talk, 2- Option Talk, 3- Decision Talk) would be quickly grasped and to explain in an easy way how to apply SDM in generic health context for healthcare professionals (17). In this article we are adapting the three steps of Model

For peer review only - http://bmjopen.bmj.com/site/about/guidelines.xhtml

to specific health context in BC screening to: 1) Team talk; 2) Option talk and exploring preferences; 3) Decision talk. A self-assessment of the SDM was included in the manual, which should be applied at the end of the appointment so that professionals can identify strengths and weaknesses in the implementation of the SDM. Finally, the guide provides a summary of the handbook to be used in the same appointment as a reminder of the three steps.

The objective of this study is *using a Delphi method to reach an agreement among experts on the contents and design of a manual and guide, designed by the research team and to be used by health professionals in the application of SDM in breast cancer screening*.

METHODS

Delphi Technique

The Delphi technique main objective is reaching consensus among experts on specific topics. For this reason, it was decided to use it since the moment you want developing training competencies, tools to support clinical practice or a response to a professional issue. Thus, seeking the opinion of experts is a common approach (18) and in this case experts are required for the development of a manual and guide because there are few documents focused on health professionals explaining the application of SDM, specifically for breast cancer screening. Another feature of the Delphi Technique is that participants undergo a series of online surveys question rounds, which are formulated with elements not agreed upon in the previous round (19-20). This process is repeated continuously until one of the completion criteria is met. (21). A further requirement for the formulation of the Delphi is that the responses of all experts must be shared in each round, allowing experts to reassess their responses in the light of other experts' views. Finally, all the rounds must be carried out anonymously and therefore ensure that they do not influence on others just because of one expert's considerable knowledge on the topic. One of the limitation the Delphi technique has is that it provides experts' opinion; however, other complementary techniques could also be considered to determine a final position on the subject of the study (18-20). The experts participating in a virtual way can overcome barriers related to economic circumstances and geographical or time-related constraints (19,20). Experts, according to literature, can be grouped into two broad categories: *Subjects (Su)* – people who would use the instrument in their profession; and *Specialists (Sp)* – people who have knowledge about the subject due to their academic and/or professional experience (19,20).

Participants

The handbook and clinical practice guide, entitled 'The participation of health professionals in Shared Decision-Making in breast cancer screening' (*La participación de los profesionales de la salud en la Toma de Decisiones Compartida en el cribado de cáncer de mama*) (Annex 4 and Annex 5) (15), were developed by the Pro-Share research team. The first version was produced with the participation of three researchers with experience in SDM and BC screening, who acted as external reviewers, and two health professionals, who designed the plan for piloting the questionnaire online (Google form).

The included criteria for participants were as follows*:*

- *Subjects*: a) health professionals, preferably from primary care services, who provide direct care to women through breast cancer prevention activities, and b) health

For peer review only - http://bmjopen.bmj.com/site/about/guidelines.xhtml

professionals, who have at least five years' experience (21) in the Spanish Health System.

- *Specialists*: a) international-level researchers whose research career has focused on the Shared Decision-Making model, and b) those who are proficient in Spanish (given that the handbook has been produced in Spanish). Preference was given to individuals who had developed educational support material for professionals (21).

To determine the size of the sample, literature was consulted. It is mentioning that large numbers (over 50 people) could imply an impediment for so many people reaching an agreement in a limited time. Moreover, it depends on the heterogeneity of the experts. If they are from different countries or various specializations, they enrich the opinions formulated (20). Therefore, a limit between 7 and 30 (20) was decided, most commonly being a total of 15 to 20 experts (20).

Patient and Public Involvement

Health professional and researcher gave feedback on the Delphi rounds about the manual and guide practice, which were adapted accordingly.

Procedure and Data collection

The researcher's two sampling strategies were used to recruit participants: convenience sampling for specialists and snowball sampling for health professionals. For specialists, the researchers were looking for published articles about SDM and contacted the authors via e-mail (MJH, MC, MJP). For health professionals, researchers sent an e-mail with an invitation to (NC, AC), and they could be resent to other colleagues. Finally, the researchers (NC-AC) sent invitations via e-mail to 43 potential experts to participate in a Delphi, 30 of whom accepted. The aim was to determine the usefulness of the topics, the relevance of the content and the designed document of the material for the SDM on BC screening. The Delphi was being done on Google form between July and October 2020.

For round 1, open and close questions were considered with relevant topics to the research objective "*The sections of the handbook are effective for understanding the application of SDM to breast cancer screening* " or "*Do you think that a guide concisely summarizing the SDM steps is necessary?"*. Participants should mark the degree of agreement to the questions using a Likert scale of 1 to 6, in which 1 was 'completely disagree' and 6 was 'completely agree'. Later, when all experts finished the survey research (MJH-MC-MJP) and were already sent, experts received a report with the answers so that the participants could consider the other participants' views (anonymous), especially in those questions, in which no agreement was reached, group (Cc = 75). Disagreement questions were raised again in the following rounds until the necessary agreement was reached in most transversal aspects. This was finally achieved in round 3.

Data analysis

The researchers (MJH-MC-MJP-NC-AC) analysed participants' responses at the end of each round, considering the responses whose score on the Likert scale was 4 or above to be positive. Agreement was determined to be reached when the coefficient of concordance (Cc) was higher than 75 (22) The Coefficient of Concordance (Cc) >75 was used. For calculation consider the next formula:

For peer review only - http://bmjopen.bmj.com/site/about/guidelines.xhtml

$$Cc = \left(1 - \frac{Vn}{Vt}\right) x100$$

Vn = Number of negative votes (a score of lower than 4); Vt = Total number of votes (n=6) (22)

For R3, the criteria established by Martínez (2003) were considered to bring the Delphi close (23).

This research was approved by the Medicinal Product Research Ethics Committee (CEIm) of *Institut d'Investigació Sanitària Pere Virgili* (Pere Virgili Health Research Institute). Informed consent, which stated that participants accepted the conditions of participation upon agreeing to respond to the questionnaire which was secure. These conditions specified that responses were confidential and would only be used for the purpose of this research.

RESULTS

Out of the 30 professionals who initially agreed to participate, 20 (66.6%) went on to respond in the first round (R1), 16 (53.3%) in the second one (R2) and 17 (56.6%) in the third one (R3) (Figure 1). In R1, the mean age of the experts was 46.6 years (SD 10.25), 75% were female, 65% were doctors, 70% worked in the public sector and they were on average of 19 years' (SD 9.69) experience (Table 1).

Figure 1: Flow diagram of participation in each round

Table 1: Characteristics of the participants

Variable		Round 1		Round 2		Round 3	
		N	%	N	%	N	%
Sex	Female	15	75	12	75	13	76.47
	Male	5	25	4	25	4	23.52
	Total	20	100	16	100	17	100
Age range (years)	31-40	7	35	7	43.75	7	41.17
	41-50	6	30	4	25	5	29.41
	51-60	5	25	4	25	4	23.52
	61-70	2	10	1	6.25	1	5.88
	Total	20	100	16	100	17	100
Ownership of the affiliated institute, health centre or research site	Public sector	14	70	11	68.75	11	64.7
	Private sector	6	30	5	31.25	6	35.29
	Total	20	100	16	100	17	100
Profession	Nursing	4	20	2	12.5	3	17.64
	Medicine	13	65	11	68.75	11	64.7
	Psychology	1	5	1	6.25	1	5.88
	Other	2	10	2	12.5	2	11.76
	Total	20	100	16	100	17	100
Specialty	Family and community medicine or nursing	14	70	11	68.75	12	70.58
	Public health	1	5	1	6.25	1	5.88
	Gynaecology	1	5	1	6.25	1	5.88
	Endocrinology	1	5	1	6.25	1	5.88
	Research in health services	1	5	1	6.25	1	5.88
	Content development for Decision Support Systems for Healthcare	1	5	1	6.25	1	5.88
	None	1	5	0	0	0	0

For peer review only - http://bmjopen.bmj.com/site/about/guidelines.xhtml

	Total	20	100	16	100	17	100
Experience (years)	6-10	6	30	6	37.5	6	35.29
	11-20	6	30	5	31.25	6	35.29
	21-30	6	30	5	31.25	5	29.41
	31-40	2	10	0	0	0	0
	Total	20	100	16	100	17	100

Among the outcomes of the Delphi an agreement on the content and design of the documents could be reached. Among the three rounds carried out, four significant changes were made regarding the contents: 1) including examples of practical dialogues for each phase, 2) annexed additional information on communicative skills, 3) incorporation of information on how to manage professionals' responsibility in SDM 4) additional information about limitation of SDM model 5) elimination of the flow of the screening program in Catalonia.
It was impossible to determine why professionals changed their decisions in the rounds, since they only had options to change their vote once the previous rounds results were known and their peers' arguments were read. Below there are the results for each round.

Round 1

R1 was designed to achieve two objectives: determinate its utility and clarify the content and the design of the supporting material. For this purpose, participants were asked 33 Likert-scale questions, 1 multiple-choice question and 6 open questions on the handbook and they were also given 2 Likert-scale questions and 4 open questions on the clinical practical guide (Table 2).

Table 2: R1 responses

Section	Questions using a Likert scale of 1 (Completely disagree) to 6 (Completely agree)	1	2	3	4	5	6	[illegible]
Evaluation of the Handbook on Shared Decision-Making in Breast Cancer Screening	1. The sections of the handbook are effective for understanding the application of SDM to breast cancer screening	0	5	5	10	55	25	[illegible]
	4. The 'Contents' section is suitable for this handbook	0	5	5	5	35	50	[illegible]
	5. The 'Objective of the material' section is suitable for this handbook	0	5	0	5	30	60	[illegible]
	6. The 'Who is it aimed at?' section is suitable for this handbook	0	5	0	10	35	50	[illegible]
	7. The 'Introduction' section is suitable for this handbook	5	10	0	10	45	30	[illegible]
	8. The 'Shared Decision-Making: What is it?' section is suitable for this handbook	0	5	5	10	40	40	[illegible]
	9. The 'Shared Decision-Making: Why is it important?' section is suitable for this handbook	0	5	0	5	45	45	[illegible]
	10. The 'Shared Decision-Making: 'What skills or competencies do health professionals need?' section is suitable for this handbook	0	5	5	35	35	20	[illegible]
	11. The 'Shared Decision-Making: What do patients think?' section is suitable for this handbook	0	10	0	10	35	45	[illegible]
	12. The 'Shared Decision-Making in breast cancer screening: The screening programme' section is suitable for this handbook	0	10	10	5	30	45	[illegible]
	13. The 'Shared Decision-Making in breast cancer screening: Implementation of SDM in breast cancer screening' section is suitable for this handbook	0	5	0	5	45	45	[illegible]
	14. The 'Shared Decision-Making in breast cancer screening: Self-assessment of the SDM process' section is suitable for this handbook	0	10	10	10	35	35	[illegible]
	15. The handbook provides the minimum content on SDM in	0	5	0	20	50	25	[illegible]

For peer review only - http://bmjopen.bmj.com/site/about/guidelines.xhtml

For peer review only

	breast cancer screening that health professionals should be familiar with							
	16. The content of the handbook is sufficiently detailed	0	5	5	5	35	50	[illegible]
	19.a. Figure 1: Models of healthcare (page 14) is useful	0	0	10	15	30	45	[illegible]
	19.b. Figure 1: Models of healthcare (page 14) is clear	0	0	15	10	20	55	[illegible]
	20.a. Figure 2: Role of the participants in the clinical encounter (page 15) is useful	0	5	5	10	35	45	[illegible]
	20.b. Figure 2: Role of the participants in the clinical encounter (page 15) is clear	0	5	5	15	30	45	[illegible]
	21.a. Figure 3: Elements of Shared Decision-Making (page 16) is useful	0	0	10	20	25	45	[illegible]
	21.b. Figure 3: Elements of Shared Decision-Making (page 16) is clear	0	0	5	20	25	50	[illegible]
	22.a. Figure 4: Communication skills (page 21) is useful	5	0	10	30	15	40	[illegible]
	22.b. Figure 4: Communication skills (page 21) is clear	0	0	10	20	25	45	[illegible]
	23.a. Figure 5: Flow diagram of the Early Detection of Breast Cancer programme (page 27) is useful	5	5	20	15	20	35	[illegible]
	23.b. Figure 5: Flow diagram of the Early Detection of Breast Cancer programme (page 27) is clear	10	10	20	15	15	30	[illegible]
	24.a. Figure 6: Team talk (page 34) is useful	10	5	0	30	25	30	[illegible]
	24.b. Figure 6: Team talk (page 34) is clear	10	0	15	20	25	30	[illegible]
	26.a. Figure 7: Option talk (page 36) is useful	5	5	0	30	40	20	[illegible]
	26.b. Figure 7: Option talk (page 36) is clear	5	0	10	45	15	25	[illegible]
	28.a. Figure 8: Decision talk (page 38) is useful	0	5	10	5	35	45	[illegible]
	28.b. Figure 8: Decision talk (page 38) is clear	0	0	5	15	30	50	[illegible]
	30.a. Figure 9: Shared Decision-Making steps (page 39) is useful	0	0	5	20	25	50	[illegible]
	30.b. Figure 9: Shared Decision-Making steps (page 39) is clear	0	0	10	35	10	45	[illegible]
	31. Does its design (colours, images) make the handbook easier to read for an SDM professional?	0	0	5	20	35	40	[illegible]

	Closed questions	Options	Percentage
	2. Which section of the handbook do you think should be changed?	a) Front cover	0
		b) Objective of the material	0
		c) Who is it aimed at?	0
		d) Introduction	10
		e) Shared Decision-Making: What is it?	0
		f) Shared Decision-Making: Why is it important?	0
		g) Shared Decision-Making: What skills or competencies do health professionals need?	25
		h) Shared Decision-Making: What do patients think?	0
		i) Shared Decision-Making in breast cancer screening: The screening programme	15
		j) Shared Decision-Making in breast cancer screening: Implementation of SDM in breast cancer screening	0
		k) Shared Decision-Making in breast cancer screening: Self-assessment of the SDM process	0
		l) None	50
		Total	100

Evaluation of the Clinical Practice Guide: Implementation of SDM for Health Professionals	Questions using a Likert scale of 1 (Completely disagree) to 6 (Completely agree)	1	2	3	4	5	6	[illegible]
	1. Do you think that a clinical practice guide concisely summarising the SDM steps is necessary?	0	15	10	5	5	65	[illegible]
	6. Is it useful to incorporate the Self-assessment section in the clinical practice guide?	0	5	10	25	25	35	[illegible]

A Cc higher than 75 was recorded for 32 of the Likert-scale questions and the minimum Cc was not reached by only 3 of them; in other words, no agreement was reached. These questions

For peer review only

For peer review only - http://bmjopen.bmj.com/site/about/guidelines.xhtml

concluded that ‘Flow diagram of the Early Detection of Breast Cancer programme’, was clear (Cc=60) and useful (Cc=70) (Figure 2). The same applied to the question that determined Team talk (page 34) – to be clear (Cc=75). These questions were incorporated into R2.

Figure 2: Flow diagram of the Early Detection of Breast Cancer Programme

In the multiple-choice question, participants were asked which section of the handbook should be edited: 10 responded ‘none’; 5 chose the section entitled ‘Which skills or competencies do health professionals need?’; 3 chose the ‘Screening programme’ section, and 2 chose the ‘Introduction’ (Figure 3).

Figure 3: Changes made to the index

In their open responses, most participants considered the initiative to be positive and thought it would enable health professionals to access information on SDM using the Three-talk model in BC screening (Box 1). However, one of the participants suggested using the Agency for Healthcare Research and Quality model.

Box 1: Response to the question: Are the steps based on “Three-talk” suitable for the application of SDM in breast cancer screening? Please explain briefly.

P3 (R1): *Yes, it shows how the health professional can implement SDM in a three-step process in a brief, practical and easy-to-read way. It describes the characteristics that differentiate each step, and specific examples of implementation in breast cancer screening.*

The participants also provided some suggestions to modify the handbook. The most frequently cited were concerned about the length of the handbook and recommended simplifying the content (Box 2) and incorporating example dialogues, communication skills (Box 3) and instructions for using the PtDAs. The comments were incorporated in the questions in R2.

Box 2: Response to the question: How would you improve the elements selected in the previous question?

P7: *I think that the handbook is very long, which may reduce motivation to read it.*
P6: *Very long and it doesn’t show how to use the tool.*

Box 3: Response to the question: What other content would you include in the clinical practice guide?

P3: *Provide more information or example dialogues on how to use communication skills. This last [point] if the health professionals don’t have a grounding or training in active listening, motivational interviewing, empathy, reflection, etc.*
P10: *I’d go into greater depth on relationship-building skills and give a few links to where they can find exercises to train themselves [in this].*

For peer review only - http://bmjopen.bmj.com/site/about/guidelines.xhtml

Finally, in response to the question of whether the dialogues in each step represent their objective, most participants agreed (‘Team talk’ step, n=10; ‘option talk’ step, n=7; ‘Decision talk’ step, n=12) and made suggestions on the wording of the dialogues. Suggestions were also made to adapt the name of the original *the Three-talk* steps to a more representative one in the screening context. All the suggestions were incorporated into R2 to be approved or rejected by the other participants.

Only one of the questions evaluating the clinical practice guide did not reach the minimum Cc established: ‘Do you consider a guide that concisely summarises the SDM steps to be necessary?’ (*¿Cree necesaria una guía que resuma de forma rápida las fases de la TDC?*) (Cc=75). This question was incorporated into R2. In the open questions, participants suggested changing the wording of the step 1 dialogues (n=3) and incorporating a review of communicative skills (Box 4); the same was applied to step 2, but participants added a comment about using relative risks instead of absolute ones (n=1) (Box 5).

Box 4: Response to the question: What elements would you change in step 1: ‘Team talk’?

P3: *I’d include a few reviews, such as [on] active listening and deliberation. Perhaps using a phrase like ‘Remember to pay close attention and give assertive responses (active listening), and to think the options through carefully for the decision (deliberation)’.*

Box 5: Response to the question: What elements would you change in step 2: ‘Option talk?

P15: *Change relative risks to absolute risks.*

They also proposed: eliminating the definition of SDM for step 3 in the guide (n=4), incorporating a brief clarification noting that women may also consult other people for support in making their decision (n=3) and mentioning the possibility of reversing the decision (n=4) (Box 6). Between 6 and 8 people stated that they would not make any change to steps 1, 2 or 3.

Box 6: Response to the question: What elements would you change in step 3: ‘Decision talk’?

P11: *I’d add the possibility of reversing the decision; I’d take out the explanation about SDM.*

Finally, in the last question – ‘What other content would you include in the clinical practice guide?’ – participants reiterated the need to include a review of communication skills (n=3) and one of them proposed changing the self-assessment to use either the ASQ3 or the CollaboRATE instrument.

Round 2

R2 was structured around/ was based on open-question responses and included the elements about which agreement had not been reached in the previous round. Thirteen Likert-scale questions, 5 multiple-choice question and 6 open questions were produced in the handbook.

For peer review only - http://bmjopen.bmj.com/site/about/guidelines.xhtml

For the clinical practice guide, 2 Likert-scale questions and 5 open questions were included (Table 3).

Table 3: R2 responses

<table>
<tr><th>Section</th><th colspan="2">Questions using a Likert scale of 1 (Completely disagree) to 6 (Completely agree)</th><th>1</th><th>2</th><th>3</th><th>4</th><th>5</th><th>6</th><th>CC*</th></tr>
<tr><td rowspan="29">Evaluation of the Handbook on Shared Decision-Making in Breast Cancer Screening</td><td colspan="2">1. Eliminate Figure 5: Flow diagram of the Early Detection of Breast Cancer Programme (page 27)</td><td>6.3</td><td>18.8</td><td>18.8</td><td>6.3</td><td>43.8</td><td>6.3</td><td>56.4</td></tr>
<tr><td colspan="2">2. Shorten content: the handbook format is very long</td><td>0</td><td>12.5</td><td>6.3</td><td>18.8</td><td>25</td><td>37.5</td><td>81.3</td></tr>
<tr><td colspan="2">4. Incorporate more examples of dialogues between the professional and the woman into each phase</td><td>18.8</td><td>6.3</td><td>6.3</td><td>31.3</td><td>31.3</td><td>6.3</td><td>68.9</td></tr>
<tr><td colspan="2">5. Add information on communication skills and competencies resources</td><td>0</td><td>12.5</td><td>25</td><td>12.5</td><td>43.8</td><td>6.3</td><td>62.6</td></tr>
<tr><td colspan="2">6. Add information on joint responsibility for the shared decision-making agreement</td><td>6.3</td><td>31.3</td><td>12.5</td><td>0</td><td>37.5</td><td>12.5</td><td>50</td></tr>
<tr><td colspan="2">7. Add information about resources on using the Patients Decision Aids (PtDAs). Note that this tool is intended to be used with the women</td><td>0</td><td>18.8</td><td>12.5</td><td>18.8</td><td>18.8</td><td>31.3</td><td>68.9</td></tr>
<tr><td colspan="2">8. Add information on the limitations of the SDM model</td><td>6.3</td><td>18.8</td><td>25</td><td>12.5</td><td>25</td><td>12.5</td><td>50</td></tr>
<tr><td colspan="2">9. Provide example dialogues on exploring the women's values, beliefs and preferences</td><td>0</td><td>18.8</td><td>12.5</td><td>18.8</td><td>31.3</td><td>18.8</td><td>68.9</td></tr>
<tr><th>Closed questions</th><th colspan="6">Options</th><th colspan="2">Percentage (%)</th></tr>
<tr><td rowspan="12">3. Which element of the handbook would you shorten?</td><td colspan="6">a) Objective of the material</td><td colspan="2">0</td></tr>
<tr><td colspan="6">b) Who is it aimed at?</td><td colspan="2">0</td></tr>
<tr><td colspan="6">c) Introduction</td><td colspan="2">50</td></tr>
<tr><td colspan="6">d) Shared Decision-Making: What is it?</td><td colspan="2">0</td></tr>
<tr><td colspan="6">e) Shared Decision-Making: Why is it important?</td><td colspan="2">0</td></tr>
<tr><td colspan="6">f) Shared Decision-Making: What skills or competencies do health professionals need?</td><td colspan="2">0</td></tr>
<tr><td colspan="6">g) Shared Decision-Making: What do patients think?</td><td colspan="2">6.3</td></tr>
<tr><td colspan="6">h) Shared Decision-Making in breast cancer screening: The screening programme</td><td colspan="2">6.3</td></tr>
<tr><td colspan="6">i) Shared Decision-Making in breast cancer screening: Implementation of SDM in breast cancer screening</td><td colspan="2">6.3</td></tr>
<tr><td colspan="6">j) Shared Decision-Making in breast cancer screening: Self-assessment of the SDM process</td><td colspan="2">0</td></tr>
<tr><td colspan="6">k) None</td><td colspan="2">31.3</td></tr>
<tr><td colspan="6">Total</td><td colspan="2">100</td></tr>
<tr><td rowspan="4">10. Change the name of phase 2</td><td colspan="6">a) Option talk (current name)</td><td colspan="2">18.8</td></tr>
<tr><td colspan="6">b) Option talk and exploring preferences (proposal)</td><td colspan="2">81.3</td></tr>
<tr><td colspan="6">c) Other</td><td colspan="2">0</td></tr>
<tr><td colspan="6">Total</td><td colspan="2">100</td></tr>
<tr><td rowspan="3">12. Phase 1 dialogue: Team Talk (page 34):</td><td colspan="6">a) Now that we know that you can decide what to do about screening, we're going to talk about the characteristics of screening, so that you know what your options are (current dialogue).</td><td colspan="2">12.5</td></tr>
<tr><td colspan="6">a) You have the option of deciding whether or not to have early-detection tests for breast cancer. If you're happy to, we can explore together what risks and benefits the test involves for you (proposal).</td><td colspan="2">81.3</td></tr>
<tr><td colspan="6">c) Other</td><td colspan="2">6.2</td></tr>
</table>

For peer review only

		Total	100
	14. Phase 2 dialogue: Option Talk (page 36)	a) I appreciate you sharing your views with me and I'm here to help you come to a good decision. Let's do a recap of your preferences and check whether you have any more questions (current dialogue).	18.8
		B) I'm here to help you make a decision. Let's look at what your preferences are and the various options available, and we'll check whether you have any questions about them (proposal).	75
		c) Other	6.2
		Total	100
	16. Phase 3 dialogue: Decision Talk (page 38):	a) Do you think that you're ready to make the decision or do you need more time? (current dialogue).	12.5
		b) Now that we've gone over the advantages and disadvantages of early detection, do you think that you can make the decision? Bear in mind that this can be delayed if you need more time or to talk about it with someone of your choice (proposal).	81.3
		c) Other	6.2
		Total	100

Evaluation of the Clinical Practice Guide: Implementation of SDM for Health Professionals	Questions using a Likert scale of 1 (Completely disagree) to 6 (Completely agree)	1	2	3	4	5	6	Cc*
	1. A clinical practice guide is necessary for this handbook	6.3	0	12.5	25	31.3	25	81.3
	2. Improve the design of the clinical practice guide to improve understanding (colour, structure, etc.)	6.3	0	18.8	37.5	25	12.5	75
	3. Eliminate additional information (definitions of Risk factors, Mammography, Shared Decision-Making)	6.3	18.8	18.8	6.3	18.8	31.3	56.4
	4. Mention the possibility of reversing the decision in the follow-up plan	6.3	0	6.3	18.8	25	43.8	87.6
	5. Mention relationship-building competencies: active listening, showing empathy, clarification, etc.	12.5	6.3	12.5	6.3	43.8	18.8	68.9

Of the 13 Likert-scale questions, only 3 reached a score of Cc>75. These underlined the need to: reduce the length of the handbook (Cc=81.3), create a clinical practice guide to accompany the handbook (Cc=81.3), and mention the possibility of reversing the decision in the follow-up plan (Cc=87.6).

The close ended questions included the following – 'Which elements of the handbook would you shorten?' (*¿Qué elementos reducirían del Manual?*) – to which the two most significant answers were 'the Introduction' (50%) and 'None' (31.3%). Following the comments made in the previous round, alternative formulations of the sample phrases for the dialogues in each of *the Three-talk* steps were given, as well as a change of name for step 2: 'Option talk and exploring preferences' (*Plantear opciones y explorar preferencias*), on which consensus was reached (81,3%).

In their responses to the open questions, those who considered the proposed dialogues unrepresentative of the steps had the opportunity to suggest a rewording. Finally, participants were able to include their final comments in both the handbook and the guide (Figure 4).

Figure 4: Changes made to the guide

For peer review only

For peer review only - http://bmjopen.bmj.com/site/about/guidelines.xhtml

Most had no further suggestions for each document, but some participants included comments about shortening the handbook (Box 7) and including this material in clinical practice guides, in order to improve implementation (Box 8).

Box 7: Response to 'Provide your final comments on the handbook'

P10: *None, the idea of including appendices on communication skills for the health professional, and on the screening tests for the women, seems like an excellent idea to me, to avoid making the handbook longer but offer additional tools for those health workers and women who would like more information.*

Box 8: Response to 'Provide your final comments on the guide'

P10: *Clinical practice guidelines on the preventive approach to breast cancer that includes these points on shared decision-making would be very useful to support implementation. In any case, I don't think that it is a prerequisite to be able to produce the handbook that you are working on. This handbook could be incorporated into future Clinical Practice Guidelines (CPG).*

Round 3

R3 was structured according to the 10 elements on which no agreement was reached in R2. Six questions with close ended, dichotomous answers were posed in the section evaluating the handbook, and 1 in the section evaluating the clinical practice guide; in addition to an open question. Of these, only those proposing an improvement to the organisation of the clinical practice guide, a change of colours and a review of cross-cutting communication skills in SDM reached a Cc of over 75% (Table 4).

Table 4: R3 responses

Section	Closed questions	Options	Percentage (%)
Evaluation of the Handbook on Shared Decision-Making in Breast Cancer Screening	1. Given that no consensus has been reached (56.4 %) on whether or not to eliminate Figure 5: Flow diagram of the Early Detection of Breast Cancer programme (page 27), please select one of the following options:	a) Eliminate. It does not add relevant information to this handbook	47.1
		b) Keep. Translate to Spanish and improve the image resolution	52.9
		Total	
	2. Given that there is no consensus (68.9 %) about whether to add more examples of dialogues between the professional and the women for each phase, please select one of the following options:	a) One example per phase (current format)	35.3
		b) Three examples per phase (proposed new format) The image will be adapted to a more readable size for the handbook	64.7
		Total	100
	3. Given that there is no consensus (62.6 %) about whether to add information on communication skills and competencies resources to the	a) Yes, it is necessary to incorporate bibliographic references into the handbook for those who would like to find out more about this topic.	58.8

For peer review only

	handbook, please select one of the following options:	b) No, the handbook is already too long to add more information.	64.7
		Total	100
	4. Given that there is no consensus (50 %) about whether to include information on joint responsibility for the SDM agreement, please select one of the following options:	a) Yes, it should be included because the information is not clear	41.2
		b) It is not necessary, it is already clear that the responsibility is shared	58.8
		Total	100
	5. Given that there is no consensus (68.9 %) about whether bibliographic references should be added on the PtDAs– note that the PtDAs is an appendix to the handbook, to be used by the woman and health professional – please select one of the following options:	a) Yes, they should be added	52.9
		b) No, this is not necessary	47.1
		Total	100
	6. Given that there is no consensus (50 %) about whether to add information on the limitations of the model, please select one of the following options:	a) Yes, this is necessary because not doing so would mean producing one-sided material	58.8
		b) No, it is not necessary because the objective of the handbook is to show the advantages of implementing it	41.2
		Total	100
Evaluation of the Clinical Practice Guide: Implementation of SDM for Health Professionals	1. Given that there is no consensus about the design and content of the guide, please select one of the following options. The infographic will be adapted to a more readable size for the guide.	a) Current format	23.5
		b) Proposed new format	76.5
		Total	100

Since that agreement was not reached on the Flow diagram for the Early Detection of Breast Cancer Programme, this figure was removed from the handbook, in light of the fact that it only applies to the region of Catalonia. The other elements in which no agreement was reached were the need for incorporating more samples of professional dialogues (64.7%); incorporating information about joint responsibility for the decision (41.2%); adding information on the limitations of the SDM model (58.8%), as well as adding supplementary resources on the way to use the DST (52.9%) and on communication skills and competencies (58.8%). The researchers believed that the additional content would not entail substantial changes to the handbook but would provide more information for professionals who are not familiar with the model, and that is why all these elements were incorporated into the handbook.

The texts included were developed according to the proposals submitted by the participants in previous rounds. For example, the following elements were highlighted in the professional dialogues: the possibility of reversing the decision, needing more time, and accessing to support from a third person to make the decision (Figure 5).

Figure 5: Example of dialogues for the professionals to "Team talk" step

For peer review only

For peer review only - http://bmjopen.bmj.com/site/about/guidelines.xhtml

The Delphi was brought to close in R3, taking into account the criteria cited by Martínez, regarding the elements about which agreement was not reached (23): a) the limited number of items for which Cc>75 was not achieved (6 of the 61 Likert-scale and closed questions); b) limited resources and time; c) the possibility that participants would abandon the study in a subsequent round, which would affect the external validity of the study. The last two criteria were applied in the context of the COVID-19 pandemic, given that half of the participants were health professionals who work in health centres.

DISCUSSION

The literature mentions certain barriers when applying SDM in BC screening, including limited time in clinical appointments and health professionals' lack of training in providing more participatory care (21). This was the motivation for producing the first handbook and clinical practice guide on this subject, aimed at supporting health professionals by providing them with the essential elements for implementing SDM among women in a BC screening context.

The most relevant results included the validation of usefulness and relevance of support materials when using Delphi technique, considering the experts' opinion to reach agreements on editing the design and content, as well as their recommendation to incorporate these materials into the clinical practice guide. Delphi may be adapted to a generic model –The *Three-talk* – to one specifically designed for the BC screening context.

Of the 43 participants who were invited to respond the Delphi questionnaires, more than a half-showed interest in the topic of the research and collaborated in it. However, only 20 of them went on participating in the study. This may be related to the timetabling of the questionnaires, which coincided with the end of the first wave of the COVID-19 pandemic and the resurgence of cases at the beginning of the second wave. Despite this, the professionals who decided to participate at the beginning of the process fulfilled their commitment, illustrated by the fact that the number of participants simply decreased by three between rounds, these having been lost from the Subjects category (n=3).

Discussion between the participants

It was easy to reach an agreement on the main content elements in the first round. Regarding the structure and development of SDM using the *Three-talk* model (17), which was considered suitable for BC screening, one of the participants initially suggested using the model created by the Agency for Healthcare Research and Quality (24). However, this alternative model contains five steps, and the model proposed by the authors, with fewer steps, met all the requirements of SDM. Regarding the set of nine figures in the handbook, only one was eliminated, and the wording of three was edited.

The participants easily agreed that the initial version of the handbook was very long, 56 pages. Its length was due to the fact that it would be published in a pocket edition, which corresponds to 23 pages in a larger textbook edition. The researchers decided to maintain the smaller format because it is more transportable, whereas they eliminated the content elements agreed by the participants.

It was impossible to reach an agreement on six items. While agreement should be ideally reached for all items, yet, when a new round does not provide more information or it is unlikely to achieve a better result, the rounds of questions may come to an end despite there being a small number of disagreements remaining (21). The formulation changes of the responses

For peer review only - http://bmjopen.bmj.com/site/about/guidelines.xhtml

between R2 (Likert scale) and R3 (dichotomous) meant that participants had to opt for one of the options rather than rating their level of agreement on the statements, which undoubtedly made it more difficult to reach an agreement.

Certain responses to the open questions were analysed in depth by the researchers. One of the participants in R1 suggested that the professional self-assessment method could be changed from SDM-Q-doc (25) to Ask 3Q (26) or CollaboRATE (27). However, Ask 3Q is a methodology for applying SDM, making it equivalent to the Three-talk model. Given that the Three-talk model received positive evaluation from the participants, the change was not made. The other tool, CollaboRATE, is designed for the patient's evaluation of the professional, which was not the purpose of this questionnaire (28). Our objective was to enable the professional to evaluate the way he or she performs SDM, resulting in a self-guided learning of this methodology. The researchers, therefore, kept the original version, SDM-Q-doc, and adapted it for screening.

The decision on the flow diagram was affected by whether participants came from the region of Catalonia (of those living in Catalonia, 5/6 wanted to keep it, albeit improving its resolution; in contrast, the specialists from outside Spain (7/11) opted to remove it). Given that the objective of the handbook is to be used in other territories, the research group decided to eliminate the flow diagram.

The example dialogues suggesting how professionals should conduct SDM at each point in the process were widely accepted as a fundamental part of the handbook, even though no consensus was reached on whether to include more example dialogues for each step (Su=4/6; Sp=7/11). While Cc>75 was not reached, a larger proportion of both groups advocated for providing more examples. This may be directly related to the fact that both groups believed that SDM training for health professionals is still incomplete. Some of these participants therefore called for the handbook to provide more support, giving professionals greater confidence in implementation using the dialogues. The same conclusion can be reached regarding the decision to include more bibliographic references on communication skills and relationship-building competencies (Su=3/6; Sp=7/11) and including information about PtDAs (Su=5/6; Sp=4/11). In the latter case, the results differed from the two groups: most of the Subject participants wanted to add information to these tools, perhaps highlighting their lack of knowledge about them or lack of training in their use, while Specialists did not consider their inclusion so relevant, due to their familiarity with the tools.

How to improve the application of SDM to screening

While 83% of health professionals were strongly interested in promoting Shared Decision-Making during the clinical encounter (28), they admitted their lack of training in the SDM model as one of the most significant barriers to its implementation in the screening context (13).

A review of the training health professionals had received confirmed our belief that there is a lack of strategies to familiarise health professionals with this model. In Spain, the topic has been introduced into medicine and health-related degree programmes (29-32). However, it is not framed accurately within a SDM model, however, it is closer to communication or clinical communication skills, which have been used interchangeably as equivalents to the model. The level of accuracy and strategies used in this training are also unknown. Most training in SDM is acquired in postgraduate-level studies aimed for doctors and nurses (33), whereas particular

For peer review only - http://bmjopen.bmj.com/site/about/guidelines.xhtml

attention should also be paid to health workers in primary care centres (including support and technical staff, as well as clinicians), who provide person-centred healthcare in a holistic manner (34).

Experts in SDM have argued it is necessary to prioritise adapting curricula to consolidate this training, by emphasising an education in communication skills and the accreditation of these competencies (35), within the framework of a horizontal care model. In addition, experts highlight the need to create partnerships between universities and interdisciplinary research groups to develop this material (35).

Experts also recommend a training methodology based on practical activities such as role plays, as well as teamwork, in teams of six people for instance, in a day-long training, and providing constructive feedback on students' capacity to express empathy, giving assertive responses, engaging them in active listening, and other skills (36). This handbook and clinical practice guide, therefore, include dialogues and specific examples of how to apply them. And it will serve as reference material supporting an initial grounding in SDM for professionals who have not received any formal training in this subject, but also as supplementary material for those who have; enabling them to apply the skills and competencies acquired in the specific context of BC screening.

The final structure of our document responds to the need described in the preceding paragraph and highlighted by the participants in the study.

Given the change of paradigm that SDM entails, all measures that help familiarise professionals with SDM are important. For example, adding a section into Clinical Practice Guidelines (CPG) on how to include the patient in decision-making; thereby, coordinating evidence-based practice with SDM (37) may be useful. Patients may even participate, to some extent, in its development, as it is a current practice in such organisations such as the National Institute for Health and Clinical Excellence (NICE) and the Scottish Intercollegiate Guidelines Network (38). In this sense, our proposed handbook and clinical practice guide, as well as the PtDAs, whose quality has already been evaluated and certified by international organisations such as The Ottawa Hospital (39), may be considered as complementary materials.

LIMITATIONS

The main limitation of the study was participants recruitment, which is a typical constraint. It was a particular problem in this case, since the empirical work coincided with the successive waves of the COVID-19 pandemic, which hindered the active participation of some professionals who had initially agreed to participate in the study. Despite this, there were fewer withdrawals from Round 2 onwards that might have been expected in those circumstances.

The change in the formulation of the R2 (Likert scale) and R3 (dichotomous) responses may have made it more difficult to reach the established minimum Cc for agreement. Nevertheless, with reference to Martínez (2003) (21), the research team determined that one more round would not have provided any added value to the results, as shown in the reasons described in the preceding sections. Nevertheless, the decision made regarding those elements about which no agreement had been reached did not significantly affect the participants' opinions regarding the basic concepts on which the initial questionnaire was based.

For peer review only - http://bmjopen.bmj.com/site/about/guidelines.xhtml

Finally, it should be noted that a systematic literature review (2018) showed that further research is still needed to determine the real impact that training interventions have on health professionals regarding SDM, since the level of certainty of the studies was low or very low. In this research, professionals who had received standard training were compared with those who had been trained in SDM; from the 15 studies, it was concluded that the results for patients' satisfaction, knowledge, decision-related conflict, regret, level of health and quality of life differed little or not at all from one to another (33). Despite this, the demand for information and training expressed by this study's participants makes us believe that this first handbook aimed at health professionals for implementation in a BC screening context will help clarify the healthcare model focused on patients' needs and preferences. However, we have also noted the need to expand the training in SDM and develop empirical strategies to facilitate its implementation.

CONCLUSION

A horizontal relationship between patients and health professionals enables person-centred care to be delivered, in which that patient is considered a protagonist in the decisions made on his or her health. This has been recognised by several governmental organisations and incorporated into discourse and strategies. However, the practical application of this model is an area in which progress is still to be made. The handbook and clinical practice guide therefore aim to familiarise professionals with the model, helping them to engage women in the decision of either having BC screening or not. The results obtained enable us to conclude that, to apply it as a public policy, first there must be a pilot study with health professionals, which should be supplemented by formal training in SDM.

ACKNOWLEDGEMENTS

We would like to express our gratitude to the three expert reviewers in Shared Decision-Making and breast cancer: Victor Montori, Lilisbeth Perestelo-Pérez and Montserrat Rué; as well as the external reviewers, Lluís Colomés Figuera and Josep Maria Sabaté. We would also like to thank the 20 participants in the study who spent their time, effort and perseverance into answering all the rounds of questions the research team had posed them.

FUNDING

Financial support for this study was provided entirely by a grant from Instituto de Salud Carlos III through the project PI18/00773 (co-funded by the European Regional Development Fund), and by the European Union's Horizon 2020 research and innovation programme, under Marie Skłodowska-Curie grant agreement No 713679 from *Universitat Rovira i Virgili* (URV). The funding agreement ensured the authors' independence in designing the study, interpreting the data, and writing and publishing the report.

DECLARATION OF CONFLICTS OF INTEREST

The authors declare that they have no conflict of interest.

CONTRIBUTORSHIP STATEMENT

For peer review only - http://bmjopen.bmj.com/site/about/guidelines.xhtml

Authors MJHL, MJPL and MCL designing of work, data analysis, interpretation, manuscript writing and obtaining funding. Also, NCB and AC collaborate in designing work, interpretation and collecting data. Author CVL contribution of patients or study material. All authors make a critical review of the manuscript and adoption of the final version.

DATA AVAILABILITY STATEMENT

All data relevant to the study are included in the article or uploaded as supplementary information

REFERENCES

1. Hargraves IG, Montori VM, Brito JP, Kunneman M, Shaw K, LaVecchia C, Wilson M, Walker L, Thorsteinsdottir B. Purposeful SDM: A problem-based approach to caring for patients with shared decision making. Patient Educ Couns. 2019;102(10):1786-1792. doi: 10.1016/j.pec.2019.07.020. Epub 2019 Jul 19. PMID: 31353170; PMCID: PMC6717012.
2. Munthe C, Sandman L, Cutas D. Person Centred Care and Shared Decision Making: Implications for Ethics, Public Health and Research. Health Care Anal [Internet]. 2012;20(3):231–49. Available from: http://link. springer.com/10.1007/s10728-011-0183-y
3. Charles C, Gafni A, Whelan T. Decision-making in the physician-patient encounter: revisiting the shared treatment decision-making model. Soc Sci Med [Internet]. 1999;49(5):651–61. Available from: http://www.ncbi.nlm.nih.gov/pubmed/10452420
4. Sandman L, Granger BB, Ekman I, Munthe C. Adherence, shared decision-making and patient autonomy. Med Health Care Philos. 2012;15(2):115-27. doi: 10.1007/s11019-011-9336-x. PMID: 21678125.
5. Boletín Oficial del Estado. Ley 14/1986 de 2 de febrero, General de Sanidad. BOE de 21/2000 [Official State Gazette. Law 14/1986 of 2 February, General Health. BOE of 21/2000]. Available from: https://www.boe.es/boe/dias/2001/02/02/pdfs/A04121-04125.pdf
6. Toledo-Chavarri A, Rué M, Codern-Bove N, Carles-Lavila M, Perestelo-Perez L, Perez-Lacasta MJ, and Feijoo-Cid M. A Qualitative Study on a Decision Aid for Breast Cancer Screening: Views from Women and Health Professionals. Eur J Cancer Care (Engl) [Internet]. 2017:26(3). Available from: https://onlinelibrary.wiley.com/doi/abs/10.1111/ecc.12660
7. Yu L, Yang S, Zhang C, Guo P, Zhang X, Xu M, Tian Q, Cui X, Zhang W, Fan S. Decision aids for breast cancer screening in women approximately 50 years of age: A systematic review and meta-analysis of randomised controlled trials. J Clin Nurs. 2021. doi: 10.1111/jocn.16112. Epub ahead of print. PMID: 34738288.
8. Generalitat de Catalunya. Departament de Salut. Pla director 2017. [Master plan 2017]. Available from:http://salutweb.gencat.cat/ca/ambits_actua-cio/linies_dactuacio/estrategies_salut/cancer/pla_director/
9. Generalitat de Catalunya. Departament de Salut. Pla director D'Oncologia 2016-2020: Un sistema centrat en la persona: públic, universal i just [Master Oncology Plan 2016-2020: A person-centred system: Public, universal and fair]. 2016. [Internet]. Available from: http://salutweb.gencat.cat/web/.con-tent/_departament/pla-de-salut/Pla-de-salut-2016-2020/documents/ Pla_salut_Catalunya_2016_2020.pdf
10. Institut Català d`Oncologia. Detecció Precoç de Càncer de Mama [Early detection of breast cancer]. [Internet]. 2020 [cited 30 June 2020]. Available from: http://ico.

For peer review only - http://bmjopen.bmj.com/site/about/guidelines.xhtml

gencat.cat/ca/el-cancer/programes_de_deteccio_precoc/programa_de_deteccio_precoc_de_cancer_de_mama/index.html
11. Légaré F, Witteman H. Shared decision making: examining key elements and barriers to adoption into routine clinical practice. Health Aff (Mill- wood). [Internet]. 2013;32(2):276-84 Available from: https://www. healthaffairs.org/doi/pdf/10.1377/hlthaff.2012.1078
12. Charles C, Gafni A, Whelan T. Self-reported use of shared decision-making among breast cancer specialists and perceived barriers and facilitators to implementing this approach. Health Expect [Internet]. 2004;7(4):338–48. Available from: http://www.ncbi.nlm.nih.gov/pubmed/15544686
13. Hernández-Leal MJ, Pérez-Lacasta M, Feijoo-Cid M, Ramos-García V, Carles-Lavila M. Healthcare professionals behaviour to implement in shared decision making in screening programs: A systematic review. Patient Educ Couns. 2021. doi: https://doi.org/10.1016/j.pec.2021.01.032
14. Álvarez-Torres G. Capítulo 2 Los Manuales [Chapter 2: The Manuals]. En: Manual para elaborar manuales de políticas y procedimientos [Manual for developing policy and procedures manuals]. Mexico D.F.: Panorama editorial, S.A; 1996. p. 23-26.
15. Hernández-Leal MJ, Carles-Lavila M, Pérez-Lacasta M. La participación de los profesionales de la salud en la Toma de Decisiones Compartida en el cribado de cáncer de mama: Manual de apoyo para implementación de la TDC [The participation of health professionals in Shared Decision-Making in breast cancer screening: A handbook to support the implementation of SDM]. Spain: María José Hernández editor; 2020.
16. Croes KD, Jones NR, DuBenske LL, Schrager SB, Mahoney JE, Little TA, Burnside ES. Core Elements of Shared Decision-making for Women Considering Breast Cancer Screening: Results of a Modified Delphi Survey. J Gen Intern Med. 2020;35(6):1668-1677. doi: 10.1007/s11606-019-05298-8. Epub 2020 Mar 19. PMID: 32193817; PMCID: PMC7280383.
17. Elwyn G, Duran M, Song J, Aarts J, J Barr P, Berger Z, etc al. A three-talk model for shared decision making: multistage consultation process. BMJ. 2017;359. [Internet]. Available from:https://www.bmj.com/con-tent/359/bmj.j4891.long
18. Barrett D, Heale R. What are Delphi studies? Evid Based Nurs. 2020 Jul;23(3):68-69. doi: 10.1136/ebnurs-2020-103303. Epub 2020:19. PMID: 32430290.
19. Reguant-Álvarez M. y Torrado-Fonseca M. El método Delphi [The Delphi method]. Revista d'Innovació i Recerca en Educació. Universidad de Barcelona. Institut de Ciéncies de P´Educació. 2016:9(1). Available from: https://revistes.ub.edu/index.php/REIRE/article/view/reire2016.9.1916
20. López-Gómez, E. El método Delphi en la investigación actual en educación: una revisión teórica y metodológica [The Delphi method in current education research: A theoretical and methodological review]. Educación XX1. 2018;21(1):17-40
21. Benner P, Lee P, Stannard D. (1999). Clinical wisdom in critical care: a thinking- in-action approach. Saunders.
22. García-Ruiz M, Lena-Acebo F. Aplicación del metodo delphi en el diseño de una investigación cuantitativa sobre el fenómeno FABLAB [Application of the Delphi method to the design of quantitative research on the FABLAB phenomenon]. Empiria. Revista de metodología de ciencias sociales, 2018:40;129-166. doi:https://doi.org/10.5944/empiria.40.2018.22014.
23. Martínez-Piñeiro M. La técnica Delphi como estrategia de consulta a los implicados en la evaluación de programas [The Delphi technique as a strategy for consulting

For peer review only - http://bmjopen.bmj.com/site/about/guidelines.xhtml

participants in the evaluation of programmes]. RIE. 2003; 21(2): 449-463. Available from: https://revistas.um.es/rie/article/view/99311/94911
24. The SHARE Approach—Essential Steps of Shared Decision Making: Quick Reference Guide. 2014. [Internet]. Available from: https://www.ahrq.gov/health-literacy/curriculum-tools/shareddecisionmaking/tools/tool-1/index.html
25. De las Cuevas C, Perestelo-Perez L, Rivero-Santana A, Cebolla-Martí A, Scholl I, Härter M. Validation of the Spanish version of the 9-item Shared Decision-Making Questionnaire. Health Expect. 2015; 18(6):2143-53. doi: 10.1111/hex.12183. Epub 2014 Mar 5. PMID: 24593044; PMCID: PMC5810694.
26. Shepherd HL, Barratt A, Trevena LJ, McGeechan K, Carey K, Epstein RM, Butow PN, Del Mar CB, Entwistle V, Tattersall MH. Three questions that patients can ask to improve the quality of information physicians give about treatment options: a cross-over trial. Patient Educ Couns. 2011;84(3):379-85. doi: 10.1016/j.pec.2011.07.022. Epub 2011 Aug 9. PMID: 21831558.
27. Elwyn G, Barr PJ, Grande SW, Thompson R, Walsh T, Ozanne EM. Developing CollaboRATE: a fast and frugal patient-reported measure of shared decision making in clinical encounters. Patient Educ Couns. 2013;93(1):102-7. doi: 10.1016/j.pec.2013.05.009. Epub 2013 Jun 12. PMID: 23768763.
28. Martínez KA, Deshpande A, Ruff AL, Bolen SD, Teng K, Rothberg MB. Are Providers Prepared to Engage Younger Women in Shared Decision-Making for Mammography? J Womens Health (Larchmt). 2018;27(1):24-31. DOI: 10.1089/jwh.2016.6047. Epub 2017 Jun 28. PMID: 28657820.
29. Universidad Complutense de Madrid. Guía Docente 2015-2016 [Teaching guide 2015-2016]. Facultad de Medicina. Available from: https://medicina.ucm.es/data/cont/media/www/pag-17227/Gu%C3%ADa%20Medicina%202015-2016.pdf
30. Universitat Autónoma de Barcelona. Programa Bioética y Comunicación 2020/2021 [Bioethics and Communication Programme 2020/2021]. Available from: https://guies.uab.cat/guies_docents/public/portal/html/2020/assignatura/102950/es
31. Complejo Hospitalario Universitario de Badajoz (CHUB). Guía Docente Medicina Intensiva CHUB [CHUB Intensive Care Medicine Teaching Guide]. 2020. Available from: https://www.areasaludbadajoz.com/docencia_investigacion/comision_docencia/Guias_e_Itinerarios_Formativos/Guia_formativa_UCI.pdf
32. Ministerio de Ciencia e Innovación de España. Orden CIN/2134/2008, de 3 de julio, por la que establecen los requisitos para la verificación de los títulos universitarios oficiales que habilitan para el ejercicio de la profesión de Enfermero [Order CIN/2134/2008 of 3 July, establishing the requirements for verification of the official university certificates that act as qualifications for practising the profession of Nurse]. Agencia Estatal Boletín Oficial del Estado. 2008 (174). Available from: https://www.boe.es/eli/es/o/2008/07/03/cin2134
33. Légaré F, Adekpedjou R, Stacey D, Turcotte S, Kryworuchko J, Graham ID, et. al. Interventions for increasing the use of shared decision making by healthcare professionals. Cochrane Database Syst Rev. 2018;7(7):CD006732. DOI: 10.1002/14651858.CD006732.pub4. PMID: 30025154; PMCID: PMC6513543.
34. Dois A, Bravo P, Contreras A, Soto MG, Mora I. Formación y competencias para los equipos de atención primaria desde la mirada de expertos chilenos [Training and competencies for primary care teams from the perspective of Chilean experts; Treinamento e competências para equipes de atenção primária na perspectiva de

For peer review only

especialistas chilenos]. Rev Panam Salud Publica. 2018 Sep 24;42:e147. Spanish. doi: 10.26633/RPSP.2018.147. PMID: 31093175; PMCID: PMC6386070.
35. García-Altés A, Peiró M, Artells JJ. Priorización de medidas para la consolidación de la toma de decisiones compartidas en las prestaciones del Sistema Nacional de Salud español [Prioritising measures for consolidating shared decision-making in the services rendered by the Spanish National Health System]. Gac Sanit. 2019;33(5):408-414. Spanish. doi: 10.1016/j.gaceta.2018.04.017. Epub 2018 Jul 20. PMID: 30033094.
36. Berkhof M, van Rijssen HJ, Schellart AJ, Anema JR, van der Beek AJ. Effective training strategies for teaching communication skills to physicians: an overview of systematic reviews. Patient Educ Couns. 2011;84(2):152-62. doi: 10.1016/j.pec.2010.06.010. Epub 2010 Jul 29. PMID: 20673620.
37. Perestelo-Perez L, Rivero-Santana A, Perez-Ramos J, Gonzalez-Lorenzo M, Roman JG, Serrano-Aguilar P. Shared decision making in Spain: current state and future perspectives. Z Evid Fortbild Qual Gesundhwes. 2011;105(4):289-95. doi: 10.1016/j.zefq.2011.04.013. Epub 2011 Apr 30. PMID: 21620323.
38. Grupo de trabajo de implicación de pacientes en el desarrollo de GPC. Implicación de Pacientes en el Desarrollo de Guías de Práctica Clínica: Manual Metodológico [Involvement of Patients in the Development of Clinical Practice Guides: Methodological Handbook]. Ministerio de Sanidad, Servicios Sociales e Igualdad. Instituto Aragonés de Ciencias de la Salud-IACS. Guías de Práctica Clínica en el SNS: IACS No 2010/01.
39. The Ottawa Hospital. Patient Decision Aids. Available from: https://decisionaid.ohri.ca/AZsearch.php?criteria=screening

For peer review only

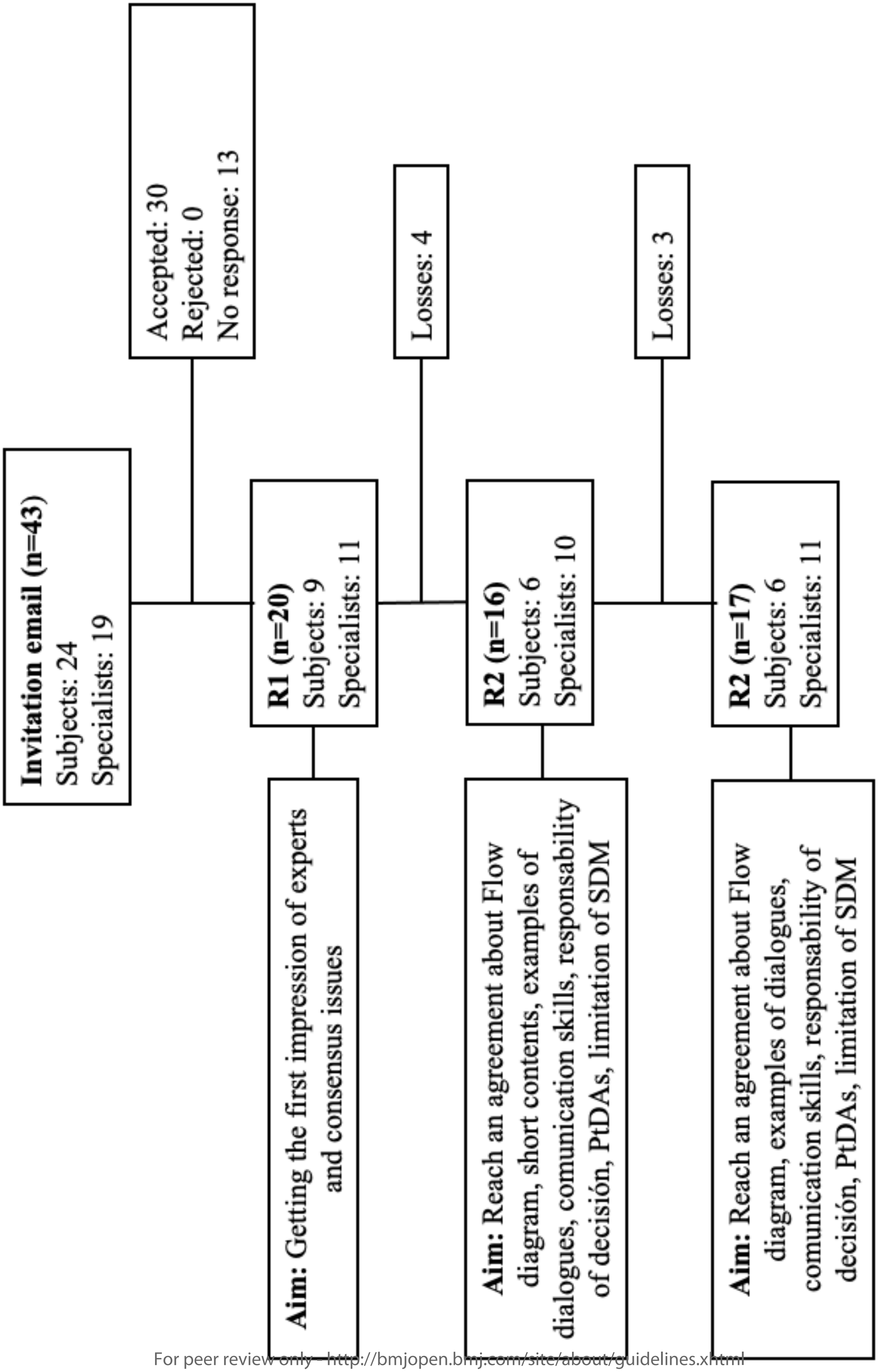
Invitation email (n=43)
Subjects: 24
Specialists: 19
Accepted: 30
Rejected: 0
No response: 13
R1 (n=20)
Subjects: 9
Specialists: 11
Aim: Getting the first impression of experts and consensus issues
Losses: 4
R2 (n=16)
Subjects: 6
Specialists: 10
Aim: Reach an agreement about Flow diagram, short contents, examples of dialogues, comunication skills, responsability of decisión, PtDAs, limitation of SDM
Losses: 3
R2 (n=17)
Subjects: 6
Specialists: 11
Aim: Reach an agreement about Flow diagram, examples of dialogues, comunication skills, responsability of decisión, PtDAs, limitation of SDM

Figura 5: Flujograma del programa de Detección Precoz del Cáncer de Mama

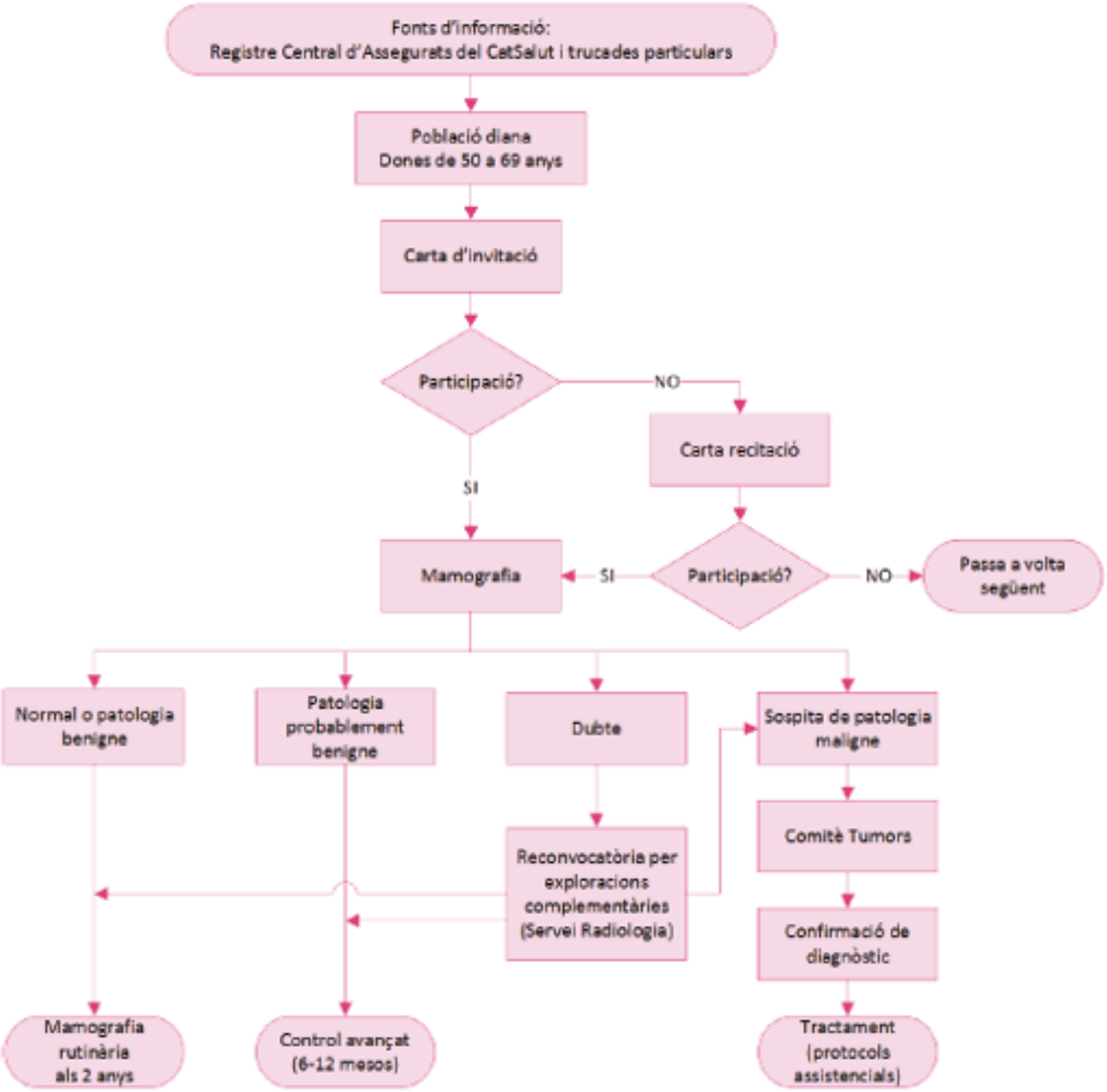

Figure 2 (Annex 4): the clarity and usefulness of this figure was not agreed; therefore, it will be eliminated according to the results of the R3.

First version: Prior to discussion with experts (Annex 4)

ÍNDICE

OBJETIVO DEL MANUAL	6
¿A QUIÉN VA DIRIGIDO?	6
**Introducción**	**7**
**Toma de decisiones compartida**	**13**
1. ¿Qué es?	14
2. ¿Por qué es importante?	17
3. ¿Qué habilidades o competencias necesitan los profesionales sanitarios?	19
4. ¿Qué opinan los pacientes?	22
**La toma de decisiones compartida en el cribado de cáncer de mama**	**25**
1. El Programa de cribado	26
2. Implementación de la TDC en cribado de cáncer de mama	31
3. Autoevaluación del proceso de TDC	40
**Referencias bibliográficas**	**45**

Final version: With the suggestion by the experts (Annex 2)

ÍNDICE

OBJETIVO DEL MANUAL	6
¿A QUIÉN VA DIRIGIDO?	6
**Introducción**	**7**
**Toma de decisiones compartida**	**11**
1. ¿Qué es?	12
2. ¿Por qué es importante?	15
3. Las limitaciones del modelo	17
4. ¿Qué habilidades o competencias necesitan los profesionales sanitarios?	18
5. ¿Qué opinan los pacientes?	22
**La toma de decisiones compartida en el cribado de cáncer de mama**	**25**
1. El Programa de cribado	26
2. Implementación de la TDC en cribado de cáncer de mama	30
3. Autoevaluación del proceso de TDC	39
**Referencias bibliográficas**	**43**

First version: Prior to discussion with experts (Annex 5)	Final version: With the suggestion by the experts (Annex 3)
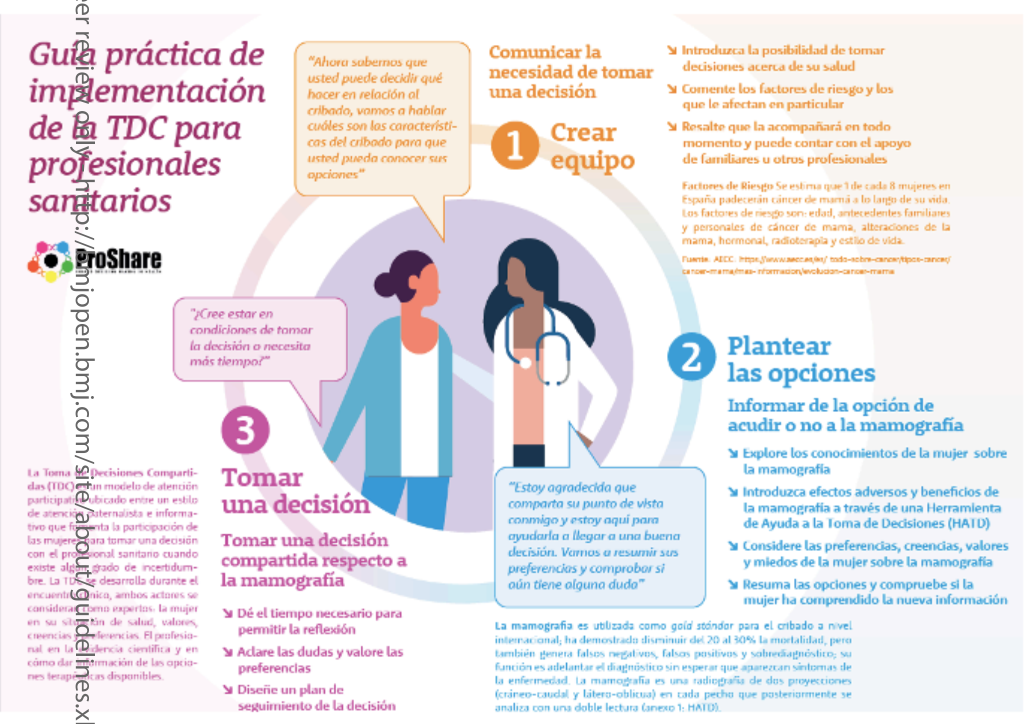	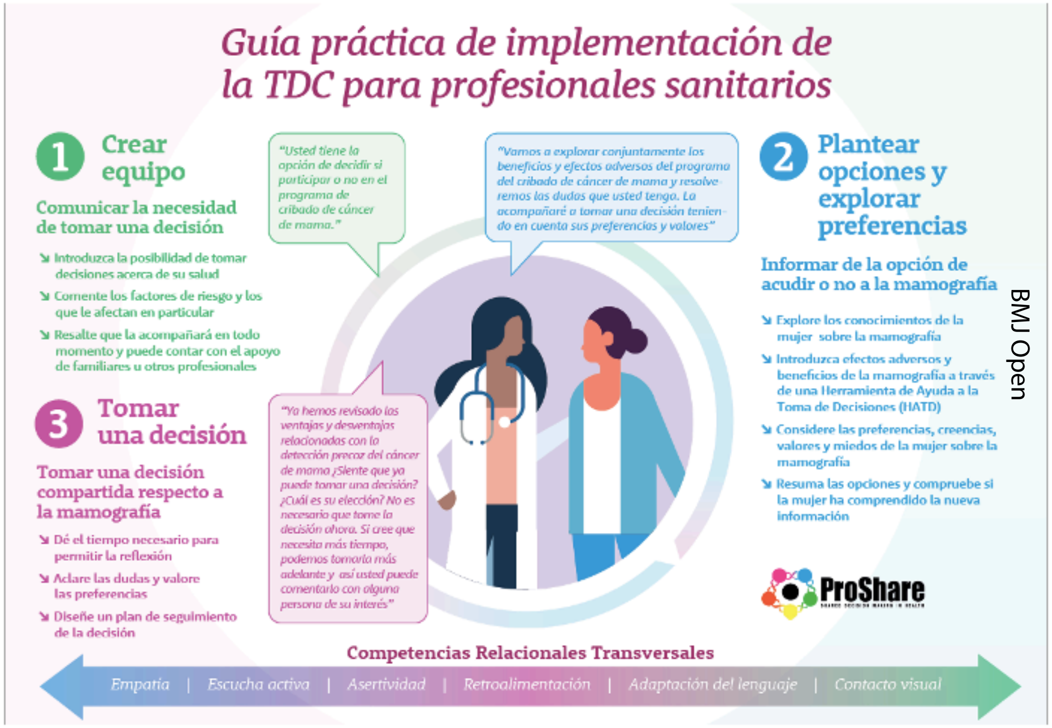

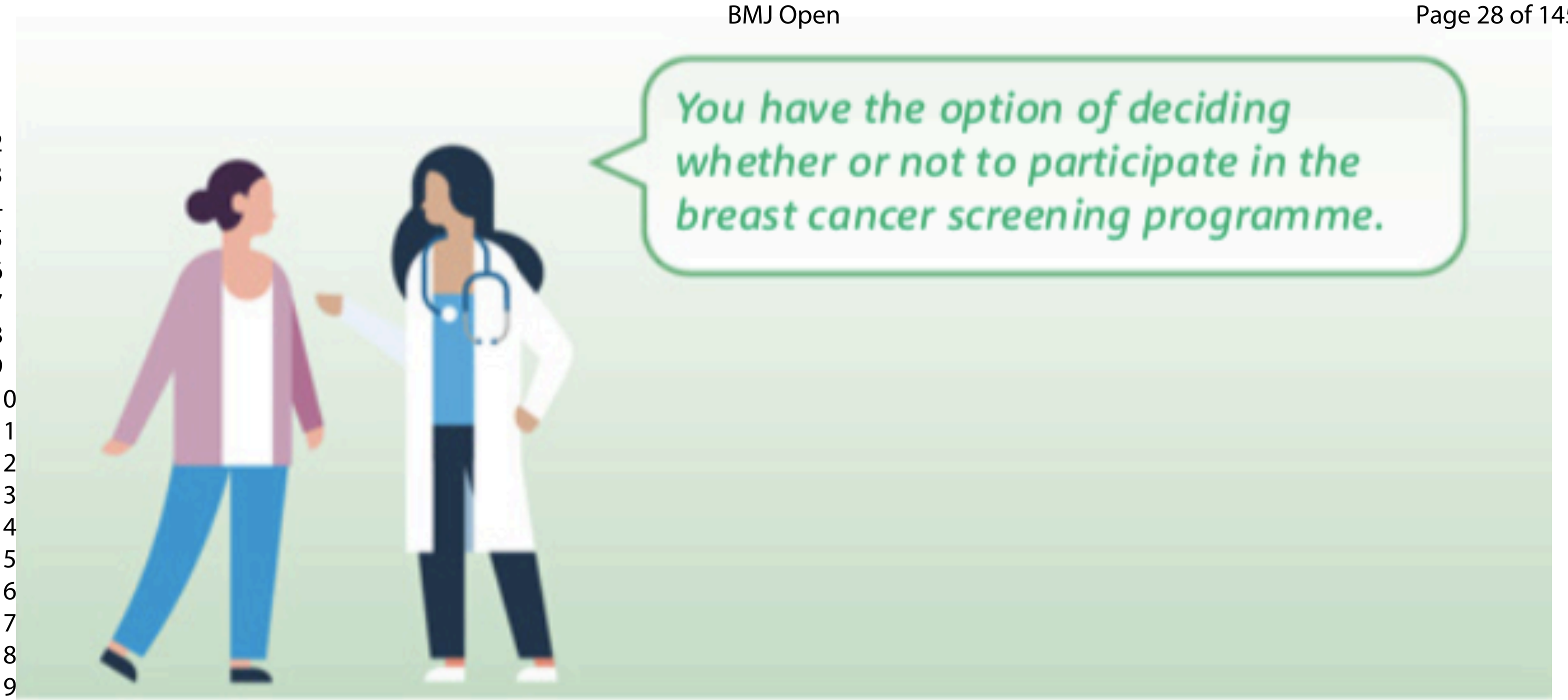
You have the option of deciding whether or not to participate in the breast cancer screening programme.

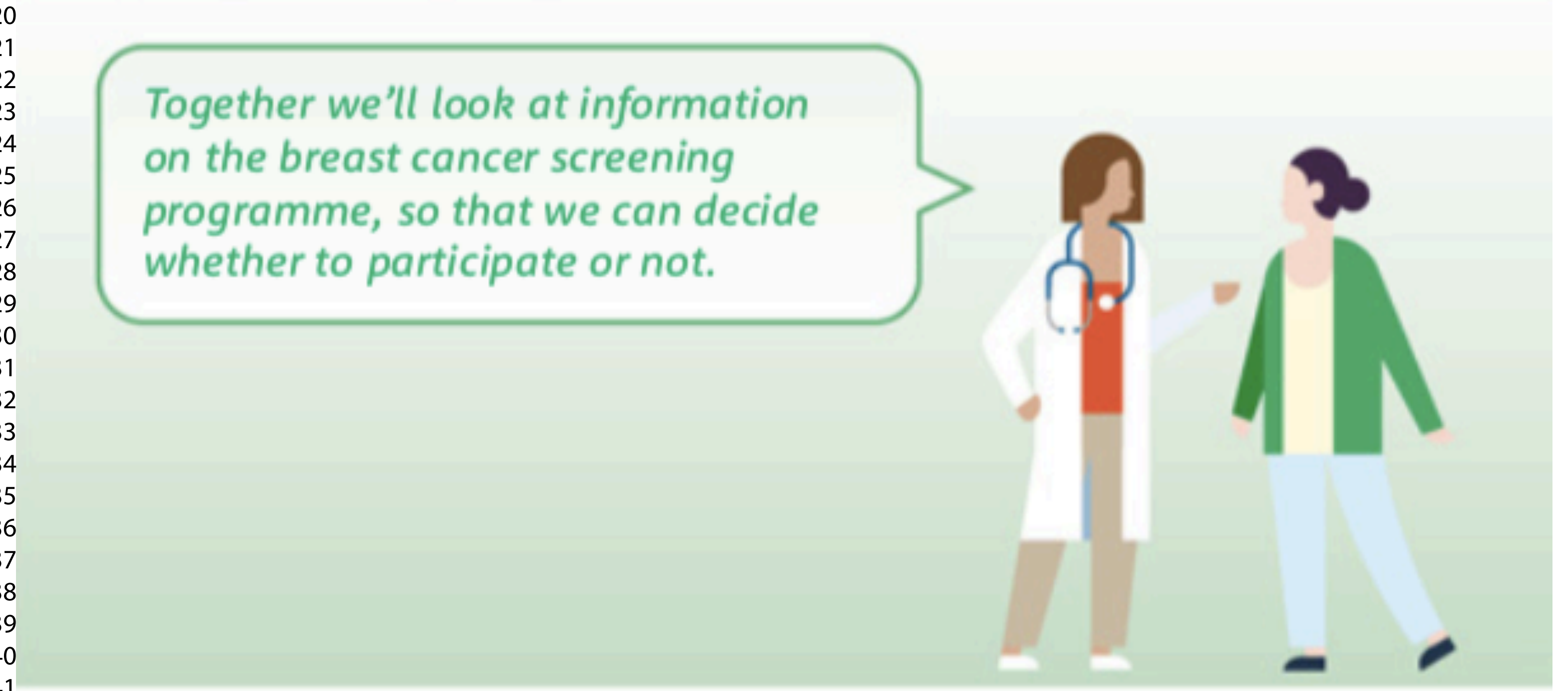
Together we'll look at information on the breast cancer screening programme, so that we can decide whether to participate or not.

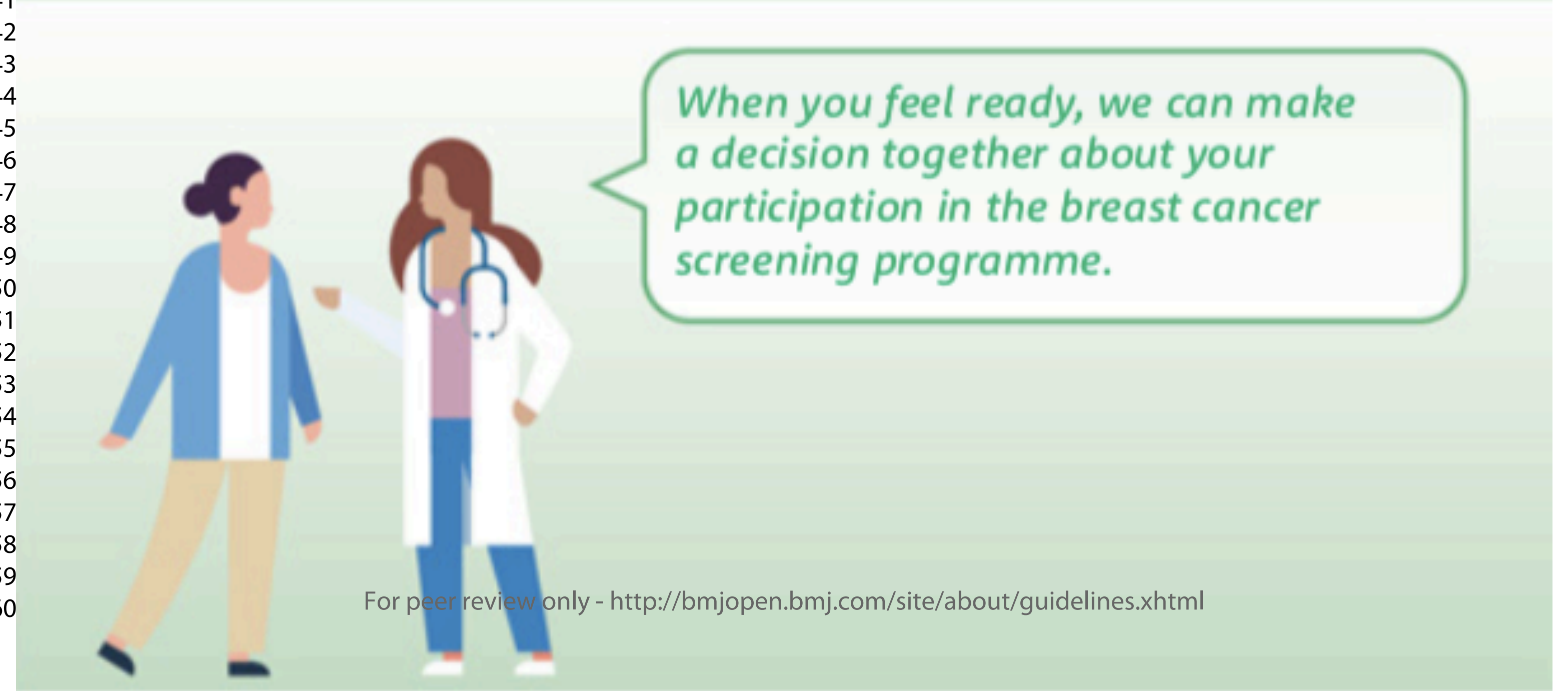
When you feel ready, we can make a decision together about your participation in the breast cancer screening programme.

NO OLVIDES QUE...

La mamografía no evita que tengas cáncer de mama. Además, no es un método perfecto; algunos tumores son muy difíciles de ver en una mamografía.

Puede ser que no tengas cáncer. Pero si lo tuvieras, el diagnóstico y tratamiento en una fase inicial del tumor puede suponer una mayor probabilidad de supervivencia.

Aunque te hayas hecho una mamografía recientemente, es importante que si notas algún cambio en el pecho vayas al médico.

La información presentada en este folleto se ha basado en artículos científicos y materiales desarrollados por el Programa de Cribado de Cáncer de Mama del National Health Service en Inglaterra, por la Colaboración Cochrane y por programas de cribado de Cataluña.

GOBIERNO DE ESPAÑA
MINISTERIO DE ECONOMÍA Y COMPETITIVIDAD
Instituto de Salud Carlos III
Unión Europea
Fondo Europeo de Desarrollo Regional
"Una manera de hacer Europa"

Estudio PI14/00113 Participación de las mujeres en las decisiones y estrategias de detección precoz del cáncer de mama. Cofinanciado por el Instituto de Salud Carlos III y fondos FEDER de la Unión Europea. Participan: Institut de Recerca Biomèdica de Lleida-Universitat de Lleida, Universitat Rovira i Virgili, Institut Català d'Oncologia, Hospital del Mar y Servicio Canario de Salud.

BENEFICIOS Y EFECTOS ADVERSOS A LARGO PLAZO DE LA DETECCIÓN PRECOZ DEL CÁNCER DE MAMA

Si un grupo de 200 mujeres entre 50 y 69 años se hacen mamografías de cribado cada 2 años, cuando cumplan 80 años...

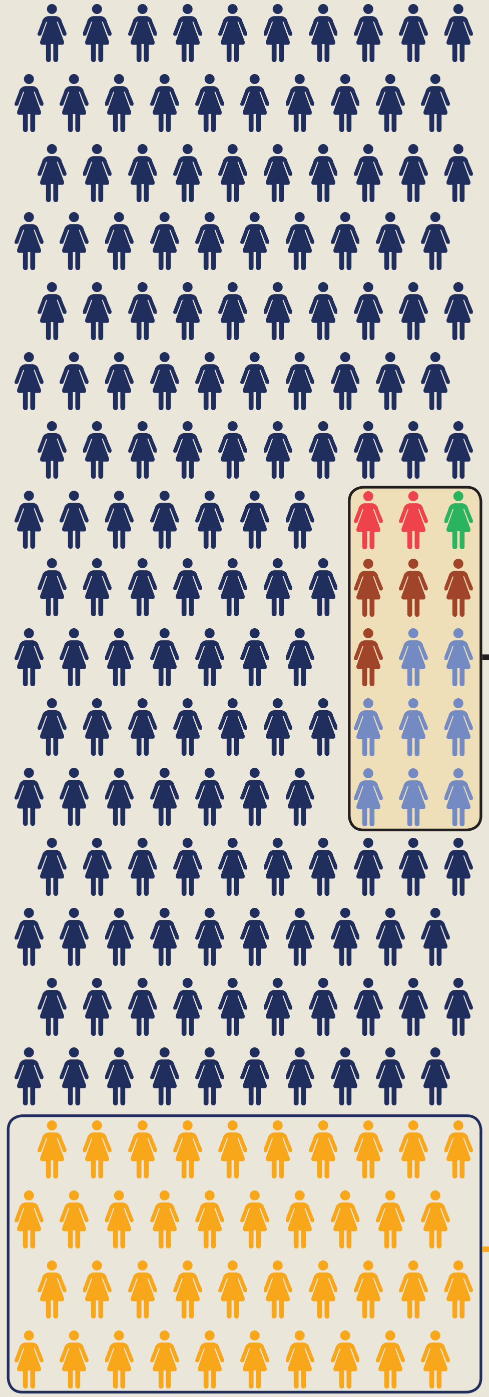

15 mujeres habrán sido diagnosticadas de cáncer de mama

BENEFICIOS DEL CRIBADO

1 sobrevivirá al cáncer de mama
Habría fallecido sin el cribado
Los tratamientos de algunas mujeres pueden ser menos agresivos

CON O SIN CRIBADO

4 habrán muerto de cáncer de mama a pesar de haber participado en el cribado

8 habrán sido diagnosticadas y sobrevivirán
Habrían sobrevivido aunque no se hubieran cribado

EFECTOS ADVERSOS DEL CRIBADO

2 serán diagnosticadas y tratadas de un cáncer de mama
Este cáncer nunca habría sido una amenaza para su vida

40 habrán necesitado pruebas adicionales
Posteriormente se descartará que hubieran tenido cáncer de mama (falsos positivos)

Por cada muerte evitada por el programa de cribado, 2 mujeres son diagnosticadas y tratadas de un cáncer que nunca hubiera puesto en riesgo su vida.

For peer review only - http://bmjopen.bmj.com/site/about/guidelines.xhtml

LA DETECCIÓN PRECOZ DEL CÁNCER DE MAMA

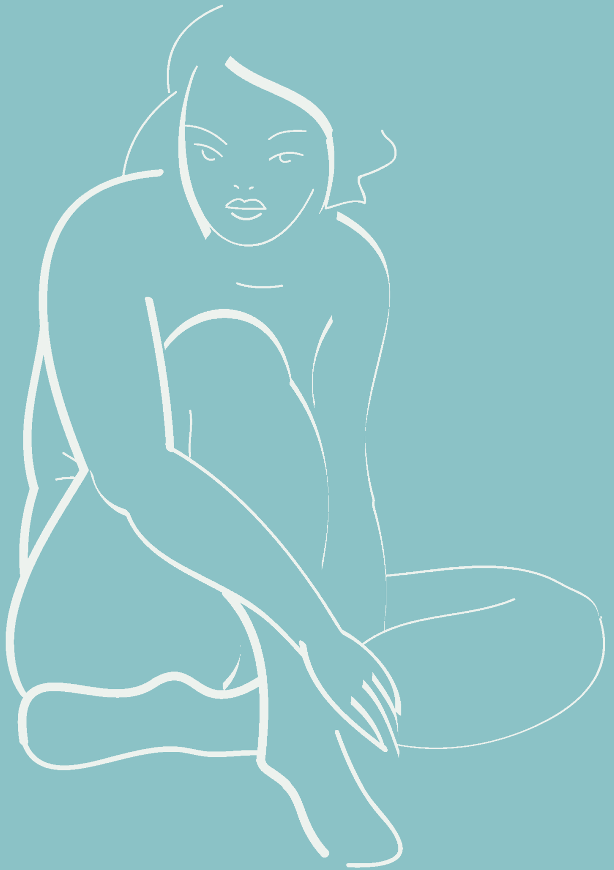

AYUDÁNDOTE A DECIDIR

PARTICIPAR O NO PARTICIPAR EN EL CRIBADO DEL CÁNCER DE MAMA: ESTA ES LA CUESTIÓN

Estudios científicos recientes han identificado efectos adversos, antes desconocidos, de la detección precoz de cáncer de mama mediante mamografía. Por esta razón, este folleto tiene como objetivo informar sobre los beneficios y efectos adversos de participar en la detección precoz de cáncer de mama.

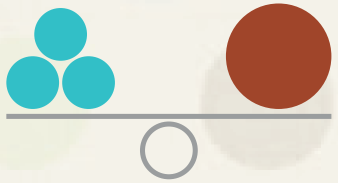

Este material informativo pretende ayudarte a sopesar pros y contras para que puedas tomar una decisión personal sobre si deseas participar o no en la detección precoz del cáncer de mama, en función de tus valores y preferencias.

¿QUÉ ES EL CÁNCER DE MAMA?

El cáncer de mama se desarrolla cuando algunas células empiezan a crecer de forma descontrolada, formando un tumor. A medida que el tumor crece las células malignas se pueden desplazar a otras partes del cuerpo y poner en peligro la vida de la persona afectada.

En Cataluña se diagnostican unos 4.000 casos nuevos de cáncer de mama al año. Las estadísticas nos dicen que 1 de cada 9 mujeres padecerá cáncer de mama a lo largo de su vida y que el 83% de las mujeres afectadas sobrevivirán a esta enfermedad.

¿QUÉ ES LA DETECCIÓN PRECOZ DEL CÁNCER DE MAMA?

La detección precoz del cáncer de mama, también denominada cribado, tiene por objetivo detectar un cáncer de mama en una etapa muy inicial, antes de que aparezcan síntomas. En su etapa inicial, el cáncer es más fácil de tratar y las oportunidades de sobrevivir son superiores.

El sistema sanitario público ofrece la posibilidad de participar en la detección precoz del cáncer de mama con el objetivo de reducir la mortalidad causada por este tumor. El programa de cribado se dirige a las mujeres entre 50 y 69 años y consiste en realizar una mamografía cada dos años.

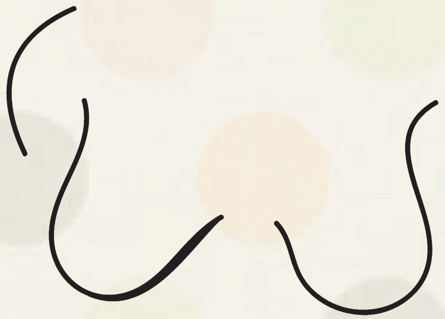

La mamografía es una radiografía de la mama. Es la prueba más eficaz para detectar el cáncer de mama en mujeres que no presentan síntomas. El riesgo de algún daño por la exposición a esta radiación es muy pequeño.

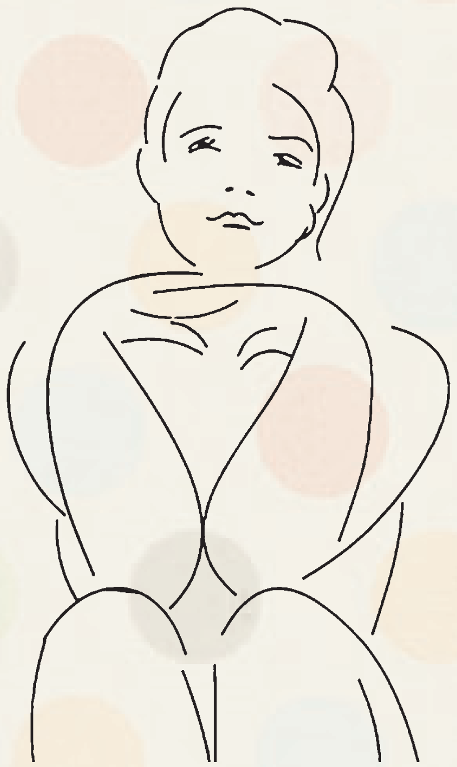

BENEFICIOS DEL CRIBADO

El cribado reduce el riesgo de morir por cáncer de mama

La detección precoz puede salvar la vida a algunas mujeres porque se diagnostican y tratan antes de lo que se habría hecho sin cribado.

De cada 200 mujeres que se realizan mamografías de cribado cada dos años, entre los 50 y los 69 años, 1 mujer se salva gracias a la detección precoz del tumor.

El cribado detecta el cáncer en estadios más iniciales

Un cáncer detectado en estadios iniciales no necesita tratamientos tan agresivos como cuando está más avanzado; estos tratamientos tienen menos efectos secundarios y la probabilidad de recuperación es más alta.

EFECTOS ADVERSOS DEL CRIBADO

Errores en el diagnóstico: falsos positivos y falsos negativos

Los falsos positivos se producen cuando los resultados de la mamografía hacen sospechar de un posible cáncer de mama que en realidad no existe. Esto conlleva exploraciones adicionales que no serían necesarias.

De cada 200 mujeres que se realizan mamografías de cribado cada dos años entre los 50 y los 69 años, 40 tendrán un resultado falso positivo.

La situación contraria, el falso negativo, es mucho menos frecuente y se puede producir cuando la mamografía no muestra ninguna señal de cáncer de mama, aunque la mujer lo padezca.

El cribado puede detectar tumores inofensivos

Algunos tipos de cáncer que se detectan mediante la mamografía de cribado crecen tan lentamente que nunca hubieran llegado a ser un problema de salud. Algunos, incluso, habrían desaparecido de forma espontánea, sin tratamiento.

Actualmente no se puede saber qué lesiones progresarían y cuáles no, y por tanto, se ofrece tratamiento a todas las mujeres diagnosticadas. Algunas mujeres pueden recibir tratamientos que tienen efectos secundarios importantes, sin necesitarlos. Esto se conoce como **sobrediagnóstico** y **sobretratamiento**.

De cada 200 mujeres que se realizan mamografías de cribado cada dos años, entre los 50 y los 69 años, 2 serán tratadas de cáncer sin necesidad.

For peer review only - http://bmjopen.bmj.com/site/about/guidelines.xhtml

La participación de los profesionales de la salud en la Toma de Decisiones Compartida en el cribado de cáncer de mama

Manual de apoyo para implementación de la Toma de Decisiones Compartida

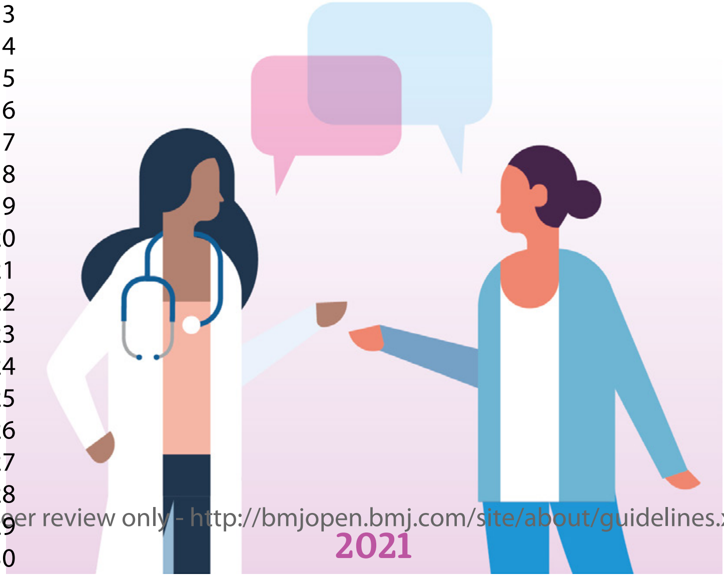

r peer review only - http://bmjopen.bmj.com/site/about/guidelines.xhtr

2021

For peer review only

For peer review only - http://bmjopen.bmj.com/site/about/guidelines.xhtml

La participación de los profesionales de la salud en la Toma de Decisiones Compartida en el cribado de cáncer de mama

Manual de apoyo para implementación de la Toma de Decisiones Compartida

2021

Unión Europea
Fondo Europeo de Desarrollo Regional
"Una manera de hacer Europa"
MINISTERIO DE CIENCIA, INNOVACIÓN Y UNIVERSIDADES
Instituto de Salud Carlos III
UNIVERSITAT ROVIRA i VIRGILI
ProShare

For peer review only - http://bmjopen.bmj.com/site/about/guidelines.xhtml

AUTORES

María José Hernández-Leal[1,a,b,c], María José Pérez-Lacasta[2,a,c], Misericòrdia Carles-Lavila[3,a,b,c] on behalf of the ProShare Group.

1. PhD student, Msc and Bsc Nursing. Mail: mariajose.hernandez@urv.cat
2. PhD, Mail: mariajose.perez@urv.cat
3. PhD. Mail: misericordia.carles@urv.cat

a. Department of Economics, Universitat Rovira i Virgili.
b. Centre de Recerca en Economia i Sostenibilitat (ECO-SOS).
c. Grup de Recerca en Anàlisi Estadístic i Econòmic en Salut (GRAEES).

Equipo de investigación ProShare:

Misericòrdia Carles Lavila
Núria Codern-Bové
María José Hernández Leal
María José Pérez Lacasta
Roger Pla
Vanesa Ramos García
Carmen Vidal

Cómo citar este documento:

Hernández-Leal MJ, Carles-Lavila M, Pérez-Lacasta M. La participación de los profesionales de la salud en la Toma de Decisiones Compartida en el cribado de cáncer de mama: Manual de apoyo para implementación de la TDC. España: María José Hernández editor; 2021.

For peer review only - http://bmjopen.bmj.com/site/about/guidelines.xhtml

For peer review only

EVALUADORES EXTERNOS

Dra. Lilisbeth Perestelo-Pérez
Servicio de Evaluación y Planificación.
Dirección del Servicio Canario de la Salud, España.
Red de Investigación en Servicios de Salud en Enfermedades Crónicas (REDISSEC).

Dr. Victor Montori
Knowledge and Evaluation Research (KER) Unit.
Mayo Clinic - Minnesota, USA.

Dra. Montserrat Rué
Institut de Recerca Biomèdica de Lleida (IRBLLEIDA).
Departament de Ciències Mèdiques Bàsiques.
Universitat de Lleida, España.

DISEÑO

Miquel A. Fernández
www.mafsdisseny.com

FINANCIACIÓN

- The European Regional Development Fund (ERDF). European Union's Horizon 2020 research and innovation programme under the Marie Skłodowska-Curie grant agreement No. 713679 from the Universitat Rovira i Virgili (URV).
- Proyecto PI18/00773 "La colaboración de los profesionales sanitarios para incluir la toma de decisiones compartida en el programa de cribado de cáncer de mama" financiado por el Instituto de Salud Carlos III y cofinanciado por la Unión Europea (FEDER) "Una manera de hacer Europa"

For peer review only - http://bmjopen.bmj.com/site/about/guidelines.xhtml

For peer review only

For peer review only - http://bmjopen.bmj.com/site/about/guidelines.xhtml

ÍNDICE

OBJETIVO DEL MANUAL 6

¿A QUIÉN VA DIRIGIDO? 6

Introducción 7

Toma de decisiones compartida 11

1. ¿Qué es? 12

2. ¿Por qué es importante? 15

3. Las limitaciones del modelo 17

4. ¿Qué habilidades o competencias necesitan los profesionales sanitarios? 18

5. ¿Qué opinan los pacientes? 22

La toma de decisiones compartida en el cribado de cáncer de mama 25

1. El Programa de cribado 26

2. Implementación de la TDC en cribado de cáncer de mama 30

3. Autoevaluación del proceso de TDC 39

Referencias bibliográficas 43

For peer review only - http://bmjopen.bmj.com/site/about/guidelines.xhtml

OBJETIVO DEL MANUAL

Este manual pretende ser un documento de referencia que sirva de guía para el desarrollo e implementación de la **Toma de Decisiones Compartida (TDC)** en el cribado del cáncer de mama.

¿A QUIÉN VA DIRIGIDO?

A profesionales sanitarios de la comunidad autónoma de Catalunya relacionados con el cribado de cáncer de mama y que tengan contacto directo con las mujeres que deben participar en el programa.

For peer review only

For peer review only - http://bmjopen.bmj.com/site/about/guidelines.xhtml

Introducción

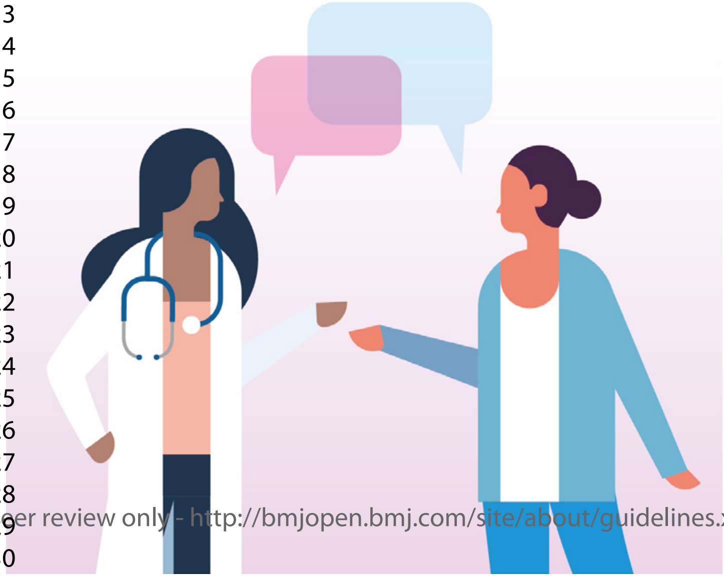

For peer review only - http://bmjopen.bmj.com/site/about/guidelines.xhtml

El objetivo del **cribado de cáncer de mama** es detectar tempranamente un tumor, en una fase preclínica. Esto permite mejorar las opciones de tratamiento y disminuir la mortalidad[1,2]. A pesar de estos beneficios, el cribado puede producir también efectos adversos: falsos negativos, falsos positivos, sobrediagnóstico y sobretratamiento[2,3,4]. Ante el desconocimiento del grado en que afectarán los efectos positivos y negativos a cada mujer en la decisión del cribado, el modelo de **Toma de Decisiones Compartida** (TDC) permite que pacientes y profesionales de la salud disminuyan la incertidumbre de la decisión[5,6].

La TDC ha sido utilizada principalmente en los países occidentales para mejorar las decisiones en salud de acuerdo a las preferencias de los pacientes y la evidencia científica[7]. Así, en 2012 The European Patients' Forum inició la campaña *"nothing about me, without me"* (nada sobre mí, sin mí)[8] para involucrar a las personas en las decisiones sobre su salud[9].

En este contexto, algunas investigaciones han explorado cómo se desarrolla la TDC, así, por ejemplo, en España solo el 24% de los pacientes afirma haber tomado la decisión conjunta con su profe-

For peer review only

For peer review only - http://bmjopen.bmj.com/site/about/guidelines.xhtml

sional sanitario considerando sus características y preferencias personales o sociales[10]. Esto evidencia la falta de estrategias para involucrar a las personas en su salud, debido a posibles deficiencias en las habilidades comunicativas y la inexistencia de vías de diálogo productivas entre los distintos actores del encuentro clínico[11].

Para mejorar las estrategias, la **Agencia de Calidad y Evaluación Sanitarias de Cataluña** (AQuAs) describe a las Herramienta de Ayuda a la Toma de Decisiones (HATD) como un elemento central para la TDC, aunque actualmente no dispone una de ellas para el cribado de cáncer de mama[12]. Sin embargo, un estudio reciente desarrolló una HATD[13].

A pesar de que en la actualidad ha habido un incremento de materiales destinados a las pacientes[14], son escasos aquellos que ayudan a los profesionales para incorporar las preferencias y los valores de las personas en la toma de decisiones en salud. En este sentido, este documento ofrece a los profesionales sanitarios evidencias científicas sobre la TDC para que sean aplicables al proceso de cribado de cáncer de mama.

For peer review only - http://bmjopen.bmj.com/site/about/guidelines.xhtml

For peer review only

Toma de decisiones compartida

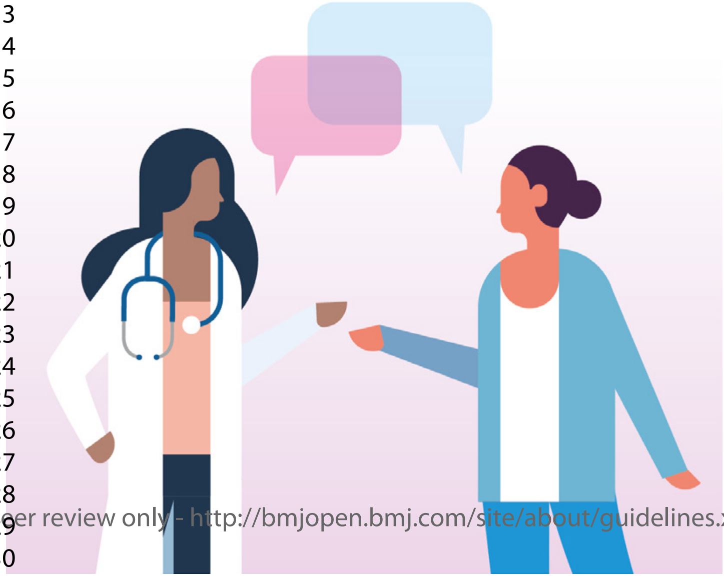

For peer review only - http://bmjopen.bmj.com/site/about/guidelines.xhtml

1. ¿Qué es?

La Toma de Decisiones Compartida (TDC) surge en la década de los 60-70 y corresponde a un modelo de atención participativo ubicado entre el estilo de atención paternalista y el informativo[15,16].

Figura 1: Modelos de atención en salud

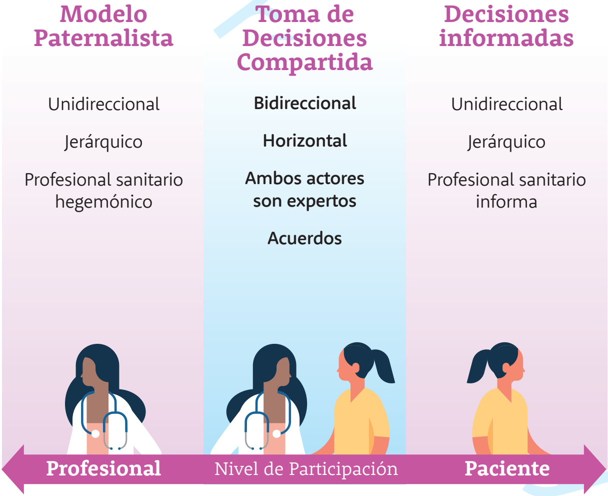

Fuente: Elaboración propia del grupo ProShare

For peer review only - http://bmjopen.bmj.com/site/about/guidelines.xhtml

La TDC fomenta la participación del paciente[17] para tomar una decisión conjunta con el profesional sanitario en relación con cambios en el estilo de vida, pruebas diagnósticas, tratamientos y acciones terapéuticas donde pueda existir algún grado de incertidumbre[18,19]. La TDC se desarrolla durante el encuentro clínico y ambos actores son considerados expertos: el paciente en su situación de salud, valores, creencias y preferencias; y el profesional sanitario en la evidencia científica e información de las opciones terapéuticas disponibles[20].

Figura 2: Rol de los participantes en el encuentro clínico

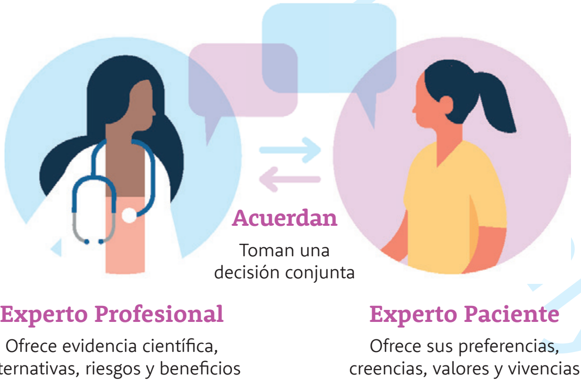

Fuente: Elaboración propia del grupo [illegible]

For peer review only - http://bmjopen.bmj.com/site/about/guidelines.xhtml

Por tanto, el objetivo de la TDC se centra en garantizar que las personas tomen decisiones sobre su salud cuando están suficientemente informadas[21]. Para lograrlo se requiere de una negociación continua entre ambos expertos centrando el diálogo en los valores, preferencias, circunstancias del paciente, así cómo en los beneficios, daños, riesgos y opciones terapéuticas ofrecidas por el profesional sanitario. Como resultado final de esta discusión se consigue personas más autónomas, un mayor nivel de compromiso y responsabilidad en su salud[20,22–24].

Figura 3: Elementos de la Toma de Decisiones Compartida

A) Intercambio de información entre el paciente y el profesional sanitario	B) Deliberación sobre las distintas opciones	**C) Tomar una decisión consensuada**

Fuente: Adaptación de Elwyn et al. 2012[20]

For peer review only

2. ¿Por qué es importante?

La TDC se sustenta en el principio de autonomía de los pacientes. La Ley 21/2000 Derechos de Información relativos a la Salud, Autonomía del Paciente y Documentación Clínica protege su derecho a decidir libremente después de recibir la información adecuada entre las opciones clínicas disponibles[25]. Por tanto, los profesionales están legalmente sujetos al cumplimiento de este principio, y no puede limitarse a la voluntad del profesional.

Junto con lo anterior, la implementación de la TDC ha evidenciado una serie de beneficios en los pacientes, en los profesionales y también en los sistemas sanitarios[10, 24, 26, 27]:

- ✔ Aumenta la participación de los pacientes.
- ✔ Mejora la comunicación paciente-profesional sanitario.
- ✔ Mayor adherencia a tratamientos.
- ✔ Mejora los resultados biométricos en salud.
- ✔ Aumenta la satisfacción de los pacientes en la atención de salud.

For peer review only - http://bmjopen.bmj.com/site/about/guidelines.xhtml

- Disminuye la preocupación y la ansiedad en los pacientes.
- Reduce el conflicto decisional de los pacientes cuando se enfrentan a tomar una decisión diagnóstica y/o terapéutica.
- Mejora el conocimiento de la enfermedad, las opciones diagnósticas y terapéuticas en los pacientes.
- Aumenta la precisión en la percepción del riesgo.
- Aumenta la elección de opciones más beneficiosas.
- Reduce el uso de tratamientos muy invasivos y costosos.
- Reduce la variabilidad injustificada en la práctica asistencial.
- Contribuye a la racionalización del uso de recursos del sistema sanitario.

For peer review only - http://bmjopen.bmj.com/site/about/guidelines.xhtml

3. Las limitaciones del modelo

Aun existen pocos estudios que realicen un seguimiento prolongado a los pacientes para determinar con certeza cuál es el impacto de su aplicación a largo plazo. Por otro lado, existe la creencia entre los profesionales sanitarios que ellos ya aplican la SDM[28]. Sin embargo, algunos estudios han demostrado que esta presunción no se refleja en la práctica[29,30]. Finalmente, aunque los pacientes tienden a elegir las mismas opciones que si no se hubiese aplicado una SDM, se ha demostrado que, en el caso del cribado para el cáncer de mama, las mujeres valoran casi 5 veces más la reducción de la mortalidad que el riesgo de un sobrediagnóstico[31] y esta diferencia radica en un mayor conocimiento, en la adherencia al acuerdo, y la disminución del conflicto decisional.

For peer review only - http://bmjopen.bmj.com/site/about/guidelines.xhtml

4. ¿Qué habilidades o competencias necesitan los profesionales sanitarios?

Comunicar de forma equilibrada los riesgos y beneficios de cualquier opción terapéutica no es tarea fácil[26], para conseguirlo se han identificado dos tipos de competencias que deben desarrollar los profesionales sanitarios para aplicarlas en la TDC[32]:

4.1 Competencias relacionales

Son las habilidades que proporcionan un ambiente cómodo para que el paciente comparta sus preocupaciones. Para lograrlo el profesional debe tener un interés genuino en querer involucrarse, comprender el punto de vista del paciente y utilizar un lenguaje sencillo.

For peer review only

For peer review only - http://bmjopen.bmj.com/site/about/guidelines.xhtml

Entre las competencias del profesional destacan:

- Realizar una escucha activa.
- Respetar las decisiones tomadas por el paciente.
- Realizar preguntas abiertas.
- Generar en todo momento contacto visual.
- Dejar que los tiempos sean pautados por el paciente.
- Reconocer sus señales emocionales o verbales.
- Usar habilidades comunicacionales: el resumen, la clarificación, el reflejo, la empatía, entre otras[33].

4.2 Competencias de comunicación de riesgo

Son las habilidades que sirven para discutir con el paciente la incertidumbres y comunicar de forma efectiva los riesgos y beneficios de las diferentes opciones. Se debe evaluar la evidencia en relación a cada contexto particular, es decir, considerar los antecedentes personales: historia familiar, historia clínica y factores de riesgo o protectores que podrían aumentar o disminuir los beneficios/daños de las opciones[32].

For peer review only - http://bmjopen.bmj.com/site/about/guidelines.xhtml

Para esto se recomienda evitar el lenguaje técnico, adaptar la cantidad de información a las necesidades actuales del paciente, utilizar diagramas, comprobar la comprensión de la información ofrecida, incorporar valores del paciente a la evidencia, transmitir información objetiva, facilitar la participación y evaluar la información que ya dispone el paciente[34].

En resumen, para poseer una óptima relación con el paciente se resumen en las siguientes habilidades[33] (Figura 4).

Figura 4: Habilidades comunicativas

Escucha	Lenguaje	No verbal	Cultural	Actitudinal
Escucha general y activa	**Verbal:** tono apropiado y adaptado al nivel educativo<br><br>**Escrita:** comunicación clara y uso de material educativo	**Expresivo:** lenguaje corporal y contacto visual<br><br>**Receptivo:** responde a lenguaje corporal y emociones	Adaptar comunicación a la cultura, edad y enfermedad	Respetar las opiniones y derecho de decidir del paciente

Fuente: Adaptación de Laughlin T, Wetmore S, Allen T, Brailovsky C, Crichton T, Bethune C, Donoff M, Lawrence K. 2012[34].

For peer review only - http://bmjopen.bmj.com/site/about/guidelines.xhtml

Si quieres **profundizar en habilidades comunicacionales** revisa los siguientes enlaces:

- Shared decision making and the concept of equipoise: the competences of involving patients in healthcare choices.

  https://bjgp.org/content/bjgp/50/460/892.full.pdf

- The role of physician–patient communication in promoting patient–participatory decision making.

  https://www.ncbi.nlm.nih.gov/pmc/articles/PMC5060521/

- La comunicación médico-paciente: ¿Cuáles son las habilidades efectivas?

  https://scielo.conicyt.cl/pdf/rmc/v138n8/art16.pdf

- Video Decisions Compartides, Generalitat de Catalunya.

  http://decisionscompartides.gencat.cat/ca/inici

For peer review only - http://bmjopen.bmj.com/site/about/guidelines.xhtml

5. ¿Qué opinan los pacientes?

Un estudio desarrollado en España del año 2012 identificó que al 60% de los pacientes les hubiese gustado que el profesional sanitario les pidiese su opinión, aunque no se les animó a hacerlo. Además, la mayoría hubieran deseado recibir más información de la que se les entregó[35]. Otros estudios en el cribado de cáncer de mama han demostrado que sólo entre un 8% a 10% de las mujeres recibieron información respecto al sobrediagnóstico[36].

Los pacientes consideran que el profesional sanitario debe involucrarse en las decisiones de sus pacientes, es decir, no abandonarlos en el proceso de decisión[37]. En 2013, otro estudio determinó cuáles son los elementos que más valoran los pacientes en la TDC, siendo los más significativos el rol comunicativo del profesional sanitario, la percepción de una escucha comprensiva, la sensación de una preocupación real por su salud y por sus necesidades, una conversación acorde al contexto y la constatación de un dominio de la información[38].

For peer review only - http://bmjopen.bmj.com/site/about/guidelines.xhtml

La TDC fortalece la relación profesional sanitario-paciente y la alianza terapéutica porque el hecho de participar activamente en las decisiones de su salud, disminuye la incertidumbre, aumenta el conocimiento y la posibiidad de manejar mejor su enfermedad[37]. En definitiva, los pacientes sienten mayor tranquilidad.

For peer review only - http://bmjopen.bmj.com/site/about/guidelines.xhtml

For peer review only

For peer review only - http://bmjopen.bmj.com/site/about/guidelines.xhtml

La toma de decisiones compartida en el cribado de cáncer de mama

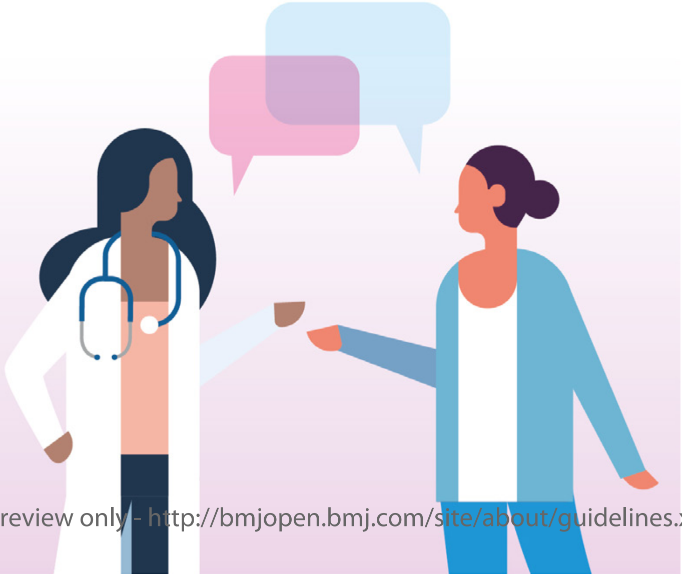

For peer review only - http://bmjopen.bmj.com/site/about/guidelines.xhtml

For peer review only

1. El Programa de cribado

1.1 Cribado poblacional en Cataluña

El Plan Estratégico del *Pla Director d'Oncologia* de la Generalitat de Catalunya menciona como objetivo disminuir el impacto del cáncer de mama en la población mediante el cribado[39]. Sin embargo, no hace referencia a la forma de incorporar a la mujer en las decisiones de su salud, a pesar de que su participación es uno de los pilares del Plan de Salud de Cataluña 2016-2020[40].

Actualmente, cada Programa dependiente del *Pla Director d'Oncologia*, se realiza cada dos años y en base al Registro Central de Asegurados (RCA), la captación de las mujeres entre los 50 y los 69 años mediante una carta dirigida a su domicilio particular. En ella, se les invita a realizar, de forma gratuita, una mamografía en un centro de salud previamente asignado[41]. Este mecanismo no incorpora un espacio de contacto entre el profesional sanitario y la mujer, donde ella pueda resolver sus dudas o inquietudes, ni permite ofrecerle información su-

For peer review only - http://bmjopen.bmj.com/site/about/guidelines.xhtml

ficiente para hacerla partícipe en la decisión sobre su participación -o no- en el programa de cribado.

Para subsanar esta falta de contacto se requiere un cambio en la organización y en los medios de información a las mujeres[42]. Así, el uso de HATD ha demostrado ser un apoyo para el profesional sanitario y para las mujeres en el momento de tomar una decisión conjunta respecto al cribado.

Si quieres **profundizar en HATD** revisa los siguientes enlaces:

- Documento *Desarrollo de Herramientas de Ayuda para la Toma de Decisiones Compartida derivadas de las recomendaciones de las Guías de Práctica Clínica.*
- Sitios web PyDeSalud: *https://pydesalud.com/toma-de-decisiones-compartidas/*
- Sitio web de The Ottawa Hospital: *https://decisionaid.ohri.ca/AZsearch.php?criteria=screening*
- Video demostrativo *Una Demostración - Toma de decisiones compartidas - Mayo Clinic:* *https://www.youtube.com/watch?v=qwyx7yAP5zA&t=4s*

For peer review only - http://bmjopen.bmj.com/site/about/guidelines.xhtml

1.2 ¿Por qué aplicar la TDC al cribado de cáncer de mama?

Las pruebas diagnósticas para la detección precoz del cáncer de mama han tomado fuerza como estándar de salud pública al reconocer la reducción de la mortalidad, así de cada 200 mujeres que se realizan mamografías de cribado cada dos años, entre los 50 y los 69 años, 1 mujer se salva gracias a la detección precoz del tumor y 40 necesitarán pruebas adicionales para descartar el cáncer[13]. Sin embargo, estudios recientes demuestran que existe una escasa o nula percepción de sus daños o efectos adversos.

Los principales riesgos atribuidos al cribado de cáncer de mama son los falsos positivos, falsos negativos y el sobrediagnóstico[19]. Este último concepto se define como aquellos tumores que crecen tan lentamente que nunca llegarían a ser un problema de salud e incluso desaparecerían de forma espontánea, sin necesidad de tratamiento. Actualmente, se desconoce qué tipo de lesiones progresarán y cuáles no, por tanto, se ofrece tratamiento

For peer review only - http://bmjopen.bmj.com/site/about/guidelines.xhtml

a todas las mujeres diagnosticadas de cáncer de mama (lo que se conoce como sobretratamiento)[13] que provoca una sensación de fragilidad y vulnerabilidad en la mujer, intolerancia a la incertidumbre, vinculación a procedimientos de mayor riesgo, como son las biopsias[43] y, finalmente, se traduce en un aumento del gasto sanitario[8].

Ante la incertidumbre entre los beneficios y efectos adversos, se recomienda el uso de TDC con el fin de definir en base a la evidencia científica actual y los valores de las mujeres la decisión de participar o no en el cribado.

Para mayor información sobre las definiciones, incidencia, estadísticas de riesgo, beneficios y efectos adversos del cribado de cáncer de mamas se pueden encontrar en la HATD desarrollada el año 2016[13].

For peer review only

2. Implementación de la TDC en cribado de cáncer de mama

Antes de iniciar el proceso de TDC se debe establecer una relación de confianza basada en la empatía, facilitar una comunicación fluida y de calidad[23,44]. El proceso debe ser deliberativo[21], es decir, las mujeres toman conciencia que tomarán una decisión y que puede requerir más de un encuentro clínico[21]. Además, este debe ser dinámico ya que las fases deben adaptarse a las necesidades, inquietudes y prioridades de cada mujer[45].

Una vez proporcionada la información, se debe explorar explícitamente si la mujer desea desempeñar un rol activo o pasivo en la decisión[23,46] de lo contrario puede inducir a un rol pasivo en las mujeres[17,23,46]. Sin embargo, no es impedimento para corroborar durante todo el encuentro clínico el rol que desean desempeñar, ya que éste puede cambiar de uno activo a uno pasivo o viceversa en el transcurso de la conversación.

For peer review only - http://bmjopen.bmj.com/site/about/guidelines.xhtml

Existen algunos factores que promueven la participación, entre el profesional y la mujer, la motivación de los profesionales de la salud para involucrar a la persona en sus decisiones, la percepción de que la TDC producirá un impacto positivo en el proceso clínico, la alta alfabetización de la mujer o el propio deseo de ella de ser parte activa en las decisiones que afectan a su salud[26,47]. Sin embargo, la falta del tiempo en el encuentro clínico, la edad avanzada de las mujeres, personas con dificultad de comunicarse en el idioma del profesional, el bajo nivel socioeconómico de las mujeres, su baja alfabetización[23,26,46,47] y la presencia de patologías de salud mental[23,43] limitan la TDC.

For peer review only - http://bmjopen.bmj.com/site/about/guidelines.xhtml

2.1 Fases y modelo *"Three-talk"*

Las tres fases que componen este modelo son[48]:

FASE 1<br>**Crear equipo**

El principal objetivo de esta fase es comunicar la necesidad de tomar una decisión en equipo, cuyos integrantes son el profesional sanitario y la mujer. Aquí se comunican los objetivos de la decisión, por qué se debe tomar (presencia de factores de riesgo personal) y las alternativas disponibles basadas en la evidencia. El profesional debe enfatizar en que (a) la mujer pude decidir no tomar una decisión en ese momento y solicitar el apoyo de otros actores como familiares u otros especialistas y (b) debe ser receptivo a las reacciones que puede generar en la mujer el enfrentarse a esta decisión. Por tanto, debe recalcar que la acompañará en el proceso hasta que se sienta segura para llevar a cabo la decisión.

For peer review only - http://bmjopen.bmj.com/site/about/guidelines.xhtml

Figura 5: Crear equipo

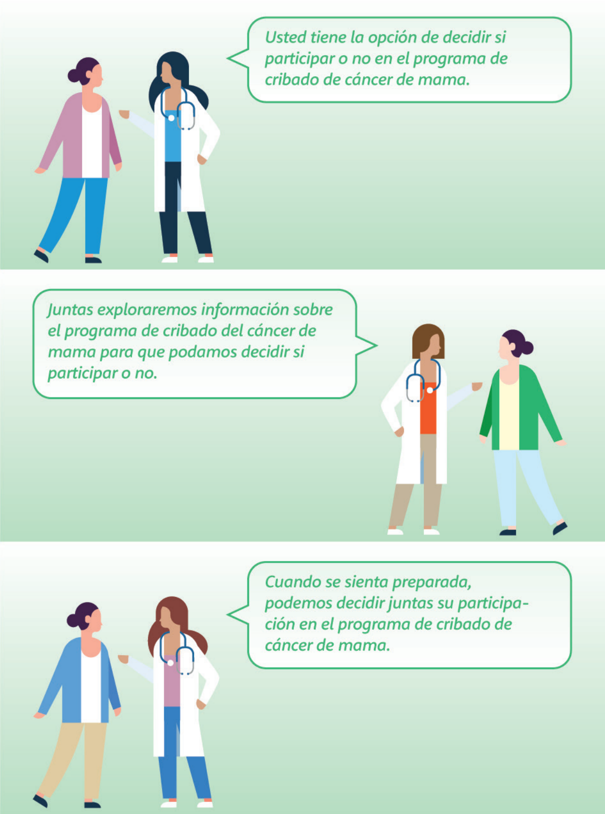

Fuente: Elaboración propia del grupo ProShare.

For peer review only - http://bmjopen.bmj.com/site/about/guidelines.xhtml

FASE 2
Plantear las opciones y explorar preferencias

El principal objetivo de esta fase es informar claramente, según las características de cada mujer, los efectos adversos y beneficios del cribado. Para esto debe explorar sus valores, sus preocupaciones, expectativas y preferencias iniciales (prioridades basadas en los conocimientos preexistentes o ideas preconcebidas respecto al cribado[21]). Además ampliar en detalle las opciones, considerando riesgos y beneficios. De esta forma, las preferencias iniciales pasarán a ser preferencias informadas (preferencias personales basadas en los valores una vez que se ha asegurado la comprensión de los riesgos y beneficios más relevantes del cribado[21]).

Para explicar los riesgos específicos se recomienda utilizar alguna HATD, ya que mejorará la comprensión de la información incluso en mujeres con baja alfabetización[13].

For peer review only - http://bmjopen.bmj.com/site/about/guidelines.xhtml

Figura 6: Plantear las opciones y explorar preferencias

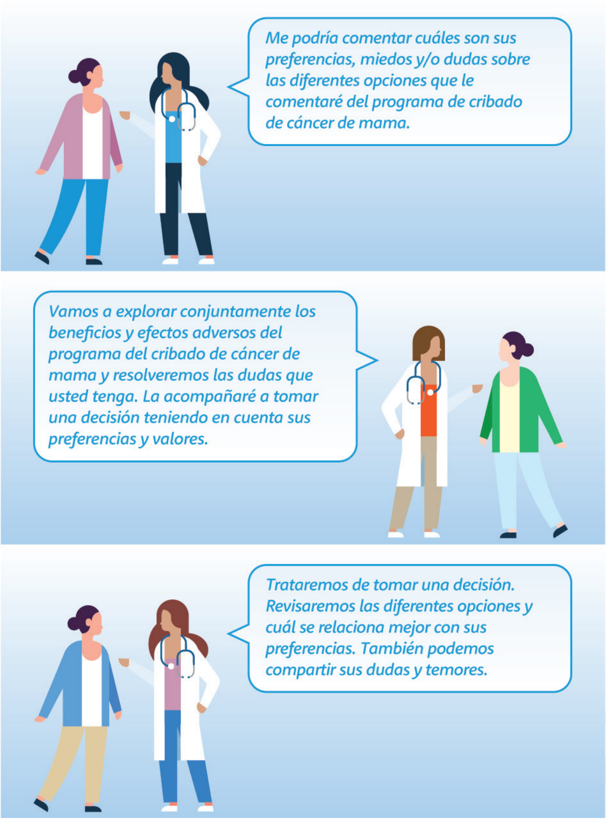

Fuente: Elaboración propia del grupo ProShare.

FASE 3

Tomar una decisión compartida

En esta fase se argumentan las alternativas y se toma una decisión respecto a la participación en el cribado de cáncer de mama[48]. El profesional debe reforzar la idea de acompañamiento en la decisión. Además, debe dar el tiempo suficiente que permita a la mujer reflexionar en torno a sus prioridades, incluso incluyendo la idea de diferirla para otro momento o delegarla en el profesional; en este último caso se recomienda identificar los elementos que le impiden hacerlo por ella misma. Finalmente, debe confirmar la decisión y acordar un plan de acción[23,46] y seguimiento que permita un circuito de retroalimentación entre el profesional y la mujer[45,46].

For peer review only

For peer review only - http://bmjopen.bmj.com/site/about/guidelines.xhtml

Figura 7: Tomar una decisión compartida

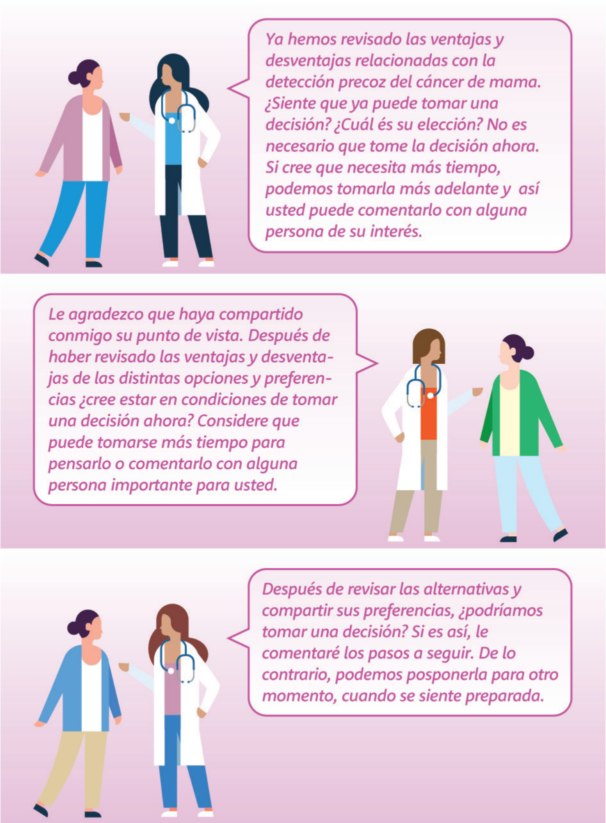

Fuente: Elaboración propia del grupo [illegible]

For peer review only - http://bmjopen.bmj.com/site/about/guidelines.xhtml

Manual de apoyo para implementación de la TDC

38

Figura 8: Fases de la Toma de Decisiones Compartida

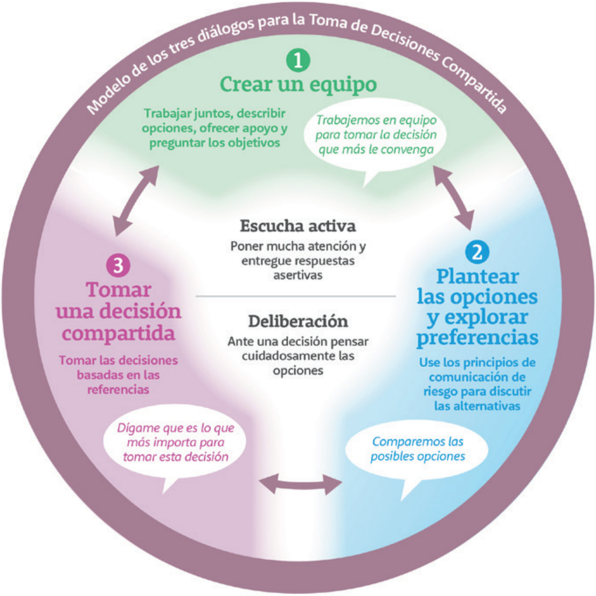

Fuente: Adaptación de Three-talk model of shared decision making. Elwyn G, et al 2017. Uso autorizado por el autor[48].

3. Autoevaluación del proceso de TDC

El instrumento SDM-Q-doc[49] es una encuesta de autoevaluación para profesionales sanitarios que permite medir el nivel de participación que se le ha ofrecido a la mujer para tomar decisiones. Este instrumento está compuesto por nueve ítems que deben ser valorados por el profesional dentro de seis alternativas; desde totalmente en desacuerdo (valor 1) a totalmente de acuerdo (valor 6).

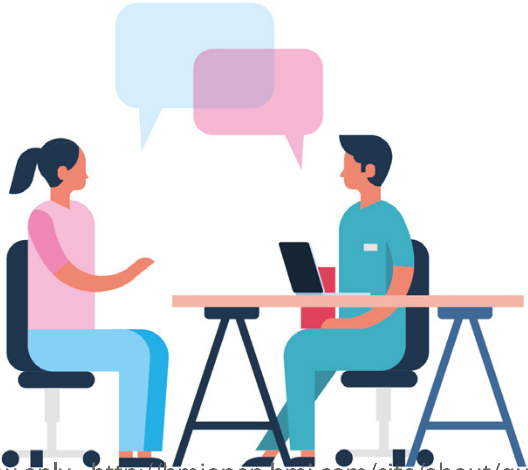

For peer review only - http://bmjopen.bmj.com/site/about/guidelines.xhtml

Criterio	Puntos* 1	2	3	4	5	6
Informé claramente a la mujer de la necesidad de tomar una decisión sobre su participación en el cribado de cáncer de mama	☐	☐	☐	☐	☐	☐
Pregunté a la mujer de forma precisa cómo le gustaría participar en la toma de decisiones	☐	☐	☐	☐	☐	☐
Informé a la mujer que existe la opción de participar o no en el cribado	☐	☐	☐	☐	☐	☐
Expliqué claramente a la mujer las ventajas y desventajas de cada opción	☐	☐	☐	☐	☐	☐
Ayudé a la mujer a entender toda la información sobre beneficios y efectos adversos	☐	☐	☐	☐	☐	☐
Pregunté a la mujer qué opción prefería	☐	☐	☐	☐	☐	☐
La mujer y yo hemos valorado ampliamente todas las opciones	☐	☐	☐	☐	☐	☐
La mujer y yo hemos escogido conjuntamente una opción	☐	☐	☐	☐	☐	☐
La mujer y yo nos hemos puesto de acuerdo sobre el seguimiento de su atención sanitaria posterior	☐	☐	☐	☐	☐	☐

* La puntuación va de: "Totalmente en desacuerdo" (1) a "Totalmente de acuerdo" (6)

Fuente: SDM-Q-doc adaptado al cribado de cáncer de mama.

For peer review only - http://bmjopen.bmj.com/site/about/guidelines.xhtml

Procedimiento y Resultados de la autoevaluación

Para conocer el resultado se deben sumar los puntos obtenidos en cada sección, identificando así las fases del modelo “*Three-talk*”.

Fase de la TDC	Puntos	Interpretación
Fase 1 **“Crear equipo”**		3 a 12 puntos: sin adherencia a la TDC<br>13 a 18 puntos: adherente a la TDC
Fase 2 **“Plantear opciones y explorar preferencias”**		3 a 12 puntos: sin adherencia a la TDC<br>13 a 18 puntos: adherente a la TDC
Fase 3 **“Tomar una decisión”**		3 a 12 puntos: sin adherencia a la TDC<br>13 a 18 puntos: adherente a la TDC
**Puntuación total:**		**9 a 27:** Falta de adherencia a la TDC<br>**28 a 36:** Indiferencia a la TDC<br>**37 a 45:** Leve adherencia a la TDC<br>**46 a 54:** Fuerte adherencia a favor de la TDC

For peer review only - http://bmjopen.bmj.com/site/about/guidelines.xhtml

La interpretación y puntos de corte se basaron en la evidencia de Pollard, Bansback y Bryan (2015)[5]. Se dividieron los puntos totales (54 puntos) según los porcentajes de corte descritos; >80% "Fuerte adherencia del profesional a favor de la TDC"; 60-80% "Leve adherencia del profesional a la TDC"; 40-60% "Indiferencia del profesional a la TDC" y <40% "Falta de adherencia del profesional a la TDC". Por otro lado, los puntos de corte por cada fase definen como "adherente" aquellos superiores al 60% y "sin adherencia" a la TDC el resto, de un total de 15 puntos (3 al 18). Finalmente, la división por fases corresponde a una adaptación de la escala SDM-Q-doc contrastándola con las características del modelo "*Three-talk*"[48].

Se recomienda realizar esta autoevaluación con periodicidad ya que permite a los profesionales sanitarios identificar los puntos fuertes y débiles en cuanto a la forma en que incorporan la participación en salud de las mujeres. De este modo, se facilita focalizar los esfuerzos en mejorar los aspectos más débiles con formación y entrenamiento, y finalmente hacer un seguimiento de los avances conseguidos.

For peer review only

Referencias bibliográficas

1. Organización Mundial de la Salud. (WHO) Control del cáncer de mama; 2020 [citado 30 de junio de 2020]. Available from: https://www.who.int/topics/cancer/breastcancer/es/index3.html

2. Febles G. Revisión de los beneficios del screening del cáncer de mama y análisis de las controversias. *Rev. Imagenol.* [Internet] 2015;19 (1)9-18. Available from: https://www.webcir.org/revistavirtual/articulos/2016/4_noviembre/ur/febles_esp.pdf

3. Day N. Overdiagnosis and breast cancer screening. *Breast Cancer Res* [Internet]. 2005;7(5):228–9. Available from: http://www.ncbi.nlm.nih.gov/pubmed/16168144

4. Nehmat H. Overdiagnosis of breast cancer in population screening: does it make breast screening worthless? *Cancer Biol Med* [Internet]. 2017;14(1):1–8. Available from: http://www.cancerbiomed.org/index.php/cocr/article/view/1003

5. Pollard S, Bansback N, Bryan S. Physician attitudes toward shared decision making: A systematic review. *Patient Educ Couns* [Internet]. 2015; 98(9):1046-57. Available from: https://www.sciencedirect.com/science/article/abs/pii/S0738399115002256?via%3Dihub

6. Munthe C, Sandman L, Cutas D. Person Centred Care and Shared Decision Making: Implications for Ethics, Public Health and Research. *Health Care Anal* [Internet]. 2012;20(3):231–49. Available from: http://link.springer.com/10.1007/s10728-011-0183-y

7. Barry M, Edgman-Levitan S. Shared Decision Making — The Pinnacle of Patient-Centered Care. *N Engl J Med* [Internet]. 2012;366(9):780–1. Available from: http://www.nejm.org/doi/10.1056/NEJMp1109283

8. Coulter A, Collins A. Making shared decision-making a reality. No decision about me, without me [Internet]. The King's Fund. Edwina Rowling, editor. London; 2011. Available from: www.kingsfund.org.uk

9. European patients' forum. 150 Million reasons to act; *EPF's Patients' Manifesto for the European Parliament and Commission*. [Internet]. 2009. Available from: https://www.eu-patient.eu/globalassets/events/old/manifesto/epf_manifesto.pdf

For peer review only - http://bmjopen.bmj.com/site/about/guidelines.xhtml

10. Confederación Crohn y Colitis Ulcerosa. Se incumple el modelo de decisiones compartidas con los pacientes [Internet]. Available from: https://www.accuesp.com/se-incumple-el-modelo-de-decisiones-compartidas-con-los-pacientes

11. Ministerio de Sanidad, Consumo y Bienestar Social de España. Marco Estratégico para la Atención Primaria y Comunitaria [Internet]. 2019. Available from: https://www.mscbs.gob.es/profesionales/proyectosActividades/docs/Marco_Estrategico_APS_25Abril_2019.pdf

12. Agencia de Calidad y Evaluación Sanitarias de Cataluña (AQuAs). Estado de situación de las herramientas de ayuda a la toma de decisiones clínicas en Cataluña. [Internet]. 2020 [citado 30 de junio de 2020]. Available from: http://aquas.gencat.cat/ca/ambits/avaluacio-tecnologies-qualitat/guies/eines_ajut_presa_decisions_catalunya/

13. Toledo-Chavarri A, Rué M, Codern-Bove N, Carles-Lavila M, Perestelo-Perez L, Perez-Lacasta MJ, and Feijoo-Cid M. A Qualitative Study on a Decision Aid for Breast Cancer Screening: Views from Women and Health Professionals. *Eur J Cancer Care (Engl)* [Internet]. 2017. 26(3). Available from: https://onlinelibrary.wiley.com/doi/abs/10.1111/ecc.12660

14. Grupo Español de Investigación en Cáncer de Mama. Guía GEICAM de Práctica Clínica: Para el Diagnóstico y Tratamiento del Cáncer de Mama Metastásico. Edición: Comuniland S.L. Madrid; 2015. Available from: https://portal.guiasalud.es/wp-content/uploads/2019/06/gpc_538_geicam_cancer_mama_resumida.pdf

15. Charles C, Gafni A, Whelan T. Self-reported use of shared decision-making among breast cancer specialists and perceived barriers and facilitators to implementing this approach. *Health Expect* [Internet]. 2004 Dec;7(4):338–48. Available from: http://www.ncbi.nlm.nih.gov/pubmed/15544686

16. Barr P, Scholl I, Bravo P, Faber M, Elwyn G, McAllister M. Assessment of Patient Empowerment - A Systematic Review of Measures. *PLoS One* [Internet]. 2015;10(5). Available from: http://journals.plos.org/plosone/article/file?id=10.1371/journal.pone.0126553&type=printable

For peer review only

For peer review only

17. Elwyn G, Edwards A, Kinnersley P, Grol R. Shared decision making and the concept of equipoise: the competences of involving patients in healthcare choice. *Br J Gen Pract* [Internet]. 2000;50:892–7. Available from: https://bjgp.org/content/bjgp/50/460/892.full.pdf

18. Participa y decide sobre tu salud. ¿Qué es la toma de decisiones compartida? [Internet]. 2020 [citado 30 de junio de 2020]. Available from: https://www.pydesalud.com/toma-de-decisiones-compartidas/

19. Marmot M, Altman D, Cameron D, Dewar J, Thompson S, Wilcox M. The benefits and harms of breast cancer screening: An independent review. *Br J Cancer* [Internet]. 2013;108(11): 2205–2240. Available from: https://www.ncbi.nlm.nih.gov/pmc/articles/PMC3693450/

20. Reyes A. Toma de decisiones compartidas: por una medicina cuidadosa y amable [Internet]. Parte 1. *Por una Medicina Interna de Alto Valor*. 2018. Available from: https://medicinainternaaltovalor.fesemi.org/el-universo-del-paciente/toma-de-decisiones-compartidas-por-una-medicina-cuidadosa-y-amable-parte-1/

21. Elwyn G, Frosch D, Thomson R, Joseph-Williams N, Lloyd A, Kinnersley P, et al. Shared decision making: a model for clinical practice. *J Gen Intern Med* [Internet]. 2012;27(10):1361–7. Available from: http://www.ncbi.nlm.nih.gov/pubmed/22618581

22. Peralta L, Ruiz J, Castillo R. La participación del paciente en la toma de decisiones en las consultas de atención primaria. *Universidad de Cantabria*. 2010; Available from: http://www.tesisenred.net/bitstream/handle/10803/10705/TesisLPM.pdf?sequence=1

23. Información farmacoterapéutica de la Comarca. Participación del paciente en la toma de decisiones. *INFAC* [Internet]. 2014;22(3):12–7. Available from: https://www.euskadi.eus/contenidos/informacion/cevime_infac_2014a/es_def/adjuntos/INFAC_Vol_22_n_3_Decisiones_compartidas.pdf

24. Bravo P, Contreras A. Perestelo-Pérez L, Pérez-Ramos J, Málaga G. En busca de una salud más participativa: Compartiendo decisiones de salud. *Rev Peru Med Exp Salud Publica*. 2013;30(4):691-7.

25. Boletín Oficial del Estado. Ley 14/1986 de 2 de febrero, General de Sanidad. BOE de 21/2000. [Internet]. Available from: https://www.boe.es/boe/dias/2001/02/02/pdfs/A04121-04125.pdf

For peer review only - http://bmjopen.bmj.com/site/about/guidelines.xhtml

For peer review only

26. Gravel K, Légaré F, Graham I. Barriers and facilitators to implementing shared decision-making in clinical practice: a systematic review of health professionals' perceptions. *Implementation Sci*. 2006;1(16). Available from: http://www.implementationscience.com/content/1/1/16

27. Légaré F, Ratté S, Stacey D, Kryworuchko J, Gravel K, Graham ID, et al. Interventions for improving the adoption of shared decision making by healthcare professionals. *Cochrane Database of Systematic Reviews* [Internet]. Chichester, UK: John Wiley & Sons, Ltd; 2010. p. CD006732. Available from: http://www.ncbi.nlm.nih.gov/pubmed/20464744

28. Stiggelbout AM, Pieterse AH, De Haes JC. Shared decision making: Concepts, evidence, and practice. *Patient Educ Couns.* 2015;(10):1172-9. doi: 10.1016/j.pec.2015.06.022. Epub 2015 Jul 15. PMID: 26215573.

29. Hernández-Leal M, Perestelo-Pérez L, Bravo P. Percepción de los profesionales de salud para la Toma de Decisiones Compartida en Atención Primaria. *Revista chilena de medicina familiar*. forthcoming.

30. Couët N, Desroches S, Robitaille H, Vaillancourt H, Leblanc A, Turcotte S, Elwyn G, Légaré F. Assessments of the extent to which health-care providers involve patients in decision making: a systematic review of studies using the OPTION instrument. *Health Expect.* 2015;18(4):542-61. doi: 10.1111/hex.12054.

31. Pérez-Lacastra, Carles-Lavila M, Codern-Bové N, Cardona-Cardona A, Rué-Monné M. Eliciting women's preferences for the design of a breast cancer screening program. *Patient Educ Couns.* forthcoming.

32. Légaré F, Moumjid-Ferdjaoui N, Drolet R, Stacey D, Härter M, Bastian H, et al. Core competencies for shared decision making training programs: insights from an international, interdisciplinary working group. *J Contin Educ Health Prof* [Internet]. 2013;33(4):267–73. Available from: http://www.ncbi.nlm.nih.gov/pubmed/24347105

33. Ford S, Schofield T, Hope T. What are the ingredients for a successful evidence-based patient choice consultation? A qualitative study. *Soc Sci Med* [Internet]. 2003;56:589–602. Available from: https://pubmed.ncbi.nlm.nih.gov/12570976/

For peer review only - http://bmjopen.bmj.com/site/about/guidelines.xhtml

34. Laughlin T, Wetmore S, Allen T, et al. Defining competency-based evaluation objectives in family medicine: communication skills. *Can Fam Physician* [Internet]. 2012;58(4):e217–e224. Available from: https://www.ncbi.nlm.nih.gov/pmc/articles/PMC3325474/.

35. Ruiz R, Peralta L, Pérula L, Olloqui Mundet J, Carrión T, Sobrino A, et al. Opiniones y percepciones de los pacientes sobre su participación en la toma de decisiones en las consultas de medicina de familia. *Aten Primaria* [Internet]. 2012;44(1):5–10. Available from: https://www.sciencedirect.com/science/article/pii/S0212656711001545?via%3Dihub

36. Hersch, J. Jansen & K. McCaffery (2018) Decision-making about mammographic screening: pursuing informed choice, Climacteric, 21:3, 209-213, DOI: 10.1080/13697137.2017.1406912

37. Moreau A, Carol L, Dedianne M, Dupraz C, Perdrix C, Lainé X, et al. What perceptions do patients have of decision making (DM)? Toward an integrative patient-centered care model. A qualitative study using focus-group interviews. *Patient Educ Couns* [Internet]. 2012:1;87(2):206–11. Available from: https://www.sciencedirect.com/science/article/abs/pii/S0738399111004599

38. Thorne S, Oliffe JL, Stajduhar KI. Communicating shared decision-making: Cancer patient perspectives. Patient Educ Couns [Internet]. 2013;90(3):291–6. Available from: https://www.sciencedirect.com/science/article/abs/pii/S0738399112000912

39. Generalitat de Catalunya. Departament de Salut. Pla director 2017. [Internet]. Available from: http://salutweb.gencat.cat/ca/ambits_actuacio/linies_dactuacio/estrategies_salut/cancer/pla_director/

40. Generalitat de Catalunya. Departament de Salut. Pla director D'Oncologia 2016-2020: Un sistema centrat en la persona: públic, universal i just. 2016. [Internet]. Available from: http://salutweb.gencat.cat/web/.content/_departament/pla-de-salut/Pla-de-salut-2016-2020/documents/Pla_salut_Catalunya_2016_2020.pdf

41. Institut Català d'Oncologia. Detecció Precoç de Càncer de Mama. [Internet]. 2020 [citado 30 de junio de 2020]. Available from: http://ico.gencat.cat/ca/el-cancer/programes_de_deteccio_precoc/programa_de_deteccio_precoc_de_cancer_de_mama/index.html

For peer review only

For peer review only - http://bmjopen.bmj.com/site/about/guidelines.xhtml

42. Servicio de epidemiología y Prevención del cáncer (SEPC). Esquema funcional del Programa de Detección Precoz del Cáncer de Mama. [Internet]. 2020 [citado 30 de junio de 2020]. Available from: https://epicancer.cat/prevencion_cancer_mama.

43. Coll-Benejam T, Bravo-Toledo R , Marcos-Calvo M, Astier-Peña M. Impacto del sobrediagnóstico y sobretratamiento en el paciente, el sistema sanitario y la Sociedad. *Aten Primaria.* [Internet]. 2018;50(S2):86-95. Available from: https://www.elsevier.es/es-revista-atencion-primaria-27-articulo-impacto-del-sobrediagnostico-sobretratamiento-el-S0212656718305158

44. Rowland K, Politi M. Shared Decision-Making and the Patient-Provider Relationship. In: Handbook of Health Decision Science [Internet]. New York, NY: Springer New York; 2016. p. 181–92. Available from: http://link.springer.com/10.1007/978-1-4939-3486-7_14

45. Elwyn G, Petronella Catharina Anna Vermunt N. Goal-Based Shared Decision-Making: Developing an Integrated Model. *Journal of Patient Experience*. 2019;1-9. Available from: https://journals.sagepub.com/doi/full/10.1177/2374373519878604

46. Barani M, Kopitowski K. Toma de decisiones compartidas: centrando los cuidados médicos realmente en nuestros pacientes. *Rev Hosp Ital BAires* [Internet]. 2013;33(2):60–4. Available from: https://www.hospitalitaliano.org.ar/multimedia/archivos/noticias_attachs/47/documentos/14745_60-64-HI-33-2-2BARANI_A.pdf

47. Hernández-Leal, MJ, Pérez-Lacasta MJ, Feijoo-Cid M, Ramos-García V, Carles-Lavila M. Healthcare professionals' behaviour regarding the implementation of shared decision-making in screening programmes: A systematic review. Patient Educ Couns [Internet]. 2021. https://doi.org/10.1016/j.pec.2021.01.032

48. Elwyn G, Duran M, Song J, Aarts J, J Barr P, Berger Z, etc al. A three-talk model for shared decision making: multistage consultation process. *BMJ*. 2017;359. [Internet]. Available from: https://www.bmj.com/content/359/bmj.j4891.long

49. De las Cuevas C, Perestelo-Perez L, Rivero-Santana A, Cebolla-Martí A, Scholl I, Härter M. Validation of the Spanish version of the 9-item Shared Decision-Making Questionnaire. *Health Expect* [Internet]. 2015;18(6):2143–53. Available from: https://pubmed.ncbi.nlm.nih.gov/24593044/

For peer review only - http://bmjopen.bmj.com/site/about/guidelines.xhtml

For peer review only

For peer review only

La participación de los profesionales de la salud en la Toma de Decisiones Compartida en el cribado de cáncer de mama

Manual de apoyo para implementación de la Toma de Decisiones Compartida

2021

Unión Europea
Fondo Europeo de Desarrollo Regional
"Una manera de hacer Europa"

MINISTERIO DE CIENCIA, INNOVACIÓN Y UNIVERSIDADES

Instituto de Salud Carlos III

UNIVERSITAT ROVIRA i VIRGILI

ProShare

For peer review only - http://bmjopen.bmj.com/site/about/guidelines.xhtml

cc BY NC

Guía práctica de implementación de la TDC para profesionales sanitarios

1 Crear equipo

Comunicar la necesidad de tomar una decisión

- ↘ Introduzca la posibilidad de tomar decisiones acerca de su salud
- ↘ Comente los factores de riesgo y los que le afectan en particular
- ↘ Resalte que la acompañará en todo momento y puede contar con el apoyo de familiares u otros profesionales

"Usted tiene la opción de decidir si participar o no en el programa de cribado de cáncer de mama."

"Vamos a explorar conjuntamente los beneficios y efectos adversos del programa del cribado de cáncer de mama y resolveremos las dudas que usted tenga. La acompañaré a tomar una decisión teniendo en cuenta sus preferencias y valores"

2 Plantear opciones y explorar preferencias

Informar de la opción de acudir o no a la mamografía

- ↘ Explore los conocimientos de la mujer sobre la mamografía
- ↘ Introduzca efectos adversos y beneficios de la mamografía a través de una Herramienta de Ayuda a la Toma de Decisiones (HATD)
- ↘ Considere las preferencias, creencias, valores y miedos de la mujer sobre la mamografía
- ↘ Resuma las opciones y compruebe si la mujer ha comprendido la nueva información

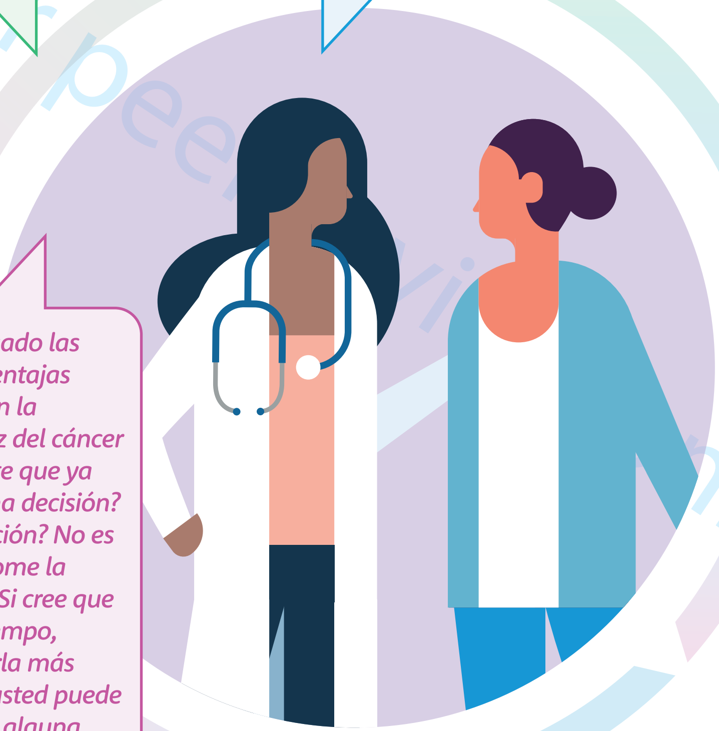

3 Tomar una decisión

Tomar una decisión compartida respecto a la mamografía

- ↘ Dé el tiempo necesario para permitir la reflexión
- ↘ Aclare las dudas y valore las preferencias
- ↘ Diseñe un plan de seguimiento de la decisión

"Ya hemos revisado las ventajas y desventajas relacionadas con la detección precoz del cáncer de mama ¿Siente que ya puede tomar una decisión? ¿Cuál es su elección? No es necesario que tome la decisión ahora. Si cree que necesita más tiempo, podemos tomarla más adelante y así usted puede comentarlo con alguna persona de su interés"

ProShare
SHARED DECISION MAKING IN HEALTH

Competencias Relacionales Transversales

Empatía | Escucha activa | Asertividad | Retroalimentación | Adaptación del lenguaje | Contacto visual

Guía práctica de implementación de la TDC para profesionales sanitarios

ProShare
SHARED DECISION MAKING IN HEALTH

Resultados

Fase de la TDC	Puntos	Interpretación
Fase 1 **"Crear equipo"**		3 a 12 puntos: sin adherencia a la TDC<br>13 a 18 puntos: adherente a la TDC
Fase 2 **"Plantear opcio-nes y explorar preferencias"**		3 a 12 puntos: sin adherencia a la TDC<br>13 a 18 puntos: adherente a la TDC
Fase 3 **"Tomar una decisión"**		3 a 12 puntos: sin adherencia a la TDC<br>13 a 18 puntos: adherente a la TDC
**Puntuación total:**		**9 a 27:** Falta de adherencia a la TDC<br>**28 a 36:** Indiferencia a la TDC<br>**37 a 45:** Leve adherencia a la TDC<br>**46 a 54:** Fuerte adherencia a favor de la TDC

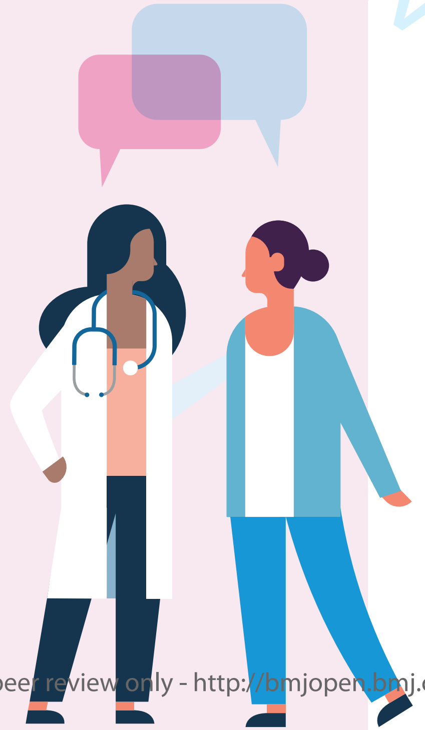

Criterio	Puntos* 1	2	3	4	5	6
Informé claramente a la mujer de la necesidad de tomar una decisión sobre su participación en el cribado de cáncer de mama	☐	☐	☐	☐	☐	☐
Pregunté a la mujer de forma precisa cómo le gustaría participar en la toma de decisiones	☐	☐	☐	☐	☐	☐
Informé a la mujer que existe la opción de participar o no en el cribado	☐	☐	☐	☐	☐	☐
Expliqué claramente a la mujer las ventajas y desventajas de cada opción	☐	☐	☐	☐	☐	☐
Ayudé a la mujer a entender toda la información sobre beneficios y efectos adversos	☐	☐	☐	☐	☐	☐
Pregunté a la mujer qué opción prefería	☐	☐	☐	☐	☐	☐
La mujer y yo hemos valorado ampliamente todas las opciones	☐	☐	☐	☐	☐	☐
La mujer y yo hemos escogido conjuntamente una opción	☐	☐	☐	☐	☐	☐
La mujer y yo nos hemos puesto de acuerdo sobre el seguimiento de su atención sanitaria posterior	☐	☐	☐	☐	☐	☐

* La puntuación va de: "Totalmente en desacuerdo" (1) a "Totalmente de acuerdo" (6)

De las Cuevas C, Perestelo-Perez L, Rivero-Santana A, Cebolla-Martí A, Scholl I, Härter M. Validation of the Spanish version of the 9-item Shared Decision-Making Questionnaire. Health Expect [Internet]. 2015;18(6):2143–53. Available from: http://www.ncbi.nlm.nih.gov/pubmed/24593044

For peer review only - http://bmjopen.bmj.com/site/about/guidelines.xhtml

cc BY NC

La participación de los profesionales de la salud en la Toma de Decisiones Compartida en el cribado de cáncer de mama

Manual de apoyo para implementación de la Toma de Decisiones Compartida

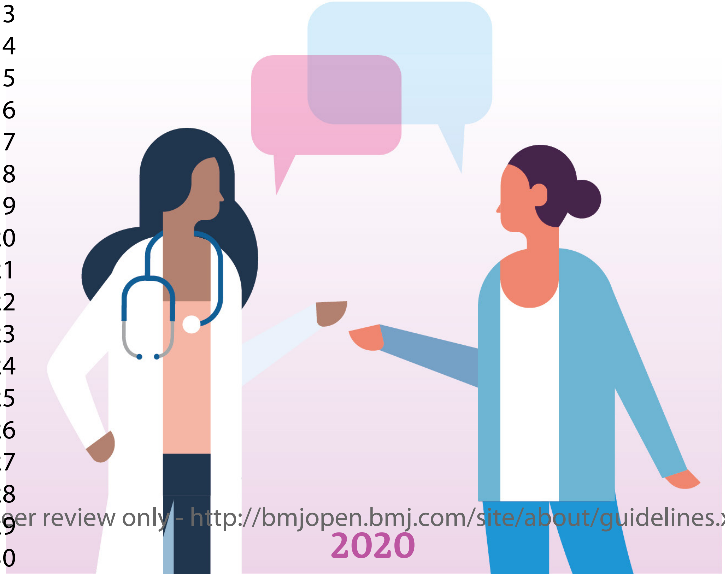

For peer review only - http://bmjopen.bmj.com/site/about/guidelines.xhtml

2020

For peer review only

La participación de los profesionales de la salud en la Toma de Decisiones Compartida en el cribado de cáncer de mama

Manual de apoyo para implementación de la Toma de Decisiones Compartida

2020

Unión Europea
Fondo Europeo de Desarrollo Regional
"Una manera de hacer Europa"
MINISTERIO DE CIENCIA, INNOVACIÓN Y UNIVERSIDADES
Instituto de Salud Carlos III
UNIVERSITAT ROVIRA i VIRGILI
ProShare

For peer review only - http://bmjopen.bmj.com/site/about/guidelines.xhtml

AUTORES

María José Hernández-Leal[1,a,b], María José Pérez-Lacasta[2,a,c], Misericòrdia Carles-Lavila[3,a,b,c] on behalf of the ProShare Group.

1. PhD student, Msc and Bsc Nursing. Mail: mariajose.hernandez@urv.cat
2. PhD, Mail: mariajose.perez@urv.cat
3. PhD. Mail: misericordia.carles@urv.cat

a. Department of Economics, Universitat Rovira i Virgili.
b. Centre de Recerca en Economia i Sostenibilitat (ECO-SOS).
c. Grup de Recerca en Anàlisi Estadístic i Econòmic en Salut (GRAEES).

Equipo de investigación ProShare:

Misericòrdia Carles Lavila
Núria Codern-Bové
María Feijoo
María José Hernández Leal
María José Pérez Lacasta
Roger Pla
Vanesa Ramos García
Carmen Vidal

Cómo citar este documento:

Hernández-Leal MJ, Carles-Lavila M, Pérez-Lacasta M. La participación de los profesionales de la salud en la Toma de Decisiones Compartida en el cribado de cáncer de mama: Manual de apoyo para implementación de la TDC. España: María José Hernández editor; 2020.

For peer review only - http://bmjopen.bmj.com/site/about/guidelines.xhtml

EVALUADORES EXTERNOS

Dra. Lilisbeth Perestelo-Pérez
Servicio de Evaluación y Planificación.
Dirección del Servicio Canario de la Salud, España.
Red de Investigación en Servicios de Salud en Enfermedades Crónicas (REDISSEC).

Dr. Victor Montori
Knowledge and Evaluation Research (KER) Unit.
Mayo Clinic - Minnesota, USA.

Dra. Montserrat Rué
Institut de Recerca Biomèdica de Lleida (IRBLLEIDA).
Departament de Ciències Mèdiques Bàsiques.
Universitat de Lleida, España.

DISEÑO

Miquel A. Fernández
www.mafsdisseny.com

FINANCIACIÓN

- The European Regional Development Fund (ERDF). European Union’s Horizon 2020 research and innovation programme under the Marie Skłodowska-Curie grant agreement No. 713679 from the Universitat Rovira i Virgili (URV).
- Proyecto PI18/00773 “La colaboración de los profesionales sanitarios para incluir la toma de decisiones compartida en el programa de cribado de cáncer de mama” financiado por el Instituto de Salud Carlos III y cofinanciado por la Unión Europea (FEDER) “Una manera de hacer Europa”

For peer review only

For peer review only - http://bmjopen.bmj.com/site/about/guidelines.xhtml

For peer review only

For peer review only - http://bmjopen.bmj.com/site/about/guidelines.xhtml

ÍNDICE

OBJETIVO DEL MANUAL 6

¿A QUIÉN VA DIRIGIDO? 6

Introducción 7

Toma de decisiones compartida 13

1. ¿Qué es? 14

2. ¿Por qué es importante? 17

3. ¿Qué habilidades o competencias necesitan los profesionales sanitarios? 19

4. ¿Qué opinan los pacientes? 22

La toma de decisiones compartida en el cribado de cáncer de mama 25

1. El Programa de cribado 26

2. Implementación de la TDC en cribado de cáncer de mama 31

3. Autoevaluación del proceso de TDC 40

Referencias bibliográficas 45

For peer review only

OBJETIVO DEL MANUAL

Este manual pretende ser un documento de referencia que sirva de guía para el desarrollo e implementación de la **Toma de Decisiones Compartida (TDC)** en el cribado del cáncer de mama.

¿A QUIÉN VA DIRIGIDO?

A profesionales sanitarios de la comunidad autónoma de Catalunya relacionados con el cribado de cáncer de mama y que tengan contacto directo con las mujeres que deben participar en el programa.

For peer review only - http://bmjopen.bmj.com/site/about/guidelines.xhtml

Introducción

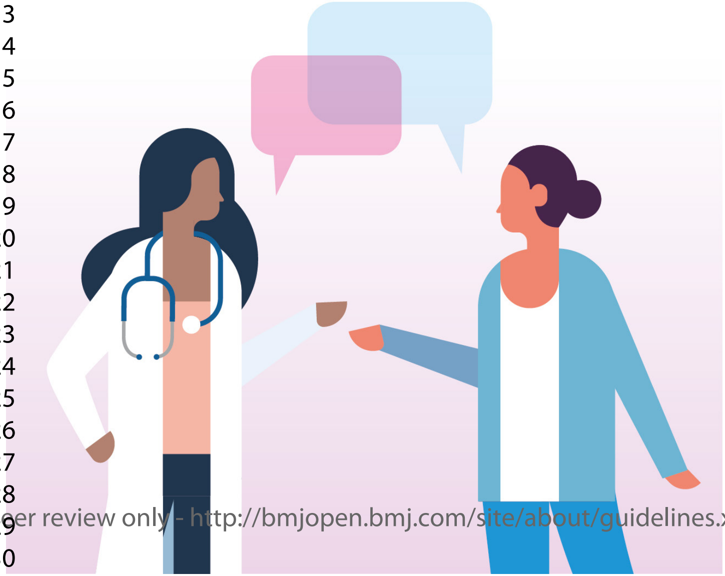

For peer review only - http://bmjopen.bmj.com/site/about/guidelines.xhtml

El objetivo del **cribado de cáncer de mama** es detectar tempranamente el tumor, en una fase preclínica. Esto permite mejorar las opciones de tratamiento y disminuir la mortalidad [1,2]. A pesar de estos beneficios, el cribado puede producir también efectos adversos: falsos negativos, falsos positivos, sobrediagnóstico y sobretratamiento [2,3,4]. Ante el desconocimiento del grado en que afectará los efectos positivos y negativos a cada mujer en la decisión del cribado, el modelo de **Toma de Decisiones Compartida** (TDC) permite que pacientes y profesionales de la salud disminuyan la incertidumbre de la decisión [3,5,6].

La TDC ha sido utilizada principalmente en los países occidentales para mejorar las decisiones en salud[7]. Así, en 2012 The European Patients' Forum inició la campaña *"nothing about me, without me"* (nada sobre mí, sin mí) [8] para involucrar a las personas en las decisiones sobre su salud [9].

Por otro lado, la **Medicina Personalizada** (MP) se ha consolidado desde la segunda mitad del siglo XX[10,11,12], centrando la atención sanitaria en la combi-

For peer review only

or peer review only - http://bmjopen.bmj.com/site/about/guidelines.xhtr

nación de la información clínica, genética y ambiental de cada persona. Al individualizar la atención, los profesionales sanitarios acceden a un enfoque integrado y basado en la evidencia para la medición de riesgo, diagnósticos, aplicación de terapias farmacológicas y manejo clínico. Estas medidas han permitido optimizar la atención clínica según las características de cada individuo[13].

La MP es un modelo de salud que está tomando cada vez más relevancia en las estrategias sanitarias en Europa, por lo que prepararse para su implementación forma parte de los desafíos actuales. La MP se complementa con la TDC ya que ambos modelos buscan posicionar a la persona en el centro de la atención, mejorar su participación en las decisiones de salud y priorizar un enfoque de atención preventiva sobre la curativa. Es decir, mientras que en la MP se utilizan los antecedentes médicos, ambientales y genéticos para estimar resultados clínicos individuales, en la TDC se consideran los valores, preferencias y vivencias de los pacientes[13].

For peer review only - http://bmjopen.bmj.com/site/about/guidelines.xhtml

La aplicación de la MP en los programas de cribado permite [10-12,14]:

- Reducir los efectos adversos (falsos positivos, falsos negativos, sobrediagnóstico y sobretratamiento.
- Aumentar la eficiencia del cribado en todos los subgrupos de riesgo de cáncer de mama.
- Ampliar el periodo y la frecuencia del cribado en las mujeres con riesgo elevado de cáncer de mama y reducir la frecuencia en mujeres con bajo riesgo.
- Informar a las mujeres sobre la detección precoz para que las decisiones que adopten estén mejor sustentadas.
- Ajustar la percepción del riesgo de padecer un cáncer de mama al riesgo real.

For peer review only - http://bmjopen.bmj.com/site/about/guidelines.xhtml

En este contexto, algunas investigaciones han explorado cómo se desarrolla la TDC. Martínez, et al. (2018)[15] identificaron que el 83% de los profesionales creían que las decisiones de detección deberían ser compartidas o centradas en el paciente. Sin embargo, el 77% dedicó menos de 5 minutos para discutir con ellos los beneficios y los riesgos relacionados con la mamografía[16]. Por otro lado, en España solo el 24% de los pacientes afirma haber tomado la decisión conjunta con su profesional sanitario teniendo en cuenta sus características y preferencias personales y sociales[17]. Esto evidencia la falta de estrategias para involucrar a las personas en su salud, posibles deficiencias en las habilidades comunicativas y la inexistencia de vías de diálogo productivas entre los distintos actores del encuentro clínico del cribado de mama[18].

En este sentido, la literatura ha reportado algunas barreras para la implementación de la TDC[19]: falta de tiempo en los encuentros clínicos, falta de conocimientos del profesional sanitario acerca del modelo TDC y de los efectos adversos del cribado, así como la dificultad de los pacientes para tener

For peer review only - http://bmjopen.bmj.com/site/about/guidelines.xhtml

una actuación proactiva[20,21]. La **Agencia de Calidad y Evaluación Sanitarias de Cataluña** (AQuAs) describe las Herramienta de Ayuda a la Toma de Decisiones (HATD) como un elemento central para la TDC, aunque actualmente no dispone de una para el cribado de cáncer de mama[22]. Un estudio reciente desarrolló una HATD en el cribado de cáncer de mama (anexo 1). Su evaluación posterior puso de manifiesto que los profesionales sanitarios y las mujeres valoraron positivamente el intercambio de información equilibrada del cribado mediante una HATD con el fin de mejorar la toma de decisiones en salud[23].

Sin embargo, a pesar de que en la actualidad ha habido un incremento de materiales destinados a las pacientes[24], son escasos aquellos que ayudan a los profesionales para incorporar las preferencias y los valores de las personas en la toma de decisiones en salud. En este sentido, este documento y sus anexos ofrece a los profesionales sanitarios evidencias científicas sobre la TDC para que sea aplicable al proceso de cribado de cáncer de mama.

For peer review only - http://bmjopen.bmj.com/site/about/guidelines.xhtml

Toma de decisiones compartida

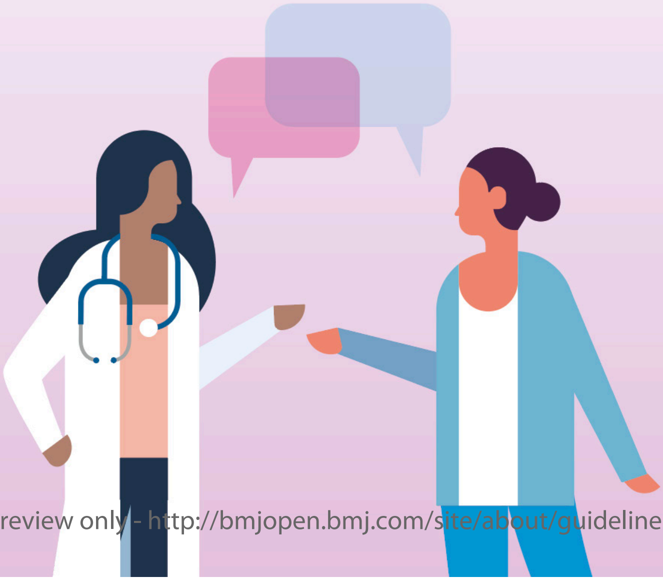

r peer review only - http://bmjopen.bmj.com/site/about/guidelines.xhtr

1. ¿Qué es?

La Toma de Decisiones Compartida (TDC) surge en la década de los 60-70 desarrollándose en mayor medida a partir de los 90. Corresponde a un modelo de atención participativa ubicada entre el estilo de atención paternalista y el informativo [25,26].

Figura 1: Modelos de atención en salud

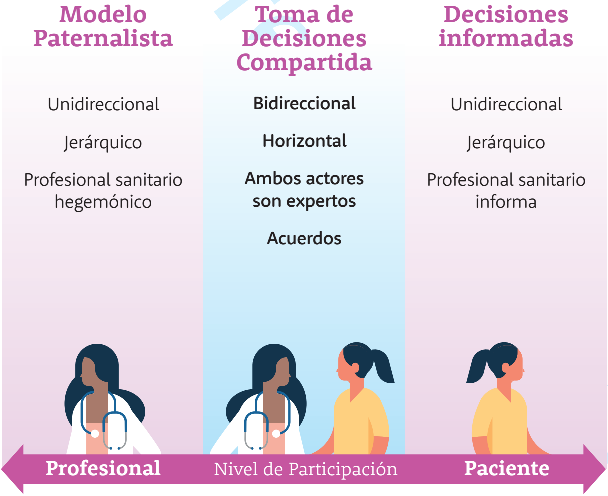

Fuente: Elaboración propia del grupo ProShare

For peer review only - http://bmjopen.bmj.com/site/about/guidelines.xhtml

La TDC fomenta la participación del paciente[27] para tomar una decisión conjunta con el profesional sanitario en relación con cambios en el estilo de vida, pruebas diagnósticas, tratamientos y acciones terapéuticas donde pueda existir algún grado de incertidumbre[28,29]. La TDC se desarrolla durante el encuentro clínico y ambos actores son considerados expertos: el paciente en su situación de salud, valores, creencias y preferencias; y el profesional sanitario en la evidencia científica e información de las opciones terapéuticas disponibles[30].

Figura 2: Rol de los participantes en el encuentro clínico

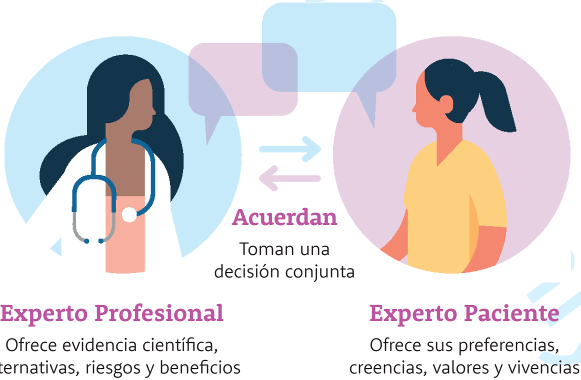

Fuente: Elaboración propia del grupo ProShare

For peer review only - http://bmjopen.bmj.com/site/about/guidelines.xhtml

Por tanto, el objetivo de la TDC se centra en garantizar que las personas tomen decisiones sobre su salud cuando están suficientemente informadas [31]. Para lograrlo se requiere de una negociación continua mediante la discusión entre ambos expertos (paciente y profesional) centrando el diálogo en los valores, preferencias, circunstancias del paciente, así cómo en los beneficios, daños, riesgos y opciones terapéuticas ofrecidas por el profesional sanitario. Como resultado final de esta discusión se consigue que las personas sean más autónomas y presenten un mayor nivel de compromiso y responsabilidad en su salud [(30,32–34)].

Figura 3: Elementos de la Toma de Decisiones Compartida

A) Intercambio de información entre el paciente y el profesional sanitario	B) Deliberación sobre las distintas opciones	**C) Tomar una decisión consensuada**

Fuente: Adaptación de Elwyn et al. 2012 [30]

For peer review only - http://bmjopen.bmj.com/site/about/guidelines.xhtml

2. ¿Por qué es importante?

La TDC se sustenta en el principio de autonomía de los pacientes. La Ley 21/2000 Derechos de Información relativos a la Salud, Autonomía del Paciente y Documentación Clínica protege su derecho a decidir libremente después de recibir la información adecuada entre las opciones clínicas disponibles [35]. Por tanto, los profesionales están sujetos jurídicamente al cumplimiento de este principio, que no puede limitarse al deseo o voluntariedad del profesional.

Asimismo, en Cataluña, el **Modelo Asistencial del Instituto Catalán de Oncología 2019-2022** promueve intervenciones centradas en la persona, teniendo como primer objetivo considerar las necesidades de las personas, para luego planificar el cuidado y atención en salud [36].

Junto con lo anterior, la implementación de la TDC ha evidenciado una serie de beneficios en los pacientes, en los profesionales y también en los sistemas sanitarios [8, 17, 34, 37, 38]:

For peer review only - http://bmjopen.bmj.com/site/about/guidelines.xhtml

- Aumenta la participación de los pacientes.
- Mejora la comunicación paciente-profesional sanitario.
- Mayor adherencia a tratamientos.
- Mejora los resultados biométricos en salud.
- Aumenta la satisfacción de los pacientes en la atención de salud.
- Disminuye la preocupación y la ansiedad en los pacientes.
- Reduce el conflicto decisional de los pacientes cuando se enfrentan a tomar una decisión diagnóstica y/o terapéutica.
- Mejora el conocimiento de la enfermedad, las opciones diagnósticas y terapéuticas en los pacientes.
- Aumenta la precisión en la percepción del riesgo.
- Aumenta la elección de opciones más beneficiosas.
- Reduce el uso de tratamientos muy invasivos y costosos.
- Reduce la variabilidad injustificada en la práctica asistencial.
- Contribuye a la racionalización del uso de recursos del sistema sanitario.

For peer review only - http://bmjopen.bmj.com/site/about/guidelines.xhtml

3. ¿Qué habilidades o competencias necesitan los profesionales sanitarios?

Comunicar de forma equilibrada los riesgos y beneficios de cualquier opción terapéutica no es tarea fácil, pero es necesario ayudar a los pacientes a estar mejor informados [37]; para conseguirlo se han identificado dos tipos de competencias que deben desarrollar los profesionales sanitarios para aplicarlas en la TDC [39]:

3.1 Competencias relacionales

Son las habilidades que favorecen una buena comunicación durante el encuentro clínico y proporcionan un ambiente cómodo que permite al paciente compartir sus preocupaciones. Para lograrlo el profesional debe tener un interés genuino en querer involucrarse y comprender el punto de vista del usuario y utilizar el tipo de lenguaje más adecuado.

For peer review only - http://bmjopen.bmj.com/site/about/guidelines.xhtml

Entre las competencias del profesional destacan:

- Realizar una escucha activa.
- Respetar las decisiones tomadas por el paciente.
- Realizar preguntas abiertas.
- Generar en todo momento contacto visual.
- Dejar que los tiempos sean pautados por el paciente.
- Reconocer sus señales emocionales o verbales.
- Usar habilidades comunicacionales: el resumen, la clarificación, el reflejo, la empatía, entre otras [40].

3.2 Competencias de comunicación de riesgo

Son las habilidades de los profesionales sanitarios que sirven para discutir con el paciente la incertidumbre que existe sobre las opciones terapéuticas y comunicar de forma efectiva los riesgos y beneficios de las diferentes opciones. Se debe evaluar la evidencia en relación con cada contexto en particular, es decir, considerar los antecedentes personales: historia familiar, historia clínica y factores de riesgo o protectores que podrían aumentar o disminuir los beneficios/daños de algunas opciones [19].

For peer review only - http://bmjopen.bmj.com/site/about/guidelines.xhtml

Para esto se recomienda evitar el lenguaje técnico, adaptar la cantidad de información a las necesidades actuales del paciente, utilizar diagramas, comprobar la comprensión de la información ofrecida, incorporar valores del paciente a la evidencia, transmitir información objetiva, facilitar la participación y evaluar la información de la que ya dispone el paciente [41].

Como resumen, las habilidades comunicativas necesarias para poseer una óptima relación con el paciente se pueden resumir en cinco categorías [40] (Figura 4).

Figura 4: Habilidades comunicativas

Escucha	Lenguaje	No verbal	Cultural	Actitudinal
Escucha general y activa	**Verbal:** tono apropiado y adaptado al nivel educativo<br><br>**Escrita:** comunicación clara y uso de material educativo	**Expresivo:** lenguaje corporal y contacto visual<br><br>**Receptivo:** responde a lenguaje corporal y emociones	Adaptar comunicación a la cultura, edad y enfermedad	Respetar las opiniones y derecho de decidir del paciente

Fuente: Adaptación de Laughlin T, Wetmore S, Allen T, Brailovsky C, Crichton T, Bethune C, Donoff M, Lawrence K. 2012 [illegible].

For peer review only - http://bmjopen.bmj.com/site/about/guidelines.xhtml

4. ¿Qué opinan los pacientes?

En un estudio desarrollado en España el 2012, usuarios de Atención Primaria con distintas patologías, valoraron una serie de elementos como positivos en la atención clínica: entrega de información, comunicación de los efectos adversos y beneficios, recomendaciones por parte del profesional, posibilidad de participar activamente en la decisión y la percepción de habilidades comunicativas básicas de los profesionales sanitarios. En este mismo estudio se identificó que al 60% de los pacientes les hubiese gustado que el profesional sanitario les pidiese su opinión, aunque no se les animó a hacerlo. Además, la mayoría hubieran deseado recibir más información de la que se les entregó [42]. En relación con la entrega de información, estudios internacionales sobre cribado de cáncer de mama han demostrado que solo entre un 8% a 10% de las mujeres recibieron información respecto al sobrediagnóstico [43].

Los pacientes sienten mayor tranquilidad, se fortalece la relación profesional sanitario-paciente

For peer review only - http://bmjopen.bmj.com/site/about/guidelines.xhtml

y la alianza terapéutica al participar activamente en las decisiones de su salud, ya que aumentan sus conocimientos, disminuye la incertidumbre y aumenta la posibilidad de manejar mejor su enfermedad. Además, las personas consideran que el profesional sanitario debe involucrarse en las decisiones de sus pacientes, es decir, no abandonarlos en el proceso de decisión, presentarles las opciones disponibles y brindarles asesoramiento, a pesar de que la última decisión sea la del paciente [44].

En 2013, otro estudio determinó cuáles son los elementos que más valoran los pacientes en la TDC, siendo los más significativos el rol comunicativo del profesional sanitario, la percepción de una escucha comprensiva, la sensación de una preocupación real por su salud y por sus necesidades, una conversación acorde al contexto y la constatación de un dominio de la información [45]. Finalmente, la evidencia también concluye que aún falta un largo camino de empoderamiento por el cual las personas fortalezcan su capacidad de ejercer autonomía en el autocuidado de su salud [42].

For peer review only - http://bmjopen.bmj.com/site/about/guidelines.xhtml

For peer review only

For peer review only - http://bmjopen.bmj.com/site/about/guidelines.xhtml

La toma de decisiones compartida en el cribado de cáncer de mama

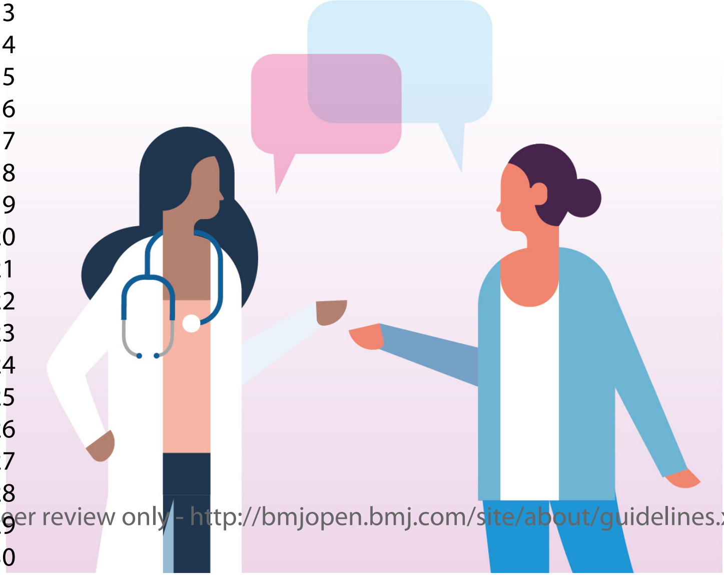

For peer review only - http://bmjopen.bmj.com/site/about/guidelines.xhtml

1. El Programa de cribado

1.1 Cribado poblacional en Cataluña

El Plan Estratégico del *Pla Director d'Oncologia* de la Generalitat de Catalunya menciona como objetivo disminuir el impacto del cáncer de mama en la población mediante el cribado [46]. Sin embargo, no se hace referencia a la forma de incorporar a la mujer en las decisiones de su salud, a pesar de que su participación es uno de los pilares del Plan de Salud de Cataluña 2016-2020 [47].

Actualmente, cada Programa dependiente del *Pla Director d'Oncologia* realiza, cada dos años y en base al Registro Central de Asegurados (RCA), la captación de las mujeres entre los 50 y los 69 años mediante una carta dirigida a su domicilio particular. En ella, se las invita a realizar, de forma gratuita, una mamografía en un centro de salud previamente asignado [48]. Este mecanismo no incorpora un espacio de contacto entre el profesional sanitario y la mujer, en el que ella pueda resolver sus dudas o inquietudes, ni permite

For peer review only - http://bmjopen.bmj.com/site/about/guidelines.xhtml

For peer review only

Figura 5: Flujograma del programa de Detección Precoz del Cáncer de Mama

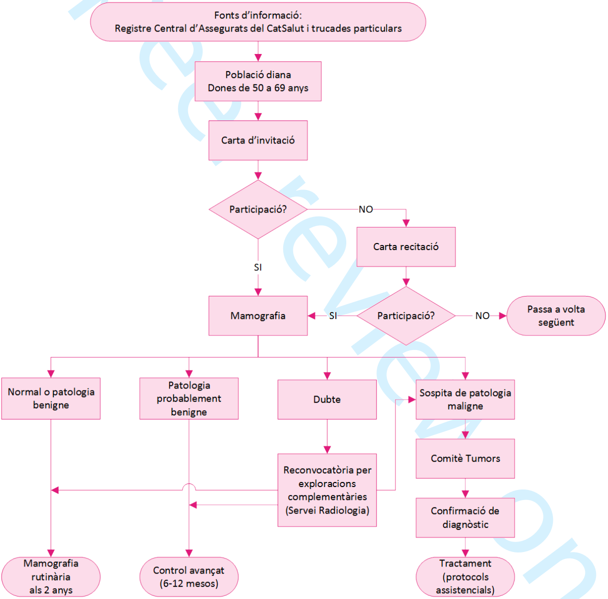

Fuente: Elaborado por Servicio de Epidemiología y Prevención del cáncer (SEPC)[49]

For peer review only

ofrecerle información suficiente para hacerla partícipe en la decisión sobre su participación -o no- en el programa de cribado.

Para subsanar esta falta de contacto se requiere un cambio en la organización y en los medios de información a las mujeres [49]. Así, el uso de Herramientas de Ayuda a la Toma de Decisiones (HATD) ha demostrado ser un apoyo para el profesional sanitario y para las mujeres en el momento de tomar una decisión conjunta respecto al cribado.

1.2 ¿Por qué aplicar la TDC al cribado de cáncer de mama?

Las pruebas diagnósticas para la detección precoz del cáncer de mama han tomado fuerza como estándar de salud pública al reconocerse, tanto por la comunidad científica como por las mujeres, su influencia sobre la reducción de la mortalidad en un 20% [50] y un incremento de la supervivencia al cáncer de mama que alcanza el 80% a los cinco años [24]. Sin embargo, estudios recientes demues-

For peer review only - http://bmjopen.bmj.com/site/about/guidelines.xhtml

tran que existe una escasa o nula percepción de sus daños o efectos adversos[27]. Resultados, que al menos vienen condicionados por la sensibilidad de la prueba, que pueden variar entre un 61% y el 95%, y su especificidad que varía entre el 80% al 90%[42,51].

Los principales riesgos atribuidos al cribado de cáncer de mama son los falsos positivos, falsos negativos y el sobrediagnóstico[29]. Este último concepto se define como aquellos tumores que crecen tan lentamente que nunca llegarían a ser un problema de salud e incluso desaparecerían de forma espontánea, sin necesidad de tratamiento. Actualmente, se desconoce qué tipo de lesiones progresarán y cuáles no, por tanto, se ofrece tratamiento a todas las mujeres diagnosticadas de cáncer de mama (lo que se conoce como sobretratamiento)[23] que provoca una sensación de fragilidad y vulnerabilidad en la mujer, intolerancia a la incertidumbre, grados de estrés en áreas personal-social-familiar-laboral, vinculación a procedimientos de mayor riesgo, como es el caso de las biopsias[52] y, finalmente, un aumento del gasto

For peer review only - http://bmjopen.bmj.com/site/about/guidelines.xhtml

sanitario[8]. Ante la incertidumbre entre los beneficios y efectos adversos en el cribado de cáncer de mama, se recomienda el uso de TDC con el fin de definir en base a la evidencia científica actual y los valores de las mujeres la decisión de participar o no en el cribado.

La información referente al cribado de cáncer de mama se encuentra resumida en la HATD elaborada el año 2016 (anexo 1). Allí se presenta información obtenida del *United Kingdom National Health Service (NHS)*, respecto a definiciones, incidencia, estadísticas de riesgo, beneficios y efectos adversos de una forma sencilla de comprender para el profesional sanitario y la mujer.

For peer review only - http://bmjopen.bmj.com/site/about/guidelines.xhtml

2. Implementación de la TDC en cribado de cáncer de mama

Antes de iniciar el proceso de TDC es importante construir un ambiente que permita una conversación cercana entre el profesional sanitario y la mujer. En este sentido, se debe establecer una relación de confianza basada en la empatía, para facilitar una comunicación fluida y de calidad [33,53]. El proceso debe ser deliberativo [31], es decir, las mujeres en el cribado de cáncer de mama toman conciencia de que pueden tomar una decisión, lo cual puede requerir más de un encuentro clínico [54]. Además este proceso debe ser dinámico ya que las fases deben adaptarse a las necesidades, inquietudes y prioridades de cada mujer [55].

Una vez proporcionada la información, se debe explorar explícitamente si la mujer desea desempeñar un rol activo o pasivo en la decisión [33,56] ya que si no se hace de esta forma puede inducir a un rol pasivo en las mujeres [27,33,56]. Sin embargo, no es impedimento para corroborar durante todo el encuentro clínico el rol que desean desempeñar, ya que este

Manual de apoyo para implementación de la TDC

31

For peer review only - http://bmjopen.bmj.com/site/about/guidelines.xhtml

For peer review only

puede cambiar de uno activo a uno pasivo o viceversa en el transcurso de la conversación. Además, se debe considerar que existen factores que pueden aumentar o disminuir la participación de las mujeres [33]. Algunos factores, entre otros, que promueven su participación son la motivación de los profesionales de la salud para involucrar a la persona en sus decisiones, la percepción de que la TDC producirá un impacto positivo en el proceso clínico, la alta alfabetización de la mujer [57,58] o el propio deseo de ella de ser parte activa en las decisiones que afectan a su salud [37,57,59-61]. Sin embargo, la falta del tiempo en el encuentro clínico, la edad avanzada de las mujeres, personas inmigrantes y/o con dificultad de comunicarse en el idioma del profesional, el bajo nivel socioeconómico de las mujeres, su baja alfabetización [33,37,56] y la presencia de patologías de salud mental [33,52] limitan la TDC.

For peer review only - http://bmjopen.bmj.com/site/about/guidelines.xhtml

2.1 Fases y modelo *"The three-talk"*

Las tres fases que componen este modelo son [62]:

FASE 1
Crear equipo

El principal objetivo de esta fase es comunicar la necesidad de tomar una decisión en equipo, cuyos integrantes son el profesional sanitario y la mujer. Aquí se comunican los objetivos de la decisión, por qué se debe tomar (presencia de factores de riesgo personal) y las alternativas disponibles basadas en la evidencia que se deberán tener en cuenta. El profesional debe enfatizar en que (a) la mujer pude decidir no tomar una decisión en ese momento y solicitar el apoyo de otros actores como familiares u otros especialistas y (b) debe ser receptivo a las reacciones que puede generar en la mujer el enfrentarse a esta decisión. Por tanto, debe recalcar que la acompañará en el proceso hasta que se sienta segura para llevar a cabo la decisión.

For peer review only - http://bmjopen.bmj.com/site/about/guidelines.xhtml

Figura 6: Crear equipo

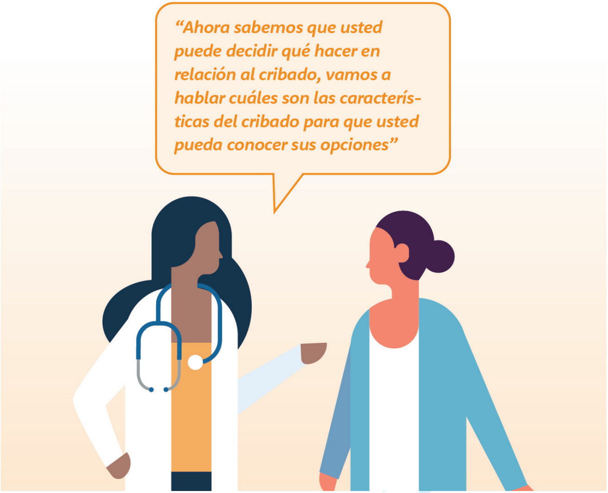

For peer review only - http://bmjopen.bmj.com/site/about/guidelines.xhtml

FASE 2

Plantear las opciones

El principal objetivo de esta fase es informar claramente, y según las características de cada mujer, sobre los efectos adversos y beneficios del cribado. Para esto debe explorar sus preferencias iniciales (prioridades basadas en los conocimientos preexistentes o ideas preconcebidas respecto al cribado[31]), sus valores, sus preocupaciones y expectativas y ampliar en detalle las opciones, considerando riesgos y beneficios. De esta forma, las preferencias iniciales pasarán a ser preferencias informadas (preferencias personales basadas en los valores una vez que se ha asegurado la comprensión de los riesgos y beneficios más relevantes del cribado[31]).

Para explicar los riesgos específicos se recomienda utilizar alguna HATD (anexo 1), ya que mejorarán la comprensión de la información incluso en mujeres con baja alfabetización[23]. En el contexto general, existen dos grandes categorías de HATD; las que se utilizan durante el encuentro clínico de forma con-

For peer review only - http://bmjopen.bmj.com/site/about/guidelines.xhtml

junta profesional sanitario-mujer suelen ser concisas, como los folletos (la que se proporciona en este manual). Las más extensas pueden ser utilizadas antes o después del encuentro clínico, como por ejemplo los documentos, páginas web, etc.[31]

Figura 7: Plantear las opciones

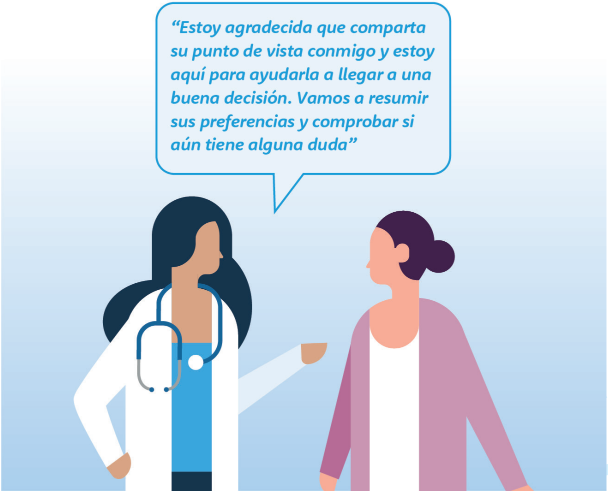

For peer review only - http://bmjopen.bmj.com/site/about/guidelines.xhtml

FASE 3
Tomar una decisión compartida

En esta fase se argumentan las alternativas y se toma una decisión respecto a la participación en el cribado de cáncer de mama [62]. El profesional debe reforzar la idea de acompañamiento en la decisión. Además, debe dar el tiempo suficiente que permita a la mujer reflexionar en torno a sus prioridades, incluso incluyendo la idea de diferirla para otro momento o delegarla en el profesional; en este último caso se recomienda identificar los elementos que le impiden hacerlo por ella misma. Finalmente, debe confirmar la decisión y acordar un plan de acción [33,56] y seguimiento que permita un circuito de retroalimentación entre el profesional y la mujer [55,56].

For peer review only - http://bmjopen.bmj.com/site/about/guidelines.xhtml

Manual de apoyo para implementación de la TDC

38

Figura 8: Tomar una decisión informada

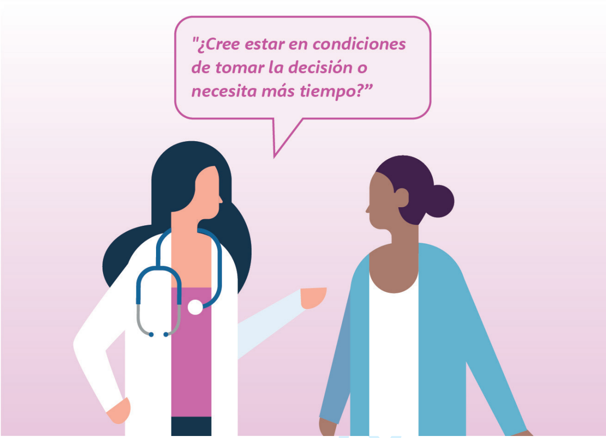

For peer review only - http://bmjopen.bmj.com/site/about/guidelines.xhtml

Figura 9: Fases de la Toma de Decisiones Compartida

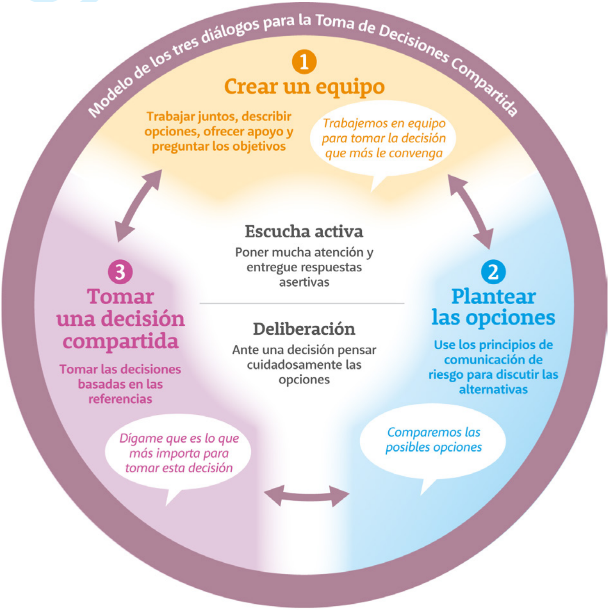

Manual de apoyo para implementación de la TDC

39

Fuente: Adaptación de Three-talk model of shared decision making. Elwyn G, et al 2017. Uso autorizado por autor[62].

For peer review only - http://bmjopen.bmj.com/site/about/guidelines.xhtml

3. Autoevaluación del proceso de TDC

El instrumento SDM-Q-doc[63] es una encuesta de autoevaluación para profesionales sanitarios que permite medir el nivel de participación que se le ha ofrecido a la mujer para tomar decisiones. Este instrumento está compuesto por nueve ítems que deben ser valorados por el profesional dentro de seis alternativas; desde totalmente en desacuerdo (valor 1) a totalmente de acuerdo (valor 6).

For peer review only - http://bmjopen.bmj.com/site/about/guidelines.xhtml

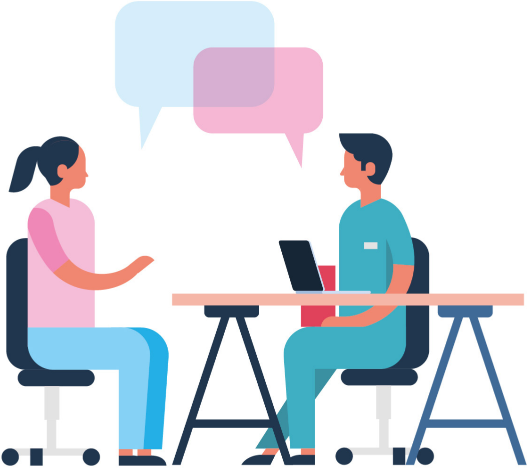

For peer review only - http://bmjopen.bmj.com/site/about/guidelines.xhtml

Criterio	Puntos* 1	2	3	4	5	6
Informé claramente a la mujer de la necesidad de tomar una decisión sobre su participación en el cribado de cáncer de mama	☐	☐	☐	☐	☐	☐
Pregunté a la mujer de forma precisa cómo le gustaría participar en la toma de decisiones	☐	☐	☐	☐	☐	☐
Informé a la mujer que existe la opción de participar o no en el cribado	☐	☐	☐	☐	☐	☐
Expliqué claramente a la mujer las ventajas y desventajas de cada opción	☐	☐	☐	☐	☐	☐
Ayudé a la mujer a entender toda la información sobre beneficios y efectos adversos	☐	☐	☐	☐	☐	☐
Pregunté a la mujer qué opción prefería	☐	☐	☐	☐	☐	☐
La mujer y yo hemos valorado ampliamente todas las opciones	☐	☐	☐	☐	☐	☐
La mujer y yo hemos escogido conjuntamente una opción	☐	☐	☐	☐	☐	☐
La mujer y yo nos hemos puesto de acuerdo sobre el seguimiento de su atención sanitaria posterior	☐	☐	☐	☐	☐	☐

* La puntuación va de: "Totalmente en desacuerdo" (1) a "Totalmente de acuerdo" (6)

Fuente: SDM-Q-doc adaptado al cribado de cáncer de mama.

For peer review only - http://bmjopen.bmj.com/site/about/guidelines.xhtml

Procedimiento y Resultados de la autoevaluación

Para conocer el resultado se deben sumar los puntos obtenidos en cada sección, identificando así las fases del modelo “*The three-talk*”.

Fase de la TDC	Puntos	Interpretación
Fase 1 **“Crear equipo”**		13 a 18 puntos: adherente a la TDC<br>3 a 12 puntos: sin adherencia a la TDC
Fase 2 **“Plantear opciones”**		13 a 18 puntos: adherente a la TDC<br>3 a 12 puntos: sin adherencia a la TDC
Fase 3 **“Tomar una decisión”**		13 a 18 puntos: adherente a la TDC<br>3 a 12 puntos: sin adherencia a la TDC
**Puntuación total:**		**46 a 54:** Fuerte adherencia a favor de la TDC<br>**37 a 45:** Leve adherencia a la TDC<br>**28 a 36:** Indiferencia a la TDC<br>**9 a 27:** Falta de adherencia a la TDC

For peer review only - http://bmjopen.bmj.com/site/about/guidelines.xhtml

La interpretación y puntos de corte se basaron en la evidencia de Pollard, Bansback y Bryan (2015) [64]. Se dividieron los puntos totales (54 puntos) según los porcentajes de corte descritos; >80% "Fuerte adherencia del profesional a favor de la TDC"; 60-80% "Leve adherencia del profesional a la TDC"; 40-60% "Indiferencia del profesional a la TDC" y <40% "Falta de adherencia del profesional a la TDC". Por otro lado, los puntos de corte por cada fase definen como "adherente" aquellos superiores al 60% y "sin adherencia" a la TDC el resto, de un total de 15 puntos (3 al 18). Finalmente, la división por fases corresponde a una adaptación de la escala SDM-Q-doc contrastándola con las características del modelo "*Three-talk*" [61].

Se recomienda realizar esta autoevaluación con cierta periodicidad ya que permite a los profesionales sanitarios identificar los puntos fuertes y deficientes en cuanto a la forma en que incorporan la participación en salud de las mujeres. De este modo, se facilita focalizar los esfuerzos en mejorar los aspectos más débiles con formación y entrenamiento, y finalmente hacer un seguimiento de los avances conseguidos.

For peer review only - http://bmjopen.bmj.com/site/about/guidelines.xhtml

For peer review only

Referencias bibliográficas

1. Organización Mundial de la Salud. (WHO) Control del cáncer de mama; 2020 [citado 30 de junio de 2020]. Available from: https://www.who.int/topics/cancer/breastcancer/es/index3.html

2. Febles G. Revisión de los beneficios del screening del cáncer de mama y análisis de las controversias. *Rev. Imagenol.* [Internet] 2015;19 (1)9-18. Available from: https://www.webcir.org/revistavirtual/articulos/2016/4_noviembre/ur/febles_esp.pdf

3. Day N. Overdiagnosis and breast cancer screening. *Breast Cancer Res* [Internet]. 2005;7(5):228–9. Available from: http://www.ncbi.nlm.nih.gov/pubmed/16168144

4. Nehmat H. Overdiagnosis of breast cancer in population screening: does it make breast screening worthless? *Cancer Biol Med* [Internet]. 2017;14(1):1–8. Available from: http://www.cancerbiomed.org/index.php/cocr/article/view/1003

5. Pollard S, Bansback N, Bryan S. Physician attitudes toward shared decision making: A systematic review. *Patient Educ Couns* [Internet]. 2015; 98(9):1046-57. Available from: https://www.sciencedirect.com/science/article/abs/pii/S0738399115002256?via%3Dihub

6. Munthe C, Sandman L, Cutas D. Person Centred Care and Shared Decision Making: Implications for Ethics, Public Health and Research. *Health Care Anal* [Internet]. 2012;20(3):231–49. Available from: http://link.springer.com/10.1007/s10728-011-0183-y

7. Barry M, Edgman-Levitan S. Shared Decision Making — The Pinnacle of Patient-Centered Care. *N Engl J Med* [Internet]. 2012;366(9):780–1. Available from: http://www.nejm.org/doi/10.1056/NEJMp1109283

8. Coulter A, Collins A. Making shared decision-making a reality. No decision about me, without me [Internet]. The King's Fund. Edwina Rowling, editor. London; 2011. Available from: www.kingsfund.org.uk

For peer review only - http://bmjopen.bmj.com/site/about/guidelines.xhtml

9. European patients`forum. 150 Million reasons to act; *EPF's Patients' Manifesto for the European Parliament and Commission*. [Internet]. 2009. Available from: https://www.eu-patient.eu/globalassets/events/old/manifesto/epf_manifesto.pdf

10. Hernández J, Serrano O. La medicina personalizada, la revolución genómica y el Sistema Nacional de Salud. *Rev Cuba Salud Pública* [Internet]. 2014;40(4):379–91. Available from: http://scielo.sld.cu/scielo.php?pid=S0864-34662014000400012&script=sci_arttext&tlng=pt

11. Chan I, Ginsburg G. Personalized Medicine: Progress and Promise. *Annu Rev Genomics Hum Genet* [Internet]. 2011;12:217–61. Available from: https://pubmed.ncbi.nlm.nih.gov/21721939/

12. Medrano-Hernández A. Medicina personalizada: hacia un nuevo modelo en la práctica médica. *Arch Neurocien* [Internet]. 2012;17(2):129–31. Available from: https://www.medigraphic.com/pdfs/arcneu/ane-2012/ane122h.pdf

13. Sala M, Domingo L, Rué M, Comas M, Torá-Rocamora I, Macià F, et al. Investigación en cribado de cáncer de mama: camino hacia estrategias personalizadas y decisiones compartidas. *Rev Senol y Patol Mamar* [Internet]. 2014;27(4):176–82. Available from: https://linkinghub.elsevier.com/retrieve/pii/S0214158214000589

14. Edwards A, Naik G, Ahmed H, Elwyn G, Pickles T, Hood K, et al. Personalised risk communication for informed decision making about taking screening tests. *Cochrane Database Syst Rev* [Internet]. 2013:28;(2). Available from: http://doi.wiley.com/10.1002/14651858.CD001865.pub3

15. Martinez K, Deshpande A, Ruff AL, Bolen S, Teng K, Rothberg M. Are Providers Prepared to Engage Younger Women in Shared Decision-Making for Mammography? *J Womens Health* (Larchmt). 2018;27(1):24–31. Available from: http://www.liebertpub.com/doi/10.1089/jwh.2016.6047

16. Confederación Crohn y Colitis Ulcerosa. Se incumple el modelo de decisiones compartidas con los pacientes [Internet]. Available from: https://www.accuesp.com/se-incumple-el-modelo-de-decisiones-compartidas-con-los-pacientes

For peer review only - http://bmjopen.bmj.com/site/about/guidelines.xhtml

17. Confederación Crohn y Colitis Ulcerosa. Se incumple el modelo de decisiones compartidas con los pacientes [Internet]. 2020 [citado 30 de junio de 2020]. Available from: https://accuesp.com/se-incumple-el-modelo-de-decisiones-compartidas-con-los-pacientes

18. Ministerio de Sanidad, Consumo y Bienestar Social de España. Marco Estratégico para la Atención Primaria y Comunitaria [Internet]. 2019. Available from: https://www.mscbs.gob.es/profesionales/proyectosActividades/docs/Marco_Estrategico_APS_25Abril_2019.pdf

19. Légaré F, Witteman H. Shared decision making: examining key elements and barriers to adoption into routine clinical practice. *Health Aff (Millwood)*. [Internet]. 2013;32(2):276-84 Available from: https://www.healthaffairs.org/doi/pdf/10.1377/hlthaff.2012.1078

20. Légaré F, Ratté S, Gravel K, Graham I. Barriers and facilitators to implementing shared decision-making in clinical practice: Update of a systematic review of health professionals' perceptions. *Patient Educ Couns* [Internet]. 2008;73(3):526–35. Available from: http://www.ncbi.nlm.nih.gov/pubmed/18752915

21. Hernández-Leal MJ, Pérez-Lacasta M, Feijoo-Cid M, Ramos-García V, Carles-Lavila M. Healthcare professionals´ behaviour to implement in shared decision making in screening programs: A systematic review. *Patient Educ Couns*. forthcoming. 2020.

22. Agencia de Calidad y Evaluación Sanitarias de Cataluña (AQuAs). Estado de situación de las herramientas de ayuda a la toma de decisiones clínicas en Cataluña. [Internet]. 2020 [citado 30 de junio de 2020]. Available from: http://aquas.gencat.cat/ca/ambits/avaluacio-tecnologies-qualitat/guies/eines_ajut_presa_decisions_catalunya/

23. Toledo-Chavarri A, Rué M, Codern-Bove N, Carles-Lavila M, Perestelo-Perez L, Perez-Lacasta MJ, and Feijoo-Cid M. A Qualitative Study on a Decision Aid for Breast Cancer Screening: Views from Women and Health Professionals. *Eur J Cancer Care (Engl)* [Internet]. 2017. 26(3). Available from: https://onlinelibrary.wiley.com/doi/abs/10.1111/ecc.12660

For peer review only

For peer review only - http://bmjopen.bmj.com/site/about/guidelines.xhtml

24. Grupo Español de Investigación en Cáncer de Mama. Guía GEICAM de Práctica Clínica: Para el Diagnóstico y Tratamiento del Cáncer de Mama Metastásico. Edición: Comuniland S.L. Madrid; 2015. Available from: https://portal.guiasalud.es/wp-content/uploads/2019/06/gpc_538_geicam_cancer_mama_resumida.pdf

25. Charles C, Gafni A, Whelan T. Self-reported use of shared decision-making among breast cancer specialists and perceived barriers and facilitators to implementing this approach. *Health Expect* [Internet]. 2004 Dec;7(4):338–48. Available from: http://www.ncbi.nlm.nih.gov/pubmed/15544686

26. Barr P, Scholl I, Bravo P, Faber M, Elwyn G, McAllister M. Assessment of Patient Empowerment - A Systematic Review of Measures. *PLoS One* [Internet]. 2015;10(5). Available from: http://journals.plos.org/plosone/article/file?id=10.1371/journal.pone.0126553&type=printable

27. Elwyn G, Edwards A, Kinnersley P, Grol R. Shared decision making and the concept of equipoise: the competences of involving patients in healthcare choice. *Br J Gen Pract* [Internet]. 2000;50:892–7. Available from: https://bjgp.org/content/bjgp/50/460/892.full.pdf

28. Participa y decide sobre tu salud. ¿Qué es la toma de decisiones compartida? [Internet]. 2020 [citado 30 de junio de 2020]. Available from: https://www.pydesalud.com/toma-de-decisiones-compartidas/

29. Marmot M, Altman D, Cameron D, Dewar J, Thompson S, Wilcox M. The benefits and harms of breast cancer screening: An independent review. *Br J Cancer* [Internet]. 2013;108(11): 2205–2240. Available from: https://www.ncbi.nlm.nih.gov/pmc/articles/PMC3693450/

30. Reyes A. Toma de decisiones compartidas: por una medicina cuidadosa y amable [Internet]. Parte 1. *Por una Medicina Interna de Alto Valor*. 2018. Available from: https://medicinainternaaltovalor.fesemi.org/el-universo-del-paciente/toma-de-decisiones-compartidas-por-una-medicina-cuidadosa-y-amable-parte-1/

31. Elwyn G, Frosch D, Thomson R, Joseph-Williams N, Lloyd A, Kinnersley P, et al. Shared decision making: a model for clinical practice. *J Gen Intern Med* [Internet]. 2012;27(10):1361–7. Available from: http://www.ncbi.nlm.nih.gov/pubmed/22618581

For peer review only - http://bmjopen.bmj.com/site/about/guidelines.xhtml

32. Peralta L, Ruiz J, Castillo R. La participación del paciente en la toma de decisiones en las consultas de atención primaria. *Universidad de Cantabria*. 2010; Available from: http://www.tesisenred.net/bitstream/handle/10803/10705/TesisLPM.pdf?sequence=1

33. Información farmacoterapéutica de la Comarca. Participación del paciente en la toma de decisiones. *INFAC* [Internet]. 2014;22(3):12–7. Available from: https://www.euskadi.eus/contenidos/informacion/cevime_infac_2014a/es_def/adjuntos/INFAC_Vol_22_n_3_Decisiones_compartidas.pdf

34. Bravo P, Contreras A. Perestelo-Pérez L, Pérez-Ramos J, Málaga G. En busca de una salud más participativa: Compartiendo decisiones de salud. *Rev Peru Med Exp Salud Publica*. 2013;30(4):691-7.

35. Boletín Oficial del Estado. Ley 14/1986 de 2 de febrero, General de Sanidad. BOE de 21/2000. [Internet]. Available from: https://www.boe.es/boe/dias/2001/02/02/pdfs/A04121-04125.pdf

36. Instituto Catalán de Oncología. 2019. Plan Estratégico 2019-2022. [Internet]. Available from: http://ico.gencat.cat/web/.content/minisite/ico/lico/documents/arxius/PLA-ESTRATEGIC-2019-2022-Original.pdf

37. Gravel K, Légaré F, Graham I. Barriers and facilitators to implementing shared decision-making in clinical practice: a systematic review of health professionals' perceptions. *Implementation Sci*. 2006;1(16). Available from: http://www.implementationscience.com/content/1/1/16

38. Légaré F, Ratté S, Stacey D, Kryworuchko J, Gravel K, Graham ID, et al. Interventions for improving the adoption of shared decision making by healthcare professionals. *Cochrane Database of Systematic Reviews* [Internet]. Chichester, UK: John Wiley & Sons, Ltd; 2010. p. CD006732. Available from: http://www.ncbi.nlm.nih.gov/pubmed/20464744

39. Légaré F, Moumjid-Ferdjaoui N, Drolet R, Stacey D, Härter M, Bastian H, et al. Core competencies for shared decision making training programs: insights from an international, interdisciplinary working group. *J Contin Educ Health Prof* [Internet]. 2013;33(4):267–73. Available from: http://www.ncbi.nlm.nih.gov/pubmed/24347105

For peer review only - http://bmjopen.bmj.com/site/about/guidelines.xhtml

40. Ford S, Schofield T, Hope T. What are the ingredients for a successful evidence-based patient choice consultation? A qualitative study. *Soc Sci Med* [Internet]. 2003;56:589–602. Available from: https://pubmed.ncbi.nlm.nih.gov/12570976/

41. Laughlin T, Wetmore S, Allen T, et al. Defining competency-based evaluation objectives in family medicine: communication skills. *Can Fam Physician* [Internet]. 2012;58(4):e217–e224. Available from: https://www.ncbi.nlm.nih.gov/pmc/articles/PMC3325474/.

42. Ruiz R, Peralta L, Pérula L, Olloqui Mundet J, Carrión T, Sobrino A, et al. Opiniones y percepciones de los pacientes sobre su participación en la toma de decisiones en las consultas de medicina de familia. *Aten Primaria* [Internet]. 2012;44(1):5–10. Available from: https://www.sciencedirect.com/science/article/pii/S0212656711001545?via%3Dihub

43. Hersch, J. Jansen & K. McCaffery (2018) Decision-making about mammographic screening: pursuing informed choice, Climacteric, 21:3, 209-213, DOI: 10.1080/13697137.2017.1406912

44. Moreau A, Carol L, Dedianne M, Dupraz C, Perdrix C, Lainé X, et al. What perceptions do patients have of decision making (DM)? Toward an integrative patient-centered care model. A qualitative study using focus-group interviews. *Patient Educ Couns* [Internet]. 2012:1;87(2):206–11. Available from: https://www.sciencedirect.com/science/article/abs/pii/S0738399111004599

45. Thorne S, Oliffe JL, Stajduhar KI. Communicating shared decision-making: Cancer patient perspectives. Patient Educ Couns [Internet]. 2013;90(3):291–6. Available from: https://www.sciencedirect.com/science/article/abs/pii/S0738399112000912

46. Generalitat de Catalunya. Departament de Salut. Pla director 2017. [Internet]. Available from: http://salutweb.gencat.cat/ca/ambits_actuacio/linies_dactuacio/estrategies_salut/cancer/pla_director/

47. Generalitat de Catalunya. Departament de Salut. Pla director D'Oncologia 2016-2020: Un sistema centrat en la persona: públic, universal i just. 2016. [Internet]. Available from: http://salutweb.gencat.cat/web/.content/_departament/pla-de-salut/Pla-de-salut-2016-2020/documents/Pla_salut_Catalunya_2016_2020.pdf

For peer review only - http://bmjopen.bmj.com/site/about/guidelines.xhtml

48. Institut Català d`Oncologia. Detecció Precoç de Càncer de Mama. [Internet]. 2020 [citado 30 de junio de 2020]. Available from: http://ico.gencat.cat/ca/el-cancer/programes_de_deteccio_precoc/programa_de_deteccio_precoc_de_cancer_de_mama/index.html

49. Servicio de epidemiología y Prevención del cáncer (SEPC). Esquema funcional del Programa de Detección Precoz del Cáncer de Mama. [Internet]. 2020 [citado 30 de junio de 2020]. Available from: https://epicancer.cat/prevencion_cancer_mama.

50. Pérez-Lacasta MJ, Martínez-Alonso M, Garcia M, Sala M, Perestelo-Pérez L, Vidal C, Corden-Bové N, Feijoo-Cid M, Toledo-Chávarri A, Cardona Á, Pons A, Carles-Lacastra M, Rué M. Effect of information about the benefits and harms of mammography on women's decision making: The InforMa randomised controlled trial. *Plos One*. [Internet]. 2019; 14(3). Available from: https://journals.plos.org/plosone/article?id=10.1371/journal.pone.0214057

51. Álvarez M, Cara M. Radiología básica de mama. Capítulo 1. Introducción al diagnóstico por la imagen de la mama. [Internet] 2015. Available from: http://www.sedim.es/nueva/wp-content/uploads/2015/01/Cap%C3%ADtulo_1_Introduccion.pdf . Córdoba, España.

52. Coll-Benejam T, Bravo-Toledo R , Marcos-Calvo M, Astier-Peña M. Impacto del sobrediagnóstico y sobretratamiento en el paciente, el sistema sanitario y la Sociedad. *Aten Primaria*. [Internet]. 2018;50(S2):86-95. Available from: https://www.elsevier.es/es-revista-atencion-primaria-27-articulo-impacto-del-sobrediagnostico-sobretratamiento-el-S0212656718305158

53. Rowland K, Politi M. Shared Decision-Making and the Patient-Provider Relationship. In: Handbook of Health Decision Science [Internet]. New York, NY: Springer New York; 2016. p. 181–92. Available from: http://link.springer.com/10.1007/978-1-4939-3486-7_14

54. Elwyn G, Frosch D, Thomson R, Joseph-Williams N, Lloyd A, Kinnersley P, et al. Shared decision making: a model for clinical practice. *J Gen Intern Med* [Internet]. 2012;27(10):1361–7. Available from: http://www.ncbi.nlm.nih.gov/pubmed/22618581

For peer review only - http://bmjopen.bmj.com/site/about/guidelines.xhtml

55. Elwyn G, Petronella Catharina Anna Vermunt N. Goal-Based Shared Decision-Making: Developing an Integrated Model. *Journal of Patient Experience*. 2019;1-9. Available from: https://journals.sagepub.com/doi/full/10.1177/2374373519878604

56. Barani M, Kopitowski K. Toma de decisiones compartidas: centrando los cuidados médicos realmente en nuestros pacientes. *Rev Hosp Ital BAires* [Internet]. 2013;33(2):60–4. Available from: https://www.hospitalitaliano.org.ar/multimedia/archivos/noticias_attachs/47/documentos/14745_60-64-HI-33-2-2BARANI_A.pdf

57. Guerra C, Jacobs S, Holmes J, Shea J. Are Physicians Discussing Prostate Cancer Screening with Their Patients and Why or Why Not? A Pilot Study. *J Gen Intern Med* [Internet]. 2007;22(7):901–7. Available from: http://www.ncbi.nlm.nih.gov/pubmed/17549576

58. Volk R, Linder S, Kallen M, Galliher J, Spano M, Mullen P, et al. Primary care physicians' use of an informed decision-making process for prostate cancer screening. *Ann Fam Med*. [Internet]. 2013;11(1):67-74. Available from: https://pubmed.ncbi.nlm.nih.gov/23319508/

59. Malli G. [Early detection of prostate cancer by PSA testing: the results of a qualitative study on barriers caused by physicians in Austria implementing informed decision making]. Gesundheitswesen [Internet]. 2013:75(1);22–8. Available from: https://www.thieme-connect.de/products/ejournals/abstract/10.1055/s-0032-1309017

60. Davis K, Haisfield L, Dorfman C, Krist A, Taylor KL. Physicians' attitudes about shared decision making for prostate cancer screening. *Fam Med* [Internet]. 2011;43(4):206-6. Available from: https://pubmed.ncbi.nlm.nih.gov/21499999/

61. Legaré F, St-Jacques S, Gagnon S, Njoya M, Brisson M,Frémont P, Rousseau F. Prenatal screening for Down syndrome: a survey of willingness in women and family physicians to engage in shared decision-making. *Prenat Diagn* [Internet]. 2011; 31:319–26. Available from: https://obgyn.onlinelibrary.wiley.com/doi/10.1002/pd.2624

For peer review only

62. Elwyn G, Duran M, Song J, Aarts J, J Barr P, Berger Z, etc al. A three-talk model for shared decision making: multistage consultation process. *BMJ*. 2017;359. [Internet]. Available from: https://www.bmj.com/content/359/bmj.j4891.long

63. De las Cuevas C, Perestelo-Perez L, Rivero-Santana A, Cebolla-Martí A, Scholl I, Härter M. Validation of the Spanish version of the 9-item Shared Decision-Making Questionnaire. *Health Expect* [Internet]. 2015;18(6):2143–53. Available from: https://pubmed.ncbi.nlm.nih.gov/24593044/

64. Pollard S, Bansback N, Bryan S. Physician attitudes toward shared decision making: A systematic review. *Patient Educ Couns* [Internet]. 2015; 98(9):1046-57. Available from: https://www.sciencedirect.com/science/article/abs/pii/S0738399115002256?via%3Dihub

For peer review only

For peer review only - http://bmjopen.bmj.com/site/about/guidelines.xhtml

La participación de los profesionales de la salud en la Toma de Decisiones Compartida en el cribado de cáncer de mama

Manual de apoyo para implementación de la Toma de Decisiones Compartida

2020

Unión Europea
Fondo Europeo de Desarrollo Regional
"Una manera de hacer Europa"

MINISTERIO DE CIENCIA, INNOVACIÓN Y UNIVERSIDADES

Instituto de Salud Carlos III

UNIVERSITAT ROVIRA i VIRGILI

ProShare

For peer review only - http://bmjopen.bmj.com/site/about/guidelines.xhtml

Guía práctica de implementación de la TDC para profesionales sanitarios

ProShare
SHARED DECISION MAKING IN HEALTH

"Ahora sabemos que usted puede decidir qué hacer en relación al cribado, vamos a hablar cuáles son las característi-cas del cribado para que usted pueda conocer sus opciones"

Comunicar la necesidad de tomar una decisión

1 Crear equipo

- ↘ Introduzca la posibilidad de tomar decisiones acerca de su salud
- ↘ Comente los factores de riesgo y los que le afectan en particular
- ↘ Resalte que la acompañará en todo momento y puede contar con el apoyo de familiares u otros profesionales

Factores de Riesgo Se estima que 1 de cada 8 mujeres en España padecerán cáncer de mamá a lo largo de su vida. Los factores de riesgo son: edad, antecedentes familiares y personales de cáncer de mama, alteraciones de la mama, hormonal, radioterapia y estilo de vida.

Fuente: AECC: https://www.aecc.es/es/ todo-sobre-cancer/tipos-cancer/cancer-mama/mas-informacion/evolucion-cancer-mama

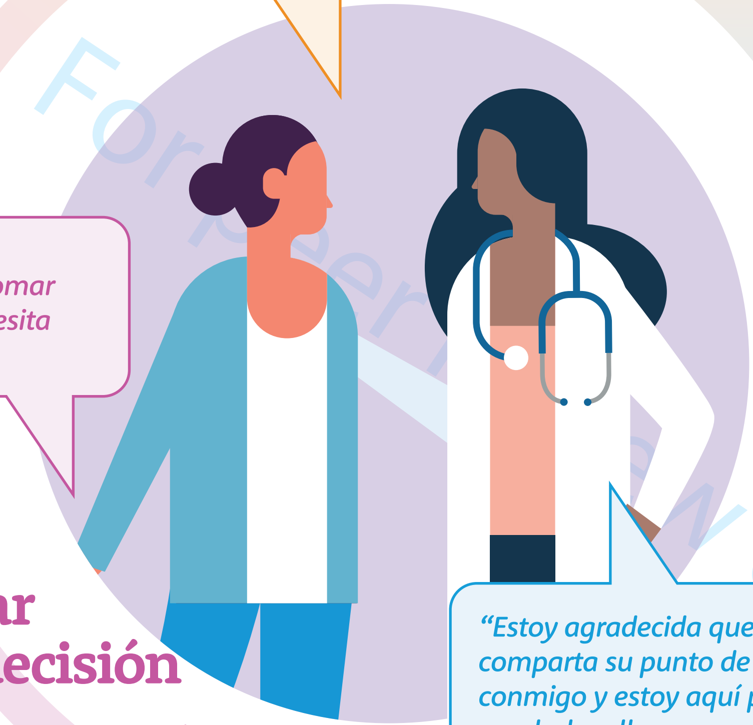

"¿Cree estar en condiciones de tomar la decisión o necesita más tiempo?"

2 Plantear las opciones

Informar de la opción de acudir o no a la mamografía

- ↘ Explore los conocimientos de la mujer sobre la mamografía
- ↘ Introduzca efectos adversos y beneficios de la mamografía a través de una Herramienta de Ayuda a la Toma de Decisiones (HATD)
- ↘ Considere las preferencias, creencias, valores y miedos de la mujer sobre la mamografía
- ↘ Resuma las opciones y compruebe si la mujer ha comprendido la nueva información

"Estoy agradecida que comparta su punto de vista conmigo y estoy aquí para ayudarla a llegar a una buena decisión. Vamos a resumir sus preferencias y comprobar si aún tiene alguna duda"

3 Tomar una decisión

Tomar una decisión compartida respecto a la mamografía

- ↘ Dé el tiempo necesario para permitir la reflexión
- ↘ Aclare las dudas y valore las preferencias
- ↘ Diseñe un plan de seguimiento de la decisión

La Toma de Decisiones Comparti-das (TDC) es un modelo de atención participativo ubicado entre un estilo de atención paternalista e informa-tivo que fomenta la participación de las mujeres para tomar una decisión con el profesional sanitario cuando existe algún grado de incertidum-bre. La TDC se desarrolla durante el encuentro clínico, ambos actores se consideran como expertos: la mujer en su situación de salud, valores, creencias y preferencias. El profesio-nal en la evidencia científica y en cómo dar información de las opcio-nes terapéuticas disponibles.

La mamografia es utilizada como *gold estándar* para el cribado a nivel internacional; ha demostrado disminuir del 20 al 30% la mortalidad, pero también genera falsos negativos, falsos positivos y sobrediagnóstico; su función es adelantar el diagnóstico sin esperar que aparezcan síntomas de la enfermedad. La mamografía es una radiografía de dos proyecciones (cráneo-caudal y látero-oblicua) en cada pecho que posteriormente se analiza con una doble lectura (anexo 1: HATD).

Guía práctica de implementación de la TDC para profesionales sanitarios

ProShare
SHARED DECISION MAKING IN HEALTH

Resultados

Fase de la TDC	Puntos	Interpretación
Fase 1 **"Crear equipo"**	☐	13 a 18 puntos: adherente a la TDC<br>3 a 12 puntos: sin adherencia a la TDC
Fase 2 **"Plantear opciones"**	☐	13 a 18 puntos: adherente a la TDC<br>3 a 12 puntos: sin adherencia a la TDC
Fase 3 **"Tomar una decisión"**	☐	13 a 18 puntos: adherente a la TDC<br>3 a 12 puntos: sin adherencia a la TDC
**Puntuación total:**	☐	**46 a 54:** Fuerte adherencia a favor de la TDC<br>**37 a 45:** Leve adherencia a la TDC<br>**28 a 36:** Indiferencia a la TDC<br>**9 a 27:** Falta de adherencia a la TDC

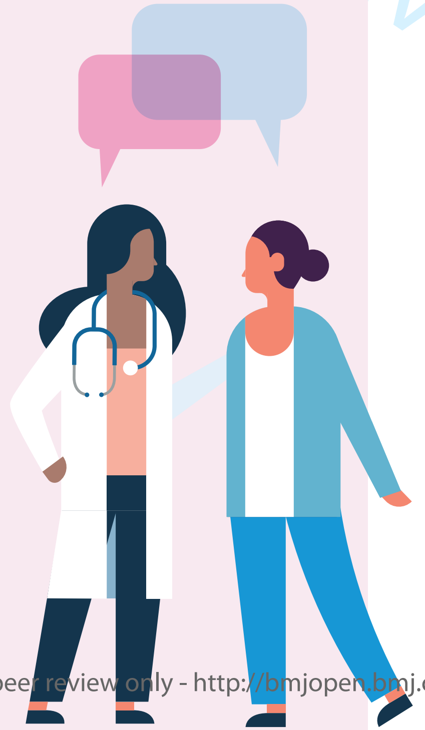

Criterio	Puntos* 1	2	3	4	5	6
Informé claramente a la mujer de la necesidad de tomar una decisión sobre su participación en el cribado de cáncer de mama	☐	☐	☐	☐	☐	☐
Pregunté a la mujer de forma precisa cómo le gustaría participar en la toma de decisiones	☐	☐	☐	☐	☐	☐
Informé a la mujer que existe la opción de participar o no en el cribado	☐	☐	☐	☐	☐	☐
Expliqué claramente a la mujer las ventajas y desventajas de cada opción	☐	☐	☐	☐	☐	☐
Ayudé a la mujer a entender toda la información sobre beneficios y efectos adversos	☐	☐	☐	☐	☐	☐
Pregunté a la mujer qué opción prefería	☐	☐	☐	☐	☐	☐
La mujer y yo hemos valorado ampliamente todas las opciones	☐	☐	☐	☐	☐	☐
La mujer y yo hemos escogido conjuntamente una opción	☐	☐	☐	☐	☐	☐
La mujer y yo nos hemos puesto de acuerdo sobre el seguimiento de su atención sanitaria posterior	☐	☐	☐	☐	☐	☐

* La puntuación va de: "Totalmente en desacuerdo" (1) a "Totalmente de acuerdo" (6)

De las Cuevas C, Perestelo-Perez L, Rivero-Santana A, Cebolla-Martí A, Scholl I, Härter M. Validation of the Spanish version of the 9-item Shared Decision-Making Questionnaire. Health Expect [Internet]. 2015;18(6):2143–53. Available from: http://www.ncbi.nlm.nih.gov/pubmed/24593044

For peer review only - http://bmjopen.bmj.com/site/about/guidelines.xhtml

Title: Development of support material for health professionals who are implementing Shared Decision-Making in breast cancer screening: Validation using Delphi technique

Revised Standards for Quality Improvement Reporting Excellence (SQUIRE 2.0)
September 15, 2015

Text Section and Item Name	Section or Item Description	Page
Notes to authors	• The SQUIRE guidelines provide a framework for reporting new knowledge about how to improve healthcare<br>• The SQUIRE guidelines are intended for reports that describe system level work to improve the quality, safety, and value of healthcare, and used methods to establish that observed outcomes were due to the intervention(s).<br>• A range of approaches exists for improving healthcare. SQUIRE may be adapted for reporting any of these.<br>• Authors should consider every SQUIRE item, but it may be inappropriate or unnecessary to include every SQUIRE element in aparticular manuscript.<br>• The SQUIRE Glossary contains definitions of many of the key words in SQUIRE.<br>• The Explanation and Elaboration document provides specific examples of well-written SQUIRE items, and an in-depth explanation of each item.<br>• Please cite SQUIRE when it is used to write a manuscript.	
Title and Abstract		
1. Title	Indicate that the manuscript concerns an initiative to improve healthcare (broadly defined to include the quality, safety, effectiveness, patient- centeredness, timeliness, cost, efficiency, and equity of healthcare)	1
2. Abstract	a) Provide adequate information to aid in searching and indexing<br><br>b) Summarize all key information from various sections of the text using the abstract format of the intended publication or a structured summary such	2

Title: Development of support material for health professionals who are implementing Shared Decision-Making in breast cancer screening: Validation using Delphi technique

	as: background, local problem, methods, interventions, results, conclusions	
**Introduction**	*Why did you start?*	
3. Problem Description	Neture and significance of the local problem	3
4. Available knowledge	Summary of what is currently known about the problem, including relevant previous studies	3
5. Rationale	Informal or formal frameworks, models, concepts, and/or theories used to explain the problem, any reasons or assumptions that were used to develop the intervention(s), and reasons why the intervention(s) was expected to work	3
6. Specific aims	Purpose of the project and of this report	4
**Methods**	*What did you do?*	
7. Context	Contextual elements considered important at the outset of introducing the intervent io n(s)	4
8. Intervention (s)	a. Description of the intervention(s) in sufficient detail that others could reproduce it<br><br>b. Specifics of the team involved in the work	4
9. Study of the Intervention (s)	a. Approach chosen for assessing the impact of the intervention(s)<br>b. Approach used to establish whether the observed outcomes were due to the intervention(s)	5
10. Measures	a. Measures chosen for studying processes and outcomes of the intervention(s), including rationale for choosing them, their operational definitions, and their validity and reliability<br><br>b. Description of the approach to the ongoing assessment of contextual elements that contributed to the success, failure, efficiency, and cost<br><br>c. Methods employed for assessing completeness and accuracy of data	5
11. Analysis	a. Qualitative and quantitative methods used to draw inferences from the data	5

Title: Development of support material for health professionals who are implementing Shared Decision-Making in breast cancer screening: Validation using Delphi technique

	b. Methods for understanding variation within the data, including the effects of time as a variable	
12. Ethical Considerations	Ethical aspects of implementing and studying the intervention(s) and how they were addressed, including, but not limited to, formal ethics review and potential conflict(s) of interest	5-6
**Results**	*What did you find?*	
13. Results	a. Initial steps of the intervention(s) and their evolution over time (e.g., time-line diagram, flow chart, or table), including modifications made to the intervention during the project<br><br>b. Details of the process measures and outcome<br><br>c. Contextual elements that interacted with the intervention(s)<br><br>d. Observed associations between outcomes, interventions, and relevant<br><br>e. contextual elements<br><br>f. Details about missing data	6-10
**Discussion**	*What does it mean?*	
14. Summary	a. Key findings, including relevance to the rationale and specific aims<br><br>b. Particular strengths of the project	10
15. Interpretation	a. Nature of the association between the intervention(s) and the outcomes<br><br>b. Comparison of results with findings from other publications<br><br>c. Impact of the project on people and systems<br><br>d. Reasons for any differences between observed and anticipated outcomes, including the influence of context<br><br>e. Costs and strategic trade-offs, including opportunity costs	10-12
16. Limitations	a. Limits to the generalizability of the work<br>b. Factors that might have limited internal validity	12

Title: Development of support material for health professionals who are implementing Shared Decision-Making in breast cancer screening: Validation using Delphi technique

	such as confounding, bias, or imprecision in the design, methods, measurement, or analysis<br><br>c. Efforts made to minimize and adjust for limitations	
17. Conclusions	a. Usefulness of the work<br>b. Sustainability<br>c. Potential for spread to other contexts<br>d. Implications for practice and for further study in the field<br>e. Suggested next steps	13
Other information		
18. Funding	Sources of funding that supported this work. Role, if any, of the funding organization in the design, implementation, interpretation, and reporting	13-14

For peer review only
